# Time to control of anthrax outbreaks in Africa, 2014–2023: A systematic review and meta-analysis

**DOI:** 10.1371/journal.pgph.0004534

**Published:** 2025-04-22

**Authors:** Alex R. Ario, Esther Buregyeya, Elizeus Rutebemberwa, Abel W. Walekhwa, Rebecca Akunzirwe, Irene B. Kyamwine, Ronald Olum, Fred Nuwaha, David Serwadda, Rhoda K. Wanyenze

**Affiliations:** 1 National Institute of Public Health, Kampala, Uganda; 2 Makerere University School of Public Health, Kampala, Uganda; 3 Diseases Dynamics Unit, Department of Veterinary Medicine, University of Cambridge, Cambridge, United Kingdom; McGill University, CANADA

## Abstract

Anthrax is a notifiable zoonotic disease targeted for control in Africa, however, outbreaks due to anthrax are still frequent and large. Surveillance systems should monitor and detect anthrax outbreaks early for prompt response. This systematic review and meta-analysis aimed to determine anthrax outbreaks epidemiological investigations gaps and time to control in Africa, Jan 2014-Dec 2023. We searched MEDLINE, PubMed, Scopus, Embase, Google Scholar, and Web of Science databases using PICO framework for studies on anthrax investigations. Using Covidence, we screened and extracted studies, analysed descriptive data using Microsoft Excel and quantitative data using RStudio version 4.3.1. We calculated heterogeneity and confidence intervals around pooled effect and performed risk of bias assessment. Ten of 1,639 studies met eligibility criteria and were included. Pooled median duration to control was 40.5 (IQR 80.8) days and estimated duration of outbreak end was 59.2 days (95% CI: 7.4-111.0), far beyond two incubation periods of anthrax (14 days). Median time to alert was 5 days (95% CI:0–490). A third (30%) didn’t investigate animal anthrax. No study reported use of all levels of case definitions, and no study translated case investigation forms into local languages. A third (30%) of studies omitted time component of descriptive epidemiology and 22% of studies used cross-sectional study design. All studies used epidemiologists for case investigations, with 90% employing field epidemiologists, only one study used a social worker. Only 20% of studies used government funds; majority (80%) instituted public health actions. Risk of bias was at 0–20%. Median duration to control was greater than two anthrax incubation periods indicating delayed response. Several epidemiological gaps including delayed outbreak verification, focus on human anthrax and neglect of zoonotic aspects, and inappropriate working case definitions were highlighted. Timely and comprehensive epidemiological investigations, with a One Health approach to anthrax outbreak control is recommended.

**Systematic Review Registration**: The protocol that guided this review was registered on PROSPERO: CRD42024498034

## Background

Anthrax is a zoonotic disease that primarily affects herbivores but can also incidentally infect humans through contact with infected animals [1]. It is classified as a neglected disease by the World Organization for Animal Health (WOAH) and is considered a significant security risk because of its potential use as a bioweapon or bioterror agent. Anthrax causes high mortality rates in wildlife and livestock and can lead to secondary human cases [2].

The global incidence of anthrax in humans varies widely across countries, ranging from 0.03 per 100,000 inhabitants in Ghana to 1.4 in Georgia [3]. Owing to its hyper-endemicity and frequent outbreaks in Africa, anthrax could be underreported worldwide. An anthrax outbreak reported in Ghana in 2024 affected 13 districts in the Upper East Region of which 100 animals died including 51% sheep, 41% cattle, 6% goats, and 2% pigs [4]). In sub-Saharan Africa, anthrax outbreaks, are frequent in the eco-rich Congo basin region [5–13]). The Congo Basin extends over six nations: Cameroon, the Central African Republic, the Democratic Republic of the Congo, the Republic of the Congo, Equatorial Guinea, and Gabon; bordering several other countries [14]. Like other transboundary animal diseases, the presence of anthrax anywhere poses a threat to other regions; hence the need for early detection and control of outbreaks [15,16].

Detection of anthrax outbreaks in animals and humans through the surveillance system triggers response by national and local government agencies such as ministries of health, veterinary, livestock etc; national public health institutes, and partners etc.. Adoption of the Integrated Disease Surveillance and Response (IDSR) strategy and One Health framework was intended to improve detection and response to notifiable diseases in African countries [17–19]. This informed development of preparedness and response structures which may vary in African countries but are largely similar in functions of detection, notification and response. The structures include: a body that provides oversight, commonly called a National Taskforce (NTF), Public Health Emergency Operations Centres (PHEOCs), Incident Management Teams (IMTs) and Rapid Response Teams (RRTs). RRTs are part of the larger emergency response strategy tasked with detecting, investigating, instituting control measures and risk communicate to the affected community [6,8,20–28].

In addition to the IDSR strategy, countries have adopted the 7:1:7 Framework. The 7:1:7 Framework is a public health strategy used for early detection and rapid response to disease outbreaks, ensuring efficient epidemic control. It is structured around three key time-bound actions: first 7 days – detect and notify a potential public health threat within 7 days of its emergence, 1 day – investigate and confirm the outbreak within 1 day of detection, final 7 days – implement an initial public health response within 7 days of confirmation to contain the outbreak. The 7:1:7 Framework is useful for infectious disease surveillance, emergency response planning, and global health security because it enhances early warning systems for disease outbreaks, improves response speed to minimize spread and impact, and strengthens public health infrastructure by setting clear benchmarks for epidemic preparedness [29].

Large and numerous public health emergencies including anthrax outbreaks with devastating consequences have continued to occur in many parts of Africa despite this setup [21,30–36]. The absence of robust surveillance and detection systems in Africa has resulted in underreporting and significant gaps in epidemic response. Epidemiological investigations are critical components of outbreak detection, notification and control. The field epidemiology training program (FETP) is a proven, strong and practical model for building a sustained capacity for health emergency response work. However, most FETPs in Africa which produce the core field epidemiologists for outbreak investigations rely majorly on donor funding, with minimal government support [37–39]. Strengthening of the surveillance systems including epidemic investigations and response using a One Health approach to curb the spread of anthrax in sub-Saharan Africa is urgently needed [40–42]. The objectives of this systematic review and meta-analysis were to; a) identify gaps in epidemiological investigations of anthrax outbreaks in Africa from January 2014 to December 2023; and b) examine the time to control of anthrax outbreaks in Africa by synthesizing studies conducted from January 2014 to December 2023.

## Materials and methods

### Definitions

**Date of alert** refers to the date when a suspected or confirmed case of Anthrax is officially reported to the health or veterinary authorities. This can be the first notification about the outbreak, which may come from various sources such as healthcare professionals, laboratory professionals or field observations.

**7-1-7** refers to the first real-time, start-to-end assessment of how quickly a country detects and contains infectious disease threats. This framework argues that countries should only take 7 days to detect a suspected infectious disease outbreak. Then take 1 day to notify public health authorities to start an investigation and 7 days to complete an initial response. **Time to control** of an anthrax outbreak is defined as the duration from initial detection of an outbreak (alert) to the last case reported in that outbreak. **Outbreak end/Outbreak controlled/Control of the outbreak** is when effective control measures are instituted, and the spread of the disease is curbed to the level considered a nonpublic health problem. Because one case of anthrax is an outbreak according to the IDSR guidelines, control of an anthrax outbreak is when no case is detected. Time to control metric reflects the timeliness of interventions in containing an outbreak and, as such, helps in assessing effectiveness of response. Epidemiological investigations of anthrax outbreaks are critical contributors to this metric. **Public health actions** are steps taken by the response team, guided by data and evidence generated from the investigation, to control and prevent outbreak spread, ultimately protecting and promoting the health of the affected population. **Team composition** in anthrax outbreak response should include epidemiologists (team leader and field epidemiologists), risk communicators, social workers, laboratory technologists, veterinarians, environmental health officers, and case management clinicians. **Sustainable funding mechanism** should have more government support for capacity building and actual response than donor funding.

### Protocol development and registration

This study followed the Preferred Reporting Items for Systematic Reviews and Meta-Analysis of observational studies and the critical appraisal checklist for systematic reviews, the PRISMA 2020 Checklist (S1 Checklist). We used the Population, Intervention, Comparison, Outcome (PICO) framework to develop focused questions for the systematic review (S1 Table). We registered the study in PROSPERO, the International Prospective Register of Systematic Reviews (CRD42024498034).

### Outcomes of interest

The primary outcome of interest was time to control of anthrax outbreaks, i.e., duration to control from different studies to understand the average time taken for outbreak control, and the secondary outcomes were gaps in epidemiological investigations of anthrax outbreaks in Africa. These include a) verification time (measured by days between alert date and confirmation by lab or identification by case finding), b) response time (measured by the days in between the alert date and the investigation start date, c) case definitions, d) objectives, e) choice of study design, f) investigation team composition, g) source of funds for the investigations, h) conclusions, and i) recommendations. This was reported as a median time – a time it takes for half of the cases to occur after the initial anthrax outbreak.

### Study selection

#### Searching for studies.

We conducted a comprehensive search in MEDLINE, PubMed, Scopus, Embase, Google Scholar, and Web of Science databases for articles on outbreak investigations on suspected and confirmed animal and human anthrax cases in Africa. To search for articles, we used the PICO framework [43] previously developed. The PICO questions for the search strategy is presented in S2 Table. We opted for an academic literature and systematic review approach as opposed to grey literature because we felt that this would provide a more scientific unbiased basis to our literature search and synthesis. Our review covered published peer reviewed studies, which provide high quality methodological findings. Grey literature and unpublished studies were not included due to concerns about methodological quality and lack of peer scrutiny.

#### Inclusion and exclusion criteria.

We reviewed article titles and abstracts using the PICO criteria and identified and excluded the following: i) reports with no data, i.e., literature reviews, letters, and other reports on anthrax outbreaks that do not contain data; ii) reports and articles with data collected before 2014 and after 2023; iii) reports with data unrelated to animal and human cases of anthrax outbreaks, and, iv) articles on anthrax outbreaks outside Africa. Articles were classified as describing anthrax outbreak investigations if they described an event or events meeting the following criteria: i) event classified as an “outbreak,” or “epidemic,” or “elevated” number of anthrax cases; ii) event included a single suspected case of animal or human anthrax; and iii) investigations or response to anthrax outbreaks. The final articles were assessed for inclusion on the basis of the following key words – animal or human, anthrax outbreak, epidemiology, investigations, response, Africa, and dates between 2014 and 2023.

### Screening and extraction of studies

The search results were exported to Zotero software [44], a research tool for collecting, organizing, and managing research publications. Following the removal of duplicates, studies were transferred to Covidence (an artificial intelligence software for systematic reviews and meta-analysis) [45]. The screening was performed by five people (ARA, AWW, RA, IBK & RO) to comply with the standard protocol for conducting systematic reviews aimed at minimizing bias throughout the entire process. The screening was performed at three different layers: duplicate removal, title, and abstract and full-text review.

The review team independently assessed all the studies from all the searches. Studies were marked as “include”, “exclude”, or identified as “maybe” following the eligibility criteria. Weekly virtual meetings were organized virtually using Zoom to address any conflicts between the five reviewers. Technical guidance was obtained from EB, ER and RW during the entire review process, which shaped the review process.

A custom extraction template was designed by ARA and adopted for use in Covidence. The use of custom extraction templates was approved by the entire review team. ARA, RA and IBK extracted data from individual studies, whereas AWW and RO rechecked the extraction process to ensure accuracy and rigor.

### Risk of bias assessment

We performed a risk of bias assessment in Microsoft Excel according to the Newcastle-Ottawa Scale (NOS) (S2 Table) [46]. The risk of bias assessment was important as it could reveal how reliable these studies were in informing our research question.

### Data management and analysis

The extracted data were exported to a spreadsheet (Microsoft Excel 365; Microsoft Corporation, Redmond, Washington) and sent to AWW for curation (S3 Table). During data curation, we deleted excess empty rows that were in the dataset, aligned the column margin to the data, well wrapped in the cells. Three data analysts (ARA, RA and AWW) inspected the data in comparison with the analysis plan that we had developed earlier during protocol development and registration. Despite the few studies that were ultimately included in this review, we decided to conduct a meta-analysis given that we had more than a threshold of five studies needed. We chose two data analysis platforms; Microsoft Excel to analyse descriptive data and RStudio version 4.3.1 [47] to analyse quantitative data.

We carried out descriptive analysis on the number of studies assessed, the affiliations of authors to understand geographical distribution, and the frequency of different study designs employed in outbreak investigations, use of case definitions, team composition, type of outbreak (animal, human or mixed outbreak), frequency of performing descriptive epidemiology, and the comprehensiveness of the descriptive epidemiology (person, place and time characteristics), and, frequency of performing analytical epidemiology. Furthermore, we described gaps reported in the literature, such as diagnostic limitations or resource constraints, as well as the public health actions carried out and recommendations by authors in response to these outbreaks.

For the meta-analysis, we extracted a new dataset with few variables (citation, sample size, date of alert, date of end of outbreak, status of public health intervention, team composition (One Health), country, and host. Time to control variable was generated by subtracting the date of end of outbreak from date of alert. This was the dependent variable in the analysis as informed by our research question. This new dataset was then exported to Rstudio. The statistical output (pooled median duration of anthrax control) was generated using the function of “metafor”, “tidyverse” [48], “ggplot2” packages [49] and the results presented in graphics and tables. First, we computed the median, lower and upper quartiles and inter-quartile range (IQR) for all studies.

As we anticipated considerable between-study heterogeneity arising from different hosts (animal and humans), different study designs, different countries across Africa and different dynamics in outbreak response per country, a random-effect model was used to pool effect sizes per study. We chose the random-effects model because it assumes that the true effect sizes vary within meta-analyses, and it was our interest to detect the variation of duration to control of anthrax outbreaks over time which could help us deduce the true pooled median of duration to control distribution underlying our data. The “rma” function was used to fit the meta-analytic model using the Restricted Maximum Likelihood “REML” method [50]. We chose the “rma” function given its robustness in handling multiple meta-analysis models including random effects. Then we generated a forest plot using ggplot2. We chose ggplot2 given the excellent visual outputs in Rstudio.

The REML estimator was used to calculate the heterogeneity variance τ*2*τ*2*. We used *Knapp-Hartung* adjustments [51] to calculate the confidence interval around the pooled effect. Furthermore, we assessed for heterogeneity between the studies with Higgin’s inconsistency statistics (I^2^) and the Cochran’s Q statistic. A value approximating to zero suggests homogeneity, and values of 25–50%, 50–75% and 75–100% represent low, medium and large heterogeneity, respectively. Publication bias was explored with a visual inspection of the symmetry of the funnel plot but also depended on the magnitude of the index, and classified accordingly. For example, a value of -/+ 1, -/+2 within the range and -/+2 outside the range was considered symmetrical with no bias, minor asymmetry with slight bias and major asymmetry with high bias respectively. We also performed sensitivity analysis to assess changes in results with exclusion and inclusion of other studies and we made a decision to include all the 10 studies in the meta-analysis.

We wanted to explore the factors associated with the duration to control. Given the fewer variables we were keen not to eliminate all of them in the model, first we used a step by step back-drop approach in building a logistic regression model. The logistic regression model was Generalized Linear Model (GLM) using “binomial” family. We fitted a complete model with different variables informed by epidemiological knowledge on duration to control of anthrax outbreaks. We fitted the model with; public health intervention, team composition, funding mechanism for the outbreak control, the use of case-definitions, and the host. For the host, we were keen to explore if outbreaks in humans could be more easily controlled than those in animals. The associations were assessed by odds ratios, with a 5% level of statistical significance.

## Results

**Fig 1:**
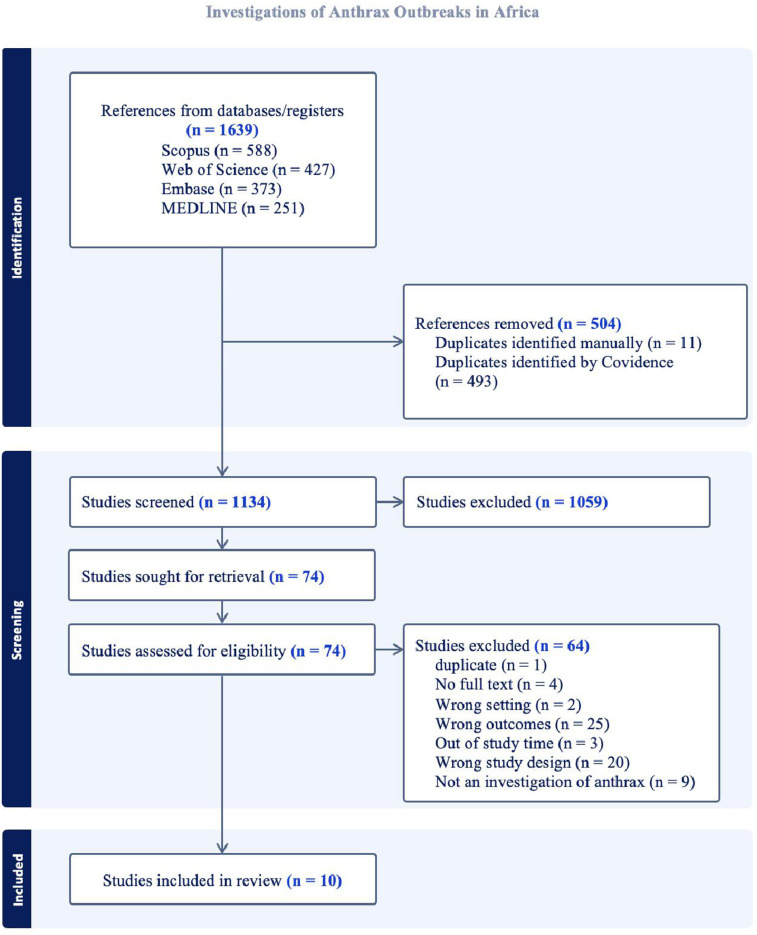
PRISMA flow chart for anthrax outbreaks investigations review.

A total of 1,639 studies were identified following the literature search. Of these, 10 met our eligibility criteria and were considered for review (Fig 1). The studies that were included in the study are summarized in (S4 Table) [30,32,35,36,13,^52^–^54^]. Details of the studies that were excluded in the title and abstract stage of the review are summarized in (S5 Table), and those that were excluded after assessment for eligibility and reasons for exclusion are summarized in (S6 Table). Examples of studies which were excluded include “Potential distribution of *Bacillus anthracis* suitability across Uganda using INLA”, which did not include epidemiological investigations information [55]; and “Investigation and source-tracing of an anthrax outbreak in Gansu Province, China” which occurred outside Africa [56].

### Time to control

**Fig 2:**
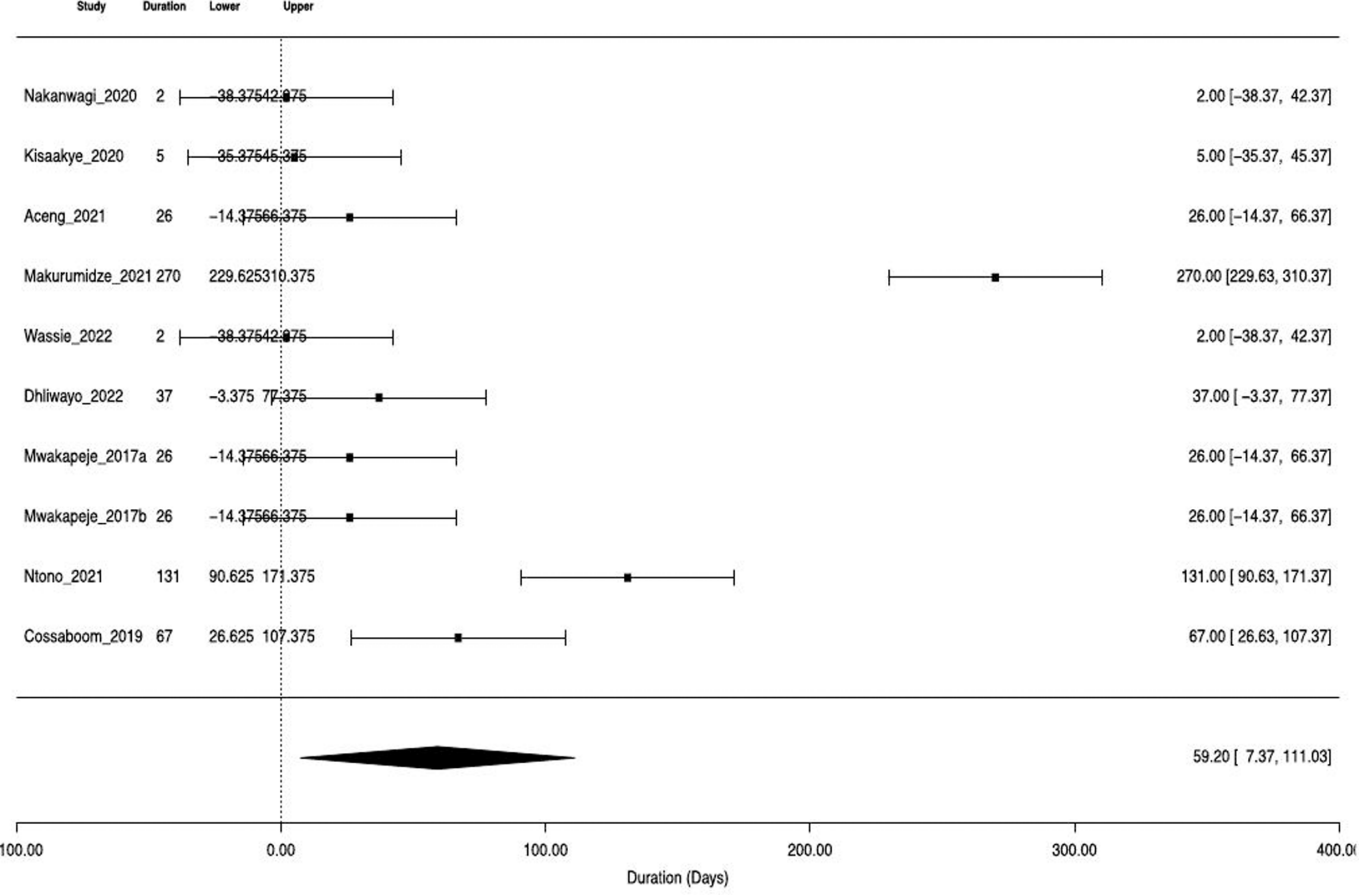
Forest plot for pooled median duration to control anthrax epidemics in Africa.

**Fig 3:**
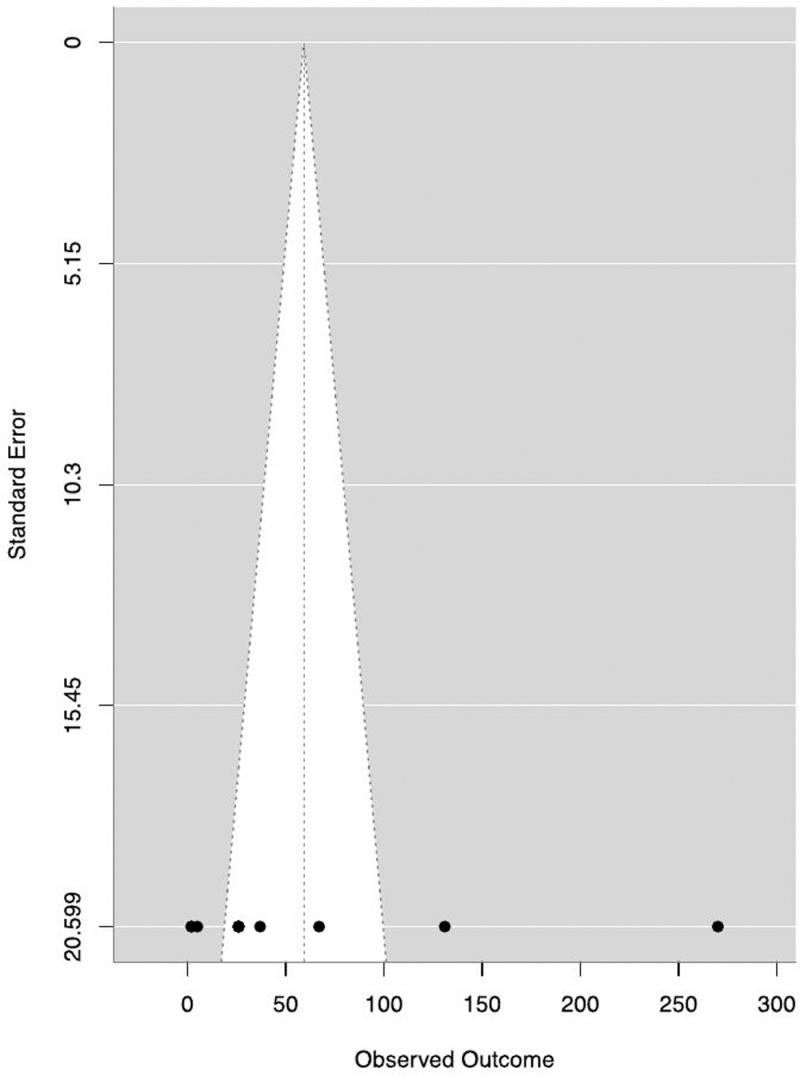
Funnel plot showing heterogeneity in the duration of outbreaks across studies.

The pooled median duration to control was 40.5 (IQR 80.8) days and the estimated duration of outbreak end was 59.2 days (95%CI: 7.4-111.0, p = 0.03 (SE: 26.4, Z value: 2.24) (Fig 2). High heterogeneity was observed across different studies (93.9%, tau=81.4, with total sampling variability = 16.5), as reflected by the funnel plot (Fig 3). All the factors included in the logistic regression were not statistically significant.

### Verification period

A total of 8 of the 10 studies reported delays in verification; 5/8 reported delayed laboratory confirmation of B. *anthracis*. The median time to alert was 5 days, min, max (0, 490). Only 4 of the 8 studies reported dates of confirmation, and the median was 96.5 days, min, max (6, 490).

### Authorship

**Fig 4:**
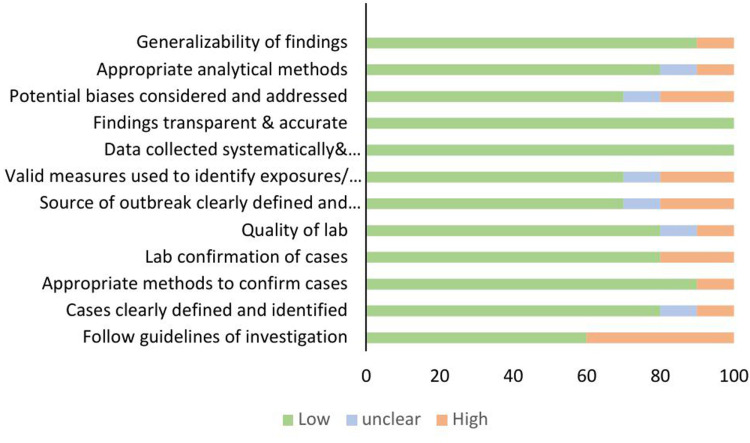
Risk of bias assessment across studies included in the anthrax outbreaks review.

In terms of authorship, 9/10, had the first authors from Africa with Eastern Africa contributing the majority, 7/9 followed by the Southern African region (2/9). Uganda contributed the majority (5/10) followed by Zimbabwe (2/10) (Fig 4).

### Host

For hosts of infections, the majority of investigations reported anthrax outbreaks in humans alone (7/10) followed by those where the outbreak occurred in both humans and animals. The source of the outbreak included handling the carcass of an animal that died suddenly (5/10) and consuming meat from an animal that died suddenly (4/10).

### Outbreak declaration

The mean number of days to national level declaration of outbreaks, among the 6 reports that included this data, was 59.2 (95%CI: 7.4-111.0, p=0.03 (SE: 26.4, Z value: 2.24). Four papers did not report declaration of outbreaks.

### Case definitions

All the studies reported that investigations used case definitions although there was no information on how many of these definitions were translated into local languages. On further interrogation of the case definitions, 4/10 used suspected and confirmed case definitions, 1 used only the confirmed case definition, 1 used the probable and confirmed case definitions, 1 used only the probable case definition while 3 used only the suspected case definition.

### Study design

All studies performed descriptive epidemiology to characterize the outbreak, 9/10 performed analytical epidemiology, and 1/10 reported no need for analytical epidemiology. Furthermore, 7/10 described person/host, place and time descriptions followed by 3/10 which reported only person/host aspects and left out the time component. In terms of study design, 4/9 used a case control study design, 2/9 used a cross-sectional and 3/9 used a cohort study design.

### Team composition

All studies reported including an epidemiologist, a local leader, and laboratory personnel in their response. Additionally, 9/10 studies featured FETP personnel, 6/10 included a veterinarian, 1/10 had a social worker, and 2/10 included an environmental health officer.

### Public health actions

Public health interventions play a critical role in determining the duration to control—we explored which actions were taken. Most studies (8/10) reported instituting public health actions during outbreak investigations. All the 8 studies that conducted public health actions, reported health education of communities on the anthrax mode of transmission and safe carcass disposal practices, whereas 4/8 reported giving postexposure prophylaxis to exposed persons. Fewer studies reported livestock vaccination (3/8) and setting up a One Health team to enhance collaboration between the human and animal sectors during anthrax outbreaks (2/8).

### Risk of bias assessment

There was low bias detected in the studies with 60%, 80%, 80%, 90%, and 100% bias in following guidelines, clearly defining cases, laboratory confirmation of cases, having appropriate methods to confirm cases, findings being transparent and accurate and data collection being systematically performed (Fig 4).

## Discussion

This systematic review and meta-analysis is the first to synthesize strengths and gaps in epidemiological investigations and compute duration to control of anthrax outbreaks in Africa. The pooled median duration to control of anthrax outbreaks was 40.5 days and the estimated duration to outbreak end was 59.2 days. This implies that it took this period of time for outbreaks to register the last case and for the outbreaks to have been declared controlled. The 7-1-7 framework proposes a target for timeliness to detect, notify and mount an effective response to public health emergencies to improve population health outcomes. The 7-1-7 metric sets 7 days to identify every suspected outbreak, 1 day to notify it, and 7 days to initiate response to public health emergencies [57]. Response initiation includes epidemiological investigations as a critical component required for control of an outbreak. The WHO 13^th^ General Program of Work framework [58] describes the “earliest date of any public health intervention to control the event”, which can include the time the field investigation started, the time the incident management system was established, the time the rapid response team was established, or the time that risk communications were started [59].

The median duration to control in this meta-analysis extends beyond the period an outbreak of infectious disease should be controlled, which ideally would be 21 days for anthrax [60,61]. Several factors that could have contributed to this long duration to control period in the studies examined include: limited awareness of anthrax symptoms and transmission routes or cultural practices that may lead to failure by communities to recognize the disease early or understand the risks associated with handling or consuming infected animals [62,63]; death of an animal in a community that relies on livestock for their livelihoods may create economic pressure to salvage meat from diseased animals, increasing the risk of human infection [64,65]; failure to map hotspots lowers the index of suspicion in areas that may not have experienced anthrax outbreaks before [66,67]; and historical or current mistrust in government and health authorities may make communities reluctant to report outbreaks or cooperate with response efforts [68]. Since our review targeted only epidemiological investigations, our discussion focuses on the epidemiological gaps identified. The longest incubation period of anthrax is 7 days, if we exclude inhalational anthrax that could take 60 days. That implies that the two incubation periods that define the dynamics of communicable disease control for declaration of an outbreak would be 14 days. The incubation period defines the exposure period when the vegetative form of anthrax is present and hence infection can spread. During this period, the infected animal host sheds the vegetative bacilli onto the ground and these sporulate on exposure to the air within hours. The spores, which can persist in soil for decades, wait to be taken up by another animal host, when germination and multiplication can again take place upon infection [1]. Control of an outbreak is about ensuring that the vegetative form of anthrax does not reach the next human victim to be infected.

The timeliness of anthrax outbreaks start to outbreaks end would be 54.5 days taking into account the two longest incubation periods of anthrax [69]. This does not differ much from our estimated duration to outbreak end of 59.2 days. This adds to the body of knowledge in this field. For example Impouma et al described timeliness of different disease outbreaks response in WHO member states across Africa and reported an improvement in response from 146 days in 2017–53 days in 2019 [70]. However, this finding is far from the ideal if we are to control anthrax outbreaks in time. This implies that response is still delayed with resultant spread and diverse effects of the outbreaks which include higher incidence of disease, and increased risk of complications and fatalities. The period from alert to control serves as a learning opportunity for future outbreaks. These shortcomings in epidemiological response highlights need for improved preparedness, and refinement of response approaches to quickly control outbreaks and curb the potential for widespread transmission.

Much as the median time to alert was 5 days, majority of the studies reported delays in verification of anthrax outbreaks with 63% reporting no or delayed laboratory confirmation of B. *anthracis* and less than half mentioning actual dates of confirmation. The WHO IDSR guidelines set cumulative detection of disease timelines at 7 days and African countries adapted these guidelines and targets [17,71]. The 7:1:7 metrics sets 7 days within which detection should have been carried out [57]. The verification period for an anthrax outbreak refers to the timeframe during which the presence, extent, and nature of the outbreak is confirmed. This is done by confirmation of a true case by laboratory diagnosis using appropriate methods or cross-checking of the reported case against a standard case definition. In order for verification to be done, a case notification has to be effected which generates an alert. This defines the period from disease occurrence to initiation of epidemiological investigation and response. Delayed laboratory confirmation of outbreaks would henceforth affect initiation of response including epidemiological investigations.

The median duration to outbreak end in this review extends beyond the two incubation periods that would be ideal for outbreak control declaration [72]. Because the mean incubation period of anthrax is 7 days [73,74], dynamics of infectious disease implies that a duration of 2 weeks from onset of the outbreak would be ideal for declaring the end of an anthrax outbreak.

The majority of the investigations reported anthrax outbreaks in humans only with 30% reporting the outbreak in both humans and animals. Exploration of the source of the outbreak revealed contact with or consumption of meat from an animal that died suddenly. Anthrax transmission is an established fact in science, i.e., contact with anthrax spores via skin cuts or abrasions, handling contaminated animal products, direct contact with infected animals or ingestion of undercooked or contaminated meat from infected animals [2]. Epidemiological investigation of human anthrax in isolation from animal anthrax outbreak leaves a gap in evidence generation and recommendations for effective control.

All the studies reported that investigations used case definitions. However, a quarter of the studies omitted the probable case definition. A few went further to use single levels of case definitions. A case definition is an important epidemiological tool for standardizing criteria for the identification of cases is a critical step in outbreak investigations. All case definitions must include the three classical dimensions of epidemiological variables: *time*, *place* and *person.* A case definition should include clinical and demographic variables but must not include exposure or risk factor of interest. It’s classified in 3 levels – suspected, probable and confirmed [75–77]. Case definitions that fall short of the standard epidemiological criteria for setting, present a gap in identification of cases and determination of risk factors for transmission which culminates in incorrect or ineffective recommendations for public health interventions. Additionally, none of the studies mentioned translation of the questionnaire into local languages. Epidemiologists often assume that one of the field investigators speaks the local language where the outbreak has occurred. However, this is an erroneous assumption and the use of interpreters may lead to inaccurate information being elicited from the respondents.

All studies performed descriptive epidemiology to characterize the outbreak, and 90% performed analytical epidemiology. However, 30% of the studies did not report on the time component of descriptive epidemiology. Descriptive epidemiology involves examining the distribution of a disease in a population and observing the basic features of its distribution; whereas analytical epidemiology involves investigating a hypothesis about the cause of a disease by studying how exposures relate to the disease [78,79]. Descriptive epidemiology is antecedent to analytical epidemiology for an investigator to know where to look, what to look for and develop a viable hypothesis to test. The essential characteristics of diseases that are characterized in descriptive epidemiology are time, place and person. Missing any of these components poses a critical gap in investigations. The time component missed by some of the studies implies that the time since the anthrax outbreak occurred, the temporal trends and all benefits of the epicurve in understanding the outbreak were missed.

Among the studies that conducted analytical epidemiology, the majority used case-control and cohort study designs. The two main study designs recommended for outbreak investigations are retrospective cohort studies and case-control studies [78,79]. The choice of a cross-sectional design by two of the studies examined represents a poor choice of study design in outbreak investigations. Case-control and retrospective cohort study designs offer several advantages in identifying the source and factors associated with the outbreak. They are relatively quick and cost-effective and enable speedy identification of risk factors crucial in understanding the source of the outbreak which enables prompt public health actions for control and prevention of further spread.

All studies reported including an epidemiologist, a local leader, and laboratory personnel in their response. The majority categorized the epidemiologists as FETP personnel. Approximately two thirds of the studies included a veterinarian, while very few included a social worker, and an environmental health officer. The WHO and Africa CDC recognize that zoonotic diseases, food safety and antimicrobial resistance are key areas in greatest need of a collaborative One Health approach across African nations. The One Health framework on priority zoonotic diseases was developed to incorporate One Health approach in improving public health [80]. This applies to the constitution of teams that respond to zoonotic disease outbreaks including anthrax which should have human, animal and environmental health personnel. In addition, Stellmach et al argue for an interdisciplinary conception of emergencies and the recognition that social, psychological and institutional factors influence all aspects of care. Social scientists, anthropologists in particular, have been recognized as important players in disease outbreak response because of their ability to assess social, economic and political factors in local contexts [81]. FETPs train a special group of epidemiologists that apply epidemiologic methods to unexpected health problems that require a timely response in a community setting. This includes investigation and analysis of disease outbreaks, health events, and other public health issues directly in the field, often under challenging conditions. FETPs have been proven to be a game changer in response to public health emergencies and achievement of national and global health security [37,82,83].

The majority of the studies reviewed were supported through donor funding, and only 20% were funded by national governments. Importantly, the studies funded by donors had FETPs. FETPs were rolled out in Africa with support from the US CDC which covers many countries [84]. The overreliance on donor funding raises great concerns about the sustainability of response strategies in Africa. If donor priorities shift or funding is reduced, then all gains attained in response to public health emergencies within 24–48hrs because of FETPs will go down the drain. Moreso, the number of trained epidemiologists is still far from the recommended WHO target of 1:200,000 population [85]. Henceforth, there is need for increased investment by governments to allocate funds for training critical cadres including epidemiologists, if Africa is to achieve compliance with the International Health Regulations and attain global health security goals. However, traditional civil service staff at local levels who participate in outbreak investigations are employed by government. Governments should henceforth extend their support to include critical cadres of staff such as field epidemiologists, increasing public spending on health worker recruitment could reduce workforce shortages in sub-Saharan Africa [86].

The majority of studies reported having instituted public health actions during outbreak investigations; however, 20% did not do so. Public health action such as vaccination campaigns, quarantine, or public health education, is important for early control and prevention measures to reduce the spread of disease [87]. Failure to do this may result in the spread of infection and delayed control. Policy recommendations to prevent future outbreaks should be preceded by public health actions as good response practices.

## Limitations

Our review was limited by the small number of studies, which may have resulted in reduced statistical power with wider confidence intervals for the meta-analysis which affected our ability to detect significant effects In addition, the small number made subgroup analysis difficult. Because our review covered only published peer reviewed studies, grey literature and unpublished studies which were not included due to concerns about methodological quality and lack of peer scrutiny, would increase the number of articles and improve generalizability of results. There were also heterogeneity challenges where there were 3 study designs within the small number of studies. This could have affected the pooled results limiting generalizability. Limiting our studies only to articles published in English could have also excluded some studies published in countries such as North and West Africa, whose official language is not English. Despite these limitations, we are confident of the results adduced because we broadened the search strategy to include multiple databases.

## Conclusions

In conclusion, the median duration to control in this meta-analysis was much longer than the IDSR and 7:1:7 metric recommended periods. Several gaps in epidemiological investigations have been identified including: delayed verification of outbreaks with most investigations focusing on human anthrax outbreaks, often neglecting the zoonotic aspect, which is crucial for effective control; and inappropriate working case definitions. This study underscores the need for timely verification, comprehensive epidemiological investigations, and a One Health approach. Addressing these gaps is crucial for achieving faster and more effective control of anthrax outbreaks, thereby improving public health outcomes.

## Supporting information

S1 Checklist PRISMA 2020 Checklist. (PDF)

S1 Table PICO questions for the search strategy. (DOCX)

S2 Table Risk of Bias Assessment (NOS). (XLSX)

S3 Table Dataset for Metaanalysis. (XLSX)

S4 Table Database of studies included in analysis. (CSV)

S5 Table Irrelevant articles at title and abstract screening. (DOCX)

S6 Table Articles excluded at full-text screening with reasons for exclusion. (DOCX)
