# Supplementary material for: Time to control of anthrax outbreaks in Africa, 2014–2023: A systematic review and meta-analysis
Source: PLOS Glob Public Health. 2025 Apr 22;5(4):e0004534. doi: 10.1371/journal.pgph.0004534 (PMC12013908; doi:10.1371/journal.pgph.0004534)
Supplement: S1 Table — (DOCX) [file pgph.0004534.s002.docx]

**S1 Table: PICO questions for the search strategy**

| **Population** | Humans, Cattle, Goats, Sheep, Wildlife  All ages, All gender |
| --- | --- |
| **Intervention** | Anthrax outbreak investigation, reporting of anthrax, date of alert notification, start date of investigation, outbreak end date |
| **Comparison** | 7-1-7 timeliness metrics |
| **Outcome** | Time to control, anthrax.mp., Anthrax/, bacillus anthracis.mp., Anthrax disease, Bacillus anthracis/ |
|  | **Countries:** Angola/ or Benin/ or Botswana/ or Burkina Faso/ or Burundi/ or Cabo Verde/ or Cape Verde/ or Cameroon/ or Central African Republic/ or Chad/ or Comoros/ or Congo/ or Republic of Congo/ or Democratic Republic of Congo/ or Cote d'Ivoire/ or Egypt/ or Ivory Coast/ or Djibouti/ or Equatorial Guinea/ or Eritrea/ or Eswatini/ or Ethiopia/ or Gabon/ or Gambia/ or Ghana/ or Guinea/ or Guinea Bissau/ or Kenya/ or Lesotho/ or Liberia/ or Libya/ or Madagascar/ or Malawi/ or Mali/ or Mauritania/ or Mauritius/ or Mayotte/ or Morocco/ or Mozambique/ or Namibia/ or Niger/ or Nigeria/ or Rwanda/ or Reunion/ or Saint Helena/ or "Sao Tome and Principe"/ or Senegal/ or Seychelles/ or Sierra Leone/ or Somalia/ or South Africa/ or South Sudan/ or Sudan/ or Swaziland/ or Tanzania/ or Togo/ or Tunisia/ or Uganda/ or Western Sahara/ or Zambia/ or Zimbabwe/  (Angola or Benin or Botswana or "Burkina Faso" or Burundi or "Cabo Verde" or "Cape Verde" or Cameroon or "Central African Republic" or Chad or Comoros or Congo or "Republic of Congo" or "Democratic Republic of Congo" or "Cote d'Ivoire" or Egypt or "Ivory Coast" or Djibouti or "Equatorial Guinea" or Eritrea or Eswatini or Ethiopia or Gabon or Gambia or Ghana or Guinea or "Guinea Bissau" or Kenya or Lesotho or Liberia or Libya* or Madagascar or Malawi or Mali or Mauritania or Mauritius or Mayotte or Morocco or Mozambique or Namibia or Niger or Nigeria or Rwanda or Reunion or "Saint Helena" or "Sao Tome and Principe" or Senegal or Seychelles or "Sierra Leone" or Somalia or "South Africa" or "South Sudan" or Sudan or Swaziland or Tanzania or Togo or Tunisia or Uganda or Western Sahara or Zambia or Zimbabwe).mp. |
|  | **Timeline:** limit 10 to yr="2014 - 2023" |
