# Supplementary material for: Time to control of anthrax outbreaks in Africa, 2014–2023: A systematic review and meta-analysis
Source: PLOS Glob Public Health. 2025 Apr 22;5(4):e0004534. doi: 10.1371/journal.pgph.0004534 (PMC12013908; doi:10.1371/journal.pgph.0004534)
Supplement: S5 Table — (DOCX) [file pgph.0004534.s006.docx]

**S5 Table. Irrelevant articles at title and abstract screening (n=1059)**

| **#** | **Title** | **Authors** | **Published Year** | **Journal** | **DOI** |
| --- | --- | --- | --- | --- | --- |
| 1 | Neuroinfectiology of an atypical anthrax-causing pathogen in wild chimpanzees | Grassle T.; Jager C.; Kirilina E.; Jaffe J.E.; Carlier P.; Pizarro A.; Jauch A.; Reimann K.; Lipp I.; Wittig R.M.; Crockford C.; Weiskopf N.; Leendertz F.H.; Morawski M. | 2023 | bioRxiv | https://dx.doi.org/10.1101/2023.11.21.568053 |
| 2 | KNOWLEDGE OF AND PRACTICES AROUND ZOONOTIC DISEASES AMONGST ACTORS IN THE LIVESTOCK TRADE IN THE LAKE VICTORIA CRESCENT ECOSYSTEM IN EAST AFRICA | Majiwa H.; Bukachi S.A.; Omia D.; Fevre E.M. | 2022 | medRxiv | https://dx.doi.org/10.1101/2022.03.10.22272135 |
| 3 | The World Health Organization's Disease Outbreak News: a retrospective database | Carlson C.J.; Boyce M.R.; Dunne M.; Graeden E.; Lin J.; Abdellatif Y.O.; Palys M.A.; Pavez M.; Phelan A.L.; Katz R. | 2022 | medRxiv | https://dx.doi.org/10.1101/2022.03.22.22272790 |
| 4 | Genomic surveillance of Bacillus cereus sensu lato strains isolated from meat and poultry products in South Africa enables inter- and intra-national surveillance and source tracking | Carroll L.M.; Pierneef R.; Mathole A.; Atanda A.; Matle I. | 2022 | bioRxiv | https://dx.doi.org/10.1101/2022.01.18.476746 |
| 5 | Strengthening surveillance, disease detection, and outbreak response through Guinea-Bissau's Frontline Field Epidemiology Training Program: a cross-sectional descriptive study | Camara M.; da Costa F.P.; Chambe G.; Betunde A.; Cardoso P.; Johnson K.; Rullan-Oliver P.; Lopez A. | 2023 | The Pan African medical journal | https://dx.doi.org/10.11604/pamj.2023.45.133.30807 |
| 6 | Efficacy of different AV7909 dose regimens in a nonclinical model of pulmonary anthrax | Henning L.; Anderson M.; Triplett C.; Smith T.; Boyce K.; Hendey L.; Ridenour A.; Eng J.; Schaeufele D.; Wilson E.; Sabourin C.L.; Adams L.E.; Babas T.; Parish L.; Wolfe D. | 2023 | Human Vaccines and Immunotherapeutics | https://dx.doi.org/10.1080/21645515.2023.2290345 |
| 7 | Construction of a biological risk scale for the military during external operations | Cherouvrier A.; Meynard J.-B.; Bedubourg G.; Tanti H.M. | 2023 | Environnement, Risques et Sante | https://dx.doi.org/10.1684/ers.2023.1752 |
| 8 | Use of wild edible and nutraceutical plants in Raya-Azebo District of Tigray Region, northern Ethiopia | Giday M.; Teklehaymanot T. | 2023 | Tropical Medicine and Health | https://dx.doi.org/10.1186/s41182-023-00550-8 |
| 9 | One hundred years of zoonoses research in the horn of africa: A scoping review | Cavalerie L.; Wardeh M.; Lebrasseur O.; Nanyingi M.; McIntyre K.M.; Kaba M.; Asrat D.; Christley R.; Pinchbeck G.; Baylis M.; Mor S.M. | 2021 | PLoS Neglected Tropical Diseases | https://dx.doi.org/10.1371/journal.pntd.0009607 |
| 10 | Common Garlic (Allium sativum L.) has Potent Anti-Bacillus anthracis Activity | Kaur R.; Tiwari A.; Manish M.; Maurya I.K.; Bhatnagar R.; Singh S. | 2020 | Journal of ethnopharmacology | https://dx.doi.org/10.1016/j.jep.2020.113230 |
| 11 | Adoption of Biosecurity Practices in Smallholder Dairy Farms in Ethiopia | Nyokabi N.S.; Berg S.; Mihret A.; Almaw G.; Worku G.G.; Lindahl J.F.; Wood J.L.N.; Moore H.L. | 2023 | Transboundary and Emerging Diseases | https://dx.doi.org/10.1155/2023/2277409 |
| 12 | Risk factors associated with the spatial distribution of priority zoonoses in animal health in Cameroon from 2012 to 2021 | Amawota A.A.F.; Feussom J.-M.K.; Mouiche M.M.M.; Mamoudou A.; Oyetola W.D.; Akakpo J.A.; Alambedji R.B. | 2023 | Virologie | https://dx.doi.org/10.1684/vir.2023.1011 |
| 13 | Implementation and evaluation of a participatory process to support the co-development of an integrated surveillance system for anthrax in Burkina Faso | Nana S.D.; Duboz R.; Hendrikx P.; Bordier M. | 2023 | Virologie | https://dx.doi.org/10.1684/vir.2023.1011 |
| 14 | Assessing and Prioritizing Zoonotic Diseases in Punjab, India: A One Health Approach | Sakshi; Dhaka P.; Bedi J.S.; Aulakh R.S.; Singh R.; Gill J.P.S. | 2023 | EcoHealth | https://dx.doi.org/10.1007/s10393-023-01654-7 |
| 15 | Veterinary services under siege: how the armed conflict in Sudan threatens animal and human health and how to respond | Mohammed A.A.A.; Ahmed M. | 2023 | Infection Ecology and Epidemiology | https://dx.doi.org/10.1080/20008686.2023.2281054 |
| 16 | Unraveling crime scenes strand by strand: The forensic odyssey of Bruce Budowle | Budowle B. | 2023 | BioTechniques | https://dx.doi.org/10.2144/btn-2023-0069 |
| 17 | Zoonotic diseases transmitted from the camels | Khalafalla A.I. | 2023 | Frontiers in Veterinary Science | https://dx.doi.org/10.3389/fvets.2023.1244833 |
| 18 | Examination of critical factors influencing ruminant disease dynamics in the Black Sea Basin | Arede M.; Beltran-Alcrudo D.; Aliyev J.; Chaligava T.; Keskin I.; Markosyan T.; Morozov D.; Oste S.; Pavlenko A.; Ponea M.; Starciuc N.; Zdravkova A.; Raizman E.; Casal J.; Allepuz A. | 2023 | Frontiers in Veterinary Science | https://dx.doi.org/10.3389/fvets.2023.1174560 |
| 19 | Trends in public health emergencies in the WHO African Region: An analysis of the past two decades public health events from 2001 to 2022 | Koua E.L.; Njingang J.R.N.; Kimenyi J.P.; Williams G.S.; Okeibunor J.; Oka S.; Gueye A.S. | 2023 | BMJ Global Health | https://dx.doi.org/10.1136/bmjgh-2023-012015 |
| 20 | Prioritization of zoonoses for multisectoral, One Health collaboration in Somalia, 2023 | Osman A.Y.; Mohamed H.; Mumin F.I.; Mahrous H.; Saidouni A.; Elmi S.A.; Adawe A.K.; Mo'allim A.A.; Lubogo M.; Malik S.M.M.R.; Mwatondo A.; Raji T.; Ahmed A.D.; Zumla A.; Dar O.; Kock R.; Mor S.M. | 2023 | One Health | https://dx.doi.org/10.1016/j.onehlt.2023.100634 |
| 21 | Elephant tourism: An analysis and recommendations for public health, safety, and animal welfare | Warwick C.; Pilny A.; Steedman C.; Grant R. | 2023 | International Journal of One Health | https://dx.doi.org/10.14202/IJOH.2023.49-66 |
| 22 | Use of technology to provide continual education to sub-national level event-based surveillance coordinators in six high-risk regions in Tanzania mainland in 2021/2022 | Musyani A.; Ngailo L.; Mutayoba R.; Ngalesoni F. | 2023 | BMC Proceedings | https://dx.doi.org/10.1186/s12919-023-00273-y |
| 23 | Improvement in the Surveillance System for Livestock Diseases and Antimicrobial Use Following Operational Research Studies in Sierra Leone January-March 2023 | Konteh S.A.; Bangura F.I.; Leno A.; Satyanarayana S.; Nair D.; Bah M.A.; Saidu S.; Sellu-Sallu D.; Gborie S.R.; Kamara S.M.; Jalloh A.T.; Kanu J.S.; Kamara K.N.; Moiwo M.M.; Dsani E.; Nantima N. | 2023 | Tropical Medicine and Infectious Disease | https://dx.doi.org/10.3390/tropicalmed8080408 |
| 24 | Genomic and Phylogenetic Analysis of Bacillus cereus Biovar anthracis Isolated from Archival Bone Samples Reveals Earlier Natural History of the Pathogen | Norris M.H.; Zincke D.; Daegling D.J.; Krigbaum J.; McGraw W.S.; Kirpich A.; Hadfield T.L.; Blackburn J.K. | 2023 | Pathogens | https://dx.doi.org/10.3390/pathogens12081065 |
| 25 | The persistence of time: the lifespan of Bacillus anthracis spores in environmental reservoirs | Barandongo Z.R.; Dolfi A.C.; Bruce S.A.; Rysava K.; Huang Y.-H.; Joel H.; Hassim A.; Kamath P.L.; van Heerden H.; Turner W.C. | 2023 | Research in Microbiology | https://dx.doi.org/10.1016/j.resmic.2023.104029 |
| 26 | Whole genome sequencing of Bacillus anthracis isolated from animal in the 1960s, Brazil, belonging to the South America subclade | de Andrade T.S.; Camargo C.H.; Campos K.R.; Reis A.D.; Santos M.B.D.N.; Zanelatto V.N.; Takagi E.H.; Sacchi C.T. | 2023 | Comparative Immunology, Microbiology and Infectious Diseases | https://dx.doi.org/10.1016/j.cimid.2023.102027 |
| 27 | Modeling gastrointestinal anthrax disease | Oh S.Y.; Chateau A.; Tomatsidou A.; Elli D.; Gula H.; Schneewind O.; Missiakas D. | 2023 | Research in Microbiology | https://dx.doi.org/10.1016/j.resmic.2023.104026 |
| 28 | The global burden of neglected zoonotic diseases: Current state of evidence | Di Bari C.; Venkateswaran N.; Fastl C.; Gabriel S.; Grace D.; Havelaar A.H.; Huntington B.; Patterson G.T.; Rushton J.; Speybroeck N.; Torgerson P.; Pigott D.M.; Devleesschauwer B. | 2023 | One Health | https://dx.doi.org/10.1016/j.onehlt.2023.100595 |
| 29 | Identifying macroplastic pathobiomes and antibiotic resistance in a subtropical fish farm | Naudet J.; d'Orbcastel E.R.; Bouvier T.; Godreuil S.; Dyall S.; Bouvy S.; Rieuvilleneuve F.; Restrepo-Ortiz C.X.; Bettarel Y.; Auguet J.-C. | 2023 | Marine Pollution Bulletin | https://dx.doi.org/10.1016/j.marpolbul.2023.115267 |
| 30 | RNA-seq research landscape in Africa: systematic review reveals disparities and opportunities | Doughan A.; Adingo W.; Salifu S.P. | 2023 | European Journal of Medical Research | https://dx.doi.org/10.1186/s40001-023-01206-3 |
| 31 | The Utilization and Development of Viral Vectors in Vaccines as a Prophylactic Treatment Against Ebola Virus as an Emerging and Zoonotic Infectious Disease | Garcia A.; Grundmann O. | 2023 | Mini reviews in medicinal chemistry | https://dx.doi.org/10.2174/1389557523666230725115324 |
| 32 | Developing Vaccines to Improve Preparedness for Filovirus Outbreaks: The Perspective of the USA Biomedical Advanced Research and Development Authority (BARDA) | Parish L.A.; Stavale E.J.; Houchens C.R.; Wolfe D.N. | 2023 | Vaccines | https://dx.doi.org/10.3390/vaccines11061120 |
| 33 | Ethnomedicinal review of plants utilized by the Abagusii people of Western Kenya | Onyancha J.M.; Moriasi G.A.; Nyandoro V.O.; Onyancha B.M.; Onsinyo J.M. | 2023 | Advances in Traditional Medicine | https://dx.doi.org/10.1007/s13596-023-00689-z |
| 34 | Defining system requirements for simplified blood culture to enable widespread use in resource-limited settings | Dailey P.J.; Osborn J.; Ashley E.A.; Baron E.J.; Dance D.A.B.; Fusco D.; Fanello C.; Manabe Y.C.; Mokomane M.; Newton P.N.; Tessema B.; Isaacs C.; Dittrich S. | 2019 | Diagnostics | https://dx.doi.org/10.3390/diagnostics9010010 |
| 35 | Approaches to demonstrating the effectiveness of filovirus vaccines: Lessons from Ebola and COVID-19 | Gruber M.F.; Rubin S.; Krause P.R. | 2023 | Frontiers in Immunology | https://dx.doi.org/10.3389/fimmu.2023.1109486 |
| 36 | Linking geospatial and laboratory sciences to define mechanisms behind landscape level drivers of anthrax outbreaks | Norris M.H.; Blackburn J.K. | 2019 | International Journal of Environmental Research and Public Health | https://dx.doi.org/10.3390/ijerph16193747 |
| 37 | Retrospective Screening of Anthrax-like Disease Induced by Bacillus tropicus str. JMT from Chinese Soft-Shell Turtles in Taiwan | Tsai J.-M.; Kuo H.-W.; Cheng W. | 2023 | Pathogens | https://dx.doi.org/10.3390/pathogens12050693 |
| 38 | DEVELOPMENT OF CONJUGATED SECONDARY ANTIBODIES FOR WILDLIFE DISEASE SURVEILLANCE | Ochai S.; Crafford J.; Kamath P.; Turner W.; van Heerden H. | 2023 | International Journal of Infectious Diseases | https://dx.doi.org/10.1016/j.ijid.2023.04.079 |
| 39 | PASTEUR, HIS NEPHEW, AND VETERINARY SCIENCE | Schwartz P.M. | 2022 | Bulletin de l'Academie Veterinaire de France | https://dx.doi.org/10.3406/bavf.2022.71012 |
| 40 | Proprotein convertases regulate trafficking and maturation of key proteins within the secretory pathway | Cendron L.; Rothenberger S.; Cassari L.; Dettin M.; Pasquato A. | 2023 | Advances in Protein Chemistry and Structural Biology | https://dx.doi.org/10.1016/bs.apcsb.2022.10.001 |
| 41 | Compulsory licensing of patents | Kumutha N.; Amutha N.; Venkatesh G.S. | 2022 | Pharmaceutical Patent Analyst | https://dx.doi.org/10.4155/ppa-2022-0039 |
| 42 | Endo-Lysosomal Cation Channels and Infectious Diseases | Chao Y.-K.; Chang S.-Y.; Grimm C. | 2023 | Reviews of Physiology, Biochemistry and Pharmacology | https://dx.doi.org/10.1007/112_2020_31 |
| 43 | The Use of Drones to Deliver Rift Valley Fever Vaccines in Rwanda: Perceptions and Recommendations | Griffith E.F.; Schurer J.M.; Mawindo B.; Kwibuka R.; Turibyarive T.; Amuguni J.H. | 2023 | Vaccines | https://dx.doi.org/10.3390/vaccines11030605 |
| 44 | Whole Genome Sequence of Bacillus anthracis MDMC_159, Isolated from the Moroccan Desert Erg Lihoudi | Manni A.; Laamarti M.; Azaroual S.E.; Kadmiri I.M.; Daoud R.; Allali A.E.; Filali-Maltouf A. | 2023 | Microbiology Resource Announcements | https://dx.doi.org/10.1128/mra.01209-22 |
| 45 | Detection of Bacillus anthracis in animal tissues using InBios active anthrax detect rapid test lateral flow immunoassay | Kolton C.B.; Marston C.K.; Stoddard R.A.; Cossaboom C.; Salzer J.S.; Kozel T.R.; Gates-Hollingsworth M.A.; Cleveland C.A.; Thompson A.T.; Dalton M.F.; Yabsley M.J.; Hoffmaster A.R. | 2019 | Letters in Applied Microbiology | https://dx.doi.org/10.1111/lam.13134 |
| 46 | Epidemics and the Military: Responding to COVID-19 in Uganda | Parker M.; Baluku M.; Ozunga B.E.; Okello B.; Kermundu P.; Akello G.; MacGregor H.; Leach M.; Allen T. | 2022 | Social Science and Medicine | https://dx.doi.org/10.1016/j.socscimed.2022.115482 |
| 47 | Multiplex peptide microarray profiling of antibody reactivity against neglected tropical diseases derived B-cell epitopes for serodiagnosis in Zimbabwe | Vengesai A.; Naicker T.; Midzi H.; Kasambala M.; Mduluza-Jokonya T.L.; Rusakaniko S.; Mutapi F.; Mduluza T. | 2022 | PLoS ONE | https://dx.doi.org/10.1371/journal.pone.0271916 |
| 48 | New formulation of a recombinant anthrax vaccine stabilised with structurally modified plant viruses | Granovskiy D.L.; Ryabchevskaya E.M.; Evtushenko E.A.; Kondakova O.A.; Arkhipenko M.V.; Kravchenko T.B.; Bakhteeva I.V.; Timofeev V.S.; Nikitin N.A.; Karpova O.V. | 2022 | Frontiers in Microbiology | https://dx.doi.org/10.3389/fmicb.2022.1003969 |
| 49 | Multisectoral prioritization of zoonotic diseases in Haryana (India) using one health approach | Thukral H.; Shanmugasundaram K.; Riyesh T.; Kumar N.; Singha H.; Gambhir D.; Laura A.; Tiwari S.; Gulati B.R. | 2023 | Preventive Veterinary Medicine | https://dx.doi.org/10.1016/j.prevetmed.2022.105835 |
| 50 | Assessment of community perceptions and risk to common zoonotic diseases among communities living at the human-livestock-wildlife interface in Nakuru West, Kenya: A participatory epidemiology approach | Owiny M.O.; Ngare B.K.; Mugo B.C.; Rotich J.; Mutembei A.; Chepkorir K.; Sitawa R.; Obonyo M.; Onono J.O. | 2023 | PLoS Neglected Tropical Diseases | https://dx.doi.org/10.1371/journal.pntd.0011086 |
| 51 | Pathogenicity and virulence of African trypanosomes: From laboratory models to clinically relevant hosts | Morrison L.J.; Steketee P.C.; Tettey M.D.; Matthews K.R. | 2023 | Virulence | https://dx.doi.org/10.1080/21505594.2022.2150445 |
| 52 | A Review of Laboratory-Acquired Infections in the Asia-Pacific: Understanding Risk and the Need for Improved Biosafety for Veterinary and Zoonotic Diseases | Siengsanan-Lamont J.; Blacksell S.D. | 2018 | Tropical Medicine and Infectious Disease | https://dx.doi.org/10.3390/tropicalmed3020036 |
| 53 | Midguts of Culex pipiens L. (Diptera: Culicidae) as a potential source of raw milk contamination with pathogens | Adly E.; Hegazy A.A.; Kamal M.; Abu-Hussien S.H. | 2022 | Scientific reports | https://dx.doi.org/10.1038/s41598-022-16992-9 |
| 54 | Participatory mapping identifies risk areas and environmental predictors of endemic anthrax in rural Africa | Aminu O.R.; Forde T.L.; Ekwem D.; Johnson P.; Nelli L.; Mmbaga B.T.; Mshanga D.; Shand M.; Shirima G.; Walsh M.; Zadoks R.N.; Biek R.; Lembo T. | 2022 | Scientific reports | https://dx.doi.org/10.1038/s41598-022-14081-5 |
| 55 | Bacterial and viral zoonotic infections: Bugging the world | Sheykhsaran E.; Hemmat N.; Leylabadlo H.E.; Baghi H.B. | 2022 | Reviews and Research in Medical Microbiology | https://dx.doi.org/10.1097/MRM.0000000000000273 |
| 56 | Biosafety of human environments can be supported by effective use of renewable biomass | Yu F.; Qin T.; Zhao W.; Chen Y.; Miao X.; Lin L.; Shang H.; Sui G.; Peng D.; Yang Y.; Zhu Y.; Zhang S.; Zhu X. | 2022 | Proceedings of the National Academy of Sciences of the United States of America | https://dx.doi.org/10.1073/pnas.2106843119 |
| 57 | Genomic Sequencing of Bacillus cereus Sensu Lato Strains Isolated from Meat and Poultry Products in South Africa Enables Inter- and Intranational Surveillance and Source Tracking | Carroll L.M.; Pierneef R.; Mathole A.; Atanda A.; Matle I. | 2022 | Microbiology Spectrum | https://dx.doi.org/10.1128/spectrum.00700-22 |
| 58 | The dual burden of animal and human zoonoses: A systematic review | Noguera Z. L.P.; Charypkhan D.; Hartnack S.; Torgerson P.R.; Ruegg S.R. | 2022 | PLoS Neglected Tropical Diseases | https://dx.doi.org/10.1371/journal.pntd.0010540 |
| 59 | Bioterrorism: An analysis of biological agents used in terrorist events | Tin D.; Sabeti P.; Ciottone G.R. | 2022 | American Journal of Emergency Medicine | https://dx.doi.org/10.1016/j.ajem.2022.01.056 |
| 60 | Competition between obligate and facultative scavengers and infection: vulture-jackal-anthrax dynamics in Etosha National Park | Kribs C.M.; Mackey C. | 2022 | Journal of Theoretical Biology | https://dx.doi.org/10.1016/j.jtbi.2021.110981 |
| 61 | Greater Horn of Africa's crown of thorns | Balakrishnan V.S. | 2022 | The Lancet Child and Adolescent Health | https://dx.doi.org/10.1016/S2352-4642%2822%2900253-X |
| 62 | mRNA vaccines and clinical research in Africa - From hope to reality | Saied A.A. | 2022 | International Journal of Surgery | https://dx.doi.org/10.1016/j.ijsu.2022.106833 |
| 63 | Effectiveness and profitability of preventive veterinary interventions in controlling infectious diseases of ruminant livestock in sub-Saharan Africa: a scoping review | Nuvey F.S.; Arkoazi J.; Hattendorf J.; Mensah G.I.; Addo K.K.; Fink G.; Zinsstag J.; Bonfoh B. | 2022 | BMC Veterinary Research | https://dx.doi.org/10.1186/s12917-022-03428-9 |
| 64 | The efficiency of estrus synchronization protocols and artificial insemination in the Abergelle goat on-station and on-farm conditions of Northern Ethiopia | Wondim B.; Taye M.; Alemayehu K.; Rouatbi M.; Getachew T.; Haile A.; Rekik M. | 2022 | Journal of Applied Animal Research | https://dx.doi.org/10.1080/09712119.2022.2108815 |
| 65 | Foodborne Pathogens and Antimicrobial Resistance in Ethiopia: An Urgent Call for Action on "One Health" | Asfaw T.; Genetu D.; Shenkute D.; Shenkutie T.T.; Amare Y.E.; Yitayew B. | 2022 | Infection and Drug Resistance | https://dx.doi.org/10.2147/IDR.S375043 |
| 66 | A multipathogen DNA vaccine elicits protective immune responses against two class A bioterrorism agents, anthrax and botulism | Kim N.Y.; Son W.R.; Lee M.H.; Choi H.S.; Choi J.Y.; Song Y.J.; Yu C.H.; Song D.H.; Hur G.H.; Jeong S.T.; Hong S.Y.; Shin Y.K.; Shin S. | 2022 | Applied Microbiology and Biotechnology | https://dx.doi.org/10.1007/s00253-022-11812-6 |
| 67 | A Systematic Review of Medicinal Plants of Kenya used in the Management of Bacterial Infections | Odongo E.A.; Mutai P.C.; Amugune B.K.; Mungai N.N. | 2022 | Evidence-based Complementary and Alternative Medicine | https://dx.doi.org/10.1155/2022/9089360 |
| 68 | Seasonality and Ecological Suitability Modelling for Anthrax (Bacillus anthracis) in Western Africa | Pittiglio C.; Shadomy S.; El Idrissi A.; Soumare B.; Lubroth J.; Makonnen Y. | 2022 | Animals | https://dx.doi.org/10.3390/ani12091146 |
| 69 | Population genomics of Bacillus anthracis from an anthrax hyperendemic area reveals transmission processes across spatial scales and unexpected within-host diversity | Forde T.L.; Dennis T.P.W.; Rhoda Aminu O.; Harvey W.T.; Hassim A.; Kiwelu I.; Medvecky M.; Mshanga D.; Van Heerden H.; Vogel A.; Zadoks R.N.; Mmbaga B.T.; Lembo T.; Biek R. | 2022 | Microbial Genomics | https://dx.doi.org/10.1099/mgen.0.000759 |
| 70 | Survey of Ethnoveterinary Medicines Used to Treat Livestock Diseases in Omusati and Kunene Regions of Namibia | Eiki N.; Maake M.; Lebelo S.; Sakong B.; Sebola N.; Mabelebele M. | 2022 | Frontiers in Veterinary Science | https://dx.doi.org/10.3389/fvets.2022.762771 |
| 71 | Immunological Evidence of Variation in Exposure and Immune Response to Bacillus anthracis in Herbivores of Kruger and Etosha National Parks | Ochai S.O.; Crafford J.E.; Hassim A.; Byaruhanga C.; Huang Y.-H.; Hartmann A.; Dekker E.H.; van Schalkwyk O.L.; Kamath P.L.; Turner W.C.; van Heerden H. | 2022 | Frontiers in Immunology | https://dx.doi.org/10.3389/fimmu.2022.814031 |
| 72 | One Health Paradigm to Confront Zoonotic Health Threats: A Pakistan Prospective | Yasmeen N.; Jabbar A.; Shah T.; Fang L.-X.; Aslam B.; Naseeb I.; Shakeel F.; Ahmad H.I.; Baloch Z.; Liu Y. | 2022 | Frontiers in Microbiology | https://dx.doi.org/10.3389/fmicb.2021.719334 |
| 73 | Risk factors for Brucellosis and knowledge-attitude practice among pastoralists in Afar and Somali regions of Ethiopia | Tschopp R.; GebreGiorgis A.; Abdulkadir O.; Molla W.; Hamid M.; Tassachew Y.; Andualem H.; Osman M.; Waqjira M.W.; Mohammed A.; Negron M.; Walke H.; Kadzik M.; Mamo G. | 2022 | Preventive Veterinary Medicine | https://dx.doi.org/10.1016/j.prevetmed.2021.105557 |
| 74 | The monetary impact of zoonotic diseases on society: The Turkish Case | Ari H.O.; Islek E.; Bilir Uslu M.K.; Ozatkan Y.; Karakas F.; Yildirim H.H.; Alp E. | 2022 | Ankara Universitesi Veteriner Fakultesi Dergisi | https://dx.doi.org/10.33988/auvfd.789598 |
| 75 | The potential distribution of Bacillus anthracis suitability across Uganda using INLA | Ndolo V.A.; Redding D.; Deka M.A.; Salzer J.S.; Vieira A.R.; Onyuth H.; Ocaido M.; Tweyongyere R.; Azuba R.; Monje F.; Ario A.R.; Kabwama S.; Kisaakye E.; Bulage L.; Kwesiga B.; Ntono V.; Harris J.; Wood J.L.N.; Conlan A.J.K. | 2022 | Scientific reports | https://dx.doi.org/10.1038/s41598-022-24281-8 |
| 76 | The evolution of how Guinea pigs are housed at high containment in the Biological Investigations Group | Emm L.; Heydon J. | 2022 | Animal Technology and Welfare |  |
| 77 | COST-EFFECTIVENESS OF DIFFERENT OPTIONS FOR VACCINATION OF LIVESTOCK AGAINST ANTHRAX IN UGANDA | Geofrey A.; Mwenge L.; Zambia L.; Fasanmi A.; Okello P.; Migisha R.; Mwonje F.; Kisaakye E.; Kadobera D.; Harris J.; Ario A.R. | 2022 | Journal of Public Health in Africa |  |
| 78 | Halting Epidemics at the Outset: Community-Based Surveillance (CBS) in an Epidemic Preparedness Model for Early Detection and Early Action | Byrne A. | 2022 | International Journal of Infectious Diseases | https://dx.doi.org/10.1016/j.ijid.2021.12.231 |
| 79 | Identification of Bacillus anthracis, Brucella spp., and Coxiella burnetii DNA signatures from bushmeat | Katani R.; Schilling M.A.; Lyimo B.; Eblate E.; Martin A.; Tonui T.; Cattadori I.M.; Francesconi S.C.; Estes A.B.; Rentsch D.; Srinivasan S.; Lyimo S.; Munuo L.; Tiambo C.K.; Stomeo F.; Gwakisa P.; Mosha F.; Hudson P.J.; Buza J.J.; Kapur V. | 2021 | Scientific reports | https://dx.doi.org/10.1038/s41598-021-94112-9 |
| 80 | Pathological findings in African buffaloes (Syncerus caffer) in South Africa | Woodburn D.B.; Steyl J.; Du Plessis E.C.; Last R.D.; Reininghaus B.; Mitchell E.P. | 2021 | Journal of the South African Veterinary Association | https://dx.doi.org/10.4102/JSAVA.V92I0.2117 |
| 81 | No hints for abundance of Bacillus anthracis and Burkholderia pseudomallei in 100 environmental samples from Cameroon B. anthracis and B. pseudomallei in Cameroon | Frickmann H.; Poppert S. | 2021 | European Journal of Microbiology and Immunology | https://dx.doi.org/10.1556/1886.2021.00014 |
| 82 | In other journals | Ash C.; Smith J. | 2021 | Science | https://dx.doi.org/10.1126/science.acx9639 |
| 83 | The role of Namibia Field Epidemiology and Laboratory Training Programme in strengthening the public health workforce in Namibia, 2012-2019 | Nyarko K.M.; Miller L.A.; Baughman A.L.; Katjiuanjo P.; Evering-Watley M.; Antara S.; Angula P.; Mitonga H.K.; Prybylski D.; Dziuban E.J.; Ndevaetela E.-E. | 2021 | BMJ Global Health | https://dx.doi.org/10.1136/bmjgh-2021-005597 |
| 84 | A Comprehensive Database and Geographical Distribution Model of Vectors and Vector Borne Diseases in Ardabil Province, Borderline of Iran and Azerbaijan Republic 2001-2018 | Adham D.; Vatandoost H.; Moradi-Asl E. | 2021 | Journal of Arthropod-Borne Diseases |  |
| 85 | A Survey of Priority Livestock Diseases and Laboratory Diagnostic Needs of Animal Health Professionals and Farmers in Uganda | Vudriko P.; Ekiri A.B.; Endacott I.; Williams S.; Gityamwi N.; Byaruhanga J.; Alafiatayo R.; Mijten E.; Tweyongyere R.; Varga G.; Cook A.J.C. | 2021 | Frontiers in Veterinary Science | https://dx.doi.org/10.3389/fvets.2021.721800 |
| 86 | Anthrax Surveillance and the Limited Overlap between Obligate Scavengers and Endemic Anthrax Zones in the United States | Walker M.A.; Uribasterra M.; Asher V.; Getz W.M.; Ryan S.J.; Ponciano J.M.; Blackburn J.K. | 2021 | Vector-Borne and Zoonotic Diseases | https://dx.doi.org/10.1089/vbz.2020.2747 |
| 87 | Viability evaluation of freeze dried and suspension anthrax spore vaccine formulations stored at different temperatures | Abayneh T.; Getachew B.; Gelaye E.; Traxler R.; Vieira A.R. | 2021 | Vaccine | https://dx.doi.org/10.1016/j.vaccine.2021.09.023 |
| 88 | Efficacy assessment of a triple anthrax chimeric antigen as a vaccine candidate in guinea pigs: challenge test with Bacillus anthracis 17 JB strain spores | Abdous M.; Hasannia S.; Salmanian A.H.; Arab S.-S. | 2021 | Immunopharmacology and Immunotoxicology | https://dx.doi.org/10.1080/08923973.2021.1945087 |
| 89 | Development of Digital Health Messages for Rural Populations in Tanzania: Multi- and Interdisciplinary Approach | Holst C.; Isabwe G.M.N.; Sukums F.; Ngowi H.; Kajuna F.; Radovanovic D.; Mansour W.; Mwakapeje E.; Cardellichio P.; Ngowi B.; Noll J.; Winkler A.S. | 2021 | JMIR mHealth and uHealth | https://dx.doi.org/10.2196/25558 |
| 90 | Disease or drought: environmental fluctuations release zebra from a potential pathogen-triggered ecological trap | Huang Y.-H.; Joel H.; Kusters M.; Barandongo Z.R.; Cloete C.C.; Hartmann A.; Kamath P.L.; Kilian J.W.; Mfune J.K.E.; Shatumbu G.; Zidon R.; Getz W.M.; Turner W.C. | 2021 | Proceedings of the Royal Society B: Biological Sciences | https://dx.doi.org/10.1098/rspb.2021.0582 |
| 91 | Uganda mountain community health system-perspectives and capacities towards emerging infectious disease surveillance | Siya A.; Mafigiri R.; Migisha R.; Kading R.C. | 2021 | International Journal of Environmental Research and Public Health | https://dx.doi.org/10.3390/ijerph18168562 |
| 92 | A putative exosporium lipoprotein GBAA0190 of Bacillus anthracis as a potential anthrax vaccine candidate | Jeon J.H.; Kim Y.H.; Kim K.A.; Kim Y.-R.; Woo S.-J.; Choi Y.J.; Rhie G.-E. | 2021 | BMC Immunology | https://dx.doi.org/10.1186/s12865-021-00414-y |
| 93 | Modeling the potential future distribution of anthrax outbreaks under multiple climate change scenarios for Kenya | Otieno F.T.; Gachohi J.; Gikuma-Njuru P.; Kariuki P.; Oyas H.; Canfield S.A.; Bett B.; Njenga M.K.; Blackburn J.K. | 2021 | International Journal of Environmental Research and Public Health | https://dx.doi.org/10.3390/ijerph18084176 |
| 94 | GPS Telemetry Reveals a Zebra With Anthrax as Putative Cause of Death for Three Cheetahs in the Namib Desert | Portas R.; Aschenborn O.H.K.; Melzheimer J.; Le Roux M.; Uiseb K.H.; Czirjak G.A.; Wachter B. | 2021 | Frontiers in Veterinary Science | https://dx.doi.org/10.3389/fvets.2021.714758 |
| 95 | One health in India: Time to act together | Dhaka P.; Bedi J.S.; Malik Y.S. | 2021 | Indian Journal of Animal Sciences |  |
| 96 | Assessment of major animal health problems and their impact on beef cattle production in doba district of west harerghe zone, ethiopia | Geletu U.S.; Musa A.A.; Waqe S.L.; Usmael M.A.; Mummed Y.Y.; Bari F.D.; Ibrahim A.M. | 2021 | Veterinary Medicine International | https://dx.doi.org/10.1155/2021/5533398 |
| 97 | Prioritization of zoonotic diseases of public health significance in Nigeria using the one-health approach | Ihekweazu C.; Michael C.A.; Nguku P.M.; Waziri N.E.; Habib A.G.; Muturi M.; Olufemi A.; Dzikwi-Emennaa A.A.; Balogun M.S.; Visa T.I.; Dalhat M.M.; Atama N.C.; Umeokonkwo C.D.; Mshelbwala G.M.; Vakuru C.T.; Okolocha E.C.; Umoh J.U.; Olugasa B.; Babalobi O.; Lombin L.; Cadmus S.; Sandhaus K.; Ricks P.M.; Ogunkoya A.; Aruna S.; Abubakar A.; Bidemi Y.; Njenga K.; Ibrahim G.; Adekanmbi O.; Nwadiuto I.; Hadejia I.S.; Nganda G.; Jacob K.; Owolodun O.; Christoper O.; Gandi Benjamin Tule T.Z.; Abdulrazak H.; Kwange D.; Kabiru S.; Muhammed G.M.; Joannis T.; Omilabu S.; Kabir J.; Ogundipe G.A.T.; Ojo O.; Joshua O.; Sadiq A.A.; Adebola O.; Magaji A.A.; Nasir A.; Duvall D.; Tekki S.; Ngulukun S.; Soruuke D.; Egumenu A.; Idiona I.; Ferdinand O.; Abayomi O.; Elsie I.; Tyakaray V.I.; Oyo-Ita A.; Ana G.; Alabi O.; Aworh M.; Kvagai J.; Chinyere G.; Gloria O. | 2021 | One Health | https://dx.doi.org/10.1016/j.onehlt.2021.100257 |
| 98 | Developing Recombinant Antibodies by Phage Display Against Infectious Diseases and Toxins for Diagnostics and Therapy | Roth K.D.R.; Wenzel E.V.; Ruschig M.; Steinke S.; Langreder N.; Heine P.A.; Schneider K.-T.; Ballmann R.; Fuhner V.; Kuhn P.; Schirrmann T.; Frenzel A.; Dubel S.; Schubert M.; Moreira G.M.S.G.; Bertoglio F.; Russo G.; Hust M. | 2021 | Frontiers in Cellular and Infection Microbiology | https://dx.doi.org/10.3389/fcimb.2021.697876 |
| 99 | Critical systematic review of zoonoses and transboundary animal diseases' prioritization in africa | Mpouam S.E.; Mingoas J.P.K.; Mouiche M.M.M.; Feussom J.M.K.; Saegerman C. | 2021 | Pathogens | https://dx.doi.org/10.3390/pathogens10080976 |
| 100 | The antimicrobial effect behind Cannabis sativa | Schofs L.; Sparo M.D.; Sanchez Bruni S.F. | 2021 | Pharmacology Research and Perspectives | https://dx.doi.org/10.1002/prp2.761 |
| 101 | Major zoonotic diseases of public health importance in Bangladesh | Chowdhury S.; Aleem M.A.; Khan M.S.I.; Hossain M.E.; Ghosh S.; Rahman M.Z. | 2021 | Veterinary Medicine and Science | https://dx.doi.org/10.1002/vms3.465 |
| 102 | An estimate of global anthrax prevalence in livestock: A meta-analysis | Sushma B.; Shedole S.; Suresh K.P.; Leena G.; Patil S.S.; Srikantha G. | 2021 | Veterinary World | https://dx.doi.org/10.14202/vetworld.2021.1263-1271 |
| 103 | Death detective | Kupferschmidt K. | 2021 | Science | https://dx.doi.org/10.1126/science.371.6528.454 |
| 104 | Modeling the spatial distribution of anthrax in southern kenya | Otieno F.T.; Gachohi J.; Gikuma-Njuru P.; Kariuki P.; Oyas H.; Canfield S.A.; Blackburn J.K.; Njenga M.K.; Bett B. | 2021 | PLoS Neglected Tropical Diseases | https://dx.doi.org/10.1371/journal.pntd.0009301 |
| 105 | High case-fatality rate for human anthrax, Northern Ghana, 2005-2016 | Blackburn J.K.; Kenu E.; Asiedu-Bekoe F.; Sarkodie B.; Kracalik I.T.; Bower W.A.; Stoddard R.A.; Traxler R.M. | 2021 | Emerging Infectious Diseases | https://dx.doi.org/10.3201/eid2704.204496 |
| 106 | A serological survey of Bacillus anthracis reveals widespread exposure to the pathogen in free-range and captive lions in Zimbabwe | Mukarati N.L.; Ndumnego O.C.; Ochai S.O.; Jauro S.; Loveridge A.; van Heerden H.; Matope G.; Caron A.; Hanyire T.G.; de Garine-Wichatitsky M.; Pfukenyi D.M. | 2021 | Transboundary and Emerging Diseases | https://dx.doi.org/10.1111/tbed.13842 |
| 107 | Small ruminant production in Tanzania, Uganda, and Ethiopia: A systematic review of constraints and potential solutions | Armson B.; Ekiri A.B.; Alafiatayo R.; Cook A.J. | 2021 | Veterinary Sciences | https://dx.doi.org/10.3390/vetsci8010005 |
| 108 | Cytotoxic and antimicrobial drimane meroterpenoids from a fungus of the Stictidaceae (Ostropales, Ascomycota) | Flores-Bocanegra L.; Augustinovic M.; Raja H.A.; Kurina S.J.; Maldonado A.C.; Burdette J.E.; Falkinham J.O.; Pearce C.J.; Oberlies N.H. | 2021 | Tetrahedron Letters | https://dx.doi.org/10.1016/j.tetlet.2021.152896 |
| 109 | Modeling anthrax-rabies interactions in zebra-jackal cycles | Mackey C.; Kribs C. | 2021 | Journal of Theoretical Biology | https://dx.doi.org/10.1016/j.jtbi.2020.110553 |
| 110 | Efficacy of the AV7909 anthrax vaccine candidate in guinea pigs and nonhuman primates following two immunizations two weeks apart | Shearer J.D.; Henning L.; Sanford D.C.; Li N.; Skiadopoulos M.H.; Reece J.J.; Ionin B.; Savransky V. | 2021 | Vaccine | https://dx.doi.org/10.1016/j.vaccine.2020.10.095 |
| 111 | Acoustofluidic device for acoustic capture of Bacillus anthracis spore analogues at low concentration | Plazonic F.; Fisher A.; Carugo D.; Hill M.; Glynne-Jones P. | 2021 | The Journal of the Acoustical Society of America | https://dx.doi.org/10.1121/10.0005278 |
| 112 | Omadacycline in Vitro Activity Against Bacillus Anthracis | Serio A.W.; Gelhaus H.C.; Eichelberger N.; Heine H.S.; Anastasiou D.M.; Eichhorst K. | 2021 | Open Forum Infectious Diseases | https://dx.doi.org/10.1093/ofid/ofab466.1400 |
| 113 | Convergent evolution of diverse Bacillus anthracis outbreak strains toward altered surface oligosaccharides that modulate anthrax pathogenesis | Norris M.H.; Kirpich A.; Bluhm A.P.; Zincke D.; Hadfield T.; Ponciano J.M.; Blackburn J.K. | 2020 | PLoS Biology | https://dx.doi.org/10.1371/journal.pbio.3001052 |
| 114 | Anthrax toxin component, Protective Antigen, protects insects from bacterial infections | Alameh S.; Bartolo G.; O'Brien S.; Henderson E.A.; Gonzalez L.O.; Hartmann S.; Klimko C.P.; Shoe J.L.; Cote C.K.; Grill L.K.; Levitin A.; Martchenko Shilman M. | 2020 | PLoS Pathogens | https://dx.doi.org/10.1371/journal.ppat.1008836 |
| 115 | Practical and effective diagnosis of animal anthrax in endemic low-resource settings | Aminu O.R.; Lembo T.; Zadoks R.N.; Biek R.; Lewis S.; Kiwelu I.; Mmbaga B.T.; Mshanga D.; Shirima G.; Denwood M.; Forde T.L. | 2020 | PLoS Neglected Tropical Diseases | https://dx.doi.org/10.1371/journal.pntd.0008655 |
| 116 | Ethnoveterinary Survey of Medicinal Plants Used for Treatment of Animal Diseases in Ambo District of Oromia Regional State of Ethiopia | Berhanu M.; Tintagu T.; Fentahun S.; Giday M. | 2020 | Evidence-based Complementary and Alternative Medicine | https://dx.doi.org/10.1155/2020/8816227 |
| 117 | Medicinal plants used to manage human and livestock ailments in Raya Kobo district of Amhara Regional State, Ethiopia | Osman A.; Sbhatu D.B.; Giday M. | 2020 | Evidence-based Complementary and Alternative Medicine | https://dx.doi.org/10.1155/2020/1329170 |
| 118 | Knowledge, attitude and practice towards anthrax in northern Ethiopia: a mixed approach study | Romha G.; Girmay W. | 2020 | BMC Infectious Diseases | https://dx.doi.org/10.1186/s12879-020-05544-z |
| 119 | Taqman assays for simultaneous detection of bacillus anthracis and bacillus cereus biovar anthracis | Zincke D.; Norris M.H.; Cruz O.; Kurmanov B.; McGraw W.S.; Daegling D.J.; Krigbaum J.; Hoang T.T.H.; Khanipov K.; Golovko G.; Hadfield T.; Blackburn J.K. | 2020 | Pathogens | https://dx.doi.org/10.3390/pathogens9121074 |
| 120 | Anthrax in the Amhara regional state of Ethiopia; spatiotemporal analysis and environmental suitability modeling with an ensemble approach | Assefa A.; Bihon A.; Tibebu A. | 2020 | Preventive Veterinary Medicine | https://dx.doi.org/10.1016/j.prevetmed.2020.105155 |
| 121 | Chronic ulcers and malnutrition in an African patient | Singer T.G.; Bray M.A.; Chan A.; Ikeda S.; Walters B.; Fuller M.Y.; Falco C. | 2020 | Pediatrics | https://dx.doi.org/10.1542/peds.2020-1717 |
| 122 | Perceptions and Practices towards Anthrax in Selected Agricultural Communities in Arua District, Uganda | Kungu J.M.; Nsamba P.; Wejuli A.; Kabasa J.D.; Bazeyo W. | 2020 | Journal of Tropical Medicine | https://dx.doi.org/10.1155/2020/9083615 |
| 123 | Environmental determinants influencing anthrax distribution in Queen Elizabeth Protected Area, Western Uganda | Driciru M.; Rwego I.B.; Ndimuligo S.A.; Travis D.A.; Mwakapeje E.R.; Craft M.; Asiimwe B.; Alvarez J.; Ayebare S.; Pelican K. | 2020 | PLoS ONE | https://dx.doi.org/10.1371/journal.pone.0237223 |
| 124 | Bacillus anthracis evolution: Taking advantage of the topology of the phylogenetic tree and human history to propose dating points | Vergnaud G. | 2020 | Erciyes Medical Journal | https://dx.doi.org/10.14744/etd.2020.64920 |
| 125 | Animal industry and veterinary science in eastern New Guinea: World War II to Independence, 1975 | Egerton J.R.; Rothwell T.L.W.; Harvey P.R.; Owen I.L.; Copland J.W. | 2020 | Australian Veterinary Journal | https://dx.doi.org/10.1111/avj.12945 |
| 126 | Bacillus paranthracis isolate from blood of fatal ebola virus disease case | Jeremiah Matson M.; Anzick S.L.; Feldmann F.; Martens C.A.; Drake S.K.; Feldmann H.; Massaquoi M.; Chertow D.S.; Munster V.J. | 2020 | Pathogens | https://dx.doi.org/10.3390/pathogens9060475 |
| 127 | Molecular characterization of B. anthracis isolates from the anthrax outbreak among cattle in Karnataka, India | Roonie A.; Majumder S.; Kingston J.J.; Parida M. | 2020 | BMC Microbiology | https://dx.doi.org/10.1186/s12866-020-01917-1 |
| 128 | Potential distributions of Bacillus anthracis and Bacillus cereus biovar anthracis causing anthrax in Africa | Romero-Alvarez D.; Peterson A.T.; Salzer J.S.; Pittiglio C.; Shadomy S.; Traxler R.; Vieira A.R.; Bower W.A.; Walke H.; Campbell L.P. | 2020 | PLoS Neglected Tropical Diseases | https://dx.doi.org/10.1371/journal.pntd.0008131 |
| 129 | Livestock owners' anthrax prevention practices and its associated factors in Sekota Zuria district, Northeast Ethiopia | Seid K.; Shiferaw A.M.; Yesuf N.N.; Derso T.; Sisay M. | 2020 | BMC Veterinary Research | https://dx.doi.org/10.1186/s12917-020-2267-0 |
| 130 | Nucleotide polymorphism assay for the identification of west African group Bacillus anthracis: A lineage lacking anthrose | Zincke D.; Norris M.H.; Kurmanov B.; Hadfield T.L.; Blackburn J.K. | 2020 | BMC Microbiology | https://dx.doi.org/10.1186/s12866-019-1693-2 |
| 131 | Development of a guinea pig inhalational anthrax model for evaluation of post-exposure prophylaxis efficacy of anthrax vaccines | Perry M.R.; Ionin B.; Barnewall R.E.; Vassar M.L.; Reece J.J.; Park S.; Lemiale L.; Skiadopoulos M.H.; Shearer J.D.; Savransky V. | 2020 | Vaccine | https://dx.doi.org/10.1016/j.vaccine.2020.01.068 |
| 132 | Early detection of public health emergencies of international concern through undiagnosed disease reports in ProMED-Mail | Rolland C.; Lazarus C.; Giese C.; Monate B.; Travert A.-S.; Salomon J. | 2020 | Emerging Infectious Diseases | https://dx.doi.org/10.3201/eid2602.191043 |
| 133 | Importance of livestock diseases identified using participatory epidemiology in the highlands of Ethiopia | Gizaw S.; Desta H.; Alemu B.; Tegegne A.; Wieland B. | 2020 | Tropical animal health and production | https://dx.doi.org/10.1007/s11250-019-02187-4 |
| 134 | Antibacterial, antibiofilm activity and cytotoxicity of crude extracts of Ptaeroxylon obliquum (Ptaeroxylaceae) used in South African ethnoveterinary medicine against Bacillus anthracis Sterne vaccine strain | Famuyide I.M.; Eloff J.N.; McGaw L.J. | 2020 | FASEB Journal | https://dx.doi.org/10.1096/fasebj.2020.34.s1.00560 |
| 135 | Knowledge concerning zoonoses among medical practitioners in Lusaka, Zambia | Chikuni G.S.; Mostafa E.M.; Minh L.H.N.; Sayed A.M.; Tawfik G.M.; Byskov J.; Huy N.T.; Hirayama K. | 2020 | American Journal of Tropical Medicine and Hygiene |  |
| 136 | Research Progress on Biosafety and Protection of Pathogenic Microorganisms | Cheng J.; Wang Q.; Lu X.; Meng C.; Wen W.; Wu Y.; Yu A.; Yang X. | 2020 | Basic and Clinical Pharmacology and Toxicology | https://dx.doi.org/10.1111/bcpt.13461 |
| 137 | Complete genome sequence of an environmental Bacillus cereus isolate belonging to the Bacillus anthracis clade | Irenge L.M.; Bearzatto B.; Ambroise J.; Gala J.-L. | 2020 | Microbiology Resource Announcements | https://dx.doi.org/10.1128/MRA.00917-20 |
| 138 | Phylogenomic structure of Bacillus anthracis isolates in the Northern Cape Province, South Africa revealed novel single nucleotide polymorphisms | Lekota K.E.; Hassim A.; Madoroba E.; Hefer C.A.; van Heerden H. | 2020 | Infection, Genetics and Evolution | https://dx.doi.org/10.1016/j.meegid.2019.104146 |
| 139 | Correction: Development and application of a Bacillus anthracis protective antigen domain-1 in-house ELISA for the detection of antiprotective antigen antibodies in cattle in Zambia (PLoS ONE (2018) 13:10 (e0205986) DOI: 10.1371/journal.pone.0205986) | Simbotwe M.; Fujikura D.; Ohnuma M.; Omori R.; Furuta Y.; Muuka G.M.; Hang'ombe B.M.; Higashi H. | 2019 | PLoS ONE | https://dx.doi.org/10.1371/journal.pone.0211592 |
| 140 | Rapid sequencing-based diagnosis of infectious bacterial species from meningitis patients in Zambia | Nakagawa S.; Inoue S.; Kryukov K.; Yamagishi J.; Ohno A.; Hayashida K.; Nakazwe R.; Kalumbi M.; Mwenya D.; Asami N.; Sugimoto C.; Mutengo M.M.; Imanishi T. | 2019 | Clinical and Translational Immunology | https://dx.doi.org/10.1002/cti2.1087 |
| 141 | Knowledge of pastoralists on livestock diseases and exposure assessment to brucellosis within rural and peri-urban areas in Kajiado, Kenya | Onono J.; Mutua P.; Kitala P.; Gathura P. | 2019 | F1000Research | https://dx.doi.org/10.12688/f1000research.20573.1 |
| 142 | Comparative knowledge, attitudes, and practices regarding anthrax, brucellosis, and rabies in three districts of northern Tanzania | Kiffner C.; Latzer M.; Vise R.; Benson H.; Hammon E.; Kioko J. | 2019 | BMC public health | https://dx.doi.org/10.1186/s12889-019-7900-0 |
| 143 | An overview on various biological warfare agents | Purba M.K.; Agrawal N.; Shukla S. | 2019 | Anil Aggrawal's Internet Journal of Forensic Medicine and Toxicology |  |
| 144 | Neglected zoonotic diseases in nigeria: Role of the public health veterinarian | Elelu N.; Aiyedun J.O.; Mohammed I.G.; Oludairo O.O.; Odetokun I.A.; Mohammed K.M.; Bale J.O.; Nuru S. | 2019 | Pan African Medical Journal | https://dx.doi.org/10.11604/pamj.2019.32.36.15659 |
| 145 | Ecological niche modeling as a tool for prediction of the potential geographic distribution of Bacillus anthracis spores in Tanzania | Mwakapeje E.R.; Ndimuligo S.A.; Mosomtai G.; Ayebare S.; Nyakarahuka L.; Nonga H.E.; Mdegela R.H.; Skjerve E. | 2019 | International Journal of Infectious Diseases | https://dx.doi.org/10.1016/j.ijid.2018.11.367 |
| 146 | Occurrence of bacteria and endotoxins in fermented foods and beverages from Nigeria and South Africa | Adekoya I.; Obadina A.; Olorunfemi M.; Akande O.; Landschoot S.; De Saeger S.; Njobeh P. | 2019 | International Journal of Food Microbiology | https://dx.doi.org/10.1016/j.ijfoodmicro.2019.108251 |
| 147 | Trends in the application of Bacillus in fermented foods | Kimura ( ) K.; Yokoyama ( ) S. | 2019 | Current Opinion in Biotechnology | https://dx.doi.org/10.1016/j.copbio.2018.09.001 |
| 148 | Identification and characterization of novel bacterial polyaromatic hydrocarbon-degrading enzymes as potential tools for cleaning up hydrocarbon pollutants from different environmental sources | Abdelhaleem H.A.R.; Zein H.S.; Azeiz A.; Sharaf A.N.; Abdelhadi A.A. | 2019 | Environmental Toxicology and Pharmacology | https://dx.doi.org/10.1016/j.etap.2019.02.009 |
| 149 | Application of Mixed Methods to Identify Small Ruminant Disease Priorities in Ethiopia | Alemu B.; Desta H.; Kinati W.; Mulema A.A.; Gizaw S.; Wieland B. | 2019 | Frontiers in Veterinary Science | https://dx.doi.org/10.3389/fvets.2019.00417 |
| 150 | Multivariate relationships between epidemiologic risk factors and zoonotic infections among military personnel in the country of Georgia: A non-linear canonical correlation analysis | Akhvlediani T.; Chitadze N.; Chlikadze R.; Rostiashvili N.; Betashvili M.; Imnadze P.; Rivard R.G.; Nikolich M.P.; Washington M.A.; Bautista C.T. | 2019 | Zoonoses and Public Health | https://dx.doi.org/10.1111/zph.12632 |
| 151 | Comparative immunogenicity and efficacy of thermostable (lyophilized) and liquid formulation of anthrax vaccine candidate AV7909 | Autumn Smiley M.; Sanford D.C.; Triplett C.A.; Callahan D.; Frolov V.; Look J.; Ruiz C.; Reece J.J.; Miles A.; Ruiz E.; Ionin B.; Shearer J.D.; Savransky V. | 2019 | Vaccine | https://dx.doi.org/10.1016/j.vaccine.2019.09.015 |
| 152 | The global distribution of Bacillus anthracis and associated anthrax risk to humans, livestock and wildlife | Carlson C.J.; Kracalik I.T.; Ross N.; Alexander K.A.; Hugh-Jones M.E.; Fegan M.; Elkin B.T.; Epp T.; Shury T.K.; Zhang W.; Bagirova M.; Getz W.M.; Blackburn J.K. | 2019 | Nature Microbiology | https://dx.doi.org/10.1038/s41564-019-0435-4 |
| 153 | UK vaccines network: Mapping priority pathogens of epidemic potential and vaccine pipeline developments | Noad R.J.; Simpson K.; Fooks A.R.; Hewson R.; Gilbert S.C.; Stevens M.P.; Hosie M.J.; Prior J.; Kinsey A.M.; Entrican G.; Simpson A.; Whitty C.J.M.; Carroll M.W. | 2019 | Vaccine | https://dx.doi.org/10.1016/j.vaccine.2019.09.009 |
| 154 | Depiction of secondary metabolites and antifungal activity of Bacillus velezensis DTU001 | Devi S.; Kiesewalter H.T.; Kovacs R.; Frisvad J.C.; Weber T.; Larsen T.O.; Kovacs A.T.; Ding L. | 2019 | Synthetic and Systems Biotechnology | https://dx.doi.org/10.1016/j.synbio.2019.08.002 |
| 155 | Ethnomedicinal, pharmacological and phytochemistry of aegle marmelos (Linn.) corr: A review | Singh S.; Ajeet Singh N. | 2019 | International Journal of Pharmaceutical Sciences Review and Research |  |
| 156 | Antigenic spectrum of causative agent of anthrax | Galiullin A.K.; Zadorina I.I.; Sofronov P.V.; Sadykov N.S.; Mustafina E.N.; Melnikova L.A.; Ivanova S.V. | 2019 | Research Journal of Pharmaceutical, Biological and Chemical Sciences |  |
| 157 | A new triple chimeric protein as a high immunogenic antigen against anthrax toxins: theoretical and experimental analyses | Abdous M.; Hasannia S.; Salmanian A.H.; Shahryar Arab S.; Shali A.; Alizadeh G.A.; Hajizadeh A.; Khafri A.; Mohseni A. | 2019 | Immunopharmacology and Immunotoxicology | https://dx.doi.org/10.1080/08923973.2018.1510419 |
| 158 | Formaldehyde and glutaraldehyde inactivation of bacterial tier 1 select agents in tissues | Chua J.; Bozue J.A.; Klimko C.P.; Shoe J.L.; Ruiz S.I.; Jensen C.L.; Tobery S.A.; Crumpler J.M.; Chabot D.J.; Quirk A.V.; Hunter M.; Harbourt D.E.; Friedlander A.M.; Cote C.K. | 2019 | Emerging Infectious Diseases | https://dx.doi.org/10.3201/eid2505.180928 |
| 159 | Plague vaccine: recent progress and prospects | Sun W.; Singh A.K. | 2019 | npj Vaccines | https://dx.doi.org/10.1038/s41541-019-0105-9 |
| 160 | Tropical rainforest flies carrying pathogens form stable associations with social nonhuman primates | Gogarten J.F.; Dux A.; Mubemba B.; Pleh K.; Hoffmann C.; Mielke A.; Muller-Tiburtius J.; Sachse A.; Wittig R.M.; Calvignac-Spencer S.; Leendertz F.H. | 2019 | Molecular ecology | https://dx.doi.org/10.1111/mec.15145 |
| 161 | A participatory epidemiological study of major cattle diseases amongst Maasai pastoralists living in wildlife-livestock interfaces in Maasai Mara, Kenya | Nthiwa D.; Alonso S.; Odongo D.; Kenya E.; Bett B. | 2019 | Tropical animal health and production | https://dx.doi.org/10.1007/s11250-018-01790-1 |
| 162 | Analytical performance of the Filmarray global fever panel | Helm J.R.; Toxopeus C.; Belgique P.; Border L.; Jackson O.; Kelley A.; Mortenson M.; Phillips C. | 2019 | American Journal of Tropical Medicine and Hygiene | https://dx.doi.org/10.4269/ajtmh.abstract2019 |
| 163 | Clinical evaluation of the Filmarray global fever panel | Jones B.W.; Rabiger D.; Gurling M.A.; Smith W.; Veloz M.; Jackson O.; King N.; Burton M.; Shorter C.; Andjelic C.D.; Phillips C.L. | 2019 | American Journal of Tropical Medicine and Hygiene | https://dx.doi.org/10.4269/ajtmh.abstract2019 |
| 164 | Geographic distribution of cattle anthrax in Western Zambia | Simbotwe M.; Mulenga E.; Furuta Y.; Munkombwe Muuka G.; Hang'ombe B.M.; Higashi H. | 2019 | Japanese Journal of Veterinary Research | https://dx.doi.org/10.14943/jjvr.67.2.195 |
| 165 | Whole genome sequencing and identification of Bacillus endophyticus and B. anthracis isolated from anthrax outbreaks in South Africa | Lekota K.E.; Bezuidt O.K.I.; Mafofo J.; Rees J.; Muchadeyi F.C.; Madoroba E.; Van Heerden H. | 2018 | BMC Microbiology | https://dx.doi.org/10.1186/s12866-018-1205-9 |
| 166 | Spores and soil from six sides: interdisciplinarity and the environmental biology of anthrax (Bacillus anthracis) | Carlson C.J.; Getz W.M.; Kausrud K.L.; Cizauskas C.A.; Blackburn J.K.; Bustos Carrillo F.A.; Colwell R.; Easterday W.R.; Ganz H.H.; Kamath P.L.; Okstad O.A.; Turner W.C.; Kolsto A.-B.; Stenseth N.C. | 2018 | Biological reviews of the Cambridge Philosophical Society | https://dx.doi.org/10.1111/brv.12420 |
| 167 | Development and application of a Bacillus anthracis protective antigen domain-1 in-house ELISA for the detection of anti-protective antigen antibodies in cattle in Zambia | Simbotwe M.; Fujikura D.; Ohnuma M.; Omori R.; Furuta Y.; Muuka G.M.; Hang'ombe B.M.; Higashi H. | 2018 | PLoS ONE | https://dx.doi.org/10.1371/journal.pone.0205986 |
| 168 | Genotyping and phylogenetic placement of Bacillus anthracis isolates from Finland, a country with rare anthrax cases | Lienemann T.; Beyer W.; Pelkola K.; Rossow H.; Rehn A.; Antwerpen M.; Grass G. | 2018 | BMC Microbiology | https://dx.doi.org/10.1186/s12866-018-1250-4 |
| 169 | Loss of bacitracin resistance due to a large genomic deletion among bacillus anthracis strains | Furuta Y.; Harima H.; Ito E.; Maruyama F.; Ohnishi N.; Osaki K.; Ogawa H.; Squarre D.; Hang'Ombe B.M.; Higashi H. | 2018 | mSystems | https://dx.doi.org/10.1128/mSystems.00182-18 |
| 170 | Immunological Efficacy and Safety of Synthesized CpG Oligodeoxynucleotides | Kudriavtseva O.M.; Semakova A.P.; Mikshis N.I.; Popova P.Y.; Kozhevnikov V.A.; Stepanov A.V.; Bugorkova S.A. | 2018 | Applied Biochemistry and Microbiology | https://dx.doi.org/10.1134/S0003683818090041 |
| 171 | Spatio-temporal patterns of foot-and-mouth disease transmission in cattle between 2007 and 2015 and quantitative assessment of the economic impact of the disease in Niger | Souley Kouato B.; Thys E.; Renault V.; Abatih E.; Marichatou H.; Issa S.; Saegerman C. | 2018 | Transboundary and Emerging Diseases | https://dx.doi.org/10.1111/tbed.12845 |
| 172 | A modified anthrax toxin-based enzyme-linked immunospot assay reveals robust T cell responses in symptomatic and asymptomatic Ebola virus exposed individuals | Herrera B.B.; Hamel D.J.; Oshun P.; Akinsola R.; Akanmu A.S.; Chang C.A.; Eromon P.; Folarin O.; Adeyemi K.T.; Happi C.T.; Lu Y.; Ogunsola F.; Kanki P.J. | 2018 | PLoS Neglected Tropical Diseases | https://dx.doi.org/10.1371/journal.pntd.0006530 |
| 173 | Multisectoral prioritization of zoonotic diseases in Uganda, 2017: A One Health perspective | Sekamatte M.; Krishnasamy V.; Bulage L.; Kihembo C.; Nantima N.; Monje F.; Ndumu D.; Sentumbwe J.; Mbolanyi B.; Aruho R.; Kaboyo W.; Mutonga D.; Basler C.; Paige S.; Behravesh C.B. | 2018 | PLoS ONE | https://dx.doi.org/10.1371/journal.pone.0196799 |
| 174 | Ecological suitability modeling for anthrax in the Kruger National Park, South Africa | Steenkamp P.J.; Van Heerden H.; van Schalkwyk O.L. | 2018 | PLoS ONE | https://dx.doi.org/10.1371/journal.pone.0191704 |
| 175 | Veterinary medicinal product usage among food animal producers and its health implications in Central Ethiopia | Tufa T.B.; Gurmu F.; Beyi A.F.; Hogeveen H.; Beyene T.J.; Ayana D.; Woldemariyam F.T.; Hailemariam E.; Gutema F.D.; Stegeman J.A. | 2018 | BMC Veterinary Research | https://dx.doi.org/10.1186/s12917-018-1737-0 |
| 176 | A review of the ethnomedicinal uses, phytochemistry and pharmacological properties of ekebergia capensis sparrm | Maroyi A. | 2018 | Asian Journal of Pharmaceutical and Clinical Research | https://dx.doi.org/10.22159/ajpcr.2018.v11i10.28816 |
| 177 | Health workers' knowledge of zoonotic diseases in an endemic region of Western Uganda | Benon A.B.; Juliet K.; Samuel M.; Catherine K.; Benjamin S.; Michael M.; Innocent R.B. | 2018 | Zoonoses and Public Health | https://dx.doi.org/10.1111/zph.12509 |
| 178 | Contribution of the French army health service in support of expertise and research in infectiology in Africa | Pradines B.; Rogier C. | 2018 | New Microbes and New Infections | https://dx.doi.org/10.1016/j.nmni.2018.05.008 |
| 179 | Use of Canonical Single Nucleotide Polymorphism (CanSNPs) to characterize Bacillus anthracis outbreak strains in Zambia between 1990 and 2014 | Fasanella A.; Serrecchia L.; Chiaverini A.; Garofolo G.; Muuka G.M.; Mwambazi L. | 2018 | PeerJ | https://dx.doi.org/10.7717/peerj.5270 |
| 180 | Sustained specific and cross-reactive T cell responses to Zika and dengue virus NS3 in West Africa | Herrera B.B.; Tsai W.-Y.; Chang C.A.; Hamel D.J.; Wang W.-K.; Lu Y.; Mboup S.; Kanki P.J. | 2018 | Journal of Virology | https://dx.doi.org/10.1128/JVI.01992-17 |
| 181 | Isolation and identification of antibiotic producing microorganisms from soil | Rafiq A.; Khan S.A.; Akbar A.; Shafi M.; Ali I.; Rehman F.U.; Rashid R.; Shakoor G.; Anwar M. | 2018 | International Journal of Pharmaceutical Sciences and Research | https://dx.doi.org/10.13040/IJPSR.0975-8232.9%283%29.1002-11 |
| 182 | Assessing behavioral risk factors for disease transmission at the human-animal interface in Laikipia County, Kenya-2017 | Shields L.M.; Kamau J.; Ambala P.; Kwallah A.; Ashby E.; Vodzak M.; Murray S.; Zimmerman D. | 2018 | American Journal of Tropical Medicine and Hygiene |  |
| 183 | Genomic and molecular characterization of the bacterial populations observed in the water of breeding sites and in larvae of anopheles coluzzii and anopheles gambiae in nanguilabougou and kouroubabougou | Diarra A.D. | 2018 | American Journal of Tropical Medicine and Hygiene |  |
| 184 | Lay perceptions, beliefs and practices linked to the persistence of anthrax outbreaks in cattle in the Western Province of Zambia | Sitali D.C.; Twambo M.C.; Chisoni M.; Bwalya M.J.; Munyeme M. | 2018 | The Onderstepoort journal of veterinary research | https://dx.doi.org/10.4102/ojvr.v85i1.1615 |
| 185 | DUST-BATHING BEHAVIORS OF AFRICAN HERBIVORES AND THE POTENTIAL RISK OF INHALATIONAL ANTHRAX | Barandongo Z.R.; Mfune J.K.E.; Turner W.C. | 2018 | Journal of wildlife diseases | https://dx.doi.org/10.7589/2017-04-069 |
| 186 | Cross-sectional survey of brucellosis and associated risk factors in the livestock-wildlife interface area of Nechisar National Park, Ethiopia | Chaka H.; Aboset G.; Garoma A.; Gumi B.; Thys E. | 2018 | Tropical animal health and production | https://dx.doi.org/10.1007/s11250-018-1528-4 |
| 187 | Use of human immunoglobulins as an anti-infective treatment: the experience so far and their possible re-emerging role | Bozzo J.; Jorquera J.I. | 2017 | Expert Review of Anti-Infective Therapy | https://dx.doi.org/10.1080/14787210.2017.1328278 |
| 188 | Temporal dynamics in microbial soil communities at anthrax carcass sites | Valseth K.; Nesbo C.L.; Easterday W.R.; Turner W.C.; Olsen J.S.; Stenseth N.C.; Haverkamp T.H.A. | 2017 | BMC Microbiology | https://dx.doi.org/10.1186/s12866-017-1111-6 |
| 189 | Use of the mice passive protection test to evaluate the humoral response in goats vaccinated with Sterne 34F2 live spore vaccine | Phaswana P.H.; Ndumnego O.C.; Koehler S.M.; Beyer W.; Crafford J.E.; Van Heerden H. | 2017 | Veterinary Research | https://dx.doi.org/10.1186/s13567-017-0451-4 |
| 190 | Bacterial species and mycotoxin contamination associated with locust bean, melon and their fermented products in south-western Nigeria | Adedeji B.S.; Ezeokoli O.T.; Ezekiel C.N.; Obadina A.O.; Somorin Y.M.; Sulyok M.; Adeleke R.A.; Warth B.; Nwangburuka C.C.; Omemu A.M.; Oyewole O.B.; Krska R. | 2017 | International Journal of Food Microbiology | https://dx.doi.org/10.1016/j.ijfoodmicro.2017.07.014 |
| 191 | Assisting differential clinical diagnosis of cattle diseases using smartphone-based technology in low resource settings: A pilot study | Beyene T.J.; Eshetu A.; Abdu A.; Wondimu E.; Beyi A.F.; Tufa T.B.; Ibrahim S.; Revie C.W. | 2017 | BMC Veterinary Research | https://dx.doi.org/10.1186/s12917-017-1249-3 |
| 192 | Correlation between anthrax lethal toxin neutralizing antibody levels and survival in guinea pigs and nonhuman primates vaccinated with the AV7909 anthrax vaccine candidate | Savransky V.; Shearer J.D.; Gainey M.R.; Sanford D.C.; Sivko G.S.; Stark G.V.; Li N.; Ionin B.; Lacy M.J.; Skiadopoulos M.H. | 2017 | Vaccine | https://dx.doi.org/10.1016/j.vaccine.2017.07.076 |
| 193 | A novel live attenuated anthrax spore vaccine based on an acapsular Bacillus anthracis Sterne strain with mutations in the htrA, lef and cya genes | Chitlaru T.; Israeli M.; Rotem S.; Elia U.; Bar-Haim E.; Ehrlich S.; Cohen O.; Shafferman A. | 2017 | Vaccine | https://dx.doi.org/10.1016/j.vaccine.2017.03.033 |
| 194 | Resource-driven encounters among consumers and implications for the spread of infectious disease | Borchering R.K.; Bellan S.E.; Flynn J.M.; Pulliam J.R.C.; McKinley S.A. | 2017 | Journal of the Royal Society Interface | https://dx.doi.org/10.1098/rsif.2017.0555 |
| 195 | Diagnosis of cutaneous anthrax in resource-poor settings in west Arsi Province, Ethiopia | Perez-Tanoira R.; Ramos J.M.; Prieto-Perez L.; Tesfamariam A.; Balcha S.; Tissiano G.; Cabello A.; Cuadros J.; Rodriguez-Valero N.; Barreiro P.; Reyes F.; Gorgolas M. | 2017 | Annals of Agricultural and Environmental Medicine | https://dx.doi.org/10.26444/aaem/80705 |
| 196 | Structural and immunochemical relatedness suggests a conserved pathogenicity motif for secondary cell wall polysaccharides in Bacillus anthracis and infection-associated Bacillus cereus | Kamal N.; Ganguly J.; Saile E.; Klee S.R.; Hoffmaster A.; Carlson R.W.; Forsberg L.S.; Kannenberg E.L.; Quinn C.P. | 2017 | PLoS ONE | https://dx.doi.org/10.1371/journal.pone.0183115 |
| 197 | A systematic review and meta-analysis of preclinical trials testing anti-toxin therapies for B. anthracis infection: A need for more robust study designs and results | Xu W.; Ohanjandian L.; Sun J.; Cui X.; Suffredini D.; Li Y.; Welsh J.; Eichacker P.Q. | 2017 | PLoS ONE | https://dx.doi.org/10.1371/journal.pone.0182879 |
| 198 | Epidemic insurance | Cohen J. | 2017 | Science | https://dx.doi.org/10.1126/science.356.6334.125 |
| 199 | Particulate delivery systems for vaccination against bioterrorism agents and emerging infectious pathogens | Fan Y.; Moon J.J. | 2017 | Wiley Interdisciplinary Reviews: Nanomedicine and Nanobiotechnology | https://dx.doi.org/10.1002/wnan.1403 |
| 200 | Model systems for pulmonary infectious diseases: Paradigms of anthrax and tuberculosis | Arora G.; Misra R.; Sajid A. | 2017 | Current Topics in Medicinal Chemistry | https://dx.doi.org/10.2174/1568026617666170130111324 |
| 201 | Pathology of wild-type and toxin-independent Bacillus anthracis meningitis in rabbits | Sittner A.; Bar-David E.; Glinert I.; Ben-Shmuel A.; Weiss S.; Schlomovitz J.; Kobiler D.; Levy H. | 2017 | PLoS ONE | https://dx.doi.org/10.1371/journal.pone.0186613 |
| 202 | Low antibody prevalence against Bacillus cereus biovar anthracis in Tai National Park, Cote d'Ivoire, indicates high rate of lethal infections in wildlife | Zimmermann F.; Kohler S.M.; Nowak K.; Dupke S.; Barduhn A.; Dux A.; Lang A.; De Nys H.M.; Gogarten J.F.; Grunow R.; Couacy-Hymann E.; Wittig R.M.; Klee S.R.; Leendertz F.H. | 2017 | PLoS Neglected Tropical Diseases | https://dx.doi.org/10.1371/journal.pntd.0005960 |
| 203 | Medicinal plants used by traditional healers in Sangurur, Elgeyo Marakwet County, Kenya | Kigen G.; Kipkore W.; Wanjohi B.; Haruki B.; Kemboi J. | 2017 | Pharmacognosy Research | https://dx.doi.org/10.4103/pr.pr_42_17 |
| 204 | Modeling the environmental suitability of anthrax in Ghana and estimating populations at risk: Implications for vaccination and control | Kracalik I.T.; Kenu E.; Ayamdooh E.N.; Allegye-Cudjoe E.; Polkuu P.N.; Frimpong J.A.; Nyarko K.M.; Bower W.A.; Traxler R.; Blackburn J.K. | 2017 | PLoS Neglected Tropical Diseases | https://dx.doi.org/10.1371/journal.pntd.0005885 |
| 205 | Antimicrobial Activity of 1,2,5-Trimethylpiperidin-4-Ol Derivatives | Dyusebaeva M.A.; Elibaeva N.S.; Kalugin S.N. | 2017 | Pharmaceutical Chemistry Journal | https://dx.doi.org/10.1007/s11094-017-1652-x |
| 206 | Analytical performance of the filmarray global fever panel | Helm J.R.; Toxopeus C.; Batty N.; Davidson O.; Marble B.; Gnade B.T.; Fernandez S.; Phillips C. | 2017 | American Journal of Tropical Medicine and Hygiene |  |
| 207 | Diagnosis of cutaneous anthrax with the naked eye in resource poor settings | Perez-Tanoira R.; Ramos J.M.; Prieto-Perez L.; Cabello A.; Carrillo I.; Cuadros J.; Reyes F.; Tesfamariam A.; Gorgolas M. | 2017 | Tropical Medicine and International Health | https://dx.doi.org/10.1111/%28ISSN%291365-3156 |
| 208 | Connecting the unconnected in sub-Saharan Africa: Nondiscriminating access for digital inclusion with an emphasis on health (DigI) | Holst C.; Knobloch J.; Schmidt V.; Mwakapeje E.; Ngowi H.; Ngowi B.; Prazeres Da Costa C.; Brugge B.; Winkler A.S.; Noll J. | 2017 | Tropical Medicine and International Health | https://dx.doi.org/10.1111/%28ISSN%291365-3156 |
| 209 | Extracellular release of non-peptide group compounds by antifungal Bacillus and Brevibacillus strains | Shrivastava A.; Gupta M.K.; Singhal P.K.; Shrivastava P. | 2017 | Current Bioactive Compounds | https://dx.doi.org/10.2174/1573407212666160804124019 |
| 210 | Antimicrobial use and veterinary care among agro-pastoralists in Northern Tanzania | Caudell M.A.; Quinlan M.B.; Subbiah M.; Call D.R.; Roulette C.J.; Roulette J.W.; Roth A.; Matthews L.; Quinlan R.J. | 2017 | PLoS ONE | https://dx.doi.org/10.1371/journal.pone.0170328 |
| 211 | Awareness and attitudes towards anthrax and meat consumption practices among affected communities in Zambia: A mixed methods approach | Sitali D.C.; Mumba C.; Skjerve E.; Mweemba O.; Kabonesa C.; Mwinyi M.O.; Nyakarahuka L.; Muma J.B. | 2017 | PLoS Neglected Tropical Diseases | https://dx.doi.org/10.1371/journal.pntd.0005580 |
| 212 | Exploring local knowledge and perceptions on zoonoses among pastoralists in northern and eastern Tanzania | Mangesho P.E.; Neselle M.O.; Karimuribo E.D.; Mlangwa J.E.; Queenan K.; Mboera L.E.G.; Rushton J.; Kock R.; Hasler B.; Kiwara A.; Rweyemamu M. | 2017 | PLoS Neglected Tropical Diseases | https://dx.doi.org/10.1371/journal.pntd.0005345 |
| 213 | Antimicrobial activity of Zanthoxylum holtizianum (Engl.) waterm and Zanthoxylum lindense (Engl.) Kokwaro growing in Bagamoyo district, Coast region, Tanzania | Runyoro D.K.B.; Ngassapa O.D.; Masimba P.; Peter B. | 2017 | Journal of Pharmaceutical Sciences and Research |  |
| 214 | CDC's Evolving Approach to Emergency Response | Redd S.C.; Frieden T.R. | 2017 | Health security | https://dx.doi.org/10.1089/hs.2017.0006 |
| 215 | Effectiveness of a mobile short-message-service-based disease outbreak alert system in Kenya | Toda M.; Njeru I.; Zurovac D.; O-Tipo S.; Kareko D.; Mwau M.; Morita K. | 2016 | Emerging Infectious Diseases | https://dx.doi.org/10.3201/eid2204.151459 |
| 216 | Reanalysis of the anthrax epidemic in Rhodesia, 1978-1984 | Wilson J.M.; Brediger W.; Albright T.P.; Smith-Gagen J. | 2016 | PeerJ | https://dx.doi.org/10.7717/peerj.2686 |
| 217 | Cu(ii) complex of a new isoindole derivative: structure, catecholase like activity, antimicrobial properties and bio-molecular interactions | Adhikari S.; Lohar S.; Kumari B.; Banerjee A.; Bandopadhyay R.; Matalobos J.S.; Das D. | 2016 | New Journal of Chemistry | https://dx.doi.org/10.1039/c6nj02193j |
| 218 | The microbiological impact of pulsed xenon ultraviolet disinfection on resistant bacteria, bacterial spore and fungi and viruses | Stibich M.; Stachowiak J. | 2016 | Southern African Journal of Infectious Diseases | https://dx.doi.org/10.4102/sajid.v31i1.103 |
| 219 | Images in clinical tropical medicine: Black eschars in the highlands of Ethiopia | Perez-Tanoira R.; Cuadros J.; Prieto-Perez L. | 2016 | American Journal of Tropical Medicine and Hygiene | https://dx.doi.org/10.4269/ajtmh.15-0763 |
| 220 | Chlamydia-related bacteria in free-living and captive great apes, Gabon | Klockner A.; Nagel M.; Greub G.; Aeby S.; Hoffmann K.; Liegeois F.; Rouet F.; De Benedetti S.; Borel N.; Henrichfreise B. | 2016 | Emerging Infectious Diseases | https://dx.doi.org/10.3201/eid2212.150893 |
| 221 | Polio infrastructure strengthened disease outbreak preparedness and response in the WHO African Region | Kouadio K.; Okeibunor J.; Nsubuga P.; Mihigo R.; Mkanda P. | 2016 | Vaccine | https://dx.doi.org/10.1016/j.vaccine.2016.05.070 |
| 222 | Polio Eradication Initiative: Contribution to improved communicable diseases surveillance in WHO African region | Mwengee W.; Okeibunor J.; Poy A.; Shaba K.; Mbulu Kinuani L.; Minkoulou E.; Yahaya A.; Gaturuku P.; Landoh D.E.; Nsubuga P.; Salla M.; Mihigo R.; Mkanda P. | 2016 | Vaccine | https://dx.doi.org/10.1016/j.vaccine.2016.05.060 |
| 223 | Comparison of French and worldwide bacillus anthracis strains favors a recent, post-columbian origin of the predominant North-American clade | Vergnaud G.; Girault G.; Thierry S.; Pourcel C.; Madani N.; Blouin Y. | 2016 | PLoS ONE | https://dx.doi.org/10.1371/journal.pone.0146216 |
| 224 | A candidate transacting modulator of fetal hemoglobin gene expression in the Arab-Indian haplotype of sickle cell anemia | Vathipadiekal V.; Farrell J.J.; Wang S.; Edward H.L.; Shappell H.; Al-Rubaish A.M.; Al-Muhanna F.; Naserullah Z.; Alsuliman A.; Qutub H.O.; Simkin I.; Farrer L.A.; Jiang Z.; Luo H.-Y.; Huang S.; Mostoslavsky G.; Murphy G.J.; Patra P.K.; Chui D.H.K.; Alsultan A.; Al-Ali A.K.; Sebastiani P.; Steinberg M.H. | 2016 | American Journal of Hematology | https://dx.doi.org/10.1002/ajh.24527 |
| 225 | Polyphasic characterization of Bacillus species from anthrax outbreaks in animals from South Africa and Lesotho | Lekota K.E.; Hassim A.; Mafofo J.; Rees J.; Muchadeyi F.C.; van Heerden H.; Madoroba E. | 2016 | Journal of Infection in Developing Countries | https://dx.doi.org/10.3855/jidc.7798 |
| 226 | Terahertz spectroscopy for bacterial detection: opportunities and challenges | Yang X.; Yang K.; Luo Y.; Fu W. | 2016 | Applied Microbiology and Biotechnology | https://dx.doi.org/10.1007/s00253-016-7569-6 |
| 227 | Serologic evidence of the geographic distribution of bacterial zoonotic agents in Kenya, 2007 | Omballa V.O.; Musyoka R.N.; Vittor A.Y.; Wamburu K.B.; Wachira C.M.; Waiboci L.W.; Abudo M.U.; Juma B.W.; Kim A.A.; Montgomery J.M.; Breiman R.F.; Fields B.S. | 2016 | American Journal of Tropical Medicine and Hygiene | https://dx.doi.org/10.4269/ajtmh.15-0320 |
| 228 | Under fire in Tanzania | Grijsen M.L.; Kaderbhai H.S.; Hamers R.L.; Masenga J.E. | 2016 | Nederlands Tijdschrift voor Dermatologie en Venereologie |  |
| 229 | Editorial | Cotton M. | 2016 | Tropical Doctor | https://dx.doi.org/10.1177/0049475516659015 |
| 230 | Preface | Katz S.A.; Salem H. | 2016 | Issues in Toxicology | https://dx.doi.org/10.1039/9781849737913-FP007 |
| 231 | Inhalational anthrax - Issues in dose-response and hazard evaluation | Falk A.; Eisenkraft A. | 2016 | Issues in Toxicology | https://dx.doi.org/10.1039/9781849737913-00072 |
| 232 | Mixed Methods Survey of Zoonotic Disease Awareness and Practice among Animal and Human Healthcare Providers in Moshi, Tanzania | Zhang H.L.; Mnzava K.W.; Mitchell S.T.; Melubo M.L.; Kibona T.J.; Cleaveland S.; Kazwala R.R.; Crump J.A.; Sharp J.P.; Halliday J.E.B. | 2016 | PLoS Neglected Tropical Diseases | https://dx.doi.org/10.1371/journal.pntd.0004476 |
| 233 | Strebloside, a Constituent of Streblus asper with Antineoplastic Activity | Kinghorn A.D.; Ren Y.; Chen W.L.; Lantvit D.D.; Ngoc Ninh T.; Sass E.J.; Chai H.B.; Zhang X.; Soejarto D.D.; Lucas D.M.; Swanson S.M.; Burdette J.E. | 2016 | Planta Medica | https://dx.doi.org/10.1055/s-0036-1596114 |
| 234 | Identifying challenges and opportunities for one health systems strengthening in guinea | Standley C.J.; Carlin E.; Sorrell E.M.; Barry A.M.; Diakite A.S.; Koivogui L.; Keita M.S.; Mane S.; Martel L.; Katz R.L. | 2016 | American Journal of Tropical Medicine and Hygiene | https://dx.doi.org/10.4269/ajtmh.abstract2016 |
| 235 | Detection of antibody inhibition of influenza H5N1 binding to a sialoglycan receptor using surface plasmon resonance (SPR) and its use as a neutralizing antibody screening assay | Norton M.G.; Khalenkov A.; Kamikawa T.L.; Kort T.; Pushko P.; Kennedy M.C.; Scott D.E. | 2016 | Glycobiology | https://dx.doi.org/10.1093/glycob/cww110 |
| 236 | In Vivo DNA-monoclonal antibody (DMAb) gene delivery protects against lethal bacterial and viral challenges in mice | Patel A.; Flingai S.; Elliott S.T.C.; Smith T.; Wise M.C.; Ramos S.; Yan J.; Schultheis K.; Keaton A.A.; Park D.H.; Broderick K.E.; Sardesai N.; Karuppiah M.; Weiner D.B. | 2016 | Molecular Therapy | https://dx.doi.org/10.1038/mt.2016.78 |
| 237 | Prioritization of zoonotic diseases in Kenya, 2015 | Munyua P.; Bitek A.; Osoro E.; Pieracci E.G.; Muema J.; Mwatondo A.; Kungu M.; Nanyingi M.; Gharpure R.; Njenga K.; Thumbi S.M. | 2016 | PLoS ONE | https://dx.doi.org/10.1371/journal.pone.0161576 |
| 238 | Bacillus cereus Biovar Anthracis Causing Anthrax in Sub-Saharan Africa-Chromosomal Monophyly and Broad Geographic Distribution | Antonation K.S.; Grutzmacher K.; Dupke S.; Mabon P.; Zimmermann F.; Lankester F.; Peller T.; Feistner A.; Todd A.; Herbinger I.; de Nys H.M.; Muyembe-Tamfun J.-J.; Karhemere S.; Wittig R.M.; Couacy-Hymann E.; Grunow R.; Calvignac-Spencer S.; Corbett C.R.; Klee S.R.; Leendertz F.H. | 2016 | PLoS Neglected Tropical Diseases | https://dx.doi.org/10.1371/journal.pntd.0004923 |
| 239 | Comparative analysis of the immunologic response induced by the Sterne 34F2 live spore Bacillus anthracis vaccine in a ruminant model | Ndumnego O.C.; Kohler S.M.; Crafford J.; van Heerden H.; Beyer W. | 2016 | Veterinary Immunology and Immunopathology | https://dx.doi.org/10.1016/j.vetimm.2016.06.005 |
| 240 | Extracellular release of non-peptide group compounds by antifungal Bacillus and Brevibacillus strains | Shrivastava A.; Gupta M.K.; Singhal P.K.; Shrivastava P. | 2016 | Current Bioactive Compounds | https://dx.doi.org/10.2174/1573407212666160804124019 |
| 241 | Health Care Providers' Knowledge and Practice Gap towards Joint Zoonotic Disease Surveillance System: Challenges and Opportunities, Gomma District, Southwest Ethiopia | Gemeda D.H.; Sime A.G.; Hajito K.W.; Gelalacha B.D.; Tafese W.; Gebrehiwot T.T. | 2016 | BioMed Research International | https://dx.doi.org/10.1155/2016/3942672 |
| 242 | Antitrypanosomal activity of Verbascum sinaiticum Benth. (Scrophulariaceae) against Trypanosoma congolense isolates | Mergia E.; Shibeshi W.; Terefe G.; Teklehaymanot T. | 2016 | BMC Complementary and Alternative Medicine | https://dx.doi.org/10.1186/s12906-016-1346-z |
| 243 | Knowledge, attitudes and practices towards spotted fever group rickettsioses and Q fever in Laikipia and Maasai Mara, Kenya | Ndeereh D.; Muchemi G.; Thaiyah A. | 2016 | Journal of Public Health in Africa | https://dx.doi.org/10.4081/jphia.2016.545 |
| 244 | Ethnopharmacological survey of the medicinal plants used in Tindiret, Nandi county, Kenya | Kigen G.; Maritim A.; Some F.; Kibosia J.; Rono H.; Chepkwony S.; Kipkore W.; Wanjoh B. | 2016 | African Journal of Traditional, Complementary and Alternative Medicines | https://dx.doi.org/10.4314/ajtcam.v13i3.19 |
| 245 | Living at the edge of an interface area in Zimbabwe: cattle owners, commodity chain and health workers' awareness, perceptions and practices on zoonoses | Gadaga B.M.; Etter E.M.; Mukamuri B.; Makwangudze K.J.; Pfukenyi D.M.; Matope G. | 2016 | BMC public health | https://dx.doi.org/10.1186/s12889-016-2744-3 |
| 246 | Next-Generation Bacillus anthracis Live Attenuated Spore Vaccine Based on the htrA(-) (High Temperature Requirement A) Sterne Strain | Chitlaru T.; Israeli M.; Bar-Haim E.; Elia U.; Rotem S.; Ehrlich S.; Cohen O.; Shafferman A. | 2016 | Scientific reports | https://dx.doi.org/10.1038/srep18908 |
| 247 | Snippet | Desikan P. | 2015 | Indian Journal of Medical Microbiology | https://dx.doi.org/10.4103/0255-0857.158613 |
| 248 | Snippets | Desikan P. | 2015 | Indian Journal of Medical Microbiology | https://dx.doi.org/10.4103/0255-0857.154904 |
| 249 | Fighting Ebola with novel spore decontamination technologies for the military | Doona C.J.; Feeherry F.E.; Kustin K.; Olinger G.G.; Setlow P.; Malkin A.J.; Leighton T. | 2015 | Frontiers in Microbiology | https://dx.doi.org/10.3389/fmicb.2015.00663 |
| 250 | Periorbital cellulitis due to cutaneous anthrax | Gilliland G.; Starks V.; Vrcek I.; Gilliland C. | 2015 | International Ophthalmology | https://dx.doi.org/10.1007/s10792-015-0057-7 |
| 251 | Diseases of livestock in the Pacific Islands region: Setting priorities for food animal biosecurity | Brioudes A.; Warner J.; Hedlefs R.; Gummow B. | 2015 | Acta Tropica | https://dx.doi.org/10.1016/j.actatropica.2014.12.012 |
| 252 | Bacillus anthracis diversity and geographic potential across Nigeria, Cameroon and chad: Further support of a Novel West African Lineage | Blackburn J.K.; Odugbo M.O.; Van Ert M.; O'Shea B.; Mullins J.; Perrenten V.; Maho A.; Hugh-Jones M.; Hadfield T. | 2015 | PLoS Neglected Tropical Diseases | https://dx.doi.org/10.1371/journal.pntd.0003931 |
| 253 | Human anthrax as a re-emerging disease | Doganay M.; Demiraslana H. | 2015 | Recent Patents on Anti-Infective Drug Discovery | http://dx.doi.org/10.2174/1574891X10666150408162354 |
| 254 | The neglected zoonoses-the case for integrated control and advocacy | Welburn S.C.; Beange I.; Ducrotoy M.J.; Okello A.L. | 2015 | Clinical Microbiology and Infection | https://dx.doi.org/10.1016/j.cmi.2015.04.011 |
| 255 | Seasonal patterns of hormones, macroparasites, and microparasites in wild african ungulates: The interplay among stress, reproduction, and disease | Cizauskas C.A.; Turner W.C.; Pitts N.; Getz W.M. | 2015 | PLoS ONE | https://dx.doi.org/10.1371/journal.pone.0120800 |
| 256 | Capsules, Toxins and AtxA as Virulence Factors of Emerging Bacillus cereus Biovar anthracis | Brezillon C.; Haustant M.; Dupke S.; Corre J.-P.; Lander A.; Franz T.; Monot M.; Couture-Tosi E.; Jouvion G.; Leendertz F.H.; Grunow R.; Mock M.E.; Klee S.R.; Goossens P.L. | 2015 | PLoS Neglected Tropical Diseases | https://dx.doi.org/10.1371/journal.pntd.0003455 |
| 257 | A novel multiplex PCR discriminates Bacillus anthracis and its genetically related strains from other Bacillus cereus group species | Ogawa H.; Fujikura D.; Ohnuma M.; Ohnishi N.; Hang'ombe B.M.; Mimuro H.; Ezaki T.; Mweene A.S.; Higashi H. | 2015 | PLoS ONE | https://dx.doi.org/10.1371/journal.pone.0122004 |
| 258 | Antibacterial activity of ethnomedicinal plants of irulars of western ghats | Shirley R.; Growther L. | 2015 | International Journal of Pharmaceutical Sciences and Research | https://dx.doi.org/10.13040/IJPSR.0975-8232.6%287%29.2837-40 |
| 259 | University of Pretoria One Health Summer School 2014 | Shotton J. | 2015 | Journal of Comparative Pathology | https://dx.doi.org/10.1016/j.jcpa.2014.11.002 |
| 260 | Synthesis and in-vitro antimicrobial studies of some new pyrazolones | Gupta P.; Gupta J.K.; Bansal S.; Halve A.K. | 2015 | International Journal of Current Pharmaceutical Research |  |
| 261 | Characterizing zoonotic disease detection in the United States: Who detects zoonotic disease outbreaks & how fast are they detected? | Allen H.A. | 2015 | Journal of Infection and Public Health | https://dx.doi.org/10.1016/j.jiph.2014.09.009 |
| 262 | Genetic characterization of Bacillus anthracis 17 JB strain | Seyed-Mohamadi S.; Bidhendi S.M.; Tadayon K.; Ghaderi R. | 2015 | Iranian Journal of Microbiology |  |
| 263 | Vaccine Preventable Diseases Surveillance and Response: The Role of a Public Health Institute | Kebede A. | 2015 | Pan African Medical Journal | https://dx.doi.org/10.11604/pamj.2015.21.208.7258 |
| 264 | Mixed methods survey of zoonotic disease awareness and practice among animal and human healthcare providers in Moshi, Tanzania | Zhang H.L.; Mnzava K.W.; Mitchell S.T.; Melubo M.L.; Kibona T.J.; Sharp J.P.; Kazwala R.R.; Cleaveland S.; Crump J.A.; Halliday J.E. | 2015 | American Journal of Tropical Medicine and Hygiene |  |
| 265 | Serologic evidence for the geographic distribution of bacterial zoonotic agents in Kenya | Omballa V.O.; Musyoka R.N.; Wamburu K.B.; Wachira C.M.; Waiboci L.W.; Abudo M.U.; Juma B.W.; Kim A.A.; Montgomery J.M.; Breiman R.F.; Fields B.S. | 2015 | American Journal of Tropical Medicine and Hygiene |  |
| 266 | A candidate trans-acting modulator of fetal hemoglobin gene expression in the Arab-Indian haplotype of sickle cell anemia | Vathipadiekal V.; Farrell J.; Shuai Z.; Edward H.; Shappell H.; Al-Rubaish A.M.; Al-Muhanna F.; Naserullah Z.; Alsuliman A.; Simkin I.; Farrer L.; Jiang Z.; Luo H.Y.; Huang S.; Mostoslavsky G.; Murphy G.J.; Patra P.K.; Chui D.H.K.; Alsultan A.; Al-Ali A.; Sebastiani P.; Steinberg M.H. | 2015 | Blood |  |
| 267 | Bacillus cereus biovar anthracis-an emerging pathogen affecting African rain forest areas | Klee S.; Brezillon C.; Dupke S.; Beudje F.; Gragnon B.; Zimmermann F.; Franz T.; Lander A.; Brzuszkiewicz E.; Liesegang H.; Grunow R.; Couacy-Hymann E.; Goossens P.; Leendertz F. | 2015 | International Journal of Medical Microbiology |  |
| 268 | A survey of UK acute clinicians' knowledge of personal protective requirements for infectious diseases and chemical, biological, and radiological warfare agents | Bond A.R.; Buckingham A.; Schumacher J. | 2015 | Critical Care | https://dx.doi.org/10.1186/cc14166 |
| 269 | Chemical and biological terrorist attacks identified through the Global Terrorism Database | Vakkalanka J.P.; Parker Cote J.L.; Schwartz R.; King J.D.; Charlton N.P.; Holstege C.P. | 2015 | Clinical Toxicology | https://dx.doi.org/10.3109/15563650.2015.1024953 |
| 270 | Optimization of avian influenza surveillance for human health and poultry production in Benghazi, Libya | Kollars T.M. | 2015 | Eastern Journal of Medicine |  |
| 271 | Bacillus cereus from the environment is genetically related to the highly pathogenic B. cereus in Zambia | Ogawa H.; Ohnuma M.; Squarre D.; Mweene A.S.; Ezaki T.; Fujikura D.; Ohnishi N.; Thomas Y.; Hang'ombe B.M.; Higashi H. | 2015 | The Journal of veterinary medical science | https://dx.doi.org/10.1292/jvms.15-0059 |
| 272 | Crossing institutional boundaries: mapping the policy process for improved control of endemic and neglected zoonoses in sub-Saharan Africa | Okello A.; Welburn S.; Smith J. | 2015 | Health policy and planning | https://dx.doi.org/10.1093/heapol/czu059 |
| 273 | Evaluation of Inhaled Versus Deposited Dose Using the Exponential Dose-Response Model for Inhalational Anthrax in Nonhuman Primate, Rabbit, and Guinea Pig | Gutting B.W.; Rukhin A.; Mackie R.S.; Marchette D.; Thran B. | 2015 | Risk analysis : an official publication of the Society for Risk Analysis | https://dx.doi.org/10.1111/risa.12326 |
| 274 | The worldwide distribution of genetically and phylogenetically diverse Bacillus cereus isolates harbouring Bacillus anthracis-like plasmids | Kaminska P.S.; Yernazarova A.; Drewnowska J.M.; Zambrowski G.; Swiecicka I. | 2015 | Environmental microbiology reports | https://dx.doi.org/10.1111/1758-2229.12305 |
| 275 | Animal Models for the Pathogenesis, Treatment, and Prevention of Infection by Bacillus anthracis | Welkos S.; Bozue J.; Twenhafel N.; Cote C. | 2015 | Microbiology spectrum | https://dx.doi.org/10.1128/microbiolspec.TBS-0001-2012 |
| 276 | Characteristics and phylogeny of Bacillus cereus strains isolated from Maari, a traditional West African food condiment | Thorsen L.; Kando C.K.; Sawadogo H.; Larsen N.; Diawara B.; Ouedraogo G.A.; Hendriksen N.B.; Jespersen L. | 2015 | International journal of food microbiology | https://dx.doi.org/10.1016/j.ijfoodmicro.2014.11.026 |
| 277 | Mapping of major diseases and devising prevention and control regimen to common diseases in cattle and shoats in Dassenech district of South Omo Zone, South-Western Ethiopia | Molla B.; Delil F. | 2015 | Tropical animal health and production | https://dx.doi.org/10.1007/s11250-014-0681-7 |
| 278 | The role of the state in stock farming in rural areas: A case study of Hertzog, Eastern Cape, South Africa | Jenjezwa V.R.; Seethal C.E.P. | 2014 | Journal of the South African Veterinary Association | https://dx.doi.org/10.4102/jsava.v85i1.912 |
| 279 | Ethnoveterinary medicinal plants used by the Maale and Ari ethnic communities in southern Ethiopia | Kidane B.; Van Der Maesen L.J.G.; Van Andel T.; Asfaw Z. | 2014 | Journal of Ethnopharmacology | https://dx.doi.org/10.1016/j.jep.2014.02.031 |
| 280 | Zoonoses in the Arabian Peninsula | Wernery U. | 2014 | Saudi Medical Journal | https://www.ncbi.nlm.nih.gov/pubmed/?term=25491209 |
| 281 | Evaluation of the notifiable diseases surveillance system in sanyati district, Zimbabwe, 2010-2011 | Maponga B.A.; Chirundu D.; Shambira G.; Gombe N.T.; Tshimanga M.; Bangure D. | 2014 | Pan African Medical Journal | https://dx.doi.org/10.11604/pamj.2014.19.278.5202 |
| 282 | The central nervous system as target of Bacillus anthracis toxin independent virulence in rabbits and guinea pigs | Levy H.; Glinert I.; Weiss S.; Bar-David E.; Sittner A.; Schlomovitz J.; Altboum Z.; Kobiler D. | 2014 | PLoS ONE | https://dx.doi.org/10.1371/journal.pone.0112319 |
| 283 | In trans complementation of lethal factor reveal roles in colonization and dissemination in a murine mouse model | Lowe D.E.; Ya J.; Glomski I.J. | 2014 | PLoS ONE | https://dx.doi.org/10.1371/journal.pone.0095950 |
| 284 | The experiments conducted by Japanese on human guinea pigs, And the use of biological weapons during the Sino-Japanese war (1937-1945) | Sabbatani S. | 2014 | Infezioni in Medicina |  |
| 285 | Immunoproteomically identified GBAA_0345, alkyl hydroperoxide reductase subunit C is a potential target for multivalent anthrax vaccine | Kim Y.H.; Kim K.A.; Kim Y.-R.; Choi M.K.; Kim H.K.; Choi K.J.; Chun J.-H.; Cha K.; Hong K.-J.; Lee N.G.; Yoo C.-K.; Oh H.-B.; Kim T.S.; Rhie G.-E. | 2014 | Proteomics | https://dx.doi.org/10.1002/pmic.201200495 |
| 286 | Factors influencing performance of internet-based biosurveillance systems used in epidemic intelligence for early detection of infectious diseases outbreaks | Barboza P.; Vaillant L.; Strat Y.L.; Hartley D.M.; Nelson N.P.; Mawudeku A.; Madoff L.C.; Linge J.P.; Collier N.; Brownstein J.S.; Astagneau P. | 2014 | PLoS ONE | https://dx.doi.org/10.1371/journal.pone.0090536 |
| 287 | Human body preservation - old and new techniques | Brenner E. | 2014 | Journal of Anatomy | https://dx.doi.org/10.1111/joa.12160 |
| 288 | Ebola: Biotech goes on counterattack | Scott C. | 2014 | BioProcess International |  |
| 289 | Anthrax meningoencephalitis: A case following a cutaneous lesion in Morocco | Ziadi A.; Hachimi A.; Soraa N.; Tassi N.; Nejmi H.; Elkhayari M.; Samkaoui M.A. | 2014 | Annales Francaises d'Anesthesie et de Reanimation | https://dx.doi.org/10.1016/j.annfar.2014.03.003 |
| 290 | Travel-associated pneumonias | Geerdes-Fenge H.F. | 2014 | Pneumologie | https://dx.doi.org/10.1055/s-0034-1378081 |
| 291 | Novel giant siphovirus from Bacillus anthracis features unusual genome characteristics | Ganz H.H.; Law C.; Schmuki M.; Eichenseher F.; Calendar R.; Loessner M.J.; Getz W.M.; Korlach J.; Beyer W.; Klumpp J. | 2014 | PLoS ONE | https://dx.doi.org/10.1371/journal.pone.0085972 |
| 292 | Toxin-independent virulence of Bacillus anthracis in rabbits | Levy H.; Glinert I.; Weiss S.; Sittner A.; Schlomovitz J.; Altboum Z.; Kobiler D. | 2014 | PLoS ONE | https://dx.doi.org/10.1371/journal.pone.0084947 |
| 293 | Limnophila (Scrophulariaceae): Chemical and pharmaceutical aspects-an update | Brahmachari G. | 2014 | Open Natural Products Journal |  |
| 294 | Protein- and DNA-based anthrax toxin vaccines confer protection in guinea pigs against inhalational challenge with Bacillus cereus G9241 | Palmer J.; Bell M.; Darko C.; Barnewall R.; Keane-Myers A. | 2014 | Pathogens and Disease | https://dx.doi.org/10.1111/2049-632X.12204 |
| 295 | Cutaneous anthrax in an African setting | Franken L.; Grossmann H.; Sanders C.J.G. | 2014 | Nederlands Tijdschrift voor Dermatologie en Venereologie |  |
| 296 | Frequent and seasonally variable sublethal anthrax infections are accompanied by short-lived immunity in an endemic system | Cizauskas C.A.; Bellan S.E.; Turner W.C.; Vance R.E.; Getz W.M. | 2014 | The Journal of animal ecology | https://dx.doi.org/10.1111/1365-2656.12207 |
| 297 | Gastrointestinal helminths may affect host susceptibility to anthrax through seasonal immune trade-offs | Cizauskas C.A.; Turner W.C.; Wagner B.; Kusters M.; Vance R.E.; Getz W.M. | 2014 | BMC ecology | https://dx.doi.org/10.1186/s12898-014-0027-3 |
| 298 | Fatal attraction: vegetation responses to nutrient inputs attract herbivores to infectious anthrax carcass sites | Turner W.C.; Kausrud K.L.; Krishnappa Y.S.; Cromsigt J.P.; Ganz H.H.; Mapaure I.; Cloete C.C.; Havarua Z.; Kusters M.; Getz W.M.; Stenseth N.C. | 2014 | Proceedings. Biological sciences / The Royal Society | https://dx.doi.org/10.1098/rspb.2014.1785 |
| 299 | Selected infectious disease disasters for nursing staff training at Egyptian Eastern Border | El-Bahnasawy M.M.; Labib N.A.; Abdel-Fattah M.A.; Ibrahim A.M.; Morsy T.A. | 2014 | Journal of the Egyptian Society of Parasitology | http://dx.doi.org/10.12816/0006445 |
| 300 | Retrospective review of the case of cutaneous anthrax-malignant pustule from 1995 in 15-year old girl | Kajfasz P.; Bartoszcze M.; Borkowski P.K.; Basiak W. | 2014 | Przeglad epidemiologiczny | https://www.ncbi.nlm.nih.gov/pubmed/?term=25848786 |
| 301 | [Immunogenicity and safety of a prototype chemical anthrax vaccine in laboratory animal models] | Mikshis N.I.; Popova P.Iu.; Kudriavtseva O.M.; Semakova A.P.; Novikova L.N.; Kravtsov A.L.; Bugorkova S.A.; Shchukovskaia T.N.; Popov Iu.A.; Kutyrev V.V. | 2014 | Zhurnal mikrobiologii, epidemiologii, i immunobiologii | https://www.ncbi.nlm.nih.gov/pubmed/?term=25286524 |
| 302 | Variation in herbivore space use: comparing two savanna ecosystems with different anthrax outbreak patterns in southern Africa. | Huang, Yen-Hua; Owen-Smith, Norman; Henley, Michelle D; Kilian, J Werner; Kamath, Pauline L; Ochai, Sunday O; van Heerden, Henriette; Mfune, John K E; Getz, Wayne M; Turner, Wendy C | 2023 | Movement ecology | https://dx.doi.org/10.1186/s40462-023-00385-2 |
| 303 | An epidemiological synthesis of emerging and re-emerging zoonotic disease threats in Cameroon, 2000-2022: a systematic review. | Tahmo, Nancy B; Wirsiy, Frankline Sevidzem; Nnamdi, Dum-Buo; Tongo, Marcel; Lawler, James V; Broadhurst, M Jana; Wondji, Charles S; Brett-Major, David M | 2023 | IJID regions | https://dx.doi.org/10.1016/j.ijregi.2022.12.001 |
| 304 | The World Health Organization's Disease Outbreak News: A retrospective database. | Carlson, Colin J; Boyce, Matthew R; Dunne, Margaret; Graeden, Ellie; Lin, Jessica; Abdellatif, Yasser Omar; Palys, Max A; Pavez, Munir; Phelan, Alexandra L; Katz, Rebecca | 2023 | PLOS global public health | https://dx.doi.org/10.1371/journal.pgph.0001083 |
| 305 | A framework for integrating inferred movement behavior into disease risk models. | Dougherty, Eric R; Seidel, Dana P; Blackburn, Jason K; Turner, Wendy C; Getz, Wayne M | 2022 | Movement ecology | https://dx.doi.org/10.1186/s40462-022-00331-8 |
| 306 | Therapeutic prospects of endophytic Bacillus species from Berberis lycium against oxidative stress and microbial pathogens. | Nisa, Sobia; Shoukat, Mubarra; Bibi, Yamin; Al Ayoubi, Samha; Shah, Waqas; Masood, Saadia; Sabir, Maimoona; Asma Bano, Syeda; Qayyum, Abdul | 2022 | Saudi journal of biological sciences | https://dx.doi.org/10.1016/j.sjbs.2021.08.099 |
| 307 | Using a Syrian (Golden) Hamster Biological Model for the Evaluation of Recombinant Anthrax Vaccines. | Kravchenko, Tatiana; Titareva, Galina; Bakhteeva, Irina; Kombarova, Tatiana; Borzilov, Alexander; Mironova, Raisa; Khlopova, Kseniya; Timofeev, Vitalii | 2021 | Life (Basel, Switzerland) | https://dx.doi.org/10.3390/life11121388 |
| 308 | Anthrax prevention practice and associated factors among farmers in Farta district, South Gondar, Amhara region, Northwest Ethiopia. | Mesfin, Dereje; Mulatu, Kebadnew; Birara, Amsalu; Shibabaw, Tebkew; Birhanu, Dereje; Yalew, Wubante | 2021 | Heliyon | https://dx.doi.org/10.1016/j.heliyon.2021.e08531 |
| 309 | Prioritizing zoonotic diseases using a multisectoral, One Health approach for The Economic Community of West African States (ECOWAS). | Goryoka, Grace W; Lokossou, Virgil Kuassi; Varela, Kate; Oussayef, Nadia; Kofi, Bernard; Iwar, Vivian; Behravesh, Casey Barton | 2021 | One health outlook | https://dx.doi.org/10.1186/s42522-021-00055-6 |
| 310 | Direct Regulons of AtxA, the Master Virulence Regulator of Bacillus anthracis. | Furuta, Yoshikazu; Cheng, Cheng; Zorigt, Tuvshinzaya; Paudel, Atmika; Izumi, Shun; Tsujinouchi, Mai; Shimizu, Tomoko; Meijer, Wim G; Higashi, Hideaki | 2021 | mSystems | https://dx.doi.org/10.1128/mSystems.00291-21 |
| 311 | Conventional knowledge, general attitudes and risk perceptions towards zoonotic diseases among Maasai in northern Tanzania. | Kriegel, E R; Cherney, D J R; Kiffner, C | 2021 | Heliyon | https://dx.doi.org/10.1016/j.heliyon.2021.e07041 |
| 312 | Zoonotic disease preparedness in sub-Saharan African countries. | Elton, Linzy; Haider, Najmul; Kock, Richard; Thomason, Margaret J; Tembo, John; Arruda, Lia Barbara; Ntoumi, Francine; Zumla, Alimuddin; McHugh, Timothy D | 2021 | One health outlook | https://dx.doi.org/10.1186/s42522-021-00037-8 |
| 313 | Detection and Profiling of Antibiotic Resistance among Culturable Bacterial Isolates in Vended Food and Soil Samples. | Muriuki, Susan W; Neondo, Johnstone O; Budambula, Nancy L M | 2020 | International journal of microbiology | https://dx.doi.org/10.1155/2020/6572693 |
| 314 | A Unique Isolation of a Lytic Bacteriophage Infected Bacillus anthracis Isolate from Pafuri, South Africa. | Hassim, Ayesha; Lekota, Kgaugelo Edward; van Dyk, David Schalk; Dekker, Edgar Henry; van Heerden, Henriette | 2020 | Microorganisms | https://dx.doi.org/10.3390/microorganisms8060932 |
| 315 | Genomic sequence data and single nucleotide polymorphism genotyping of Bacillus anthracis strains isolated from animal anthrax outbreaks in Northern Cape Province, South Africa. | Lekota, Kgaugelo Edward; Hassim, Ayesha; van Heerden, Henriette | 2020 | Data in brief | https://dx.doi.org/10.1016/j.dib.2019.105040 |
| 316 | Insights from Bacillus anthracis strains isolated from permafrost in the tundra zone of Russia. | Timofeev, Vitalii; Bahtejeva, Irina; Mironova, Raisa; Titareva, Galina; Lev, Igor; Christiany, David; Borzilov, Alexander; Bogun, Alexander; Vergnaud, Gilles | 2019 | PloS one | https://dx.doi.org/10.1371/journal.pone.0209140 |
| 317 | Yeasts and bacteria associated with kocho, an Ethiopian fermented food produced from enset (Ensete ventricosum). | Birmeta, Genet; Bakeeva, Albina; Passoth, Volkmar | 2019 | Antonie van Leeuwenhoek | https://dx.doi.org/10.1007/s10482-018-1192-8 |
| 318 | Drumming-associated anthrax incidents: exposures to low levels of indoor environmental contamination. | Bennett, E; Hall, I M; Pottage, T; Silman, N J; Bennett, A M | 2018 | Epidemiology and infection | https://dx.doi.org/10.1017/S0950268818001085 |
| 319 | Correction: Development and application of a Bacillus anthracis protective antigen domain-1 in-house ELISA for the detection of anti-protective antigen antibodies in cattle in Zambia. | Simbotwe, Manyando; Fujikura, Daisuke; Ohnuma, Miyuki; Omori, Ryosuke; Furuta, Yoshikazu; Muuka, Geoffrey Munkombwe; Hang'ombe, Bernard Mudenda; Higashi, Hideaki | 2019 | PloS one | https://dx.doi.org/10.1371/journal.pone.0211592 |
| 320 | Use of Canonical Single Nucleotide Polymorphism (CanSNPs) to characterize Bacillus anthracis outbreak strains in Zambia between 1990 and 2014. | Fasanella, Antonio; Serrecchia, Luigina; Chiaverini, Alexandra; Garofolo, Giuliano; Muuka, Geoffrey M; Mwambazi, Lucas | 2018 | PeerJ | https://dx.doi.org/10.7717/peerj.5270 |
| 321 | Increasing the Local Relevance of Epidemiological Research: Situated Knowledge of Cattle Disease Among Basongora Pastoralists in Uganda. | Chenais, Erika; Fischer, Klara | 2018 | Frontiers in veterinary science | https://dx.doi.org/10.3389/fvets.2018.00119 |
| 322 | One Health Research in Northern Tanzania - Challenges and Progress. | Ladbury, Georgia; Allan, Kathryn J; Cleaveland, Sarah; Davis, Alicia; de Glanville, William A; Forde, Taya L; Halliday, Jo E B; Haydon, Daniel T; Kibiki, Gibson; Kiwelu, Ireen; Lembo, Tiziana; Maro, Venance; Mmbaga, Blandina T; Ndyetabura, Theonest; Sharp, Jo; Thomas, Kate; Zadoks, Ruth N | 2017 | The East African health research journal | https://dx.doi.org/10.24248/EAHRJ-D-16-00379 |
| 323 | Zebra migration strategies and anthrax in Etosha National Park, Namibia. | Zidon, Royi; Garti, Shimon; Getz, Wayne M; Saltz, David | 2017 | Ecosphere (Washington, D.C) | https://dx.doi.org/10.1002/ecs2.1925 |
| 324 | Black Eschars in the Highlands of Ethiopia. | Perez-Tanoira, Ramon; Cuadros, Juan; Prieto-Perez, Laura | 2016 | The American journal of tropical medicine and hygiene | https://dx.doi.org/10.4269/ajtmh.15-0763 |
| 325 | The efficacy and safety of nine South African medicinal plants in controlling Bacillus anthracis Sterne vaccine strain. | Elisha, Ishaku Leo; Dzoyem, Jean-Paul; Botha, Francien S; Eloff, Jacobus Nicolaas | 2016 | BMC complementary and alternative medicine | https://dx.doi.org/10.1186/s12906-015-0980-1 |
| 326 | Enhanced Immune Response to DNA Vaccine Encoding Bacillus anthracis PA-D4 Protects Mice against Anthrax Spore Challenge. | Kim, Na Young; Chang, Dong Suk; Kim, Yeonsu; Kim, Chang Hwan; Hur, Gyeung Haeng; Yang, Jai Myung; Shin, Sungho | 2015 | PloS one | https://dx.doi.org/10.1371/journal.pone.0139671 |
| 327 | [General procedures in response to suspected attacks with highly contagious and pathogenic agents]. | Richter, Martin; Herzog, Christian | 2015 | Bundesgesundheitsblatt, Gesundheitsforschung, Gesundheitsschutz | https://dx.doi.org/10.1007/s00103-015-2160-3 |
| 328 | Development of a simple method for the rapid identification of organisms causing anthrax by coagglutination test. | Sumithra, T G; Chaturvedi, V K; Gupta, P K; Siju, S J; Susan, C; Bincy, J; Laxmi, U; Sunita, S C; Rai, A K | 2014 | Biologicals : journal of the International Association of Biological Standardization | https://dx.doi.org/10.1016/j.biologicals.2014.07.006 |
| 329 | Identification of peptide sequences as a measure of Anthrax vaccine stability during storage. | Whiting, Gail; Wheeler, Jun X; Rijpkema, Sjoerd | 2014 | Human vaccines & immunotherapeutics | https://dx.doi.org/10.4161/hv.28443 |
| 330 | Development of a simple and rapid method for the specific identification of organism causing anthrax by slide latex agglutination. | Sumithra, T G; Chaturvedi, V K; Gupta, P K; Sunita, S C; Rai, A K; Kutty, M V H; Laxmi, U; Murugan, M S | 2014 | Letters in applied microbiology | https://dx.doi.org/10.1111/lam.12204 |
| 331 | [The diagnostic significance of particular immune-dominating proteins of isogenic variants of Bacillus Anthracis.]. | Barkova, I A; Chervakova, M P; Barkov, A M; Novojenina, A V; Viktorov, D V | 2016 | Klinicheskaia laboratornaia diagnostika | https://dx.doi.org/10.18821/0869-2084-2016-61-12-833-837 |
| 332 | Draft Genome Sequences of Two Bacillus anthracis Strains from Etosha National Park, Namibia. | Valseth, Karoline; Nesbo, Camilla L; Easterday, W Ryan; Turner, Wendy C; Olsen, Jaran S; Stenseth, Nils C; Haverkamp, Thomas H A | 2016 | Genome announcements | https://dx.doi.org/10.1128/genomeA.00861-16 |
| 333 | Applying Science: Opportunities to Inform Disease Management Policy with Cooperative Research within a One Health Framework. | Blackburn, Jason K; Kracalik, Ian T; Fair, Jeanne Marie | 2015 | Frontiers in public health | https://dx.doi.org/10.3389/fpubh.2015.00276 |
| 334 | Draft Genome Sequences of Two South African Bacillus anthracis Strains. | Lekota, Kgaugelo E; Mafofo, Joseph; Madoroba, Evelyn; Rees, Jasper; van Heerden, Henriette; Muchadeyi, Farai C | 2015 | Genome announcements | https://dx.doi.org/10.1128/genomeA.01313-15 |
| 335 | Correction: Bacillus anthracis Diversity and Geographic Potential across Nigeria, Cameroon and Chad: Further Support of a Novel West African Lineage. | Blackburn, Jason K; Odugbo, Moses Ode; Van Ert, Matthew; O'Shea, Bob; Mullins, Jocelyn; Perreten, Vincent; Maho, Angaya; Hugh-Jones, Martin; Hadfield, Ted | 2015 | PLoS neglected tropical diseases | https://dx.doi.org/10.1371/journal.pntd.0004089 |
| 336 | Surveillance and diagnosis of plague and anthrax in Tanzania and Zambia. | Hang'ombe, B M; Ziwa, M; Haule, M; Nakamura, I; Samui, K L; Kaile, D; Mweene, A S; Kilonzo, B S; Lyamuya, E F; Matee, M; Sugimoto, C; Sawa, H; Wren, B W | 2014 | The Onderstepoort journal of veterinary research | https://dx.doi.org/10.4102/ojvr.v81i2.722 |
| 337 | Genome Sequence of a Bacillus anthracis Outbreak Strain from Zambia, 2011. | Ohnishi, Naomi; Maruyama, Fumito; Ogawa, Hirohito; Kachi, Hirokazu; Yamada, Shunsuke; Fujikura, Daisuke; Nakagawa, Ichiro; Hang'ombe, Mudenda B; Thomas, Yuka; Mweene, Aaron S; Higashi, Hideaki | 2014 | Genome announcements | https://dx.doi.org/10.1128/genomeA.00116-14 |
| 338 | <i>Bacillus cereus</i> Biovar Anthracis Causing Anthrax in Sub-Saharan Africa-Chromosomal Monophyly and Broad Geographic Distribution | Antonation, KS; Gr√ºtzmacher, K; Dupke, S; Mabon, P; Zimmermann, F; Lankester, F; Peller, T; Feistner, A; Todd, A; Herbinger, I; de Nys, HM; Muyembe-Tamfun, JJ; Karhemere, S; Wittig, RM; Couacy-Hymann, E; Grunow, R; Calvignac-Spencer, S; Corbett, CR; Klee, SR; Leendertz, FH | 2016 | PLOS NEGLECTED TROPICAL DISEASES | 10.1371/journal.pntd.0004923 |
| 339 | Polyphasic characterization of <i>Bacillus</i> species from anthrax outbreaks in animals from South Africa and Lesotho | Lekota, KE; Hassim, A; Mafofo, J; Rees, J; Muchadeyi, FC; van Heerden, H; Madoroba, E | 2016 | JOURNAL OF INFECTION IN DEVELOPING COUNTRIES | 10.3855/jidc.7798 |
| 340 | Genomic sequence data and single nucleotide polymorphism genotyping of <i>Bacillus anthracis</i> strains isolated from animal anthrax outbreaks in Northern Cape Province, South Africa | Lekota, KE; Hassim, A; van Heerden, H | 2020 | DATA IN BRIEF | 10.1016/j.dib.2019.105040 |
| 341 | Phylogenomic structure of <i>Bacillus anthracis</i> isolates in the Northern Cape Province, South Africa revealed novel single nucleotide polymorphisms | Lekota, KE; Hassim, A; Madoroba, E; Hefer, CA; van Heerden, H | 2020 | INFECTION GENETICS AND EVOLUTION | 10.1016/j.meegid.2019.104146 |
| 342 | Use of Canonical Single Nucleotide Polymorphism (CanSNPs) to characterize <i>Bacillus anthracis</i> outbreak strains in Zambia between 1990 and 2014 | Fasanella, A; Serrecchia, L; Chiaverini, A; Garofolo, G; Muuka, GM; Mwambazi, L | 2018 | PEERJ | 10.7717/peerj.5270 |
| 343 | Whole genome sequencing and identification of <i>Bacillus</i> <i>endophyticus</i> and <i>B-anthracis</i> isolated from anthrax outbreaks in South Africa | Lekota, KE; Bezuidt, OKI; Mafofo, J; Rees, J; Muchadeyi, FC; Madoroba, E; van Heerden, H | 2018 | BMC MICROBIOLOGY | 10.1186/s12866-018-1205-9 |
| 344 | Potential distributions of <i>Bacillus anthracis</i> and <i>Bacillus cereus</i> biovar <i>anthracis</i> causing anthrax in Africa | Romero-Alvarez, D; Peterson, AT; Salzer, JS; Pittiglio, C; Shadomy, S; Traxler, R; Vieira, AR; Bower, WA; Walke, H; Campbell, LP | 2020 | PLOS NEGLECTED TROPICAL DISEASES | 10.1371/journal.pntd.0008131 |
| 345 | Seasonality and Ecological Suitability Modelling for Anthrax (<i>Bacillus anthracis</i>) in Western Africa | Pittiglio, C; Shadomy, S; El Idrissi, A; Soumare, B; Lubroth, J; Makonnen, Y | 2022 | ANIMALS | 10.3390/ani12091146 |
| 346 | <i>Bacillus anthracis</i> Diversity and Geographic Potential across Nigeria, Cameroon and Chad: Further Support of a Novel West African Lineage | Blackburn, JK; Odugbo, MO; Van Ert, M; O'Shea, B; Mullins, J; Perrenten, V; Maho, A; Hugh-Jones, M; Hadfield, T | 2015 | PLOS NEGLECTED TROPICAL DISEASES | 10.1371/journal.pntd.0003931 |
| 347 | Genomic and Phylogenetic Analysis of <i>Bacillus cereus</i> Biovar <i>anthracis</i> Isolated from Archival Bone Samples Reveals Earlier Natural History of the Pathogen | Norris, MH; Zincke, D; Daegling, DJ; Krigbaum, J; Mcgraw, WS; Kirpich, A; Hadfield, TL; Blackburn, JK | 2023 | PATHOGENS | 10.3390/pathogens12081065 |
| 348 | TaqMan Assays for Simultaneous Detection of <i>Bacillus anthracis</i> and <i>Bacillus cereus</i> biovar <i>anthracis</i> | Zincke, D; Norris, MH; Cruz, O; Kurmanov, B; McGraw, WS; Daegling, DJ; Krigbaum, J; Hoang, TTH; Khanipov, K; Golovko, G; Hadfield, T; Blackburn, JK | 2020 | PATHOGENS | 10.3390/pathogens9121074 |
| 349 | A putative exosporium lipoprotein GBAA0190 of <i>Bacillus anthracis</i> as a potential anthrax vaccine candidate | Jeon, JH; Kim, YH; Kim, KA; Kim, YR; Woo, SJ; Choi, YJ; Rhie, GE | 2021 | BMC IMMUNOLOGY | 10.1186/s12865-021-00414-y |
| 350 | Spores and soil from six sides: interdisciplinarity and the environmental biology of anthrax (<i>Bacillus anthracis</i>) | Carlson, CJ; Getz, WM; Kausrud, KL; Cizauskas, CA; Blackburn, JK; Carrillo, FAB; Colwell, R; Easterday, WR; Ganz, HH; Kamath, PL; Okstad, OA; Turner, WC; Kolsto, AB; Stenseth, NC | 2018 | BIOLOGICAL REVIEWS | 10.1111/brv.12420 |
| 351 | Ecological niche modeling as a tool for prediction of the potential geographic distribution of <i>Bacillus anthracis</i> spores in Tanzania | Mwakapeje, ER; Ndimuligo, SA; Mosomtai, G; Ayebare, S; Nyakarahuka, L; Nonga, HE; Mdegela, RH; Skjerve, E | 2019 | INTERNATIONAL JOURNAL OF INFECTIOUS DISEASES | 10.1016/j.ijid.2018.11.367 |
| 352 | Low antibody prevalence against <i>Bacillus cereus</i> biovar <i>anthracis</i> in Tai National Park, Cote d'Ivoire, indicates high rate of lethal infections in wildlife | Zimmermann, F; K√∂hler, SM; Nowak, K; Dupke, S; Barduhn, A; D√ºx, A; Lang, A; De Nys, HM; Gogarten, JF; Grunow, R; Couacy-Hymann, E; Wittig, RM; Klee, SR; Leendertz, FH | 2017 | PLOS NEGLECTED TROPICAL DISEASES | 10.1371/journal.pntd.0005960 |
| 353 | Population genomics of <i>Bacillus</i> <i>anthracis</i> from an anthrax hyperendemic area reveals transmission processes across spatial scales and unexpected within-host diversity | Forde, TL; Dennis, TPW; Aminu, OR; Harvey, WT; Hassim, A; Kiwelu, I; Medvecky, M; Mshanga, D; Van Heerden, H; Vogel, A; Zadoks, RN; Mmbaga, BT; Lembo, T; Biek, R | 2022 | MICROBIAL GENOMICS | 10.1099/mgen.0.000759 |
| 354 | Detection of <i>Bacillus anthracis</i> in animal tissues using InBios active anthrax detect rapid test lateral flow immunoassay | Kolton, CB; Marston, CK; Stoddard, RA; Cossaboom, C; Salzer, JS; Kozel, TR; Gates-Hollingsworth, MA; Cleveland, CA; Thompson, AT; Dalton, MF; Yabsley, MJ; Hoffmaster, AR | 2019 | LETTERS IN APPLIED MICROBIOLOGY | 10.1111/lam.13134 |
| 355 | <i>Bacillus cereus</i> from the environment is genetically related to the highly pathogenic <i>B</i>. <i>cereus</i> in Zambia | Ogawa, H; Ohnuma, M; Squarre, D; Mweene, AS; Ezaki, T; Fujikura, D; Ohnishi, N; Thomas, Y; Hang'Ombe, BM; Higashi, H | 2015 | JOURNAL OF VETERINARY MEDICAL SCIENCE | 10.1292/jvms.15-0059 |
| 356 | A Unique Isolation of a Lytic Bacteriophage Infected<i>Bacillus anthracis</i>Isolate from Pafuri, South Africa | Hassim, A; Lekota, KE; van Dyk, DS; Dekker, EH; van Heerden, H | 2020 | MICROORGANISMS | 10.3390/microorganisms8060932 |
| 357 | No hints for abundance of <i>Bacillus anthracis</i> and <i>Burkholderia</i> <i>pseudomallei</i> in 100 environmental samples from Cameroon | Frickmann, H; Poppert, S | 2021 | EUROPEAN JOURNAL OF MICROBIOLOGY AND IMMUNOLOGY | 10.1556/1886.2021.00014 |
| 358 | Serological evidence for human exposure to <i>Bacillus cereus</i> biovar <i>anthracis</i> in the villages around Tai National Park, Cote d'Ivoire | Dupke, S; Schubert, G; Beudj√©, F; Barduhn, A; Pauly, M; Couacy-Hymann, E; Grunow, R; Akoua-Koffi, C; Leendertz, FH; Klee, SR | 2020 | PLOS NEGLECTED TROPICAL DISEASES | 10.1371/journal.pntd.0008292 |
| 359 | Quantitative Determination of Lethal Toxin Proteins in Culture Supernatant of Human Live Anthrax Vaccine <i>Bacillus anthracis</i> A16R | Zai, XD; Zhang, J; Liu, J; Li, LL; Yin, Y; Fu, L; Xu, JJ; Chen, W | 2016 | TOXINS | 10.3390/toxins8030056 |
| 360 | A novel live attenuated anthrax spore vaccine based on an acapsular <i>Bacillus</i> <i>anthracis</i> Sterne strain with mutations in the <i>htrA</i>, <i>lef</i> and <i>cya</i> genes | Chitlaru, T; Israeli, M; Rotem, S; Elia, U; Bar -Haim, E; Ehrlich, S; Cohen, O; Shafferman, A | 2017 | VACCINE | 10.1016/j.vaccine.2017.03.033 |
| 361 | The efficacy and safety of nine South African medicinal plants in controlling <i>Bacillus anthracis</i> Sterne vaccine strain | Elisha, IL; Dzoyem, JP; Botha, FS; Eloff, JN | 2016 | BMC COMPLEMENTARY AND ALTERNATIVE MEDICINE | 10.1186/s12906-015-0980-1 |
| 362 | Loss of Bacitracin Resistance Due to a Large Genomic Deletion among <i>Bacillus anthracis</i> Strains | Furuta, Y; Harima, H; Ito, E; Maruyama, F; Ohnishi, N; Osaki, K; Ogawa, H; Squarre, D; Hang'ombe, BM; Higashi, H | 2018 | MSYSTEMS | 10.1128/mSystems.00182-18 |
| 363 | Seasonal variation in foraging behaviour of plains zebra (<i>Equus quagga</i>) may alter contact with the anthrax bacterium (<i>Bacillus anthracis</i>) | Havarua, Z; Turner, WC; Mfune, JKE | 2014 | CANADIAN JOURNAL OF ZOOLOGY | 10.1139/cjz-2013-0186 |
| 364 | Nucleotide polymorphism assay for the identification of west African group <i>Bacillus anthracis:</i> a lineage lacking anthrose | Zincke, D; Norris, MH; Kurmanov, B; Hadfield, TL; Blackburn, JK | 2020 | BMC MICROBIOLOGY | 10.1186/s12866-019-1693-2 |
| 365 | Genetic Characterization of <i>Bacillus anthracis</i> 17 JB strain | Seyed-Mohamadi, S; Bidhendi, SM; Tadayon, K; Ghaderi, R | 2015 | IRANIAN JOURNAL OF MICROBIOLOGY |  |
| 366 | Molecular characterization of <i>B</i>. <i>anthracis</i> isolates from the anthrax outbreak among cattle in Karnataka, India | Roonie, A; Majumder, S; Kingston, JJ; Parida, M | 2020 | BMC MICROBIOLOGY | 10.1186/s12866-020-01917-1 |
| 367 | Common garlic (<i>Allium sativum</i> L.) has potent Anti<i>-Bacillus anthracis</i> activity | Kaur, R; Tiwari, A; Manish, M; Maurya, IK; Bhatnagar, R; Singh, S | 2021 | JOURNAL OF ETHNOPHARMACOLOGY | 10.1016/j.jep.2020.113230 |
| 368 | Whole genome sequencing of<i> Bacillus</i><i> anthracis</i> isolated from animal in the 1960s, Brazil, belonging to the South America subclade | de Andrade, TS; Camargo, CH; Campos, KR; Reis, AD; Santos, MBD; Zanelatto, VN; Takagi, EH; Sacchi, CT | 2023 | COMPARATIVE IMMUNOLOGY MICROBIOLOGY AND INFECTIOUS DISEASES | 10.1016/j.cimid.2023.102027 |
| 369 | Protection of rhesus macaques against inhalational anthrax with a <i>Bacillus anthracis</i> capsule conjugate vaccine | Chabot, DJ; Ribot, WJ; Joyce, J; Cook, J; Hepler, R; Nahas, D; Chua, J; Friedlander, AM | 2016 | VACCINE | 10.1016/j.vaccine.2016.06.031 |
| 370 | The potential distribution of <i>Bacillus anthracis</i> suitability across Uganda using INLA | Ndolo, VA; Redding, D; Deka, MA; Salzer, JS; Vieira, AR; Onyuth, H; Ocaido, M; Tweyongyere, R; Azuba, R; Monje, F; Ario, AR; Kabwama, S; Kisaakye, E; Bulage, L; Kwesiga, B; Ntono, V; Harris, J; Wood, JLN; Conlan, AJK | 2022 | SCIENTIFIC REPORTS | 10.1038/s41598-022-24281-8 |
| 371 | Draft Genome Sequences of Two South African <i>Bacillus anthracis</i> Strains | Lekota, KE; Mafofo, J; Madoroba, E; Rees, J; van Heerden, H; Muchadeyi, FC | 2015 | GENOME ANNOUNCEMENTS | 10.1128/genomeA.01313-15 |
| 372 | Efficacy assessment of a triple anthrax chimeric antigen as a vaccine candidate in guinea pigs: challenge test with <i>Bacillus anthracis</i> 17 JB strain spores | Abdous, M; Hasannia, S; Salmanian, AH; Arab, SS | 2021 | IMMUNOPHARMACOLOGY AND IMMUNOTOXICOLOGY | 10.1080/08923973.2021.1945087 |
| 373 | Draft Genome Sequences of Two <i>Bacillus anthracis</i> Strains from Etosha National Park, Namibia | Valseth, K; Nesbo, CL; Easterday, WR; Turner, WC; Olsen, JS; Stenseth, NC; Haverkamp, THA | 2016 | GENOME ANNOUNCEMENTS | 10.1128/genomeA.00861-16 |
| 374 | Occurrence, Antibiotic Resistance, Virulence Factors, and Genetic Diversity of <i>Bacillus</i> spp. from Public Hospital Environments in South Africa | Mbhele, ZN; Shobo, CO; Amoako, DG; Zishiri, OT; Bester, LA | 2021 | MICROBIAL DRUG RESISTANCE | 10.1089/mdr.2020.0543 |
| 375 | A serological survey of<i>Bacillus anthracis</i>reveals widespread exposure to the pathogen in free-range and captive lions in Zimbabwe | Mukarati, NL; Ndumnego, OC; Ochai, SO; Jauro, S; Loveridge, A; van Heerden, H; Matope, G; Caron, A; Hanyire, TG; de Garine-Wichatitsky, M; Pfukenyi, DM | 2021 | TRANSBOUNDARY AND EMERGING DISEASES | 10.1111/tbed.13842 |
| 376 | Interactions between <i>Bacillus anthracis</i> and Plants May Promote Anthrax Transmission | Ganz, HH; Turner, WC; Brodie, EL; Kusters, M; Shi, Y; Sibanda, H; Torok, T; Getz, WM | 2014 | PLOS NEGLECTED TROPICAL DISEASES | 10.1371/journal.pntd.0002903 |
| 377 | Genotyping and phylogenetic placement of <i>Bacillus anthracis</i> isolates from Finland, a country with rare anthrax cases | Lienemann, T; Beyer, W; Pelkola, K; Rossow, H; Rehn, A; Antwerpen, M; Grass, G | 2018 | BMC MICROBIOLOGY | 10.1186/s12866-018-1250-4 |
| 378 | Passive protection against anthrax in mice with plasma derived from horses hyper-immunized against <i>Bacillus anthracis</i> Sterne strain | Caldwell, M; Hathcock, T; Brock, KV | 2017 | PEERJ | 10.7717/peerj.3907 |
| 379 | The global distribution of <i>Bacillus anthracis</i> and associated anthrax risk to humans, livestock and wildlife | Carlson, CJ; Kracalik, IT; Ross, N; Alexander, KA; Hugh-Jones, ME; Fegan, M; Elkin, BT; Epp, T; Shury, TK; Zhang, WY; Bagirova, M; Getz, WM; Blackburn, JK | 2019 | NATURE MICROBIOLOGY | 10.1038/s41564-019-0435-4 |
| 380 | Protection of farm goats by combinations of recombinant peptides and formalin inactivated spores from a lethal <i>Bacillus anthracis</i> challenge under field conditions | Koehler, SM; Buyuk, F; Celebi, O; Demiraslan, H; Doganay, M; Sahin, M; Moehring, J; Ndumnego, OC; Otlu, S; van Heerden, H; Beyer, W | 2017 | BMC VETERINARY RESEARCH | 10.1186/s12917-017-1140-2 |
| 381 | Identifying Edaphic Factors and Normalized Difference Vegetation Index Metrics Driving Wildlife Mortality From Anthrax in Kenya's Wildlife Areas | Obanda, V; Otieno, VA; Kingori, EM; Ndeereh, D; Lwande, OW; Chiyo, PI | 2021 | FRONTIERS IN ECOLOGY AND EVOLUTION | 10.3389/fevo.2021.643334 |
| 382 | Analysis of a newly discovered antigen of <i>Bacillus cereus</i> biovar <i>anthracis</i> for its suitability in specific serological antibody testing | Dupke, S; Barduhn, A; Franz, T; Leendertz, FH; Couacy-Hymann, E; Grunow, R; Klee, SR | 2019 | JOURNAL OF APPLIED MICROBIOLOGY | 10.1111/jam.14114 |
| 383 | <i>Bacillus anthracis</i> Evolution: Taking Advantage of the Topology of the Phylogenetic Tree and Human History to Propose Dating Points | Vergnaud, G | 2020 | ERCIYES MEDICAL JOURNAL | 10.14744/etd.2020.64920 |
| 384 | Environmental drivers of biseasonal anthrax outbreak dynamics in two multihost savanna systems | Huang, YH; Kausrud, K; Hassim, A; Ochai, SO; van Schalkwyk, OL; Dekker, EH; Buyantuev, A; Cloete, CC; Kilian, JW; Mfune, JKE; Kamath, PL; van Heerden, H; Turner, WC | 2022 | ECOLOGICAL MONOGRAPHS | 10.1002/ecm.1526 |
| 385 | Alternative pre-approved and novel therapies for the treatment of anthrax | Head, BM; Rubinstein, E; Meyers, AFA | 2016 | BMC INFECTIOUS DISEASES | 10.1186/s12879-016-1951-y |
| 386 | Immunological Evidence of Variation in Exposure and Immune Response to <i>Bacillus anthracis</i> in Herbivores of Kruger and Etosha National Parks | Ochai, SO; Crafford, JE; Hassim, A; Byaruhanga, C; Huang, YH; Hartmann, A; Dekker, EH; van Schalkwyk, OL; Kamath, PL; Turner, WC; van Heerden, H | 2022 | FRONTIERS IN IMMUNOLOGY | 10.3389/fimmu.2022.814031 |
| 387 | Capsules, Toxins and AtxA as Virulence Factors of Emerging <i>Bacillus cereus</i> Biovar <i>anthracis</i> | Br√©zillon, C; Haustant, M; Dupke, S; Corre, JP; Lander, A; Franz, T; Monot, M; Couture-Tosi, E; Jouvion, G; Leendertz, FH; Grunow, R; Mock, ME; Klee, SR; Goossens, PL | 2015 | PLOS NEGLECTED TROPICAL DISEASES | 10.1371/journal.pntd.0003455 |
| 388 | Blowflies as vectors of <i>Bacillus anthracis</i> in the Kruger National Park | Basson, L; Hassim, A; Dekker, A; Gilbert, A; Beyer, W; Rossouw, J; van Heerden, H | 2018 | KOEDOE | 10.4102/koedoe.v60i1.1468 |
| 389 | Direct Regulons of AtxA, the Master Virulence Regulator of <i>Bacillus anthracis</i> | Furuta, Y; Cheng, C; Zorigt, T; Paudel, A; Izumi, S; Tsujinouchi, M; Shimizu, T; Meijer, WG; Higashi, H | 2021 | MSYSTEMS | 10.1128/mSystems.00291-21 |
| 390 | Ungulate use of locally infectious zones in a re-emerging anthrax risk area | Walker, MA; Uribasterra, M; Asher, V; Ponciano, JM; Getz, WM; Ryan, SJ; Blackburn, JK | 2020 | ROYAL SOCIETY OPEN SCIENCE | 10.1098/rsos.200246 |
| 391 | A Whole-Genome-Based Gene-by-Gene Typing System for Standardized High-Resolution Strain Typing of <i>Bacillus anthracis</i> | Abdel-Glil, MY; Chiaverini, A; Garofolo, G; Fasanella, A; Parisi, A; Harmsen, D; Jolley, KA; Elschner, MC; Tomaso, H; Linde, J; Galante, D | 2021 | JOURNAL OF CLINICAL MICROBIOLOGY | 10.1128/JCM.02889-20 |
| 392 | Vaccines against anthrax based on recombinant protective antigen: problems and solutions | Kondakova, OA; Nikitin, NA; Evtushenko, EA; Ryabchevskaya, EM; Atabekov, JG; Karpova, OV | 2019 | EXPERT REVIEW OF VACCINES | 10.1080/14760584.2019.1643242 |
| 393 | COMPLIANCE OF ANTHRAX RECOMBINANT VACCINE PROTOTYPE WITH THE REQUIREMENTS TO IMMUNE-BIOLOGICAL PREPARATIONS | Mikshis, NI; Semakova, AP; Popova, PY; Kudryavtseva, OM; Bugorkova, SA; Komissarov, AV; Germanchuk, VG; Popov, YA | 2018 | INFEKTSIYA I IMMUNITET | 10.15789/2220-7619-2018-3-388-392 |
| 394 | A Novel Multiplex PCR Discriminates <i>Bacillus anthracis</i> and Its Genetically Related Strains from Other <i>Bacillus cereus</i> Group Species | Ogawa, H; Fujikura, D; Ohnuma, M; Ohnishi, N; Hang'ombe, BM; Mimuro, H; Ezaki, T; Mweene, AS; Higashi, H | 2015 | PLOS ONE | 10.1371/journal.pone.0122004 |
| 395 | Development and application of a <i>Bacillus anthracis</i> protective antigen domain-1 in-house ELISA for the detection of antiprotective antigen antibodies in cattle in Zambia | Simbotwe, M; Fujikura, D; Ohnuma, M; Omori, R; Furuta, Y; Muuka, GM; Hang'ombe, BM; Higashi, H | 2018 | PLOS ONE | 10.1371/journal.pone.0205986 |
| 396 | Novel Giant Siphovirus from <i>Bacillus anthracis</i> Features Unusual Genome Characteristics | Ganz, HH; Law, C; Schmuki, M; Eichenseher, F; Calendar, R; Loessner, MJ; Getz, WM; Korlach, J; Beyer, W; Klumpp, J | 2014 | PLOS ONE | 10.1371/journal.pone.0085972 |
| 397 | Convergent evolution of diverse <i>Bacillus anthracis</i> outbreak strains toward altered surface oligosaccharides that modulate anthrax pathogenesis | Norris, MH; Kirpich, A; Bluhm, AP; Zincke, D; Hadfield, T; Ponciano, JM; Blackburn, JK | 2020 | PLOS BIOLOGY | 10.1371/journal.pbio.3001052 |
| 398 | Decontamination of <i>Bacillus anthracis</i> Spores at Subzero Temperatures by Complete Submersion | Laing, C; Janzen, T; Blinov, V; Volchek, K; Goji, N; Thomas, M; Telfer, M; Rohonczy, E; Amoako, KK | 2021 | APPLIED BIOSAFETY | 10.1089/apb.20.0067 |
| 399 | The roles of antimicrobial resistance, phage diversity, isolation source and selection in shaping the genomic architecture of <i>Bacillus anthracis</i> | Bruce, SA; Huang, YH; Kamath, PL; van Heerden, H; Turner, WC | 2021 | MICROBIAL GENOMICS | 10.1099/mgen.0.000616 |
| 400 | The worldwide distribution of genetically and phylogenetically diverse <i>Bacillus cereus</i> isolates harbouring <i>Bacillus anthracis</i>-like plasmids | Kaminska, PS; Yernazarova, A; Drewnowska, JM; Zambrowski, G; Swiecicka, I | 2015 | ENVIRONMENTAL MICROBIOLOGY REPORTS | 10.1111/1758-2229.12305 |
| 401 | Anthrax prevention through vaccine and post-exposure therapy | Manish, M; Verma, S; Kandari, D; Kulshreshtha, P; Singh, S; Bhatnagar, R | 2020 | EXPERT OPINION ON BIOLOGICAL THERAPY | 10.1080/14712598.2020.1801626 |
| 402 | Pathological findings in African buffaloes (<i>Syncerus caffer</i>) in South Africa | Woodburn, DB; Steyl, J; du Plessis, EC; Last, RD; Reininghaus, B; Mitchell, EP | 2021 | JOURNAL OF THE SOUTH AFRICAN VETERINARY ASSOCIATION | 10.4102/jsava.v92i0.2117 |
| 403 | Modeling of Anthrax Disease via Efficient Computing Techniques | Raza, A; Baleanu, D; Yousaf, M; Akhter, N; Mahmood, SK; Rafiq, M | 2022 | INTELLIGENT AUTOMATION AND SOFT COMPUTING | 10.32604/iasc.2022.022643 |
| 404 | Animal Models for the Pathogenesis, Treatment, and Prevention of Infection by <i>Bacillus anthracis</i> | Welkos, S; Bozue, J; Twenhafel, N; Cote, C | 2015 | MICROBIOLOGY SPECTRUM | 10.1128/microbiolspec.TBS-0001-2012 |
| 405 | Can scavengers save zebras from anthrax? A modeling study | Mackey, C; Kribs, C | 2021 | INFECTIOUS DISEASE MODELLING | 10.1016/j.idm.2020.10.016 |
| 406 | Spatio-temporal epidemiology of anthrax in <i>Hippopotamus amphibious</i> in Queen Elizabeth Protected Area, Uganda | Driciru, M; Rwego, IB; Asiimwe, B; Travis, DA; Alvarez, J; VanderWaal, K; Pelican, K | 2018 | PLOS ONE | 10.1371/journal.pone.0206922 |
| 407 | Interrogation of<i> Bacillus</i><i> anthracis</i> SrtA active site loop forming open/close lid conformations through extensive MD simulations for understanding binding selectivity of SrtA inhibitors | Selvaraj, C; Selvaraj, G; Ismail, RM; Vijayakumar, R; Baazeem, A; Wei, DQ; Singh, SK | 2021 | SAUDI JOURNAL OF BIOLOGICAL SCIENCES | 10.1016/j.sjbs.2021.05.009 |
| 408 | Assessing disease risk perceptions of wild meat in savanna borderland settlements in Kenya and Tanzania | Patel, EH; Martin, A; Funk, SM; Yongo, M; Floros, C; Thomson, J; Fa, JE | 2023 | FRONTIERS IN ECOLOGY AND EVOLUTION | 10.3389/fevo.2023.1033336 |
| 409 | Toxin-Independent Virulence of <i>Bacillus anthracis</i> in Rabbits | Levy, H; Glinert, I; Weiss, S; Sittner, A; Schlomovitz, J; Altboum, Z; Kobiler, D | 2014 | PLOS ONE | 10.1371/journal.pone.0084947 |
| 410 | Dynamical analysis and control strategies in modeling anthrax | Mushayabasa, S; Marijani, T; Masocha, M | 2017 | COMPUTATIONAL & APPLIED MATHEMATICS | 10.1007/s40314-015-0297-1 |
| 411 | Welder's Anthrax: A Review of an Occupational Disease | de Perio, MA; Hendricks, KA; Dowell, CH; Bower, WA; Burton, NC; Dawson, P; Schrodt, CA; Salzer, JS; Marston, CK; Feldmann, K; Hoffmaster, AR; Antonini, JM | 2022 | PATHOGENS | 10.3390/pathogens11040402 |
| 412 | Next-Generation <i>Bacillus anthracis</i> Live Attenuated Spore Vaccine Based on the <i>htrA</i><SUP>-</SUP> (High Temperature Requirement A) Sterne Strain | Chitlaru, T; Israeli, M; Bar-Haim, E; Elia, U; Rotem, S; Ehrlich, S; Cohen, O; Shafferman, A | 2016 | SCIENTIFIC REPORTS | 10.1038/srep18908 |
| 413 | Evaluation of standardized sample collection, packaging, and decontamination procedures to assess cross-contamination potential during <i>Bacillus anthracis</i> incident response operations | Calfee, MW; Tufts, J; Meyer, K; McConkey, K; Mickelsen, L; Rose, L; Dowell, C; Delaney, L; Weber, A; Morse, S; Chaitram, J; Gray, M | 2016 | JOURNAL OF OCCUPATIONAL AND ENVIRONMENTAL HYGIENE | 10.1080/15459624.2016.1200725 |
| 414 | Complete Genome Sequence of an Environmental <i>Bacillus cereus</i> Isolate Belonging to the <i>Bacillus anthracis</i> Clade | Irenge, LM; Bearzatto, B; Ambroise, J; Gala, JL | 2020 | MICROBIOLOGY RESOURCE ANNOUNCEMENTS | 10.1128/MRA.00917-20 |
| 415 | Development of a Sterne-Based Complement Fixation Test to Monitor the Humoral Response Induced by Anthrax Vaccines | Adone, R; Sali, M; Francia, M; Iatarola, M; Donatiello, A; Fasanella, A | 2016 | FRONTIERS IN MICROBIOLOGY | 10.3389/fmicb.2016.00019 |
| 416 | Comparative analysis of the immunologic response induced by the Sterne 34F2 live spore <i>Bacillus</i> <i>anthracis</i> vaccine in a ruminant model | Ndumnego, OC; K√∂hler, SM; Crafford, J; van Heerden, H; Beyer, W | 2016 | VETERINARY IMMUNOLOGY AND IMMUNOPATHOLOGY | 10.1016/j.vetimm.2016.06.005 |
| 417 | Investigation of human anthrax outbreak in Koraput district of Odisha, India | Parai, D; Pattnaik, M; Choudhary, HR; Padhi, AK; Pattnaik, S; Jena, S; Sahoo, SK; Rout, UK; Padhi, A; Sahoo, N; Biswal, S; Padhi, SK; Pati, S; Bhattacharya, D | 2023 | TRAVEL MEDICINE AND INFECTIOUS DISEASE | 10.1016/j.tmaid.2023.102659 |
| 418 | Maximization of Livestock Anthrax Vaccination Coverage in Bangladesh: An Alternative Approach | Sarker, MSA; El Zowalaty, ME; Shahid, MAH; Sarker, MA; Rahman, MB; J√§rhult, JD; Nazir, KHMNH | 2020 | VACCINES | 10.3390/vaccines8030435 |
| 419 | Hippopotamus movements structure the spatiotemporal dynamics of an active anthrax outbreak | Stears, K; Schmitt, MH; Turner, WC; McCauley, DJ; Muse, EA; Kiwango, H; Mathayo, D; Mutayoba, BM | 2021 | ECOSPHERE | 10.1002/ecs2.3540 |
| 420 | Pathology of wild-type and toxin-independent <i>Bacillus anthracis</i> meningitis in rabbits | Sittner, A; Bar-David, E; Glinert, I; Ben-Shmuel, A; Weiss, S; Schlomovitz, J; Kobiler, D; Levy, H | 2017 | PLOS ONE | 10.1371/journal.pone.0186613 |
| 421 | Coalescence modeling of intrainfection <i>Bacillus anthracis</i> populations allows estimation of infection parameters in wild populations | Easterday, WR; Ponciano, JM; Gomez, JP; van Ert, MN; Hadfield, T; Bagamian, K; Blackburn, JK; Stenseth, NC; Turner, WC | 2020 | PROCEEDINGS OF THE NATIONAL ACADEMY OF SCIENCES OF THE UNITED STATES OF AMERICA | 10.1073/pnas.1920790117 |
| 422 | OPTIMAL CONTROL APPLIED IN AN ANTHRAX EPIZOOTIC MODEL | Pantha, B; Day, J; Lenhart, S | 2016 | JOURNAL OF BIOLOGICAL SYSTEMS | 10.1142/S021833901650025X |
| 423 | Using Telemetry Data to Refine Endpoints for New Zealand White Rabbits Challenged with <i>Bacillus anthracis</i> | Dawson, DG; Bower, KA; Burnette, CN; Holt, RK; Swearengen, JR; Dabisch, PA; Scorpio, A | 2017 | JOURNAL OF THE AMERICAN ASSOCIATION FOR LABORATORY ANIMAL SCIENCE |  |
| 424 | Aerosolized Intratracheal Inoculation of Recombinant Protective Antigen (rPA) Vaccine Provides Protection Against Inhalational Anthrax in B10.D2-Hc<SUP>0</SUP> Mice | Song, XL; Zhang, W; Zhai, LA; Guo, JS; Zhao, Y; Zhang, LL; Hu, LF; Xiong, XL; Zhou, DS; Lv, M; Yang, WH | 2022 | FRONTIERS IN IMMUNOLOGY | 10.3389/fimmu.2022.819089 |
| 425 | The Biosafety Research Road Map: The Search for Evidence to Support Practices in the Laboratory-<i>Bacillus anthracis</i> and <i>Brucella melitensis</i> | Blacksell, SD; Dhawan, S; Kusumoto, M; Le, KK; Summermatter, K; O'Keefe, J; Kozlovac, J; Almuhairi, SS; Sendow, I; Scheel, CM; Ahumibe, A; Masuku, ZM; Bennett, AM; Kojima, K; Harper, DR; Hamilton, K | 2023 | APPLIED BIOSAFETY | 10.1089/apb.2022.0042 |
| 426 | Immunogenicity of Non-Living Anthrax Vaccine Candidates in Cattle and Protective Efficacy of Immune Sera in A/J Mouse Model Compared to the Sterne Live Spore Vaccine | Jauro, S; Ndumnego, OC; Ellis, C; Buys, A; Beyer, W; van Heerden, H | 2020 | PATHOGENS | 10.3390/pathogens9070557 |
| 427 | Prediction of human-<i>Bacillus anthracis</i> protein-protein interactions using multi-layer neural network | Ahmed, I; Witbooi, P; Christoffels, A | 2018 | BIOINFORMATICS | 10.1093/bioinformatics/bty504 |
| 428 | Guerrilla healthcare innovation: creative resilience in Zimbabwe's <i>chimurenga</i>, 1971-1980 | Mavhunga, CC | 2015 | HISTORY AND TECHNOLOGY | 10.1080/07341512.2015.1129205 |
| 429 | Protein- and DNA-based anthrax toxin vaccines confer protection in guinea pigs against inhalational challenge with <i>Bacillus cereus</i> G9241 | Palmer, J; Bell, M; Darko, C; Barnewall, R; Keane-Myers, A | 2014 | PATHOGENS AND DISEASE | 10.1111/2049-632X.12204 |
| 430 | Infection with a Nonencapsulated <i>Bacillus anthracis</i> Strain in RabbitsThe Role of Bacterial Adhesion and the Potential for a Safe Live Attenuated Vaccine | Glinert, I; Weiss, S; Sittner, A; Bar-David, E; Ben-Shmuel, A; Schlomovitz, J; Kobiler, D; Levy, H | 2018 | TOXINS | 10.3390/toxins10120506 |
| 431 | CHARACTERISTICS AS WELL AS PAST AND PRESENT POSSIBILITIES OF USING MICROORGANISMS LISTED ON THE CDC LIST A OF BIOLOGICAL AGENTS IN BIOTERRORIST ATTACKS | Weiner, M; Tarasiuk, K | 2019 | HEALTH PROBLEMS OF CIVILIZATION | 10.5114/hpc.2018.78910 |
| 432 | Decontamination Efficacy and Skin Toxicity of Two Decontaminants against <i>Bacillus anthracis</i> | Stratilo, CW; Crichton, MKF; Sawyer, TW | 2015 | PLOS ONE | 10.1371/journal.pone.0138491 |
| 433 | Taxonomy of the genus <i>Anthrax</i> Scopoli (Diptera: Bombyliidae) in Egypt | El-Hawagry, MS | 2021 | JOURNAL OF NATURAL HISTORY | 10.1080/00222933.2021.1914237 |
| 434 | DESCRIPTIVE EPIDEMIOLOGY OF DETECTED ANTHRAX OUTBREAKS IN WILD WOOD BISON (<i>BISON BISON ATHABASCAE</i>) IN NORTHERN CANADA, 1962-2008 | Salb, A; Stephen, C; Ribble, C; Elkin, B | 2014 | JOURNAL OF WILDLIFE DISEASES | 10.7589/2013-04-095 |
| 435 | Immunogenicity and Protective Efficacy of a Non-Living Anthrax Vaccine versus a Live Spore Vaccine with Simultaneous Penicillin-G Treatment in Cattle | Jauro, S; Ndumnego, OC; Ellis, C; Buys, A; Beyer, W; van Heerden, H | 2020 | VACCINES | 10.3390/vaccines8040595 |
| 436 | Acoustofluidic device for acoustic capture of <i>Bacillus anthracis</i> spore analogues at low concentrationa) | Plazonic, F; Fisher, A; Carugo, D; Hill, M; Glynne-Jones, P | 2021 | JOURNAL OF THE ACOUSTICAL SOCIETY OF AMERICA | 10.1121/10.0005278 |
| 437 | Biodegradation of carbofuran in soils within Nzoia River Basin, Kenya | Onunga, DO; Kowino, IO; Ngigi, AN; Osogo, A; Orata, F; Getenga, ZM; Were, H | 2015 | JOURNAL OF ENVIRONMENTAL SCIENCE AND HEALTH PART B-PESTICIDES FOOD CONTAMINANTS AND AGRICULTURAL WASTES | 10.1080/03601234.2015.1011965 |
| 438 | Exposure to <i>Bacillus anthracis</i> Capsule Results in Suppression of Human Monocyte-Derived Dendritic Cells | Jelacic, TM; Chabot, DJ; Bozue, JA; Tobery, SA; West, MW; Moody, K; Yang, D; Oppenheim, JJ; Friedlander, AM | 2014 | INFECTION AND IMMUNITY | 10.1128/IAI.01857-14 |
| 439 | Effect of reduced dose schedules and intramuscular injection of anthrax vaccine adsorbed on immunological response and safety profile: A randomized trial | Wright, JG; Plikaytis, BD; Rose, CE; Parker, SD; Babcock, J; Keitel, W; El Sahly, H; Poland, GA; Jacobson, RM; Keyserling, HL; Semenova, VA; Li, H; Schiffer, J; Dababneh, H; Martin, SK; Martin, SW; Marano, N; Messonnier, NE; Quinn, CP | 2014 | VACCINE | 10.1016/j.vaccine.2013.10.039 |
| 440 | MODEL FOR TRANSMISSION AND OPTIMAL CONTROL OF ANTHRAX INVOLVING HUMAN AND ANIMAL POPULATION | Zewdie, AD; Gakkhar, SUNITA; Gupta, SK | 2022 | JOURNAL OF BIOLOGICAL SYSTEMS | 10.1142/S0218339022500218 |
| 441 | Structural and immunochemical relatedness suggests a conserved pathogenicity motif for secondary cell wall polysaccharides in <i>Bacillus anthracis</i> and infection-associated <i>Bacillus cereus</i> | Kamal, N; Ganguly, J; Saile, E; Klee, SR; Hoffmaster, A; Carlson, RW; Forsberg, LS; Kannenberg, EL; Quinn, CP | 2017 | PLOS ONE | 10.1371/journal.pone.0183115 |
| 442 | Characteristics and phylogeny of <i>Bacillus cereus</i> strains isolated from Maari, a traditional West African food condiment | Thorsen, L; Kando, CK; Sawadogo, H; Larsen, N; Diawara, B; Ou√©draogo, GA; Hendriksen, NB; Jespersen, L | 2015 | INTERNATIONAL JOURNAL OF FOOD MICROBIOLOGY | 10.1016/j.ijfoodmicro.2014.11.026 |
| 443 | Evaluation of the AV7909 Anthrax Vaccine Toxicity in Sprague Dawley Rats Following Three Intramuscular Administrations | Rao, VV; Godin, CS; Lacy, MJ; Inglefield, JR; Park, S; Blauth, B; Reece, JJ; Ionin, B; Savransky, V | 2021 | INTERNATIONAL JOURNAL OF TOXICOLOGY | 10.1177/10915818211031239 |
| 444 | Progress toward the Development of a NEAT Protein Vaccine for Anthrax Disease | Balderas, MA; Nguyen, CTQ; Terwilliger, A; Keitel, WA; Iniguez, A; Torres, R; Palacios, F; Goulding, CW; Maresso, AW | 2016 | INFECTION AND IMMUNITY | 10.1128/IAI.00755-16 |
| 445 | An Unusual Kind of Town: Cattle Disease, Zoonosis, and Public Health in Colonial Salaga (Northern Ghana) | Akyeampong, E | 2020 | INTERNATIONAL JOURNAL OF AFRICAN HISTORICAL STUDIES |  |
| 446 | Evaluation of liposomal ciprofloxacin formulations in a murine model of anthrax | Stratilo, CW; Jager, S; Crichton, M; Blanchard, JD | 2020 | PLOS ONE | 10.1371/journal.pone.0228162 |
| 447 | Environmental niche modeling for some species of the genus <i>Anthrax</i> Scopoli (Diptera: Bombyliidae) in Egypt, with special notes on St. Catherine protected area as a suitable habitat | Nasser, M; El-Hawagry, M; Okely, M | 2019 | JOURNAL OF INSECT CONSERVATION | 10.1007/s10841-019-00174-6 |
| 448 | Carnivory in the common hippopotamus <i>Hippopotamus amphibius</i>: implications for the ecology and epidemiology of anthrax in African landscapes | Dudley, JP; Hang'ombe, BM; Leendertz, FH; Dorward, LJ; de Castro, J; Subalusky, AL; Clauss, M | 2016 | MAMMAL REVIEW | 10.1111/mam.12056 |
| 449 | Label-free electrochemical sensor based on spore-imprinted polymer for <i>Bacillus cereus</i> spore detection | Lahcen, AA; Arduini, F; Lista, F; Amine, A | 2018 | SENSORS AND ACTUATORS B-CHEMICAL | 10.1016/j.snb.2018.08.031 |
| 450 | Reanalysis of the anthrax epidemic in Rhodesia, 1978-1984 | Wilson, JM; Brediger, W; Albright, TP; Smith-Gagen, J | 2016 | PEERJ | 10.7717/peerj.2686 |
| 451 | Occurrence, heat and antibiotic resistance profile of <i>Bacillus cereus</i> isolated from raw cow and processed milk in Mezam Division, Cameroon | Fossi, BT; Akoachere, JFTK; Nchanji, GT; Wanji, S | 2017 | INTERNATIONAL JOURNAL OF DAIRY TECHNOLOGY | 10.1111/1471-0307.12315 |
| 452 | Dynamics of an Anthrax Model with Distributed Delay | Mushayabasa, S | 2016 | ACTA APPLICANDAE MATHEMATICAE | 10.1007/s10440-016-0040-y |
| 453 | A mathematical model of anthrax epidemic with behavioural change | Baloba, EB; Seidu, B | 2022 | MATHEMATICAL MODELLING AND CONTROL | 10.3934/mmc.2022023 |
| 454 | Immunogenicity of anthrax recombinant peptides and killed spores in goats and protective efficacy of immune sera in A/J mouse model | Ndumnego, OC; Koehler, SM; Crafford, JE; Beyer, W; van Heerden, H | 2018 | SCIENTIFIC REPORTS | 10.1038/s41598-018-35382-8 |
| 455 | Toxin-neutralizing antibodies elicited by naturally acquired cutaneous anthrax are elevated following severe disease and appear to target conformational epitopes | Dumas, EK; Demiraslan, H; Ingram, RJ; Sparks, RM; Muns, E; Zamora, A; Larabee, J; Garman, L; Ballard, JD; Boons, GJ; James, JA; Kayabas, U; Doganay, M; Farris, AD | 2020 | PLOS ONE | 10.1371/journal.pone.0230782 |
| 456 | An ethnobotanical study of medicinal plants used to treat livestock diseases in Onayena and Katima Mulilo, Namibia | Chinsembu, KC; Negumbo, J; Likando, M; Mbangu, A | 2014 | SOUTH AFRICAN JOURNAL OF BOTANY | 10.1016/j.sajb.2014.06.007 |
| 457 | Nanoscale Structural and Mechanical Analysis of <i>Bacillus anthracis</i> Spores Inactivated with Rapid Dry Heating | Xing, Y; Li, A; Felker, DL; Burggraf, LW | 2014 | APPLIED AND ENVIRONMENTAL MICROBIOLOGY | 10.1128/AEM.03483-13 |
| 458 | Gender Roles and One Health Risk Factors at the Human-Livestock-Wildlife Interface, Mpumalanga Province, South Africa | Coyle, AH; Berrian, AM; van Rooyen, J; Bagnol, B; Smith, MH | 2020 | ECOHEALTH | 10.1007/s10393-020-01478-9 |
| 459 | Single vector platform vaccine protects against lethal respiratory challenge with Tier 1 select agents of anthrax, plague, and tularemia | Jia, QM; Bowen, R; Dillon, BJ; Maslesa-Galic, S; Chang, BT; Kaidi, AC; Horwitz, MA | 2018 | SCIENTIFIC REPORTS | 10.1038/s41598-018-24581-y |
| 460 | Comparison of French and Worldwide <i>Bacillus anthracis</i> Strains Favors a Recent, Post-Columbian Origin of the Predominant North-American Clade | Vergnaud, G; Girault, G; Thierry, S; Pourcel, C; Madani, N; Blouin, Y | 2016 | PLOS ONE | 10.1371/journal.pone.0146216 |
| 461 | <i>In vitro</i> evaluation of the effect of linezolid and levofloxacin on Bacillus anthracis toxin production, spore formation and cell growth | Head, BM; Alfa, M; Sitar, DS; Rubinstein, E; Meyers, AFA | 2017 | JOURNAL OF ANTIMICROBIAL CHEMOTHERAPY | 10.1093/jac/dkw427 |
| 462 | A COINFECTED MODELING OF ANTHRAX AND LISTERIOSIS WITH POWER LAW | Nortey, SNN; Bonyah, E; Torny, M; Juga, M | 2022 | COMMUNICATIONS IN MATHEMATICAL BIOLOGY AND NEUROSCIENCE | 10.28919/cmbn/7231 |
| 463 | Temperature-mediated recombinant anthrax protective antigen aggregate development: Implications for toxin formation and immunogenicity | Amador-Molina, JC; Valerdi-Madrigal, ED; Dom√≠nguez-Castillo, RI; Sirota, LA; Arciniega, JL | 2016 | VACCINE | 10.1016/j.vaccine.2016.06.057 |
| 464 | Immunogenicity and Protective Efficacy of Recombinant Protective Antigen Anthrax Vaccine (GC1109) in A/J Mice Model | Kim, GL; Pyo, SW; Yi, H; Kim, SH; Shin, H; Yu, MA; Hwang, YR; Choi, SY; Jeon, JH; Jo, SK; Rhie, GE | 2023 | VACCINE | 10.1016/j.vaccine.2023.04.002 |
| 465 | Phylogeography of <i>Bacillus anthracis</i> in the Country of Georgia Shows Evidence of Population Structuring and Is Dissimilar to Other Regional Genotypes | Khmaladze, E; Birdsell, DN; Naumann, AA; Hochhalter, CB; Seymour, ML; Nottingham, R; Beckstrom-Sternberg, SM; Beckstrom-Sternberg, J; Nikolich, MP; Chanturia, G; Zhgenti, E; Zakalashvili, M; Malania, L; Babuadze, G; Tsertsvadze, N; Abazashvili, N; Kekelidze, M; Tsanava, S; Imnadze, P; Ganz, HH; Getz, WM; Pearson, O; Gajer, P; Eppinger, M; Ravel, J; Wagner, DM; Okinaka, RT; Schupp, JM; Keim, P; Pearson, T | 2014 | PLOS ONE | 10.1371/journal.pone.0102651 |
| 466 | Identification of a Substrate-selective Exosite within the Metalloproteinase Anthrax Lethal Factor | Goldberg, AB; Cho, E; Miller, CJ; Lou, HJ; Turk, BE | 2017 | JOURNAL OF BIOLOGICAL CHEMISTRY | 10.1074/jbc.M116.761734 |
| 467 | Isolation, Identification, Prevalence, and Genetic Diversity of <i>Bacillus cereus</i> Group Bacteria From Different Foodstuffs in Tunisia | Gdoura-Ben Amor, M; Siala, M; Zayani, M; Grosset, N; Smaoui, S; Messadi-Akrout, F; Baron, F; Jan, S; Gautier, M; Gdoura, R | 2018 | FRONTIERS IN MICROBIOLOGY | 10.3389/fmicb.2018.00447 |
| 468 | Elevation determines the spatial risk of Anthrax outbreaks in Karnataka, India | Chanda, MM; Prajapati, A; Yogisharadhya, R; Umesh, L; Palegar, MS; Hemadri, D; Shome, BR; Shivachandra, SB | 2023 | ACTA TROPICA | 10.1016/j.actatropica.2023.106848 |
| 469 | Comprehensive Analysis and Selection of Anthrax Vaccine Adsorbed Immune Correlates of Protection in Rhesus Macaques | Chen, LG; Schiffer, JM; Dalton, S; Sabourin, CL; Niemuth, NA; Plikaytis, BD; Quinn, CP | 2014 | CLINICAL AND VACCINE IMMUNOLOGY | 10.1128/CVI.00469-14 |
| 470 | Therapeutic prospects of endophytic<i> Bacillus</i> species from<i> Berberis</i><i> lycium</i> against oxidative stress and microbial pathogens | Nisa, S; Shoukat, M; Bibi, Y; Al Ayoubi, S; Shah, W; Masood, S; Sabir, M; Bano, SA; Qayyum, A | 2022 | SAUDI JOURNAL OF BIOLOGICAL SCIENCES | 10.1016/j.sjbs.2021.08.099 |
| 471 | Response surface modeling for the inactivation of <i>Bacillus subtilis</i> subsp <i>niger</i> spores by chlorine dioxide gas in an enclosed space | Wang, T; Qi, JC; Wu, JH; Hao, LM; Yi, Y; Lin, S; Zhang, ZX | 2016 | JOURNAL OF THE AIR & WASTE MANAGEMENT ASSOCIATION | 10.1080/10962247.2016.1150365 |
| 472 | Genome Subtyping of Autochthonous <i>Bacillus</i> Species Isolated from <i>Iru</i>, a Fermented <i>Parkia biglobosa</i> Seed | Adewumi, GA; Oguntoyinbo, FA; Romi, W; Singh, TA; Jeyaram, K | 2014 | FOOD BIOTECHNOLOGY | 10.1080/08905436.2014.931866 |
| 473 | Prevalence, virulence factor genes and antibiotic resistance of <i>Bacillus cereus sensu lato</i> isolated from dairy farms and traditional dairy products | Owusu-Kwarteng, J; Wuni, A; Akabanda, F; Tano-Debrah, K; Jespersen, L | 2017 | BMC MICROBIOLOGY | 10.1186/s12866-017-0975-9 |
| 474 | <i>Bacillus atrophaeus</i>: main characteristics and biotechnological applications - a review | Sella, SRBR; Vandenberghe, LPS; Soccol, CR | 2015 | CRITICAL REVIEWS IN BIOTECHNOLOGY | 10.3109/07388551.2014.922915 |
| 475 | Ixodid tick diversity on wild mammals, birds and reptiles in and around Etosha National Park, Namibia | Turner, WC; K√ºsters, M; Versfeld, W; Horak, IG | 2017 | AFRICAN JOURNAL OF ECOLOGY | 10.1111/aje.12369 |
| 476 | ANTITOXIN TREATMENT OF INHALATION ANTHRAX: A SYSTEMATIC REVIEW | Huang, E; Pillai, SK; Bower, WA; Hendricks, KA; Guarnizo, JT; Hoyle, JD; Gorman, SE; Boyer, AE; Quinn, CP; Meaney-Delman, D | 2015 | HEALTH SECURITY | 10.1089/hs.2015.0032 |
| 477 | Identification and characterization of a novel <i>Geobacillus thermoglucosidasius</i> bacteriophage, GVE3 | van Zyl, LJ; Sunda, F; Taylor, MP; Cowan, DA; Trindade, MI | 2015 | ARCHIVES OF VIROLOGY | 10.1007/s00705-015-2497-9 |
| 478 | Medicinal plants with traditional healthcare importance to manage human and livestock ailments in Enemay District, Amhara Region, Ethiopia | Birhan, YS; Kitaw, SL; Alemayehu, YA; Mengesha, NM | 2023 | ACTA ECOLOGICA SINICA | 10.1016/j.chnaes.2022.05.004 |
| 479 | Working conditions and public health risks in slaughterhouses in western Kenya | Cook, EAJ; de Glanville, WA; Thomas, LF; Kariuki, S; Bronsvoort, BMDC; F√®vre, EM | 2017 | BMC PUBLIC HEALTH | 10.1186/s12889-016-3923-y |
| 480 | Novel Synthesis of Thiolated Gold Nanoclusters Induced by Lanthanides for Ultrasensitive and Luminescent Detection of the Potential Anthrax Spores' Biomarker | Halawa, MI; Li, BS; Xu, GB | 2020 | ACS APPLIED MATERIALS & INTERFACES | 10.1021/acsami.0c10069 |
| 481 | Target-enrichment sequencing yields valuable genomic data for challenging-to-culture bacteria of public health importance | Dennis, TPW; Mable, BK; Brunelle, B; Devault, A; Carter, RW; Ling, CL; Mmbaga, BT; Halliday, JEB; Oravcova, K; Forde, TL | 2022 | MICROBIAL GENOMICS | 10.1099/mgen.0.000836 |
| 482 | Retrospective Screening of Anthrax-like Disease Induced by <i>Bacillus tropicus</i> str. JMT from Chinese Soft-Shell Turtles in Taiwan | Tsai, JM; Kuo, HW; Cheng, WT | 2023 | PATHOGENS | 10.3390/pathogens12050693 |
| 483 | Global Stability of an Anthrax Model with Environmental Decontamination and Time Delay | Mushayabasa, S | 2015 | DISCRETE DYNAMICS IN NATURE AND SOCIETY | 10.1155/2015/573146 |
| 484 | Evaluation of nine South African medicinal plants against attenuated <i>Bacillus anthracis</i> strain | Elisha, IL; Dzoyem, JP; Botha, FS; Eloff, JN | 2015 | SOUTH AFRICAN JOURNAL OF BOTANY | 10.1016/j.sajb.2015.03.039 |
| 485 | Total Fungi Counts and Metabolic Dynamics of Volatile Organic Compounds in Paddy Contaminated by <i>Aspergillus niger</i> During Storage Employing Gas Chromatography-Ion Mobility Spectrometry | He, PH; Hassan, MM; Tang, F; Jiang, H; Chen, MJ; Liu, R; Lin, H; Chen, QS | 2022 | FOOD ANALYTICAL METHODS | 10.1007/s12161-021-02186-y |
| 486 | Lethal exposure: An integrated approach to pathogen transmission via environmental reservoirs | Turner, WC; Kausrud, KL; Beyer, W; Easterday, WR; Barandongo, ZR; Blaschke, E; Cloete, CC; Lazak, J; Van Ert, MN; Ganz, HH; Turnbull, PCB; Stenseth, NC; Getz, WM | 2016 | SCIENTIFIC REPORTS | 10.1038/srep27311 |
| 487 | Exposure of Small-Scale Gold Miners in Prestea to Mercury, Ghana, 2012 | Iddrisah, FN; Yeboah-Manu, D; Nortey, PA; Nyarko, KM; Anim, J; Antara, SN; Kenu, E; Wurapa, F; Afari, EA | 2016 | PAN AFRICAN MEDICAL JOURNAL | 10.11604/pamj.supp.2016.25.1.6203 |
| 488 | Biohydrogen fermentation from <i>Pistia</i> <i>stratiotes</i> (aquatic weed) using mixed and pure bacterial cultures | Mthethwa, NP; Nasr, M; Kiambi, SL; Bux, F; Kumari, S | 2019 | INTERNATIONAL JOURNAL OF HYDROGEN ENERGY | 10.1016/j.ijhydene.2019.05.152 |
| 489 | Pathogens, disease, and the social-ecological resilience of protected areas | De Vos, A; Cumming, GS; Cumming, DHM; Ament, JM; Baum, J; Clements, HS; Grewar, JD; Maciejewski, K; Moore, C | 2016 | ECOLOGY AND SOCIETY | 10.5751/ES-07984-210120 |
| 490 | <i>In silico</i> analysis suggests interaction between Ebola virus and the extracellular matrix | Veljkovic, V; Glisic, S; Muller, CP; Scotch, M; Branch, DR; Perovic, VR; Sencanski, M; Veljkovic, N; Colombatti, A | 2015 | FRONTIERS IN MICROBIOLOGY | 10.3389/fmicb.2015.00135 |
| 491 | Genotypic Profiling of <i>Bacillus cereus</i> Recovered from Some Retail Foods in Ogun State, Nigeria, and Their Phylogenetic Relationship | Adesetan, TO; Efuntoye, MO; Babalola, OO | 2020 | INTERNATIONAL JOURNAL OF MICROBIOLOGY | 10.1155/2020/3750948 |
| 492 | Subterranean Mammals: Reservoirs of Infection or Overlooked Sentinels of Anthropogenic Environmental Soiling? | Retief, L; Bennett, NC; Jarvis, JUM; Bastos, ADS | 2017 | ECOHEALTH | 10.1007/s10393-017-1281-6 |
| 493 | <i>In vitro</i> antimicrobial, antioxidant and antiviral activities of the essential oil and various extracts of wild (<i>Daucus virgatus</i> (Poir.) Maire) from Tunisia | Snene, A; El Mokni, R; Jmii, H; Jlassi, I; Ja√Ødane, H; Falconieri, D; Piras, A; Dhaouadi, H; Porcedda, S; Hammami, S | 2017 | INDUSTRIAL CROPS AND PRODUCTS | 10.1016/j.indcrop.2017.08.015 |
| 494 | Establishment of a National Inventory of Dangerous Pathogens in the Republic of Uganda | Brizee, S; Kwehangana, M; Mwesigwa, C; Bleijs, DA; van den Berg, HHJL; Kampert, E; Makoba, MW; Kagirita, A; Makumbi, I; Kakooza, F; Onapa, MO; van Passel, MWJ | 2019 | HEALTH SECURITY | 10.1089/hs.2018.0112 |
| 495 | Namibian farmland cheetahs (<i>Acinonyx</i> <i>jubatus</i>) demonstrate seronegativity for antibodies against <i>Bacillus</i> <i>anthracis</i> | Switzer, A; Munson, L; Beesley, C; Wilkins, P; Blackburn, JK; Marker, L | 2016 | AFRICAN JOURNAL OF WILDLIFE RESEARCH | 10.3957/056.046.0139 |
| 496 | Cross-sectional survey of brucellosis and associated risk factors in the livestock-wildlife interface area of <i>Nechisar</i> National Park, Ethiopia | Chaka, H; Aboset, G; Garoma, A; Gumi, B; Thys, E | 2018 | TROPICAL ANIMAL HEALTH AND PRODUCTION | 10.1007/s11250-018-1528-4 |
| 497 | Dual-Readout Sandwich Immunoassay for Device-Free and Highly Sensitive Anthrax Biomarker Detection | Larkin, IN; Garimella, V; Yamankurt, G; Scott, AW; Xing, H; Mirkin, CA | 2020 | ANALYTICAL CHEMISTRY | 10.1021/acs.analchem.0c01090 |
| 498 | <i>Bacillus</i> spore germination: mechanisms, identification, and antibacterial strategies | Fan, LH; Zhang, YR; Ismail, BB; Muhammad, AI; Li, GL; Liu, DH | 2023 | CRITICAL REVIEWS IN FOOD SCIENCE AND NUTRITION | 10.1080/10408398.2023.2233184 |
| 499 | Long-term changes in the spatial distribution of lumpy skin disease hotspots in Zimbabwe | Swiswa, S; Masocha, M; Pfukenyi, DM; Dhliwayo, S; Chikerema, SM | 2017 | TROPICAL ANIMAL HEALTH AND PRODUCTION | 10.1007/s11250-016-1180-9 |
| 500 | Some scientific rationale for the use of the bark of <i>Ptaeroxylon</i> <i>obliquum</i> (Rutaceae) as anthrax remedy in South African Ethnoveterinary medicine | Famuyide, MI; Mcgaw, JL | 2021 | PLANTA MEDICA | 10.1055/s-0041-1736822 |
| 501 | Assessment of Vaccination Coverage of Sheep and Goat using Retrospective Data in Woreda Raya Alamata | Tadege, A; Afera, B | 2016 | MOMONA ETHIOPIAN JOURNAL OF SCIENCE | 10.4314/mejs.v8i1.7 |
| 502 | Designing a highly immunogenic multi epitope based subunit vaccine against Bacillus cereus | Rasheed, MA; Awais, M; Aldhahrani, A; Althobaiti, F; Alhazmi, A; Sattar, S; Afzal, U; Baeshen, HA; Enshasy, HAE; Dailin, DJ; AL-surhanee, AA; Kabir, F | 2021 | SAUDI JOURNAL OF BIOLOGICAL SCIENCES | 10.1016/j.sjbs.2021.06.082 |
| 503 | Benchmarking of different microbes for their biosurfactants antifungal action against plant pathogens | Meena, KR; Satyam; Singh, A; Jaiswal, A; Rai, D | 2022 | INDIAN JOURNAL OF EXPERIMENTAL BIOLOGY | 10.56042/ijeb.v60i12.58955 |
| 504 | Development and application of a Bacillus anthracis protective antigen domain-1 in-house ELISA for the detection of anti-protective antigen antibodies in cattle in Zambia (vol 13, e0205986, 2018) | Simbotwe, M; Fujikura, D; Ohnuma, M; Omori, R; Furuta, Y; Muuka, GM; Hang'ombe, BM; Higashi, H | 2019 | PLOS ONE | 10.1371/journal.pone.0211592 |
| 505 | Purification and characterization of gamma poly glutamic acid from newly <i>Bacillus lichenzforrnis</i> NRC20 | Tork, SE; Aly, MM; Alakilli, SY; Al-Seeni, MN | 2015 | INTERNATIONAL JOURNAL OF BIOLOGICAL MACROMOLECULES | 10.1016/j.ijbiomac.2014.12.017 |
| 506 | <i>In Trans</i> Complementation of Lethal Factor Reveal Roles in Colonization and Dissemination in a Murine Mouse Model | Lowe, DE; Ya, J; Glomski, IJ | 2014 | PLOS ONE | 10.1371/journal.pone.0095950 |
| 507 | Winning the battle against emerging pathogens A German response | Hunger, I | 2014 | BULLETIN OF THE ATOMIC SCIENTISTS | 10.1177/0096340214539133 |
| 508 | Antibacterial, antibiofilm activity and cytotoxicity of crude extracts of <it>Ptaeroxylon</it> <it>obliquum</it> (Ptaeroxylaceae) used in South African ethnoveterinary medicine against <it>Bacillus anthracis</it> Sterne vaccine strain | Famuyide, IM; Eloff, JN; Mcgaw, LJ | 2020 | FASEB JOURNAL | 10.1096/fasebj.2020.34.s1.00560 |
| 509 | Genome sequence analysis of a novel <i>Bacillus thuringiensis</i> strain BLB406 active against <i>Aedes aegypti</i> larvae, a novel potential bioinsecticide | Zghal, RZ; Ghedira, K; Elleuch, J; Kharrat, M; Tounsi, S | 2018 | INTERNATIONAL JOURNAL OF BIOLOGICAL MACROMOLECULES | 10.1016/j.ijbiomac.2018.05.119 |
| 510 | A survey of management practices and major diseases of dairy cattle in smallholdings in selected towns of Jimma zone, south-western Ethiopia | Duguma, B | 2020 | ANIMAL PRODUCTION SCIENCE | 10.1071/AN19079 |
| 511 | Mathematical Analysis of the Effects of Controls on the Transmission Dynamics of Anthrax in Both Animal and Human Populations | Baloba, EB; Seidu, B; Bornaa, CS | 2020 | COMPUTATIONAL AND MATHEMATICAL METHODS IN MEDICINE | 10.1155/2020/1581358 |
| 512 | Evaluation of a microfluidic chip system for preparation of bacterial DNA from swabs, air, and surface water samples | Julich, S; Hotzel, H; G√§rtner, C; Trouchet, D; Ahmed, MFE; Kemper, N; Tomaso, H | 2016 | BIOLOGICALS | 10.1016/j.biologicals.2016.06.013 |
| 513 | Spatial and Temporal Dynamics of a Mortality Event among Central African Great Apes | Cameron, KN; Reed, P; Morgan, DB; Ondzi√©, AI; Sanz, CM; K√ºhl, HS; Olson, SH; Leroy, E; Karesh, WB; Mundry, R | 2016 | PLOS ONE | 10.1371/journal.pone.0154505 |
| 514 | Climate variability, the proliferation and expansion of major livestock diseases in East Gojjam, Northwestern Ethiopia | Ayal, DY; Woldetisadik, M; Kassa, T; Tilahun, G; Leal, W | 2017 | INTERNATIONAL JOURNAL OF GLOBAL WARMING | 10.1504/IJGW.2017.084787 |
| 515 | Multiplex Nested Solid Phase PCR-Array Chip for Simultaneous Detection of Highly Pathogenic Microorganisms | Zhu, CC; Cui, JS; Hu, AZ; Yang, K; Zhao, J; Liu, Y; Deng, GQ; Zhu, L | 2019 | CHINESE JOURNAL OF ANALYTICAL CHEMISTRY | 10.19756/j.issn.0253-3820.191288 |
| 516 | Mixed Lignocellulosic Biomass Degradation and Utilization for Bacterial Cellulase Production | Oke, MA; Annuar, MSM; Simarani, K | 2017 | WASTE AND BIOMASS VALORIZATION | 10.1007/s12649-016-9595-0 |
| 517 | Anti-infective immunoadhesins from plants | Wycoff, K; Maclean, J; Belle, A; Yu, L; Tran, Y; Roy, C; Hayden, F | 2015 | PLANT BIOTECHNOLOGY JOURNAL | 10.1111/pbi.12441 |
| 518 | Applying Science: Opportunities to Inform Disease Management Policy with Cooperative Research within a One Health Framework | Blackburn, JK; Kracalik, IT; Fair, JM | 2016 | FRONTIERS IN PUBLIC HEALTH | 10.3389/fpubh.2015.00276 |
| 519 | Public health and economic benefits of spotted hyenas <i>Crocuta crocuta</i> in a peri-urban system | Sonawane, C; Yirga, G; Carter, NH | 2021 | JOURNAL OF APPLIED ECOLOGY | 10.1111/1365-2664.14024 |
| 520 | Effect of electrostatic force and thermal radiation of viscoelastic nanofluid flow with motile microorganisms surrounded by PST and PHF: Bacillus anthracis in biological applications | Boujelbene, M; Majeed, A; Baazaoui, N; Barghout, K; Ijaz, N; Abu-Libdeh, N; Naeem, S; Khan, I; Ali, MR | 2023 | CASE STUDIES IN THERMAL ENGINEERING | 10.1016/j.csite.2023.103691 |
| 521 | The significance of viral, bacterial and protozoan infections in zebra: a systematic review and meta-analysis of prevalence | Cossu, CA; Bhoora, RV; Cassini, R; van Heerden, H | 2022 | HYSTRIX-ITALIAN JOURNAL OF MAMMALOGY | 10.4404/hystrix-00501-2021 |
| 522 | 010 ANTIBACTERIAL ACTIVITY OF ETHNOMEDICINAL PLANTS OF IRULARS OF WESTERN GHATS | Shirley, R; Growther, L | 2015 | INTERNATIONAL JOURNAL OF PHARMACEUTICAL SCIENCES AND RESEARCH | 10.13040/IJPSR.0975-8232.6(7).2837-40 |
| 523 | A One-Health lens for anthrax Comment | Kock, R; Haider, N; Mboera, LEG; Zumla, A | 2019 | LANCET PLANETARY HEALTH | 10.1016/S2542-5196(19)30111-1 |
| 524 | Molecular analysis of bacterial community dynamics during the fermentation of <i>soy-daddawa</i> condiment | Ezeokoli, O; Gupta, A; Popoola, T; Bezuidenhout, C | 2016 | FOOD SCIENCE AND BIOTECHNOLOGY | 10.1007/s10068-016-0174-8 |
| 525 | Green Synthesis (<i>A-indica</i> Seed Extract) of Silver Nanoparticles (Ag-NPs), Characterization, Their Catalytic and Bactericidal Action Potential | Naz, M; Haider, A; Ikram, M; Qureshi, MZ; Ali, S | 2017 | NANOSCIENCE AND NANOTECHNOLOGY LETTERS | 10.1166/nnl.2017.2517 |
| 526 | Midguts of <i>Culex pipiens</i> L. (Diptera: Culicidae) as a potential source of raw milk contamination with pathogens | Adly, E; Hegazy, AA; Kamal, M; Abu-Hussien, SH | 2022 | SCIENTIFIC REPORTS | 10.1038/s41598-022-16992-9 |
| 527 | Bacteriophages for detection and control of foodborne bacterial pathogens-The case of <i>Bacillus cereus</i> and their phages | Abraha, HB; Kim, KP; Sbhatu, DB | 2023 | JOURNAL OF FOOD SAFETY | 10.1111/jfs.12906 |
| 528 | A genomic map of climate adaptation in Mediterranean cattle breeds | Flori, L; Moazami-Goudarzi, K; Alary, V; Araba, A; Boujenane, I; Boushaba, N; Casabianca, F; Casu, S; Ciampolini, R; Coeur d'acier, A; Coquelle, C; Delgado, JV; El-Beltagi, A; Hadjipavlou, G; Jousselin, E; Landi, V; Lauyie, A; Lecomte, P; Ligda, C; Marinthe, C; Martinez, A; Mastrangelo, S; Menni, D; Moulin, CH; Osman, MA; Pineau, O; Portolano, B; Rodellar, C; Saidi-Mehtar, N; Sechi, T; Semp√©r√©, G; Th√©venon, S; Tsiokos, D; Lalo√´, D; Gautier, M | 2019 | MOLECULAR ECOLOGY | 10.1111/mec.15004 |
| 529 | SYNTHESIS, CHARACTERIZATION, <i>IN-VITRO</i> ANTI-INFLAMMATORY AND ANTIMICROBIAL SCREENING OF METAL(II) MIXED DICLOFENAC AND ACETAMINOPHEN COMPLEXES | Obaleye, JA; Aliyu, AA; Rajee, AO; Bello, KE | 2021 | BULLETIN OF THE CHEMICAL SOCIETY OF ETHIOPIA | 10.4314/bcse.v35i1.6 |
| 530 | Antitrypanosomal activity of <i>Verbascum sinaiticum</i> Benth. (Scrophulariaceae) against <i>Trypanosoma congolense</i> isolates | Mergia, E; Shibeshi, W; Terefe, G; Teklehaymanot, T | 2016 | BMC COMPLEMENTARY AND ALTERNATIVE MEDICINE | 10.1186/s12906-016-1346-z |
| 531 | Exploring the application of biostimulation strategy for bacteria in the bioremediation of industrial effluent | Ijoma, GN; Selvarajan, R; Oyourou, JN; Sibanda, T; Matambo, T; Monanga, A; Mkansi, K | 2019 | ANNALS OF MICROBIOLOGY | 10.1007/s13213-019-1443-6 |
| 532 | Thermal annealing effect on structural and optical properties of 2,9-Bis [2-(4-chlorophenyl) ethyl] anthrax [2,1,9-def: 6,5,10-d'e'f'] diisoquinoline-1,3,8,10 (2H, 9H) tetrone (Ch-diisoQ) thin films | Qashou, SI; Rashad, M; Darwish, AAA; Hanafy, TA | 2017 | OPTICAL AND QUANTUM ELECTRONICS | 10.1007/s11082-017-1069-5 |
| 533 | Diversity, antimicrobial and antioxidant activities of culturable bacterial endophyte communities in <i>Aloe vera</i> | Akinsanya, MA; Goh, JK; Lim, SP; Ting, ASY | 2015 | FEMS MICROBIOLOGY LETTERS | 10.1093/femsle/fnv184 |
| 534 | Treatment of mine rejected-brine wastewater by indigenous halophilic bacterial species under aerobic and anaerobic conditions | Zintchem, AAEA; Kamika, I; Momba, MNB | 2021 | DESALINATION AND WATER TREATMENT | 10.5004/dwt.2021.27528 |
| 535 | Dielectric relaxation process and AC conductivity of 2,9-Bis [2-(4-chlorophenyl)ethyl] anthrax [2,1,9-def:6,5,10-d‚Ä≤e‚Ä≤f‚Ä≤] diisoquinoline-1,3,8,10 (2H,9H) tetron (Ch-diisoQ) thin films | Qashou, SI; Darwish, AAA; Alharbi, SR; Al Garni, SE; Hanafy, TA | 2017 | JOURNAL OF MATERIALS SCIENCE-MATERIALS IN ELECTRONICS | 10.1007/s10854-017-7283-x |
| 536 | Isolation, Partial Characterization and Application of Bacteriophages in Eradicating Biofilm Formation by <i>Bacillus cereus</i> on Stainless Steel Surfaces in Food Processing Facilities | Gdoura-Ben Amor, M; Culot, A; Techer, C; AlReshidi, M; Adnan, M; Jan, SP; Baron, F; Grosset, N; Snoussi, M; Gdoura, R; Gautier, M | 2022 | PATHOGENS | 10.3390/pathogens11080872 |
| 537 | A GCDGC-specific DNA (cytosine-5) methyltransferase that methylates the GCWGC sequence on both strands and the GCSGC sequence on one strand | Furuta, Y; Miura, F; Ichise, T; Nakayama, SMM; Ikenaka, Y; Zorigt, T; Tsujinouchi, M; Ishizuka, M; Ito, T; Higashi, H | 2022 | PLOS ONE | 10.1371/journal.pone.0265225 |
| 538 | Microwave-assisted synthesis, structural characterization, DFT studies, antibacterial and antioxidant activity of 2-methyl-4-oxo-1,2,3,4-tetrahydroquinazoline-2-carboxylic acid | Obafemi, CA; Fadare, OA; Jasinski, JP; Millikan, SP; Obuotor, EM; Iwalewa, EO; Famuyiwa, SO; Sanusi, K; Yilmaz, Y; Ceylan, √ú | 2018 | JOURNAL OF MOLECULAR STRUCTURE | 10.1016/j.molstruc.2017.11.018 |
| 539 | Two distinct non-ribosomal peptide synthetase-independent siderophore synthetase gene clusters identified in Armillaria and other species in the Physalacriaceae | Mensah, DLN; Wingfield, BD; Coetzee, MPA | 2023 | G3-GENES GENOMES GENETICS | 10.1093/g3journal/jkad205 |
| 540 | Synthesis of pyrazolo-1,2,4-triazolo[4,3-<i>a</i>]quinoxalines as antimicrobial agents with potential inhibition of DHPS enzyme | El-Attar, MAZ; Elbayaa, RY; Shaaban, OG; Habib, NS; Wahab, AEA; Abdelwahab, IA; El-Hawash, SAM | 2018 | FUTURE MEDICINAL CHEMISTRY | 10.4155/fmc-2018-0082 |
| 541 | Knowledge, attitude, and practices of cattle farmers regarding zoonotic diseases in Erzurum, Turkey | √ñzl√º, H; Atasever, M; Atasever, MA | 2020 | AUSTRAL JOURNAL OF VETERINARY SCIENCES |  |
| 542 | Cerebrospinal meningitis: lessons learnt from Africa | Simon, F; Boutin, JP; Milleliri, JM; Tournier, JN | 2019 | LANCET INFECTIOUS DISEASES | 10.1016/S1473-3099(19)30479-7 |
| 543 | Inhibitors of elastase stimulate murine B lymphocyte differentiation into IgG- and IgA-producing cells | Attia, Z; Rowe, JC; Kim, E; Varikuti, S; Steiner, HE; Zaghawa, A; Hassan, H; Cormet-Boyaka, E; Satoskar, AR; Boyaka, PN | 2018 | EUROPEAN JOURNAL OF IMMUNOLOGY | 10.1002/eji.201747264 |
| 544 | Chemical Profiling and Biological Screening of Some River Nile Derived-Microorganisms | Lotfy, MM; Hassan, HM; Mohammed, R; Hetta, M; El-Gendy, AO; Rateb, ME; Zaki, MA; Gamaleldin, NM | 2019 | FRONTIERS IN MICROBIOLOGY | 10.3389/fmicb.2019.00787 |
| 545 | Investigation of structural and electrical properties of 2,9-Bis [2-(4-2chlorophenyl)ethyl] anthrax [2,1,9-def:6,5,10-d‚Ä≤e‚Ä≤f‚Ä≤] diisoquinoline-1,3,8,10 (2H,9H) tetrone (Ch-diisoQ) nanostructured films for photoelectronic applications | Darwish, AAA; El-Zaidia, EFM; Qashou, SI | 2019 | PHYSICA B-CONDENSED MATTER | 10.1016/j.physb.2018.11.074 |
| 546 | A systematic review and meta-analysis of preclinical trials testing anti-toxin therapies for <i>B-anthracis</i> infection: A need for more robust study designs and results | Xu, WY; Ohanjandian, L; Sun, JF; Cui, XZ; Suffredini, D; Li, Y; Welsh, J; Eichacker, PQ | 2017 | PLOS ONE | 10.1371/journal.pone.0182879 |
| 547 | Quantitative Microbial Risk Assessment (QMRA) of Workers Exposure to Bioaerosols at MSW Open Dumpsites | Akpeimeh, GF; Fletcher, LA; Evans, BE; Ibanga, IE | 2021 | RISK ANALYSIS | 10.1111/risa.13670 |
| 548 | Heavy metals and antibiotics resistance of bacteria isolated from Marchica lagoon: biodegradation of anthracene on submerged aerated fixed bed reactor | Benghait, Y; Blaghen, M | 2022 | ENVIRONMENTAL TECHNOLOGY | 10.1080/09593330.2020.1839133 |
| 549 | ENVIRONMENTAL RECOVERY TRAINING FOR BIOLOGICAL INCIDENTS: IMPRESSIONS FROM PHARMACY STUDENTS | Pe√±a-Fern√°ndez, A; Pe√±a, MA; Ollero, MD; Hurtado, C; Fenoy, S | 2017 | 9TH INTERNATIONAL CONFERENCE ON EDUCATION AND NEW LEARNING TECHNOLOGIES (EDULEARN17) |  |
| 550 | Molecular diversity and hydrolytic enzymes production abilities of soil bacteria | Aarti, C; Khusro, A; Agastian, P; Darwish, NM; Al Farraj, DA | 2020 | SAUDI JOURNAL OF BIOLOGICAL SCIENCES | 10.1016/j.sjbs.2020.09.049 |
| 551 | A cross-validation-based approach for delimiting reliable home range estimates | Dougherty, ER; Carlson, CJ; Blackburn, JK; Getz, WM | 2017 | MOVEMENT ECOLOGY | 10.1186/s40462-017-0110-4 |
| 552 | The Use of Xenosurveillance to Detect Human Bacteria, Parasites, and Viruses in Mosquito Bloodmeals | Fauver, JR; Gendernalik, A; Weger-Lucarelli, J; Grubaugh, ND; Brackney, DE; Foy, BD; Ebel, GD | 2017 | AMERICAN JOURNAL OF TROPICAL MEDICINE AND HYGIENE | 10.4269/ajtmh.17-0063 |
| 553 | Production and Potential Genetic Pathways of Three Different Siderophore Types in <i>Streptomyces tricolor</i> Strain HM10 | Rehan, M; Barakat, H; Almami, IS; Qureshi, KA; Alsohim, AS | 2022 | FERMENTATION-BASEL | 10.3390/fermentation8080346 |
| 554 | Investigation on paper cup waste degradation by bacterial consortium and <i>Eudrillus eugeinea</i> through vermicomposting | Arumugam, K; Renganathan, S; Babalola, OO; Muthunarayanan, V | 2018 | WASTE MANAGEMENT | 10.1016/j.wasman.2017.11.009 |
| 555 | A novel tool for assessing microbiomes in cultural heritage documents | IOP; Righetti, PG; Zilberstein, G; Zilberstein, S | 2020 | INTERNATIONAL CONFERENCE FLORENCE HERI-TECH: THE FUTURE OF HERITAGE SCIENCE AND TECHNOLOGIES | 10.1088/1757-899X/949/1/012116 |
| 556 | Coursing hyenas and stalking lions: The potential for inter- and intraspecific interactions | Barker, NA; Joubert, FG; Kasaona, M; Shatumbu, G; Stowbunenko, V; Alexander, KA; Slotow, R; Getz, WM | 2023 | PLOS ONE | 10.1371/journal.pone.0265054 |
| 557 | Stability and bifurcation analysis of a nonlinear dynamical model studying the decline of vulture population | Tandon, A; Dutta, V; Mishra, Y | 2023 | INTERNATIONAL JOURNAL OF BIOMATHEMATICS | 10.1142/S1793524523500900 |
| 558 | Antimicrobial light-activated materials: towards application for food and environmental safety | Brovko, L; Anany, H; Bayoumi, M; Giang, K; Kunkel, E; Lim, E; Naboka, O; Rahman, S; Li, J; Filipe, CDM; Griffiths, MW | 2014 | JOURNAL OF APPLIED MICROBIOLOGY | 10.1111/jam.12622 |
| 559 | Antibacterial Activity of Newly Synthesized 5-Hydrazono-triazoloquinazolines | Al-Salahi, R; Rabab, ED; Mohamed, M | 2015 | LATIN AMERICAN JOURNAL OF PHARMACY |  |
| 560 | Myco-Synthesis of Silver Nanoparticles and Their Bioactive Role against Pathogenic Microbes | Abdel-Hadi, A; Iqbal, D; Alharbi, R; Jahan, S; Darwish, O; Alshehri, B; Banawas, S; Palanisamy, M; Ismail, A; Aldosari, S; Alsaweed, M; Madkhali, Y; Kamal, M; Fatima, F | 2023 | BIOLOGY-BASEL | 10.3390/biology12050661 |
| 561 | Seven new records of bee flies (Bombyliidae, Diptera) from Saudi Arabia | El-Hawagry, M; Al-Khalaf, AA; Soliman, AM; Abdel-Dayem, MS; Al Dhafer, HM | 2022 | EGYPTIAN JOURNAL OF BIOLOGICAL PEST CONTROL | 10.1186/s41938-022-00554-2 |
| 562 | Cytotoxicity and antimicrobial activity of isolated compounds from Monsonia angustifolia and Dodonaea angustifolia | Mcotshana, ZKS; McGaw, LJ; Kemboi, D; Fouche, G; Famuyide, IM; Krause, RWM; Siwe-Noundou, X; Tembu, VJ | 2023 | JOURNAL OF ETHNOPHARMACOLOGY | 10.1016/j.jep.2022.115170 |
| 563 | Isolation of a Membrane Protein Complex for Type VII Secretion in <i>Staphylococcus aureus</i> | Aly, KA; Anderson, M; Ohr, RJ; Missiakas, D | 2017 | JOURNAL OF BACTERIOLOGY | 10.1128/JB.00482-17 |
| 564 | Cytostatic and cytotoxic effects of a hot water and methanol extract of<i> Acokanthera</i><i> oppositifolia</i> in HepG2 hepatocarcinoma cells | Cordier, W; Steenkamp, P; Steenkamp, V | 2023 | JOURNAL OF ETHNOPHARMACOLOGY | 10.1016/j.jep.2023.116617 |
| 565 | Future threat from the past | El-Sayed, A; Kamel, M | 2021 | ENVIRONMENTAL SCIENCE AND POLLUTION RESEARCH | 10.1007/s11356-020-11234-9 |
| 566 | Exploring the Potential Anti-Inflammatory and Wound-Healing Proprieties of <i>Cepaea hortensis</i> Snail Mucin | Errajouani, F; Bakrim, H; Hourfane, S; Louajri, A; Rocha, JM; El Aouad, N; Laglaoui, A | 2023 | COSMETICS | 10.3390/cosmetics10060170 |
| 567 | Replicating bacterium-vectored vaccine expressing SARS-CoV-2 Membrane and Nucleocapsid proteins protects against severe COVID-19-like disease in hamsters | Jia, QM; Bielefeldt-Ohmann, H; Maison, RM; Maslesa-Galic, S; Cooper, SK; Bowen, RA; Horwitz, MA | 2021 | NPJ VACCINES | 10.1038/s41541-021-00321-8 |
| 568 | Film thickness effects on nanorods organic films of azo quinoline derivatives for optical applications | Mahmoud, AZ; Darwish, AAA; Qashou, SI | 2019 | PROGRESS IN NATURAL SCIENCE-MATERIALS INTERNATIONAL | 10.1016/j.pnsc.2019.04.009 |
| 569 | Antibacterial, antibiofilm, anti-inflammatory, and wound healing effects of nanoscale multifunctional cationic alternating copolymers | Hooshmand, SE; Ebadati, A; Hosseini, ES; Vahabi, AH; Oshaghi, M; Rahighi, R; Orooji, Y; Jahromi, MAM; Varma, RS; Hamblin, MR; Karimi, M | 2022 | BIOORGANIC CHEMISTRY | 10.1016/j.bioorg.2021.105550 |
| 570 | Recent progress in nanomaterial-based sensing of airborne viral and bacterial pathogens | Bhardwaj, SK; Bhardwaj, N; Kumar, V; Bhatt, D; Azzouz, A; Bhaumik, J; Kim, KH; Deep, A | 2021 | ENVIRONMENT INTERNATIONAL | 10.1016/j.envint.2020.106183 |
| 571 | Improvement of antibacterial activity of some sulfa drugs through linkage to certain phthalazin-1(2<i>H</i>)-one scaffolds | Ibrahim, HS; Eldehna, WM; Abdel-Aziz, HA; Elaasser, MM; Abdel-Aziz, MM | 2014 | EUROPEAN JOURNAL OF MEDICINAL CHEMISTRY | 10.1016/j.ejmech.2014.08.016 |
| 572 | Advocacy for identifying certain animal diseases as "neglected" | Roger, FL; Solano, P; Bouyer, J; Porphyre, V; Berthier, D; Peyre, M; Bonnet, P | 2017 | PLOS NEGLECTED TROPICAL DISEASES | 10.1371/journal.pntd.0005843 |
| 573 | Nanoparticles as potential new generation broad spectrum antimicrobial agents | Yah, CS; Simate, GS | 2015 | DARU-JOURNAL OF PHARMACEUTICAL SCIENCES | 10.1186/s40199-015-0125-6 |
| 574 | Insights Into SMAD4 Loss in Pancreatic Cancer From Inducible Restoration of TGF-Œ≤ Signaling | Fullerton, PT; Creighton, CJ; Matzuk, MM | 2015 | MOLECULAR ENDOCRINOLOGY | 10.1210/me.2015-1102 |
| 575 | <i>Staphylococcus aureus</i> Adenosine Inhibits sPLA2-IIA-Mediated Host Killing in the Airways | Pernet, E; Brunet, J; Guillemot, L; Chignard, M; Touqui, L; Wu, YZ | 2015 | JOURNAL OF IMMUNOLOGY | 10.4049/jimmunol.1402665 |
| 576 | Socio-demographic study on extent of knowledge, awareness, attitude, and risks of zoonotic diseases among livestock owners in Puducherry region | Rajkumar, K; Bhattacharya, A; David, S; Balaji, SH; Hariharan, R; Jayakumar, M; Balaji, N | 2016 | VETERINARY WORLD | 10.14202/vetworld.2016.1018-1024 |
| 577 | Phage-based detection of bacterial pathogens | van der Merwe, RG; van Helden, PD; Warren, RM; Sampson, SL; Gey van Pittius, NC | 2014 | ANALYST | 10.1039/c4an00208c |
| 578 | Fabrication of Micro and Nano Cantilevers Review | IEEE; Boukar, AJ; Shawesh, MA | 2017 | 2017 INTERNATIONAL CONFERENCE ON GREEN ENERGY & CONVERSION SYSTEMS (GECS) |  |
| 579 | Parasite-mediated selection drives an immunogenetic trade-off in plains zebras (<i>Equus quagga</i>) | Kamath, PL; Turner, WC; K√ºsters, M; Getz, WM | 2014 | PROCEEDINGS OF THE ROYAL SOCIETY B-BIOLOGICAL SCIENCES | 10.1098/rspb.2014.0077 |
| 580 | <i>Yersinia pestis</i> detection using biotinylated dNTPs for signal enhancement in lateral flow assays | Kortli, S; Jauset-Rubio, M; Tomaso, H; Abbas, MN; Bashammakh, AS; El-Shahawi, MS; Alyoubi, AO; Ben-Ali, M; O'Sullivan, CK | 2020 | ANALYTICA CHIMICA ACTA | 10.1016/j.aca.2020.03.059 |
| 581 | Effect of dual-frequency thermosonication, food matrix, and germinants on <i>Alicyclobacillus acidoterrestris</i> spore germination | Wahia, H; Fakayode, OA; Mintah, BK; Mustapha, AT; Zhou, CS; Dabbour, M | 2023 | FOOD RESEARCH INTERNATIONAL | 10.1016/j.foodres.2023.113054 |
| 582 | Detecting Biothreat Agents: From Current Diagnostics to Developing Sensor Technologies | Walper, SA; Aragon√©s, GL; Sapsford, KE; Brown, CW; Rowland, CE; Breger, JC; Medintz, IL | 2018 | ACS SENSORS | 10.1021/acssensors.8b00420 |
| 583 | Images in Clinical Tropical Medicine | P√©rez-Tanoira, R; Cuadros, J; Prieto-P√©rez, L | 2016 | AMERICAN JOURNAL OF TROPICAL MEDICINE AND HYGIENE | 10.4269/ajtmh.15-0763 |
| 584 | Modeling R<sub>0</sub> for Pathogens with Environmental Transmission: Animal Movements, Pathogen Populations, and Local Infectious Zones | Blackburn, JK; Ganz, HH; Ponciano, JM; Turner, WC; Ryan, SJ; Kamath, P; Cizauskas, C; Kausrud, K; Holt, RD; Stenseth, NC; Getz, WM | 2019 | INTERNATIONAL JOURNAL OF ENVIRONMENTAL RESEARCH AND PUBLIC HEALTH | 10.3390/ijerph16060954 |
| 585 | Uncovering the dormant food hazards, a review of foodborne microbial spores' detection and inactivation methods with emphasis on their application in the food industry | Farag, MA; Mesak, MA; Saied, DB; Ezzelarab, NM | 2021 | TRENDS IN FOOD SCIENCE & TECHNOLOGY | 10.1016/j.tifs.2020.10.037 |
| 586 | The cost of living in larger primate groups includes higher fly densities | Gogarten, JF; Jahan, M; Calvignac-Spencer, S; Chapman, CA; Goldberg, TL; Leendertz, FH; Rothman, JM | 2022 | ECOHEALTH | 10.1007/s10393-022-01597-5 |
| 587 | Using a Novel Lysin To Help <i>Control Clostridium</i> difficile Infections | Wang, Q; Euler, CW; Delaune, A; Fischetti, VA | 2015 | ANTIMICROBIAL AGENTS AND CHEMOTHERAPY | 10.1128/AAC.01357-15 |
| 588 | Integration of genomic and proteomic analyses in the classification of the <i>Siphoviridae</i> family | Adriaenssens, EM; Edwards, R; Nash, JHE; Mahadevan, P; Seto, D; Ackermann, HW; Lavigne, R; Kropinski, AM | 2015 | VIROLOGY | 10.1016/j.virol.2014.10.016 |
| 589 | Survey of laboratory-acquired infections around the world in biosafety level 3 and 4 laboratories | Wurtz, N; Papa, A; Hukic, M; Di Caro, A; Leparc-Goffart, I; Leroy, E; Landini, MP; Sekeyova, Z; Dumler, JS; Badescu, D; Busquets, N; Calistri, A; Parolin, C; Pal√π, G; Christova, I; Maurin, M; La Scola, B; Raoult, D | 2016 | EUROPEAN JOURNAL OF CLINICAL MICROBIOLOGY & INFECTIOUS DISEASES | 10.1007/s10096-016-2657-1 |
| 590 | Transboundary and infectious diseases of small ruminants: Knowledge, attitude, and practice of nomadic and semi-nomadic pastoralists in northern Iran | Jafari-Gh, A; Laven, RA; Eila, N; Yadi, J; Hatami, Z; Soleimani, P; Jafari-Gh, S; Lesko, MM; Sinafar, M; Heidari, E | 2020 | SMALL RUMINANT RESEARCH | 10.1016/j.smallrumres.2019.106039 |
| 591 | From Bits and Pieces to Whole Phage to Nanomachines: Pathogen Detection Using Bacteriophages | Anany, H; Chou, Y; Cucic, S; Derda, R; Evoy, S; Griffiths, MW | 2017 | ANNUAL REVIEW OF FOOD SCIENCE AND TECHNOLOGY, VOL 8 | 10.1146/annurev-food-041715-033235 |
| 592 | Assessment of socio-behavioural correlates and risk perceptions regarding anthrax disease in tribal communities of Odisha, Eastern India | Pattnaik, M.; Kshatri, J.S.; Choudhary, H.R.; Parai, D.; Shandilya, J.; Mansingh, A.; Padhi, A.K.; Pati, S.; Bhattacharya, D. | 2022 | BMC Infectious Diseases | 10.1186/s12879-022-07035-9 |
| 593 | Mathematical Analysis and Sensitivity Assessment of HIV/AIDS-Listeriosis Co-infection Dynamics | Chukwu, C.W.; Juga, M.L.; Chazuka, Z.; Mushanyu, J. | 2022 | International Journal of Applied and Computational Mathematics | 10.1007/s40819-022-01458-3 |
| 594 | Informing One Health Anthrax Surveillance and Vaccination Strategy from Spatial Analysis of Anthrax in Humans and Livestock in Ha Giang Province, Vietnam (1999 2020) | Luong, T.; Nguyen, T.T.; Trinh, V.B.; Walker, M.A.; Ha Hoang, T.T.; Pham, Q.T.; Hung Tran, T.M.; Pham, V.K.; Nguyen, V.L.; Pham, T.L.; Blackburn, J.K. | 2023 | American Journal of Tropical Medicine and Hygiene | 10.4269/ajtmh.22-0384 |
| 595 | Africa's drylands in a changing world: Challenges for wildlife conservation under climate and land-use changes in the Greater Etosha Landscape | Turner, W.C.; P√©riquet, S.; Goelst, C.E.; Vera, K.B.; Cameron, E.Z.; Alexander, K.A.; Belant, J.L.; Cloete, C.C.; du Preez, P.; Getz, W.M.; Hetem, R.S.; Kamath, P.L.; Kasaona, M.K.; Mackenzie, M.; Mendelsohn, J.; Mfune, J.K.E.; Muntifering, J.R.; Portas, R.; Scott, H.A.; Strauss, W.M.; Versfeld, W.; Wachter, B.; Wittemyer, G.; Kilian, J.W. | 2022 | Global Ecology and Conservation | 10.1016/j.gecco.2022.e02221 |
| 596 | Seroprevalence, associated risk factors, and molecular detection of bovine brucellosis in rural areas of Egypt | Elhaig, M.M.; Wahdan, A. | 2023 | Comparative Immunology, Microbiology and Infectious Diseases | 10.1016/j.cimid.2023.101971 |
| 597 | Synanthropic Flies‚ÄîA Review Including How They Obtain Nutrients, along with Pathogens, Store Them in the Crop and Mechanisms of Transmission | Stoffolano, J.G. | 2022 | Insects | 10.3390/insects13090776 |
| 598 | Spatial analysis of human and livestock anthrax in Dien Bien province, Vietnam (2010‚Äì2019) and the significance of anthrax vaccination in livestock | Tan, L.M.; Hung, D.N.; My, D.T.; Walker, M.A.; Ha, H.T.T.; Thai, P.Q.; Hung, T.T.M.; Blackburn, J.K. | 2022 | PLoS Neglected Tropical Diseases | 10.1371/journal.pntd.0010942 |
| 599 | A global examination of ecological niche modeling to predict emerging infectious diseases: a systematic review | Lawrence, T.J.; Takenaka, B.P.; Garg, A.; Tao, D.; Deem, S.L.; F√®vre, E.M.; Gluecks, I.; Sagan, V.; Shacham, E. | 2023 | Frontiers in Public Health | 10.3389/fpubh.2023.1244084 |
| 600 | Kaempferol: Antimicrobial Properties, Sources, Clinical, and Traditional Applications | Periferakis, A.; Periferakis, K.; Badarau, I.A.; Petran, E.M.; Popa, D.C.; Caruntu, A.; Costache, R.S.; Scheau, C.; Caruntu, C.; Costache, D.O. | 2022 | International Journal of Molecular Sciences | 10.3390/ijms232315054 |
| 601 | Alternative lifestyles: A plague persistence hypothesis | Wimsatt, J.; Eads, D.A.; Matchett, M.R.; Biggins, D.E. | 2023 | Ecosphere | 10.1002/ecs2.4673 |
| 602 | A scoping review of foot-and-mouth disease risk, based on spatial and spatio-temporal analysis of outbreaks in endemic settings | Gonz√°lez Gordon, L.; Porphyre, T.; Muhanguzi, D.; Muwonge, A.; Boden, L.; Bronsvoort, B.M.D.C. | 2022 | Transboundary and Emerging Diseases | 10.1111/tbed.14769 |
| 603 | One Health Assessment of Bacillus anthracis Incidence and Detection in Anthrax-Endemic Areas of Pakistan | Sardar, N.; Aziz, M.W.; Mukhtar, N.; Yaqub, T.; Anjum, A.A.; Javed, M.; Ashraf, M.A.; Tanvir, R.; Wolfe, A.J.; Schabacker, D.S.; Forrester, S.; Khemmani, M.; Aqel, A.A.; Warraich, M.A.; Shabbir, M.Z. | 2023 | Microorganisms | 10.3390/microorganisms11102462 |
| 604 | The African mosquito-borne diseasosome: Geographical patterns, range expansion and future disease emergence | Lehmann, T.; Kouam, C.; Woo, J.; Diallo, M.; Wilkerson, R.; Linton, Y.-M. | 2023 | Proceedings of the Royal Society B: Biological Sciences | 10.1098/rspb.2023.1581 |
| 605 | Exploring halophilic environments as a source of new antibiotics | Thompson, T.P.; Gilmore, B.F. | 2023 | Critical Reviews in Microbiology | 10.1080/1040841X.2023.2197491 |
| 606 | ENDEMIC SEROPREVALENCE AND FARMER CONTROL MEASURES OF ANTHRAX AMONG RUMINATE ANIMALS IN PAKISTAN | Sardar, N.; Yaqub, T.; Anjum, A.A.; Javed, M.; Ayub, S.; Mukhtar, N.; Aslam, H.B.; Wolfe, A.J.; Schabacker, D.S.; Forrester, S.; Khemmani, M.; Aqel, A.A.; Shahid, M.F.; Yaqub, S.; Ali, M.; Shabbir, M.A.; Shabbir, M.Z.; Nawaz, M.; Shafi, K.; Aziz, M.W. | 2023 | Journal of Animal and Plant Sciences | 10.36899/JAPS.2023.4.0685 |
| 607 | Proteomics approaches: A review regarding an importance of proteome analyses in understanding the pathogens and diseases | Zubair, M.; Wang, J.; Yu, Y.; Faisal, M.; Qi, M.; Shah, A.U.; Feng, Z.; Shao, G.; Wang, Y.; Xiong, Q. | 2022 | Frontiers in Veterinary Science | 10.3389/fvets.2022.1079359 |
| 608 | Knowledge, Attitudes, and Practices of Communal Livestock Farmers regarding Animal Health and Zoonoses in Far Northern KwaZulu-Natal, South Africa | Ngoshe, Y.B.; Etter, E.; Gomez-Vazquez, J.P.; Thompson, P.N. | 2023 | International Journal of Environmental Research and Public Health | 10.3390/ijerph20010511 |
| 609 | Co-created community contracts support biosecurity changes in a region where African swine fever is endemic ‚Äì Part II: Implementation of biosecurity measures | Chenais, E.; Fischer, K.; Aliro, T.; St√•hl, K.; Lewerin, S.S. | 2023 | Preventive Veterinary Medicine | 10.1016/j.prevetmed.2023.105902 |
| 610 | Carnivoran Ecology: The Evolution and Function of Communities | Buskirk, S.W. | 2023 | Carnivoran Ecology: the Evolution and Function of Communities | 10.1093/oso/9780192863249.001.0001 |
| 611 | Comprehensive updates on the biological features and metabolic potential of the versatile extremophilic actinomycete Nocardiopsis dassonvillei | Bhairamkar, S.; Kadam, P.; Anjulal, H.; Joshi, A.; Chaudhari, R.; Bagul, D.; Javdekar, V.; Zinjarde, S. | 2023 | Research in Microbiology | 10.1016/j.resmic.2023.104171 |
| 612 | Diseases of wild snow leopards and their wild ungulate prey | Ostrowski, S.; Gilbert, M. | 2023 | Snow Leopards | 10.1016/B978-0-323-85775-8.00050-9 |
| 613 | When wildlife comes to town: Interaction of sylvatic and domestic host animals in transmission of Echinococcus spp. in Namibia | Aschenborn, O.; Aschenborn, J.; Kern, P.; Mackenstedt, U.; Romig, T.; Wassermann, M. | 2023 | Helminthologia (Poland) | 10.2478/helm-2023-0012 |
| 614 | Ecological Role of Widespread Bacteriocin Production among Bacillus cereus Contaminating Dairy Products and Characterization of Three Cereins | Chaabouni, I.; Ouertani, A.; Koubaa, N.; Sassi, I.; Barkallah, I.; Saidi, M.; Cherif, A. | 2023 | Microbiology (Russian Federation) | 10.1134/S0026261723800019 |
| 615 | JMM Profile: Bacillus anthracis | Wales, A.; Mackintosh, A. | 2023 | Journal of Medical Microbiology | 10.1099/jmm.0.001747 |
| 616 | Feral Swine as Indirect Indicators of Environmental Anthrax Contamination and Potential Mechanical Vectors of Infectious Spores | Maison, R.M.; Priore, M.R.; Brown, V.R.; Bodenchuk, M.J.; Borlee, B.R.; Bowen, R.A.; Bosco-Lauth, A.M. | 2023 | Pathogens | 10.3390/pathogens12040622 |
| 617 | Diseases, Epidemics, and Diplomacy in Africa | Ndi, H.N.; Bang, H.N.; Kengo, E.E. | 2023 | Studies in Diplomacy and International Relations | 10.1007/978-3-031-41249-3_5 |
| 618 | Drivers of Human‚Äíwildlife interactions in a co-existence area: a case study of the Ngorongoro conservation area, Tanzania | Linuma, O.F.; Mahenge, A.S.; Mato, R.R.A.M.; Greenwood, A.D. | 2022 | Discover Sustainability | 10.1007/s43621-022-00113-7 |
| 619 | Development of conjugated secondary antibodies for wildlife disease surveillance | Ochai, S.O.; Crafford, J.E.; Kamath, P.L.; Turner, W.C.; van Heerden, H. | 2023 | Frontiers in Immunology | 10.3389/fimmu.2023.1221071 |
| 620 | Perceptions of livestock value chain actors (VCAs) on the risk of acquiring zoonotic diseases from their livestock in the central dry zone of Myanmar | Win, T.T.Z.; Campbell, A.; Soares Magalhaes, R.J.; Oo, K.N.; Henning, J. | 2023 | BMC Public Health | 10.1186/s12889-022-14968-y |
| 621 | One Health Meets the Exposome: Human, Wildlife, and Ecosystem Health | Ottinger, M.A.; Geiselman, C. | 2023 | One Health Meets the Exposome: Human, Wildlife, and Ecosystem Health | 10.1016/C2020-0-03393-1 |
| 622 | Microbial Risks Caused by Livestock Excrement: Current Research Status and Prospects | Abdugheni, R.; Li, L.; Yang, Z.-N.; Huang, Y.; Fang, B.-Z.; Shurigin, V.; Mohamad, O.A.A.; Liu, Y.-H.; Li, W.-J. | 2023 | Microorganisms | 10.3390/microorganisms11081897 |
| 623 | Assessment of knowledge and practices of hand hygiene among health workers in Rwanda | Gloria Umuhoza, A.; Bosco Kamugisha, J.; Nashwan, A.J.; Tahuna Soko, G. | 2023 | International Journal of Africa Nursing Sciences | 10.1016/j.ijans.2023.100585 |
| 624 | Prospective cohort study reveals unexpected aetiologies of livestock abortion in northern Tanzania | Thomas, K.M.; Kibona, T.; Claxton, J.R.; de Glanville, W.A.; Lankester, F.; Amani, N.; Buza, J.J.; Carter, R.W.; Chapman, G.E.; Crump, J.A.; Dagleish, M.P.; Halliday, J.E.B.; Hamilton, C.M.; Innes, E.A.; Katzer, F.; Livingstone, M.; Longbottom, D.; Millins, C.; Mmbaga, B.T.; Mosha, V.; Nyarobi, J.; Nyasebwa, O.M.; Russell, G.C.; Sanka, P.N.; Semango, G.; Wheelhouse, N.; Willett, B.J.; Cleaveland, S.; Allan, K.J. | 2022 | Scientific Reports | 10.1038/s41598-022-15517-8 |
| 625 | Spatiotemporal Patterns of Anthrax, Vietnam, 1990‚Äì2015 | Walker, M.A.; Tan, L.M.; Dang, L.H.; Khang, P.V.; Ha, H.T.T.; Hung, T.T.M.; Dung, H.H.; Anh, D.D.; Duong, T.N.; Hadfield, T.; Thai, P.Q.; Blackburn, J.K. | 2022 | Emerging Infectious Diseases | 10.3201/eid2811.212584 |
| 626 | The effects of fire frequency on vegetation structure and mammal assemblages in a savannah-woodland system | Nieman, W.A.; van Wilgen, B.W.; Radloff, F.G.T.; Tambling, C.J.; Leslie, A.J. | 2022 | African Journal of Ecology | 10.1111/aje.12971 |
| 627 | Developing Public Health Emergency Response Leaders in Incident Management: A Scoping Review of Educational Interventions | Li, Y.; Hsu, E.B.; Pham, N.; Davis, X.M.; Podgornik, M.N.; Trigoso, S.M. | 2022 | Disaster Medicine and Public Health Preparedness | 10.1017/dmp.2021.164 |
| 628 | Risk Analysis in Cattle Farmers‚Äô Prevention Practices of Anthrax and Foot and Mouth Disease in Yogyakarta Province, Indonesia | Guntoro, B.; Triatmojo, A.; Ariyadi, B.; Qui, N.H. | 2023 | Advances in Animal and Veterinary Sciences | 10.17582/journal.aavs/2023/11.6.987.997 |
| 629 | Determinants of livestock species ownership at household level: Evidence from rural OR Tambo District Municipality, South Africa | Taruvinga, A.; Kambanje, A.; Mushunje, A.; Mukarumbwa, P. | 2022 | Pastoralism | 10.1186/s13570-021-00220-6 |
| 630 | Prevalence, Antibiotics Resistance and Plasmid Profiling of Vibrio spp. Isolated from Cultured Shrimp in Peninsular Malaysia | Haifa-Haryani, W.O.; Amatul-Samahah, M.A.; Azzam-Sayuti, M.; Chin, Y.K.; Zamri-Saad, M.; Natrah, I.; Amal, M.N.A.; Satyantini, W.H.; Ina-Salwany, M.Y. | 2022 | Microorganisms | 10.3390/microorganisms10091851 |
| 631 | Seasonal Variation and Spatial-Temporal Pattern Analysis of Anthrax among Livestock in T√ºrkiye, 2005-2019 | Bayir, T. | 2023 | Pakistan Veterinary Journal | 10.29261/pakvetj/2023.059 |
| 632 | Evaluating the determinants of wildlife tolerance in the Kavango-Zambezi Transfrontier Conservation Area in Zimbabwe | Usman, M.F.; Le Bel, S.; Grimaud, P.; Nielsen, M.R. | 2023 | Journal for Nature Conservation | 10.1016/j.jnc.2023.126466 |
| 633 | Addressing biohazards to food security in primary production | Djurle, A.; Young, B.; Berlin, A.; V√•gsholm, I.; Blomstr√∂m, A.-L.; Nygren, J.; Kvarnheden, A. | 2022 | Food Security | 10.1007/s12571-022-01296-7 |
| 634 | An integrated model for anthrax-free zone development in developing countries | Sarker, M.S.A.; Shahid, M.A.H.; Rahman, M.B.; Nazir, K.H.M.N.H. | 2023 | Journal of Infection and Public Health | 10.1016/j.jiph.2023.10.024 |
| 635 | Distribution and Current State of Molecular Genetic Characterization in Pathogenic Free-Living Amoebae | Otero-Ruiz, A.; Gonzalez-Zu√±iga, L.D.; Rodriguez-Anaya, L.Z.; Lares-Jim√©nez, L.F.; Gonzalez-Galaviz, J.R.; Lares-Villa, F. | 2022 | Pathogens | 10.3390/pathogens11101199 |
| 636 | From Hippocrates to COVID-19: A bibliographic history of medicine | Stirling, D.A. | 2023 | From Hippocrates to COVID-19: A Bibliographic History of Medicine | 10.1201/9781003282785 |
| 637 | Bacillales: From Taxonomy to Biotechnological and Industrial Perspectives | Harirchi, S.; Sar, T.; Ramezani, M.; Aliyu, H.; Etemadifar, Z.; Nojoumi, S.A.; Yazdian, F.; Awasthi, M.K.; Taherzadeh, M.J. | 2022 | Microorganisms | 10.3390/microorganisms10122355 |
| 638 | Hippos alter their aggregations to mitigate density-dependent drought effects | Taillie, P.J.; Hartfelder, J.; Potash, A.; Pienaar, D.; Greaver, C.; Viljoen, P.; Fletcher, R.J.; Ferreira, S.; McCleery, R.A. | 2023 | Austral Ecology | 10.1111/aec.13317 |
| 639 | Kingdoms, empires, and domains: The history of high-level biological classification | Ragan, M.A. | 2023 | Kingdoms, Empires, and Domains: The History of High-Level Biological Classification | 10.1093/oso/9780197643037.001.0001 |
| 640 | Approaches for disease prioritization and decision-making in animal health, 2000‚Äì2021: a structured scoping review | Amenu, K.; McIntyre, K.M.; Moje, N.; Knight-Jones, T.; Rushton, J.; Grace, D. | 2023 | Frontiers in Veterinary Science | 10.3389/fvets.2023.1231711 |
| 641 | A review on recent developments in structural modification of TiO2 for food packaging applications | Kodithuwakku, P.; Jayasundara, D.R.; Munaweera, I.; Jayasinghe, R.; Thoradeniya, T.; Weerasekera, M.; Ajayan, P.M.; Kottegoda, N. | 2022 | Progress in Solid State Chemistry | 10.1016/j.progsolidstchem.2022.100369 |
| 642 | Cell Surface Hsp90- and Œ±MŒ≤2 Integrin-Mediated Uptake of Bacterial Flagellins to Activate Inflammasomes by Human Macrophages | Hoang, T.X.; Kim, J.Y. | 2022 | Cells | 10.3390/cells11182878 |
| 643 | Are hippos Africa's most influential megaherbivore? A review of ecosystem engineering by the semi-aquatic common hippopotamus | Voysey, M.D.; de Bruyn, P.J.N.; Davies, A.B. | 2023 | Biological Reviews | 10.1111/brv.12960 |
| 644 | Evaluation of knowledge, attitudes and practices regarding neosporosis and toxoplasmosis among farmers and animal health practitioners in Namibia | Samkange, A.; Chitanga, S.; Neves, L.; Matjila, T. | 2023 | Tropical Animal Health and Production | 10.1007/s11250-022-03441-y |
| 645 | Health and Safety Effects of Airborne Soil Dust in the Americas and Beyond | Tong, D.Q.; Gill, T.E.; Sprigg, W.A.; Van Pelt, R.S.; Baklanov, A.A.; Barker, B.M.; Bell, J.E.; Castillo, J.; Gass√≥, S.; Gaston, C.J.; Griffin, D.W.; Huneeus, N.; Kahn, R.A.; Kuciauskas, A.P.; Ladino, L.A.; Li, J.; Mayol-Bracero, O.L.; McCotter, O.Z.; M√©ndez-L√°zaro, P.A.; Mudu, P.; Nickovic, S.; Oyarzun, D.; Prospero, J.; Raga, G.B.; Raysoni, A.U.; Ren, L.; Sarafoglou, N.; Sealy, A.; Sun, Z.; Vimic, A.V. | 2023 | Reviews of Geophysics | 10.1029/2021RG000763 |
| 646 | Knowledge, Attitudes, Practices and Zoonotic Risk Perception of Bovine Q Fever (Coxiella burnetii) among Cattle Farmers and Veterinary Personnel in Northern Regions of Cameroon | Zangue, C.T.; Kouamo, J.; Ngoula, F.; Tawali, L.P.M.; Ndeb√©, M.M.F.; Somnjom, D.E.; Nguena, R.N.G.; Mouiche, M.M.M. | 2022 | Epidemiologia | 10.3390/epidemiologia3040036 |
| 647 | Global Health Perspectives on Race in Research: Neocolonial Extraction and Local Marginalization | Tankwanchi, A.S.; Asabor, E.N.; Vermund, S.H. | 2023 | International Journal of Environmental Research and Public Health | 10.3390/ijerph20136210 |
| 648 | Flavonoic content and antibacterial evaluation of Moringa oleifera Lam. leaves grow in Algeria | Boumaza-Hamladji, S.; Benhabyles, N.; Toubal, S.; El Haddad, D.; Bouchenak, O.; Bellalemi, N.; Berrichi, D.; Meziani, I. | 2023 | Journal of Advanced Pharmaceutical Technology and Research | 10.4103/JAPTR.JAPTR_126_23 |
| 649 | Medically important snakes and snakebite envenoming in Iran | Dehghani, R.; Monzavi, S.M.; Mehrpour, O.; Shirazi, F.M.; Hassanian-Moghaddam, H.; Keyler, D.E.; W√ºster, W.; Westerstr√∂m, A.; Warrell, D.A. | 2023 | Toxicon | 10.1016/j.toxicon.2023.107149 |
| 650 | Social insights on the implementation of One Health in zoonosis prevention and control: a scoping review | He, J.; Guo, Z.; Yang, P.; Cao, C.; Xu, J.; Zhou, X.; Li, S. | 2022 | Infectious Diseases of Poverty | 10.1186/s40249-022-00976-y |
| 651 | Fundamentals of Bioaerosols Science: From Physical to Biological Dimensions of Airborne Biological Particles | Yamamoto, N. | 2023 | Fundamentals of Bioaerosols Science: From Physical to Biological Dimensions of Airborne Biological Particles | 10.1016/C2020-0-01830-X |
| 652 | Advancing protected area effectiveness assessments by disentangling social-ecological interactions: A case study from the Luangwa Valley, Zambia | Frietsch, M.; Zafra-Calvo, N.; Ghoddousi, A.; Loos, J. | 2023 | Conservation Science and Practice | 10.1111/csp2.12974 |
| 653 | Genetic diversity of Brazilian Bacillus thuringiensis isolates with toxicity against Aedes aegypti (Diptera: Culicidae) | da Costa Fernandes, G.; de Prado Costa, D.K.; de Oliveira, N.S.; de Sousa, E.C.P.; Machado, D.H.B.; Polanczyk, R.A.; de Siqueira, H.√Å.A.; da Silva, M.C. | 2022 | Scientific Reports | 10.1038/s41598-022-18559-0 |
| 654 | The Effect of Climate Changes on Human Bacterial Infectious Diseases | Darbandi, A.; Ohadi, E.; Nezamzadeh, F.; Jalalifar, S.; Bialvaei, A.Z. | 2023 | Iranian Journal of Medical Microbiology | 10.30699/ijmm.17.3.273 |
| 655 | Fencing affects movement patterns of two large carnivores in Southern Africa | Naha, D.; P√©riquet, S.; Kilian, J.W.; Kupferman, C.A.; Hoth-Hanssen, T.; Beasley, J.C. | 2023 | Frontiers in Ecology and Evolution | 10.3389/fevo.2023.1031321 |
| 656 | Modeling the environmental suitability for Bacillus anthracis in the Qinghai Lake Basin, China | Arotolu, T.E.; Wang, H.; Lv, J.; Shi, K.; van Gils, H.; Huang, L.; Wang, X. | 2022 | PLoS ONE | 10.1371/journal.pone.0275261 |
| 657 | Pasteurella sp. associated with fatal septicaemia in six African elephants | Foggin, C.M.; Rosen, L.E.; Henton, M.M.; Buys, A.; Floyd, T.; Turner, A.D.; Tarbin, J.; Lloyd, A.S.; Chaitezvi, C.; Ellis, R.J.; Roberts, H.C.; Dastjerdi, A.; Nunez, A.; van Vliet, A.H.M.; Steinbach, F. | 2023 | Nature Communications | 10.1038/s41467-023-41987-z |
| 658 | Optimal control and cost-effectiveness analysis of anthrax epidemic model | Baloba, E.B.; Seidu, B.; Bornaa, C.S.; Okyere, E. | 2023 | Informatics in Medicine Unlocked | 10.1016/j.imu.2023.101355 |
| 659 | Non-Hepatotropic Viral, Bacterial and Parasitic Infections of the Liver | Zaki, S.R.; Alves, V.A.F.; Hale, G.L. | 2023 | MacSween's Pathology of the Liver, Eighth Edition | 10.1016/B978-0-7020-8228-3.00007-7 |
| 660 | Grappling with (re)-emerging infectious zoonoses: Risk assessment, mitigation framework, and future directions | Gwenzi, W.; Skirmuntt, E.C.; Musvuugwa, T.; Teta, C.; Halabowski, D.; Rzymski, P. | 2022 | International Journal of Disaster Risk Reduction | 10.1016/j.ijdrr.2022.103350 |
| 661 | Rapid population growth and high management costs have created a narrow window for control of introduced hippos in Colombia | Subalusky, A.L.; Sethi, S.A.; Anderson, E.P.; Jim√©nez, G.; Echeverri-Lopez, D.; Garc√≠a-Restrepo, S.; Nova-Le√≥n, L.J.; Re√°tiga-Parrish, J.F.; Post, D.M.; Rojas, A. | 2023 | Scientific Reports | 10.1038/s41598-023-33028-y |
| 662 | Spatiotemporal cluster of mpox in men who have sex with men: A modeling study in 83 countries | Shang, W.; Cao, G.; Wu, Y.; Kang, L.; Wang, Y.; Gao, P.; Liu, J.; Liu, M. | 2023 | Journal of Medical Virology | 10.1002/jmv.29166 |
| 663 | Ethical dimensions of zoonotic disease research: Perspectives of traditional livestock keepers in Zambia | Zulu, V.C.; Syakalima, M.; Ali, J. | 2023 | Wellcome Open Research | 10.12688/wellcomeopenres.17962.2 |
| 664 | Viral, Bacterial, Metabolic, and Autoimmune Causes of Severe Acute Encephalopathy in Sub-Saharan Africa: A Multicenter Cohort Study | Edridge, A.; Namazzi, R.; Tebulo, A.; Mfizi, A.; Deijs, M.; Koekkoek, S.; de Wever, B.; van der Ende, A.; Umiwana, J.; de Jong, M.D.; Jans, J.; Verhoeven-Duif, N.; Titulaer, M.; van Karnebeek, C.; Seydel, K.; Taylor, T.; Asiimwe-Kateera, B.; van der Hoek, L.; Kabayiza, J.-C.; Mallewa, M.; Idro, R.; Boele van Hensbroek, M.; van Woensel, J.B.M. | 2023 | Journal of Pediatrics | 10.1016/j.jpeds.2023.02.007 |
| 665 | Impact of COVID-19 on foodborne illness in Africa ‚Äì A perspective piece | Onyeaka, H.; Mazi, I.M.; Oladunjoye, I.O.; Njoagwuani, E.I.; Akegbe, H.; Dolapo, O.A.; Nwaiwu, O.; Tamasiga, P.; Ochulor, C.E. | 2023 | Journal of Infection and Public Health | 10.1016/j.jiph.2023.02.018 |
| 666 | Prevalence of Eucoleus garfiai in Wild Boars Hunted at Different Altitudes in the Campania and Latium Regions (Italy) | Power, K.; Martano, M.; Piscopo, N.; Viola, P.; Altamura, G.; Veneziano, V.; Carvajal Urue√±a, A.; Esposito, L. | 2023 | Animals | 10.3390/ani13040706 |
| 667 | Immunogenetics, sylvatic plague and its vectors: insights from the pathogen reservoir Mastomys natalensis in Tanzania | Haikukutu, L.; Lyaku, J.R.; Lyimo, C.M.; Eiseb, S.J.; Makundi, R.H.; Olayemi, A.; Wilhelm, K.; M√ºller-Klein, N.; Schmid, D.W.; Fleischer, R.; Sommer, S. | 2023 | Immunogenetics | 10.1007/s00251-023-01323-7 |
| 668 | Viability analysis of Kordofan giraffe (Giraffa camelopardalis antiquorum) in a protected area in Cameroon | Colston, K.P.J.; Johnson, C.L.; Nyugha, D.; Mengamenya¬†Gou√©, A.; Penny, S.G. | 2023 | African Journal of Ecology | 10.1111/aje.13196 |
| 669 | Anthrax revisited: how assessing the unpredictable can improve biosecurity | Sabra, D.M.; Krin, A.; Romeral, A.B.; Frie√ü, J.L.; Jeremias, G. | 2023 | Frontiers in Bioengineering and Biotechnology | 10.3389/fbioe.2023.1215773 |
| 670 | Environmental air sampling for detection and quantification of Mycobacterium tuberculosis in clinical settings: Proof of concept | Middelkoop, K.; Koch, A.S.; Hoosen, Z.; Bryden, W.; Call, C.; Seldon, R.; Warner, D.F.; Wood, R.; Andrews, J.R. | 2023 | Infection Control and Hospital Epidemiology | 10.1017/ice.2022.162 |
| 671 | Wastewater surveillance beyond COVID-19: a ranking system for communicable disease testing in the tri-county Detroit area, Michigan, USA | Gentry, Z.; Zhao, L.; Faust, R.A.; David, R.E.; Norton, J.; Xagoraraki, I. | 2023 | Frontiers in Public Health | 10.3389/fpubh.2023.1178515 |
| 672 | Machine learning-based farm risk management: A systematic mapping review | Ghaffarian, S.; van der Voort, M.; Valente, J.; Tekinerdogan, B.; de Mey, Y. | 2022 | Computers and Electronics in Agriculture | 10.1016/j.compag.2021.106631 |
| 673 | Identification of bio-climatic determinants and potential risk areas for Kyasanur forest disease in Southern India using MaxEnt modelling approach | Pramanik, M.; Singh, P.; Dhiman, R.C. | 2021 | BMC Infectious Diseases | 10.1186/s12879-021-06908-9 |
| 674 | Zootherapeutic uses of animals excreta: the case of elephant dung and urine use in Sayaboury province, Laos | Dubost, J.-M.; Kongchack, P.; Deharo, E.; Sysay, P.; Her, C.; Vichith, L.; S√©bastien, D.; Krief, S. | 2021 | Journal of Ethnobiology and Ethnomedicine | 10.1186/s13002-021-00484-7 |
| 675 | JACKALS, GOLDEN WOLVES, AND HONEY BADGERS: Cunning, Courage, and Conflict with Humans | Somerville, K. | 2022 | Jackals, Golden Wolves, and Honey Badgers: Cunning, Courage, and Conflict with Humans | 10.4324/9781003199793 |
| 676 | Anthrax as a potential biological warfare agent | Pile, J.C.; Malone, J.D.; Eitzen, E.M.; Friedlander, A.M. | 2022 | Bioterrorism: The History of a Crisis in American Society | 10.4324/9781003123644-19 |
| 677 | Miscellaneous Gram-Positive Bacterial Infections | Daniels, J.B.; Sykes, J.E. | 2022 | Greene's Infectious Diseases of the Dog and Cat, Fifth Edition | 10.1016/B978-0-323-50934-3.00052-5 |
| 678 | A Systematic Study on methods of Spatiotemporal Hotspot Detection and Evaluation metrics | Jain, R.; Bhat, A. | 2022 | Proceedings - 2022 4th International Conference on Advances in Computing, Communication Control and Networking, ICAC3N 2022 | 10.1109/ICAC3N56670.2022.10074268 |
| 679 | OPTIMAL CONTROL AND COST-EFFECTIVE ANALYSIS OF A SCABIES MODEL WITH DIRECT AND INDIRECT TRANSMISSIONS | Mhlanga, A.; Mupedza, T.V.; Mazikana, T.M. | 2022 | Journal of Biological Systems | 10.1142/S0218339022500097 |
| 680 | Predicting future distribution patterns of Jatropha gossypiifolia L. in South Africa in response to climate change | Moshobane, M.C.; Mudereri, B.T.; Mukundamago, M.; Chitata, T. | 2022 | South African Journal of Botany | 10.1016/j.sajb.2021.11.031 |
| 681 | Epizootological and epidemiological situation of anthrax in Ukraine in the context of mandatory specific prevention in susceptible animals | Korniienko, L.Y.; Ukhovskyi, V.V.; Moroz, O.A.; Chechet, O.M.; Haidei, O.S.; Tsarenko, T.M.; Bondarenko, T.M.; Karpulenko, M.S.; Nenych, N.P. | 2022 | Regulatory Mechanisms in Biosystems | 10.15421/022245 |
| 682 | Transient disease dynamics across ecological scales | Tao, Y.; Hite, J.L.; Lafferty, K.D.; Earn, D.J.D.; Bharti, N. | 2021 | Theoretical Ecology | 10.1007/s12080-021-00514-w |
| 683 | Structural and functional insights into the first Bacillus thuringiensis vegetative insecticidal protein of the Vpb4 fold, active against western corn rootworm | Kouadio, J.-L.; Zheng, M.; Aikins, M.; Duda, D.; Duff, S.; Chen, D.; Zhang, J.; Milligan, J.; Taylor, C.; Mamanella, P.; Rydel, T.; Kessenich, C.; Panosian, T.; Yin, Y.; Moar, W.; Giddings, K.; Park, Y.; Jerga, A.; Haas, J. | 2021 | PLoS ONE | 10.1371/journal.pone.0260532 |
| 684 | Knowledge, attitude and practices (KAP) towards Anthrax among livestock farmers in selected rural areas of Bangladesh | Dutta, P.K.; Biswas, H.; Ahmed, J.U.; Shakif-Ul-Azam, M.; Ahammed, B.M.J.; Dey, A.R. | 2021 | Veterinary Medicine and Science | 10.1002/vms3.561 |
| 685 | Risk factors and spatio-temporal patterns of livestock anthrax in Khuvsgul Province, Mongolia | Zorigt, T.; Ito, S.; Isoda, N.; Furuta, Y.; Shawa, M.; Norov, N.; Lkham, B.; Enkhtuya, J.; Higashi, H. | 2021 | PLoS ONE | 10.1371/journal.pone.0260299 |
| 686 | epidemiological and epizootiological situation on Anthrax around the World in 2021, the Forecast for 2022 in the Russian Federation | Skudareva, O.N.; Gerasimenko, D.K.; Logvin, F.V.; Chmerenko, D.K.; Semenova, O.V.; Aksenova, L.Yu.; Eremenko, E.I.; Buravtseva, N.P.; Golovinskaya, T.M.; Pechkovsky, G.A.; Kulichenko, A.N.; Ryazanova, A.G. | 2022 | Problemy Osobo Opasnykh Infektsii | 10.21055/0370-1069-2022-1-64-70 |
| 687 | EPIDEMIC RISK REDUCTION: A Civil Protection Approach | Gromek, P. | 2022 | Epidemic Risk Reduction: A Civil Protection Approach | 10.4324/9781003252856 |
| 688 | Landscape Heterogeneity and Woody Encroachment Decrease Mesocarnivore Scavenging in a Savanna Agroecosystem | Lima, K.A.; Stevens, N.; Wisely, S.M.; Fletcher, R.J.; Monadjem, A.; Austin, J.D.; Mahlaba, T.; McCleery, R.A. | 2021 | Rangeland Ecology and Management | 10.1016/j.rama.2021.06.003 |
| 689 | Man Versus Microbe: What Will It Take to Win? | Bremner, B. | 2022 | Man Versus Microbe: What Will It Take to Win? | 10.1142/q0329 |
| 690 | Ecological Niche Model of Bacillus cereus Group Isolates Containing a Homologue of the pXO1 Anthrax Toxin Genes Infecting Metalworkers in the United States | Deka, M.A.; Marston, C.K.; Garcia-Diaz, J.; Drumgoole, R.; Traxler, R.M. | 2022 | Pathogens | 10.3390/pathogens11040470 |
| 691 | Seropositivity rates of zoonotic pathogens in small ruminants and associated public health risks at informal urban markets in Zambia | Lysholm, S.; Fischer, K.; Lindahl, J.F.; Munyeme, M.; Wensman, J.J. | 2022 | Acta Tropica | 10.1016/j.actatropica.2021.106217 |
| 692 | Surveillance of antimicrobial resistant bacteria in flies (Diptera) in Rio de Janeiro city | Carramaschi, I.N.; Lopes, J.C.O.; Leite, J.A.; Carneiro, M.T.; Barbosa, R.R.; Boas, M.H.V.; Rangel, K.; Chagas, T.P.G.; Queiroz, M.M.; Zahner, V. | 2021 | Acta Tropica | 10.1016/j.actatropica.2021.105962 |
| 693 | Anthrax Bacterium: Epidemiology, Pathology, Dagnosis, Treatment and Remedial Interventions in Wild Animal | Puranik, N.; Yadav, S.K.; Singhal, S.; Khandia, R. | 2022 | Indian Veterinary Journal |  |
| 694 | The roles of environmental variation and parasite survival in virulence-transmission relationships | Turner, W.C.; Kamath, P.L.; Van Heerden, H.; Huang, Y.-H.; Barandongo, Z.R.; Bruce, S.A.; Kausrud, K. | 2021 | Royal Society Open Science | 10.1098/rsos.210088 |
| 695 | Detection of pathogens of veterinary importance harboured by Stomoxys calcitrans in South African feedlots | Makhahlela, N.B.; Liebenberg, D.; Van Hamburg, H.; Taioe, M.O.; Onyiche, T.; Ramatla, T.; Thekisoe, O.M.M. | 2022 | Scientific African | 10.1016/j.sciaf.2022.e01112 |
| 696 | A strategy for conserving old world vulture populations in the framework of one health | Ottinger, M.A.; Botha, A.; Buij, R.; Coverdale, B.; Gore, M.L.; Harrell, R.M.; Hassell, J.; Kr√ºger, S.; McClure, C.J.W.; Mullinax, J.M.; Shaffer, L.J.; Smit-Robinson, H.; Thompson, L.J.; Van Den Heever, L.; Bowerman, W.W. | 2021 | Journal of Raptor Research | 10.3356/JRR-20-98 |
| 697 | Zoonoses: Infectious diseases transmissible from animals to humans | Krauss, H.; Weber, A.; Appel, M.; Enders, B.; Isenberg, H.D.; Schiefer, H.G.; von Graevenitz, A.; Zahner, H. | 2022 | Zoonoses: Infectious Diseases Transmissible from Animals to Humans | 10.1128/9781555817787 |
| 698 | Qualitative Modeling for Bridging Expert-Knowledge and Social-Ecological Dynamics of an East African Savanna | Cosme, M.; H√©ly, C.; Pommereau, F.; Pasquariello, P.; Tiberi, C.; Treydte, A.; Gaucherel, C. | 2022 | Land | 10.3390/land11010042 |
| 699 | Phages for africa: The potential benefit and challenges of phage therapy for the livestock sector in sub-saharan Africa | Makumi, A.; Mhone, A.L.; Odaba, J.; Guantai, L.; Svitek, N. | 2021 | Antibiotics | 10.3390/antibiotics10091085 |
| 700 | Antibody response to Raboral VR-G¬Æ oral rabies vaccine in captive and free-ranging black-backed jackals (Canis mesomelas) | Koeppel, K.N.; Geertsma, P.; Kuhn, B.F.; van Schalkwyk, O.L.; Thompson, P.N. | 2022 | Onderstepoort Journal of Veterinary Research | 10.4102/ojvr.v89i1.1975 |
| 701 | Rationale and criteria for a covid-19 model framework | Messina, F.; Montaldo, C.; Abbate, I.; Antonioli, M.; Bordoni, V.; Matusali, G.; Sacchi, A.; Giombini, E.; Fimia, G.M.; Piacentini, M.; Capobianchi, M.R.; Lauria, F.N.; Ippolito, G. | 2021 | Viruses | 10.3390/v13071309 |
| 702 | A comparative GC‚ÄìMS analysis of bioactive secondary metabolites produced by halotolerant Bacillus spp. isolated from the Great Sebkha of Oran | Nas, F.; Aissaoui, N.; Mahjoubi, M.; Mosbah, A.; Arab, M.; Abdelwahed, S.; Khrouf, R.; Masmoudi, A.-S.; Cherif, A.; Klouche-Khelil, N. | 2021 | International Microbiology | 10.1007/s10123-021-00185-x |
| 703 | Over half of known human pathogenic diseases can be aggravated by climate change | Mora, C.; McKenzie, T.; Gaw, I.M.; Dean, J.M.; von Hammerstein, H.; Knudson, T.A.; Setter, R.O.; Smith, C.Z.; Webster, K.M.; Patz, J.A.; Franklin, E.C. | 2022 | Nature Climate Change | 10.1038/s41558-022-01426-1 |
| 704 | Temporal and spatial patterns of common hippopotamus populations in the Okavango Delta, Botswana | Inman, V.L.; Bino, G.; Kingsford, R.T.; Chase, M.J.; Leggett, K.E.A. | 2022 | Freshwater Biology | 10.1111/fwb.13868 |
| 705 | High-risk human-caused pathogen exposure events from 1975-2016 | Manheim, D.; Lewis, G. | 2022 | F1000Research | 10.12688/f1000research.55114.2 |
| 706 | Environmental suitability of Yersinia pestis and the spatial dynamics of plague in the Qinghai Lake region, China | Arotolu, T.E.; Wang, H.; Lv, J.; Kun, S.; Huang, L.; Wang, X. | 2022 | Veterinarni Medicina | 10.17221/81/2021-VETMED |
| 707 | Lycaon pictus (Carnivora: Canidae) | Bucci, M.E.; Nicholson, K.L.; Krausman, P.R. | 2022 | Mammalian Species | 10.1093/mspecies/seac002 |
| 708 | Geoinformatics, climate change, habitat dynamics and a case of vultures in central India | Jha, K.K. | 2021 | Critical Research Techniques in Animal and Habitat Ecology |  |
| 709 | Wildmeat consumption and zoonotic spillover: contextualising disease emergence and policy responses | Milbank, C.; Vira, B. | 2022 | The Lancet Planetary Health | 10.1016/S2542-5196(22)00064-X |
| 710 | Potential use for serosurveillance of feral swine to map risk for anthrax exposure, Texas, USA | Maison, R.M.; Pierce, C.F.; Ragan, I.K.; Brown, V.R.; Bodenchuk, M.J.; Bowen, R.A.; Bosco-Lauth, A.M. | 2021 | Emerging Infectious Diseases | 10.3201/eid2712.211482 |
| 711 | Critical Comparison between Large and Mini Vertical Flow Immunoassay Platforms forYersinia PestisDetection | Devadhasan, J.P.; Gu, J.; Chen, P.; Smith, S.; Thomas, B.; Gates-Hollingsworth, M.; Hau, D.; Pandit, S.; AuCoin, D.; Zenhausern, F. | 2021 | Analytical Chemistry | 10.1021/acs.analchem.0c05278 |
| 712 | Local community awareness and practices on Yersinia pestis plague disease management in Nkayi and Umzingwane districts, south-western Zimbabwe | Banda, A.; Gandiwa, E.; Muboko, N.; Mutanga, C.N.; Mashapa, C. | 2022 | Ecosystems and People | 10.1080/26395916.2022.2037714 |
| 713 | Novel scientific approaches to understanding emerging infectious diseases | Stevens, K.B. | 2021 | Routledge Handbook of Biosecurity and Invasive Species |  |
| 714 | Diagnosis of a Case of Enterotoxaemia in a Common Eland (Tragelaphus oryx) | Zheng, B.; Zhi, Y.; Li, Y.; Ding, Y.; Gao, Y.; Liu, J.; Zhang, C.; Wang, J. | 2022 | Chinese Journal of Wildlife | 10.12375/ysdwxb.20220324 |
| 715 | Spatially IntegratingMicrobiology and Geochemistry to Reveal Complex Environmental Health Issues: Anthrax in the Contiguous United States | Silvestri, E.E.; Douglas, S.H.; Luna, V.A.; Jean-Babtiste, C.A.O.; Pressman-Mashin n√©e Harbin, D.; Hempel, L.A.; Boe, T.R.; Nichols, T.L.; Griffin, D.W. | 2022 | Geospatial Technology for Human Well-Being and Health | 10.1007/978-3-030-71377-5_19 |
| 716 | Vulnerability and One Health assessment approaches for infectious threats from a social science perspective: a systematic scoping review | Jeleff, M.; Lehner, L.; Giles-Vernick, T.; D√ºckers, M.L.A.; Napier, A.D.; Jirovsky-Platter, E.; Kutalek, R. | 2022 | The Lancet Planetary Health | 10.1016/S2542-5196(22)00097-3 |
| 717 | Mapping habitat suitability for Asiatic black bear and red panda in Makalu Barun National Park of Nepal from Maxent and GARP models | Su, H.; Bista, M.; Li, M. | 2021 | Scientific Reports | 10.1038/s41598-021-93540-x |
| 718 | Endemic Thoracic Infections in Sub-Saharan Africa | Rydzak, C.E.; Lima, A.S.; Meirelles, G.S.P. | 2022 | Radiologic Clinics of North America | 10.1016/j.rcl.2022.01.003 |
| 719 | The direct and indirect effects of damming on the Hippopotamus amphibius population abundance and distribution at Bui National Park, Ghana | Bempah, G.; Grant, M.K.; Lu, C.; Borz√©e, A. | 2022 | Nature Conservation | 10.3897/natureconservation.50.87411 |
| 720 | A mixed-methods approach to understanding domestic dog health and disease transmission risk in an indigenous reserve in Guyana, South America | Milstein, M.S.; Shaffer, C.A.; Suse, P.; Marawanaru, A.; Heinrich, D.A.; Larsen, P.A.; Wolf, T.M. | 2022 | PLoS Neglected Tropical Diseases | 10.1371/journal.pntd.0010469 |
| 721 | Performance assessment of the new Xpert¬Æ HIV-1 viral load XC assay for quantification of HIV-1 viral loads | Ehret, R.; Harb, K.; Breuer, S.; Obermeier, M. | 2022 | Journal of Clinical Virology | 10.1016/j.jcv.2022.105127 |
| 722 | Development of ELISA based on Bacillus anthracis capsule biosynthesis protein CapA for naturally acquired antibodies against anthrax | Zorigt, T.; Furuta, Y.; Simbotwe, M.; Ochi, A.; Tsujinouchi, M.; Shawa, M.; Shimizu, T.; Isoda, N.; Enkhtuya, J.; Higashi, H. | 2021 | PLoS ONE | 10.1371/journal.pone.0258317 |
| 723 | Rabies in kudu: Revisited | M√ºller, T.; Hassel, R.; Jago, M.; Khaiseb, S.; van der Westhuizen, J.; Vos, A.; Calvelage, S.; Fischer, S.; Marston, D.A.; Fooks, A.R.; H√∂per, D.; Freuling, C.M. | 2022 | Advances in Virus Research | 10.1016/bs.aivir.2022.04.001 |
| 724 | Prevalence, Intensity, and Risk Factors for Helminth Infections in Pigs in Menoua, Western Highlands of Cameroon, with Some Data on Protozoa | Kouam, M.K.; Ngueguim, F.D. | 2022 | Journal of Parasitology Research | 10.1155/2022/9151294 |
| 725 | Clonal relationship between multidrug-resistant Escherichia coli ST69 from poultry and humans in Lusaka, Zambia | Shawa, M.; Furuta, Y.; Paudel, A.; Kabunda, O.; Mulenga, E.; Mubanga, M.; Kamboyi, H.; Zorigt, T.; Chambaro, H.; Simbotwe, M.; Hang'ombe, B.; Higashi, H. | 2021 | FEMS Microbiology Letters | 10.1093/femsle/fnac004 |
| 726 | A participatory epidemiological and One Health approach to explore the community‚Äôs capacity to detect emerging zoonoses and surveillance network opportunities in the forest region of Guinea | Guenin, M.-J.; De Nys, H.M.; Peyre, M.; Loire, E.; Thongyuanid, S.; Diallo, A.; Zogbelemou, L.; Goutard, F.L. | 2022 | PLoS Neglected Tropical Diseases | 10.1371/JOURNAL.PNTD.0010462 |
| 727 | Current trends in zoonoses and foodborne pathogens linked to the consumption of meat | Fegan, N.; McAuley, C.M.; Gray, J.A.; Duffy, L.L.; Namvar, A.; Warriner, K. | 2022 | New Aspects of Meat Quality: From Genes to Ethics, Second Edition | 10.1016/B978-0-323-85879-3.00020-9 |
| 728 | What Is Anthrax? | Bower, W.A.; Hendricks, K.A.; Vieira, A.R.; Traxler, R.M.; Weiner, Z.; Lynfield, R.; Hoffmaster, A. | 2022 | Pathogens | 10.3390/pathogens11060690 |
| 729 | Fiber Crop-Based Phytoremediation: Socio-Economic and Environmental Sustainability | Pandey, V.C.; Saikia, P.; Mahajan, P.; Praveen, A. | 2022 | Fiber Crop-Based Phytoremediation: Socio-Economic and Environmental Sustainability | 10.1016/C2020-0-00723-1 |
| 730 | Reviewing the role of vultures at the human-wildlife-livestock disease interface: An African perspective | Den Heever, L.V.; Thompson, L.J.; Bowerman, W.W.; Smit-Robinson, H.; Shaffer, L.J.; Harrell, R.M.; Ottinger, M.A. | 2021 | Journal of Raptor Research | 10.3356/JRR-20-22 |
| 731 | Breastfeeding: A Guide for the Medical Profession | Lawrence, R.A.; Lawrence, R.M. | 2022 | Breastfeeding: A Guide for the Medical Profession | 10.1016/C2018-0-02113-1 |
| 732 | Some Peculiarities of Anthrax Epidemiology in Herbivorous and Carnivorous Animals | Bakhteeva, I.; Timofeev, V. | 2022 | Life | 10.3390/life12060870 |
| 733 | Spatial clustering of fourteen tick species across districts of Zimbabwe | Shekede, M.D.; Chikerema, S.M.; Spargo, M.; Gwitira, I.; Kusangaya, S.; Mazhindu, A.N.; Ndhlovu, D.N. | 2021 | BMC Veterinary Research | 10.1186/s12917-021-02792-2 |
| 734 | The History of the Global Spread of the Causative Agent of Anthrax Based on the Whole Genome Phylogenetic Analysis of Bacillus anthracis | Onishchenko, G.G.; Kulichenko, A.N.; Eremenko, E.I.; Pisarenko, S.V. | 2022 | Vestnik Rossiiskoi Akademii Meditsinskikh Nauk | 10.15690/vramn2291 |
| 735 | Bacillus thuringiensis | Reyaz, A.L.; Balakrishnan, N.; Balasubramani, V.; Mohankumar, S. | 2022 | Microbial Approaches for Insect Pest Management | 10.1007/978-981-16-3595-3_3 |
| 736 | Brucellosis in wildlife in Africa: a systematic review and meta-analysis | Simpson, G.; Thompson, P.N.; Saegerman, C.; Marcotty, T.; Letesson, J.-J.; de Bolle, X.; Godfroid, J. | 2021 | Scientific Reports | 10.1038/s41598-021-85441-w |
| 737 | Reservoir dynamics of rabies in south-east Tanzania and the roles of cross-species transmission and domestic dog vaccination | Lushasi, K.; Hayes, S.; Ferguson, E.A.; Changalucha, J.; Cleaveland, S.; Govella, N.J.; Haydon, D.T.; Sambo, M.; Mchau, G.J.; Mpolya, E.A.; Mtema, Z.; Nonga, H.E.; Steenson, R.; Nouvellet, P.; Donnelly, C.A.; Hampson, K. | 2021 | Journal of Applied Ecology | 10.1111/1365-2664.13983 |
| 738 | The potential of diagnostic point-of-care tests (POCTs) for infectious and zoonotic animal diseases in developing countries: Technical, regulatory and sociocultural considerations | Hobbs, E.C.; Colling, A.; Gurung, R.B.; Allen, J. | 2021 | Transboundary and Emerging Diseases | 10.1111/tbed.13880 |
| 739 | Revisiting Koch's postulate to determine the plausibility of viral transmission by human milk | Van de Perre, P.; Mol√®s, J.-P.; Nagot, N.; Tuaillon, E.; Ceccaldi, P.-E.; Goga, A.; Prendergast, A.J.; Rollins, N. | 2021 | Pediatric Allergy and Immunology | 10.1111/pai.13473 |
| 740 | Assessment of listing and categorisation of animal diseases within the framework of the Animal Health Law (Regulation (EU) No 2016/429): antimicrobial-resistant Enterococcus faecalis in poultry | Nielsen, S.S.; Bicout, D.J.; Calistri, P.; Canali, E.; Drewe, J.A.; Garin-Bastuji, B.; Gonzales Rojas, J.L.; Gort√°zar, C.; Herskin, M.; Michel, V.; Miranda Chueca, M.√Å.; Padalino, B.; Pasquali, P.; Roberts, H.C.; Spoolder, H.; St√•hl, K.; Velarde, A.; Viltrop, A.; Winckler, C.; Baldinelli, F.; Broglia, A.; Kohnle, L.; Alvarez, J. | 2022 | EFSA Journal | 10.2903/j.efsa.2022.7127 |
| 741 | Disease and mortalities in selected zoological gardens in Nigeria | Akanbi, O.B.; Jegede, H.O.; Adam, M.; Oludairo, O.O.; Aiyedun, J.O.; Rimfa, A.G.; Ahmed, J.; Barde, I.J.; Hanga, A.B.; Ajadi, A.A.; Atata, J.A.; Taiwo, V.O.; Shoyinka, S.V.O. | 2021 | Comparative Clinical Pathology | 10.1007/s00580-021-03273-6 |
| 742 | Development of a geoinformation atlas of the sanitary and chemical state of water sources in the arid region | Kosarev, A.V.; Kosheleva, I.S.; Chekmizov, V.A.; Pankratova, Yu.A.; Mikerov, A.N. | 2022 | IOP Conference Series: Earth and Environmental Science | 10.1088/1755-1315/949/1/012014 |
| 743 | Spatio-temporal clustering and risk factor analysis of bovine theileriosis (Theileria parva) in Zimbabwe from 1995 to 2018 | Manyenyeka, M.; Tagwireyi, W.M.; Marufu, M.C.; Spargo, R.M.; Etter, E. | 2022 | Transboundary and Emerging Diseases | 10.1111/tbed.14081 |
| 744 | Safety and immunogenicity of a plant-derived recombinant protective antigen (rPA)-based vaccine against Bacillus anthracis: A Phase 1 dose-escalation study in healthy adults | Paolino, K.M.; Regules, J.A.; Moon, J.E.; Ruck, R.C.; Bennett, J.W.; Remich, S.A.; Mills, K.T.; Lin, L.; Washington, C.N.; Fornillos, G.A.; Lindsey, C.Y.; O'Brien, K.A.; Shi, M.; Mark Jones, R.; Green, B.J.; Tottey, S.; Chichester, J.A.; Streatfield, S.J.; Yusibov, V. | 2022 | Vaccine | 10.1016/j.vaccine.2022.01.047 |
| 745 | Updated checklist of the hard ticks (Acari: Ixodidae) of Egypt, with notes of livestock host and tick-borne pathogens | Okely, M.; Chen, Z.; Anan, R.; Gad-Allah, S. | 2022 | Systematic and Applied Acarology | 10.11158/saa.27.5.1 |
| 746 | From Amazon Floods and Australian Wildfires to Human Spills and Explosions: What Disasters Mean to Wildlife | Gallagher, C.; Fenton, H. | 2022 | Wildlife Population Health | 10.1007/978-3-030-90510-1_11 |
| 747 | Spatio-temporal patterns of lumpy skin disease outbreaks in dairy farms in northeastern Thailand | Punyapornwithaya, V.; Seesupa, S.; Phuykhamsingha, S.; Arjkumpa, O.; Sansamur, C.; Jarassaeng, C. | 2022 | Frontiers in Veterinary Science | 10.3389/fvets.2022.957306 |
| 748 | Antibiotic Isoflavonoids, Anthraquinones, and Pterocarpanoids from Pigeon Pea (Cajanus cajan L.) Seeds against Multidrug-Resistant Staphylococcus aureus | Balida, L.A.P.; Regalado, J.T.A.; Teodosio, J.J.R.; Dizon, K.A.H.; Sun, Z.; Zhan, Z.Q.; Blancaflor, J.M.D.; Sollesta, J.V.N.; Villorente, Z.M.; Saludes, J.P.; Dalisay, D.S. | 2022 | Metabolites | 10.3390/metabo12040279 |
| 749 | Anatomical, physiological, and behavioral mechanisms of thermoregulation in elephants | Dom√≠nguez-Oliva, A.; Ghezzi, M.D.; Mora-Medina, P.; Hern√°ndez-√Åvalos, I.; Jacome, J.; Castell√≥n, A.; Falc√≥n, I.; Res√©ndiz, F.; Romero, N.; Ponce, R.; Mota-Rojas, D. | 2022 | Journal of Animal Behaviour and Biometeorology | 10.31893/jabb.22033 |
| 750 | Anthrax | Fasanella, A.; Garofolo, G.; Donatiello, A.; Campese, E. | 2021 | Veterinary Vaccines: Principles and Applications | 10.1002/9781119506287.ch27 |
| 751 | Human anthrax in dolj county, romania‚Äîa series of three cases | Dumitrescu, F.; Georgescu, E.F.; Giubelan, L.; PƒÉdureanu, V.; Stoian, A.C.; DincƒÉ, V.; Georgescu, M.; Dragonu, L.; Marinescu, D. | 2021 | Pathogens | 10.3390/pathogens10060644 |
| 752 | Multi-source spatial data-based invasion risk modeling of Striga (Striga asiatica) in Zimbabwe | Mudereri, B.T.; Abdel-Rahman, E.M.; Dube, T.; Landmann, T.; Khan, Z.; Kimathi, E.; Owino, R.; Niassy, S. | 2020 | GIScience and Remote Sensing | 10.1080/15481603.2020.1744250 |
| 753 | Analysis of potentially suitable habitat within migration connections of an intra-African migrant-the Blue Swallow (Hirundo atrocaerulea) | Mudereri, B.T.; Mukanga, C.; Mupfiga, E.T.; Gwatirisa, C.; Kimathi, E.; Chitata, T. | 2020 | Ecological Informatics | 10.1016/j.ecoinf.2020.101082 |
| 754 | Temporal discrepancy of airborne total bacteria and pathogenic bacteria between day and night | Hu, Z.; Liu, H.; Zhang, H.; Zhang, X.; Zhou, M.; Lou, L.; Zheng, P.; Xi, C.; Hu, B. | 2020 | Environmental Research | 10.1016/j.envres.2020.109540 |
| 755 | Evaluation of the sensitivity and specificity of the lateral flow assay, Rose Bengal test and the complement fixation test for the diagnosis of brucellosis in cattle using Bayesian latent class analysis | Pfukenyi, D.M.; Meletis, E.; Modise, B.; Ndengu, M.; Kadzviti, F.W.; Dipuo, K.; Moesi, K.; Kostoulas, P.; Matope, G. | 2020 | Preventive Veterinary Medicine | 10.1016/j.prevetmed.2020.105075 |
| 756 | International regimes in global health governance | Jin, J. | 2021 | International Regimes in Global Health Governance | 10.4324/9781003148036 |
| 757 | Teaching Climate History: There is No Planet B | Singer, A.J. | 2021 | Teaching Climate History: There is No Planet B | 10.4324/9781003200864 |
| 758 | Review of the epizootiological and epidemiological situation on Anthrax around the World in 2020 and the Forecast for 2021 in the Russian Federation | Ryazanova, A.G.; Skudareva, O.N.; Gerasimenko, D.K.; Chmerenko, D.K.; Semenova, O.V.; Aksenova, L.Yu.; Eremenko, E.I.; Buravtseva, N.P.; Golovinskaya, T.M.; Pechkovsky, G.A.; Kulichenko, A.N. | 2021 | Problemy Osobo Opasnykh Infektsii | 10.21055/0370-1069-2021-1-81-86 |
| 759 | A gendered ecosystem services approach to identify novel and locally-relevant strategies for jointly improving food security, nutrition, and conservation in the Barotse Floodplain | Estrada-Carmona, N.; Attwood, S.; Cole, S.M.; Remans, R.; DeClerck, F. | 2020 | International Journal of Agricultural Sustainability | 10.1080/14735903.2020.1787618 |
| 760 | A qualitative exploratory study using one health approach for developing an intervention package for elimination of human anthrax in an endemic district of odisha, india | Mansingh, A.; Choudhary, H.; Shandilya, J.; Bhattacharya, D.; Kshatri, J.; Parai, D.; Pattanaik, M.; Padhi, A.K.; Jain, H.K.; Mohanty, P.; Kanungo, S.; Pati, S. | 2021 | Indian Journal of Medical Research | 10.4103/ijmr.IJMR-646-21 |
| 761 | Global stability and optimal control analysis of a foot-and-mouth disease model with vaccine failure and environmental transmission | Gashirai, T.B.; Musekwa-Hove, S.D.; Lolika, P.O.; Mushayabasa, S. | 2020 | Chaos, Solitons and Fractals | 10.1016/j.chaos.2019.109568 |
| 762 | Spatial distribution of cutaneous anthrax in western Iran from 2009 to 2016: Geographic information system mapping for predicting risk of anthrax outbreaks | Ghaderi, E.; Mohsenpour, B.; Moradi, G.; Karimi, M.; Najafi, F.; Nili, S.; Rouhi, S. | 2020 | Asian Pacific Journal of Tropical Medicine | 10.4103/1995-7645.283516 |
| 763 | Biting midges of Egypt (Diptera: Ceratopogonidae) | El-Hawagry, M.S.; El-Azab, S.E.-D.A.; Abdel-Dayem, M.S.; Al Dhafer, H.M. | 2020 | Biodiversity Data Journal | 10.3897/BDJ.8.e52357 |
| 764 | Bioaerosol field measurements: Challenges and perspectives in outdoor studies | ≈†antl-Temkiv, T.; Sikoparija, B.; Maki, T.; Carotenuto, F.; Amato, P.; Yao, M.; Morris, C.E.; Schnell, R.; Jaenicke, R.; P√∂hlker, C.; DeMott, P.J.; Hill, T.C.J.; Huffman, J.A. | 2020 | Aerosol Science and Technology | 10.1080/02786826.2019.1676395 |
| 765 | Spatial and spatio-temporal analysis of malaria cases in Zimbabwe | Gwitira, I.; Mukonoweshuro, M.; Mapako, G.; Shekede, M.D.; Chirenda, J.; Mberikunashe, J. | 2020 | Infectious Diseases of Poverty | 10.1186/s40249-020-00764-6 |
| 766 | Gas gangrene in mammals: a review | Junior, C.A.O.; Silva, R.O.S.; Lobato, F.C.F.; Navarro, M.A.; Uzal, F.A. | 2020 | Journal of Veterinary Diagnostic Investigation | 10.1177/1040638720905830 |
| 767 | Human Security in Disease and Disaster | Lindstaedt, N. | 2021 | Human Security in Disease and Disaster | 10.4324/9781003128809 |
| 768 | Pathogen detection and disease diagnosis in wildlife: challenges and opportunities | Michel, A.L.; van Heerden, H.; Prasse, D.; Rutten, V.; Al Dahouk, S.; Crossley, B.M. | 2021 | OIE Revue Scientifique et Technique | 10.20506/rst.40.1.3211 |
| 769 | Antibiotic-resistant Enterobacteriaceae from diseased freshwater goldfish | Preena, P.G.; Dharmaratnam, A.; Raj, N.S.; Raja, S.A.; Nair, R.R.; Swaminathan, T.R. | 2021 | Archives of Microbiology | 10.1007/s00203-020-02021-8 |
| 770 | A review of arguments for the existence of latent infections of bacillus anthracis, and research needed to understand their role in the outbreaks of anthrax | Gainer, R.S.; Vergnaud, G.; Hugh-Jones, M.E. | 2020 | Microorganisms | 10.3390/microorganisms8060800 |
| 771 | Implications of seasonal variations, host and vector migration on spatial spread of sleeping sickness: Insights from a mathematical model | Helikumi, M.; Lolika, P.O.; Mushayabasa, S. | 2021 | Informatics in Medicine Unlocked | 10.1016/j.imu.2021.100570 |
| 772 | Optimal control analysis applied to a two-patch model for Guinea worm disease | Mushayabasa, S.; Losio, A.A.E.; Modnak, C.; Wang, J. | 2020 | Electronic Journal of Differential Equations |  |
| 773 | Why behaviours do not change: structural constraints that influence household decisions to control pig diseases in Myanmar | Ebata, A.; MacGregor, H.; Loevinsohn, M.; Win, K.S. | 2020 | Preventive Veterinary Medicine | 10.1016/j.prevetmed.2020.105138 |
| 774 | Protected Areas and Food Security: Unravelling the Issues | Vasquez, W.; Sunderland, T. | 2020 | Participatory Biodiversity Conservation: Concepts, Experiences, and Perspectives | 10.1007/978-3-030-41686-7_4 |
| 775 | Systematic review of important viral diseases in africa in light of the ‚Äòone health‚Äô concept | Chauhan, R.P.; Dessie, Z.G.; Noreddin, A.; El Zowalaty, M.E. | 2020 | Pathogens | 10.3390/pathogens9040301 |
| 776 | Rabies in terrestrial animals | M√ºller, T.; Freuling, C.M. | 2020 | Rabies: Scientific Basis of the Disease and Its Management, Fourth Edition | 10.1016/B978-0-12-818705-0.00006-6 |
| 777 | Climatic changes and their role in emergence and re-emergence of diseases | El-Sayed, A.; Kamel, M. | 2020 | Environmental Science and Pollution Research | 10.1007/s11356-020-08896-w |
| 778 | Tuberculosis iris: Pathogenesis, presentation, and management across the spectrum of disease | Quinn, C.M.; Poplin, V.; Kasibante, J.; Yuquimpo, K.; Gakuru, J.; Cresswell, F.V.; Bahr, N.C. | 2020 | Life | 10.3390/life10110262 |
| 779 | A Review of Some Aspects of the Ecology, Population Trends, Threats and Conservation Strategies for the Common Hippopotamus, Hippopotamus amphibius L, in Zimbabwe | Utete, B. | 2020 | African Zoology | 10.1080/15627020.2020.1779613 |
| 780 | Silkworm model for Bacillus anthracis infection and virulence determination | Paudel, A.; Furuta, Y.; Higashi, H. | 2021 | Virulence | 10.1080/21505594.2021.1965830 |
| 781 | A Bibliometric Analysis of Corona Pandemic in Social Sciences: A Review of Influential Aspects and Conceptual Structure | Nasir, A.; Shaukat, K.; Hameed, I.A.; Luo, S.; Alam, T.M.; Iqbal, F. | 2020 | IEEE Access | 10.1109/ACCESS.2020.3008733 |
| 782 | The bacillus anthracis cell envelope: Composition, physiological role, and clinical relevance | Chateau, A.; Van der Verren, S.E.; Remaut, H.; Fioravanti, A. | 2020 | Microorganisms | 10.3390/microorganisms8121864 |
| 783 | Exploring environmental coverages of species: A new variable contribution estimation methodology for rulesets from the genetic algorithm for rule-set prediction | Yang, A.; Gomez, J.P.; Blackburn, J.K. | 2020 | PeerJ | 10.7717/peerj.8968 |
| 784 | The landscape of anthrax prevention and control: Stakeholders‚Äô perceptive in Odisha, India | Chandra Sahoo, K.; Negi, S.; Barla, D.; Badaik, G.; Sahoo, S.; Bal, M.; Padhi, A.K.; Pati, S.; Bhattacharya, D. | 2020 | International Journal of Environmental Research and Public Health | 10.3390/ijerph17093094 |
| 785 | Occupational Health and Safety Hazards in Agriculture: A Study on the Risks Involved for the Sustainability | Mishra, D.; Satapathy, S. | 2021 | Research Anthology on Cross-Industry Challenges of Industry 4.0 | 10.4018/978-1-6684-2405-6.ch075 |
| 786 | Geographical distribution of Anthrax using Geographic Information System (GIS) during 2010-2015 in Iran | Amiri, B.; Ghaderi, E.; Mohamadi, P.; Shirzadi, S.; Afrasiabian, S.; Zand, H.S.; Karimi, A.; Goodarzi, E.; Khazaei, Z.; Moayed, L. | 2021 | Medical Journal of the Islamic Republic of Iran | 10.34171/mjiri.35.36 |
| 787 | Bacteriophage as a therapeutic agent to combat bacterial infection: A journey from history to application | Panwar, U.; Aarthy, M.; Singh, S.K. | 2020 | Biocommunication of Phages | 10.1007/978-3-030-45885-0_17 |
| 788 | Modeling transmission dynamics of rabies in Nepal | Pantha, B.; Giri, S.; Joshi, H.R.; Vaidya, N.K. | 2021 | Infectious Disease Modelling | 10.1016/j.idm.2020.12.009 |
| 789 | Anthrax: Life cycle, mechanisms of pathogenesis and prospects in the development of veterinary vaccines (review) | Kondakova, O.A.; Nikitin, N.A.; Evtushenko, E.A.; Granovskiy, D.L.; Atabekov, J.G.; Karpova, O.V. | 2021 | Sel'skokhozyaistvennaya Biologiya | 10.15389/agrobiology.2021.3.415eng |
| 790 | Coxiella burnetii in Dromedary Camels (Camelus dromedarius): A Possible Threat for Humans and Livestock in North Africa and the Near and Middle East? | Devaux, C.A.; Osman, I.O.; Million, M.; Raoult, D. | 2020 | Frontiers in Veterinary Science | 10.3389/fvets.2020.558481 |
| 791 | Biosensors commercial off the shelf in biological warfare attack | Pohanka, M. | 2020 | Commercial Biosensors and Their Applications: Clinical, Food, and Beyond | 10.1016/B978-0-12-818592-6.00011-6 |
| 792 | A Review of Medicinal Uses, Phytochemistry and Biological Activities of Bolusanthus speciosus (Bolus) Harms (Fabaceae) | Maroyi, A. | 2020 | Journal of Pharmacy and Nutrition Sciences | 10.29169/1927-5951.2020.10.05.9 |
| 793 | Konzo outbreak in the Western Province of Zambia | Siddiqi, O.K.; Kapina, M.; Kumar, R.; Ngomah Moraes, A.; Kabwe, P.; Mazaba, M.L.; Hachaambwa, L.; Ng'uni, N.M.; Chikoti, P.C.; Morel-Espinosa, M.; Jarrett, J.M.; Baggett, H.C.; Chizema-Kawesha, E. | 2020 | Neurology | 10.1212/WNL.0000000000009017 |
| 794 | Prioritizing smallholder animal health needs in East Africa, West Africa, and South Asia using three approaches: Literature review, expert workshops, and practitioner surveys | Campbell, Z.; Coleman, P.; Guest, A.; Kushwaha, P.; Ramuthivheli, T.; Osebe, T.; Perry, B.; Salt, J. | 2021 | Preventive Veterinary Medicine | 10.1016/j.prevetmed.2021.105279 |
| 795 | Tropical Diseases of the Skin | Tiwary, A.K.; Kumar, P.; Vinay, S.; Anand, V.; Barkat, R.; Fatima, T. | 2021 | Atlas of Dermatology, Dermatopathology and Venereology: Cutaneous Infectious and Neoplastic Conditions and Procedural Dermatology | 10.1007/978-3-319-53805-1_78 |
| 796 | Study of vulture habitat suitability and impact of climate change in central india using maxent | Jha, K.K.; Jha, R. | 2021 | Journal of Resources and Ecology | 10.5814/j.issn.1674-764x.2021.01.004 |
| 797 | The Diversity Of Halotolerant And Halophilic Bacteria In The Soil Of The Nasinuan Secondary Forest In Maha Sarakham, Thailand | Kannika, C.; Thalisa, Y.A. | 2021 | Journal of Sustainability Science and Management | 10.46754/jssm.2021.02.017 |
| 798 | Social-ecological assessment of Lake Manyara basin, Tanzania: A mixed method approach | Janssens de Bisthoven, L.; Vanhove, M.P.M.; Rochette, A.-J.; Hug√©, J.; Verbesselt, S.; Machunda, R.; Munishi, L.; Wynants, M.; Steensels, A.; Malan-Meerkotter, M.; Henok, S.; Nhiwatiwa, T.; Casier, B.; Kiwango, Y.A.; Kaitila, R.; Komakech, H.; Brendonck, L. | 2020 | Journal of Environmental Management | 10.1016/j.jenvman.2020.110594 |
| 799 | Drug-induced Neurological Disorders, Fourth Edition | Jain, K.K. | 2021 | Drug-induced Neurological Disorders, Fourth Edition | 10.1007/978-3-030-73503-6 |
| 800 | Goat Production and Supply Chain Management in the Tropics | Rout, P.K.; Kumar, A.; Behera, B.K. | 2020 | Goat Production and Supply Chain Management in the Tropics |  |
| 801 | Review: The risk of contracting anthrax from spore-contaminated soil-A military medical perspective | Finke, E.-J.; Beyer, W.; Loderst√§dt, U.; Frickmann, H. | 2020 | European Journal of Microbiology and Immunology | 10.1556/1886.2020.00008 |
| 802 | Yersinia pestis: The natural history of Plague | Barbieri, R.; Signoli, M.; Chev√©, D.; Costedoat, C.; Tzortzis, S.; Aboudharam, G.; Raoult, D.; Drancourt, M. | 2021 | Clinical Microbiology Reviews | 10.1128/CMR.00044-19 |
| 803 | Black-backed jackals (Canis mesomelas) from semi-arid rangelands in South Africa harbour Hepatozoon canis and a Theileria species but apparently not Babesia rossi | Viljoen, S.; O'Riain, M.J.; Penzhorn, B.L.; Drouilly, M.; Vorster, I.; Bishop, J.M. | 2021 | Veterinary Parasitology: Regional Studies and Reports | 10.1016/j.vprsr.2021.100559 |
| 804 | Endophytic actinomycetes in Indo-Pak medicinal plants leading to new trends in drug discovery | Tanvir, R.; Sheikh, A.A.; Javeed, A. | 2021 | The Encyclopedia of Bacteriology Research Developments |  |
| 805 | Serological and molecular evidence of Brucella species in the rapidly growing pig sector in Kenya | Akoko, J.; Pelle, R.; Kivali, V.; Schelling, E.; Shirima, G.; MacHuka, E.M.; Mathew, C.; F√®vre, E.M.; Kyallo, V.; Falzon, L.C.; Lukambagire, A.S.; Halliday, J.E.B.; Bonfoh, B.; Kazwala, R.; Ouma, C. | 2020 | BMC Veterinary Research | 10.1186/s12917-020-02346-y |
| 806 | Yersinia pestis detection using biotinylated dNTPs for signal enhancement in lateral flow assays | Kortli, S.; Jauset-Rubio, M.; Tomaso, H.; Abbas, M.N.; Bashammakh, A.S.; El-Shahawi, M.S.; Alyoubi, A.O.; Ben-Ali, M.; O'Sullivan, C.K. | 2020 | Analytica Chimica Acta | 10.1016/j.aca.2020.03.059 |
| 807 | Identification of Escherichia coli and related enterobacteriaceae and examination of their phenotypic antimicrobial resistance patterns: A pilot study at a wildlife-livestock interface in Lusaka, Zambia | Kabali, E.; Pandey, G.S.; Munyeme, M.; Kapila, P.; Mukubesa, A.N.; Ndebe, J.; Muma, J.B.; Mubita, C.; Muleya, W.; Muonga, E.M.; Mitoma, S.; Hang‚Äôombe, B.M.; Wiratsudakul, A.; Ngan, M.T.; Elhanafy, E.; El Daous, H.; Huyen, N.T.; Yamazaki, W.; Okabayashi, T.; Abe, M.; Norimine, J.; Sekiguchi, S. | 2021 | Antibiotics | 10.3390/antibiotics10030238 |
| 808 | Mapping the geographic distribution of tungiasis in sub-Saharan Africa | Deka, M.A. | 2020 | Tropical Medicine and Infectious Disease | 10.3390/TROPICALMED5030122 |
| 809 | Impacts of climate change on the livestock food supply chain; a review of the evidence | Godde, C.M.; Mason-D'Croz, D.; Mayberry, D.E.; Thornton, P.K.; Herrero, M. | 2021 | Global Food Security | 10.1016/j.gfs.2020.100488 |
| 810 | Malaria and meningitis under climate change: initial assessment of climate information service in Nigeria | Ayanlade, A.; Sergi, C.; Ayanlade, O.S. | 2020 | Meteorological Applications | 10.1002/met.1953 |
| 811 | Vaginal host immune-microbiome interactions in a cohort of primarily African-American women who ultimately underwent spontaneous preterm birth or delivered at term | Florova, V.; Romero, R.; Tarca, A.L.; Galaz, J.; Motomura, K.; Ahmad, M.M.; Hsu, C.-D.; Hsu, R.; Tong, A.; Ravel, J.; Theis, K.R.; Gomez-Lopez, N. | 2021 | Cytokine | 10.1016/j.cyto.2020.155316 |
| 812 | Investigation of Genetic Relatedness of Brucella Strains in Countries Along the Silk Road | Liu, Z.; Wang, C.; Wei, K.; Zhao, Z.; Wang, M.; Li, D.; Wang, H.; Wei, Q.; Li, Z. | 2021 | Frontiers in Veterinary Science | 10.3389/fvets.2020.539444 |
| 813 | Elephants Under Human Care: The Behaviour, Ecology, and Welfare of Elephants in Captivity | Rees, P.A. | 2020 | Elephants Under Human Care: The Behaviour, Ecology, and Welfare of Elephants in Captivity | 10.1016/C2017-0-04747-X |
| 814 | The biased media and their utilization of propaganda: The true obstructionists of world events and our minds | Ross, D.B.; Peyton, G.L.; Sasso, M.T.; Matteson, R.W.; Matteson, C.E. | 2020 | Research Anthology on Fake News, Political Warfare, and Combatting the Spread of Misinformation | 10.4018/978-1-7998-7291-7.ch012 |
| 815 | A hippo in the room: Predicting the persistence and dispersion of an invasive mega-vertebrate in Colombia, South America | Castelblanco-Mart√≠nez, D.N.; Moreno-Arias, R.A.; Velasco, J.A.; Moreno-Bernal, J.W.; Restrepo, S.; Noguera-Urbano, E.A.; Baptiste, M.P.; Garc√≠a-Loaiza, L.M.; Jim√©nez, G. | 2021 | Biological Conservation | 10.1016/j.biocon.2020.108923 |
| 816 | CONTROLLING RABIES EPIDEMICS IN NEPAL WITH LIMITED RESOURCES: OPTIMAL CONTROL THEORY APPROACH | Pantha, B.; Joshi, H.R.; Vaidya, N.K. | 2020 | Mathematics in Applied Sciences and Engineering | 10.5206/mase/10847 |
| 817 | Control and prevention of anthrax, Texas, USA, 2019 | Sidwa, T.; Salzer, J.S.; Traxler, R.; Swaney, E.; Sims, M.L.; Bradshaw, P.; O‚ÄôSullivan, B.J.; Parker, K.; Waldrup, K.A.; Bower, W.A.; Hendricks, K. | 2020 | Emerging Infectious Diseases | 10.3201/EID2612.200470 |
| 818 | Livelihood, food and nutrition security in Southern Africa: What role do indigenous cattle genetic resources play? | Mapiye, O.; Chikwanha, O.C.; Makombe, G.; Dzama, K.; Mapiye, C. | 2020 | Diversity | 10.3390/d12020074 |
| 819 | Yersinia pestis plasminogen activator | Sebbane, F.; Uversky, V.N.; Anisimov, A.P. | 2020 | Biomolecules | 10.3390/biom10111554 |
| 820 | Melanin biosynthesis in bacteria, regulation and production perspectives | Pavan, M.E.; L√≥pez, N.I.; Pettinari, M.J. | 2020 | Applied Microbiology and Biotechnology | 10.1007/s00253-019-10245-y |
| 821 | Embracing nature's complexity: Immunoparasitology in the wild | Mair, I.; McNeilly, T.N.; Corripio-Miyar, Y.; Forman, R.; Else, K.J. | 2021 | Seminars in Immunology | 10.1016/j.smim.2021.101525 |
| 822 | Khoe-san genomes reveal unique variation and confirm the deepest population divergence in homo sapiens | Schlebusch, C.M.; Sj√∂din, P.; Breton, G.; G√ºnther, T.; Naidoo, T.; Hollfelder, N.; Sj√∂strand, A.E.; Xu, J.; Gattepaille, L.M.; Vicente, M.; Scofield, D.G.; Malmstr√∂m, H.; De Jongh, M.; Lombard, M.; Soodyall, H.; Jakobsson, M. | 2020 | Molecular Biology and Evolution | 10.1093/molbev/msaa140 |
| 823 | Geographically structured genomic diversity of non-human primate-infecting treponema pallidum subsp. Pertenue | Mubemba, B.; Gogarten, J.F.; Schuenemann, V.J.; D√ºx, A.; Lang, A.; Nowak, K.; Pl√©h, K.; Reiter, E.; Ulrich, M.; Agbor, A.; Brazzola, G.; Deschner, T.; Dieguez, P.; Granjon, A.; Jones, S.; Junker, J.; Wessling, E.; Arandjelovic, M.; Kuehl, H.; Wittig, R.M.; Leendertz, F.H.; Calvignac-Spencer, S. | 2020 | Microbial Genomics | 10.1099/mgen.0.000463 |
| 824 | Power, participation and their problems: A consideration of power dynamics in the use of participatory epidemiology for one health and zoonoses research | Ebata, A.; Hodge, C.; Braam, D.; Waldman, L.; Sharp, J.; MacGregor, H.; Moore, H. | 2020 | Preventive Veterinary Medicine | 10.1016/j.prevetmed.2020.104940 |
| 825 | With or without a vaccine-a review of complementary and alternative approaches to managing african swine fever in resource-constrained smallholder settings | Penrith, M.-L.; Bastos, A.; Chenais, E. | 2021 | Vaccines | 10.3390/vaccines9020116 |
| 826 | Bio-aerosols negatively affect Prochlorococcus in oligotrophic aerosol-rich marine regions | Rahav, E.; Paytan, A.; Mescioglu, E.; Bar-Zeev, E.; Ruiz, F.M.; Xian, P.; Herut, B. | 2020 | Atmosphere | 10.3390/atmos11050540 |
| 827 | Ugandan cattle farmers' perceived needs of disease prevention and strategies to improve biosecurity | Wolff, C.; Abigaba, S.; Sternberg Lewerin, S. | 2019 | BMC Veterinary Research | 10.1186/s12917-019-1961-2 |
| 828 | Analysis of spatio-temporal rainfall trends across southern African biomes between 1981 and 2016 | Marumbwa, F.M.; Cho, M.A.; Chirwa, P.W. | 2019 | Physics and Chemistry of the Earth | 10.1016/j.pce.2019.10.004 |
| 829 | Spatial ecology of male hippopotamus in a changing watershed | Stears, K.; Nu√±ez, T.A.; Muse, E.A.; Mutayoba, B.M.; McCauley, D.J. | 2019 | Scientific Reports | 10.1038/s41598-019-51845-y |
| 830 | Modelling the dynamics of direct and pathogens-induced dysentery diarrhoea epidemic with controls | Berhe, H.W.; Makinde, O.D.; Theuri, D.M. | 2019 | Journal of Biological Dynamics | 10.1080/17513758.2019.1588400 |
| 831 | Species identification of adult African blowflies (Diptera: Calliphoridae) of forensic importance | Lutz, L.; Williams, K.A.; Villet, M.H.; Ekanem, M.; Szpila, K. | 2018 | International Journal of Legal Medicine | 10.1007/s00414-017-1654-y |
| 832 | Modeling the present and future distribution of arbovirus vectors Aedes aegypti and Aedes albopictus under climate change scenarios in Mainland China | Liu, B.; Gao, X.; Ma, J.; Jiao, Z.; Xiao, J.; Hayat, M.A.; Wang, H. | 2019 | Science of the Total Environment | 10.1016/j.scitotenv.2019.01.301 |
| 833 | Techniques for Vaccinating Wildlife | Gilbert, M. | 2018 | Miller - Fowler's Zoo and Wild Animal Medicine Current Therapy: Volume 9 | 10.1016/B978-0-323-55228-8.00044-8 |
| 834 | Modelling the potential distribution of arbovirus vector Aedes aegypti under current and future climate scenarios in Taiwan, China | Liu, B.; Jiao, Z.; Ma, J.; Gao, X.; Xiao, J.; Hayat, M.A.; Wang, H. | 2019 | Pest Management Science | 10.1002/ps.5424 |
| 835 | Soil components and human health | Nieder, R.; Benbi, D.K.; Reichl, F.X. | 2018 | Soil Components and Human Health | 10.1007/978-94-024-1222-2 |
| 836 | Fire and browsers in Savannas: Traits, interactions, and continent-level patterns | Hempson, G.P.; Archibald, S.; Staver, C. | 2019 | Savanna Woody Plants and Large Herbivores | 10.1002/9781119081111.ch13 |
| 837 | Seroprevalence and associated risk factors of rift valley fever in cattle and selected wildlife species at the livestock/wildlife interface areas of gonarezhou national park, Zimbabwe | Ndengu, M.; Matope, G.; Tivapasi, M.; Pfukenyi, D.M.; Cetre-Sossah, C.; De Garine-Wichatitsky, M. | 2020 | Onderstepoort Journal of Veterinary Research | 10.4102/ojvr.v87i1.1731 |
| 838 | Simultaneous outbreaks of respiratory disease in wild chimpanzees caused by distinct viruses of human origin | Negrey, J.D.; Reddy, R.B.; Scully, E.J.; Phillips-Garcia, S.; Owens, L.A.; Langergraber, K.E.; Mitani, J.C.; Emery Thompson, M.; Wrangham, R.W.; Muller, M.N.; Otali, E.; Machanda, Z.; Hyeroba, D.; Grindle, K.A.; Pappas, T.E.; Palmenberg, A.C.; Gern, J.E.; Goldberg, T.L. | 2019 | Emerging Microbes and Infections | 10.1080/22221751.2018.1563456 |
| 839 | Missing teeth: Discordances in the trade of hippo ivory between Africa and Hong Kong | Andersson, A.; Gibson, L. | 2018 | African Journal of Ecology | 10.1111/aje.12441 |
| 840 | Best practice assessment of disease modelling for infectious disease outbreaks | Dembek, Z.F.; Chekol, T.; Wu, A. | 2018 | Epidemiology and Infection | 10.1017/S095026881800119X |
| 841 | Occupational health and safety hazards in agriculture: A study on the risks involved for the sustainability | Mishra, D.; Satapathy, S. | 2019 | Managing Operations Throughout Global Supply Chains | 10.4018/978-1-5225-8157-4.ch011 |
| 842 | Bioaerosol research: Yesterday, today and tomorrow | Zheng, Y.; Li, J.; Chen, H.; Zhang, T.; Li, X.; Wang, M.; Yao, M. | 2018 | Kexue Tongbao/Chinese Science Bulletin | 10.1360/N972018-00121 |
| 843 | Indication and Identification of Bacillus anthracis Isolates from the Middle Volga Region by Multi-Primer PCR | Aleksandrova, N.M.; Faizov, T.K.; Vasileva, A.V.; Rogozhina, I.A.; Khammadov, N.I.; Shuralev, E.A. | 2018 | BioNanoScience | 10.1007/s12668-017-0477-0 |
| 844 | Optimal control applied to a temperature dependent schistosomiasis model | Kalinda, C.; Mushayabasa, S.; Chimbari, M.J.; Mukaratirwa, S. | 2019 | BioSystems | 10.1016/j.biosystems.2018.11.008 |
| 845 | Pup Provisioning in the Cooperatively Breeding African Wild Dog, Lycaon pictus, is Driven by Pack Size, Social Status and Age | Forssman, K.R.; Marneweck, C.; O'riain, M.J.; Davies-Mostert, H.T.; Mills, M.G.L. | 2018 | African Journal of Wildlife Research | 10.3957/056.048.013005 |
| 846 | African Swine Fever Outbreak at a Farm in Central Namibia | Samkange, A.; Mushonga, B.; Mudimba, D.; Chiwome, B.A.; Jago, M.; Kandiwa, E.; Bishi, A.S.; Molini, U. | 2019 | Case Reports in Veterinary Medicine | 10.1155/2019/3619593 |
| 847 | A systematic review of human pathogens carried by the housefly (Musca domestica L.) | Khamesipour, F.; Lankarani, K.B.; Honarvar, B.; Kwenti, T.E. | 2018 | BMC Public Health | 10.1186/s12889-018-5934-3 |
| 848 | Ureide metabolism in plant-associated bacteria: purine plant-bacteria interactive scenarios under nitrogen deficiency | Izaguirre-Mayoral, M.L.; Lazarovits, G.; Baral, B. | 2018 | Plant and Soil | 10.1007/s11104-018-3674-x |
| 849 | Intellectual property and access to medicines in Africa: A regional framework for access | Owoeye, O. | 2019 | Intellectual Property and Access to Medicines in Africa: A Regional Framework for Access | 10.4324/9780429439186 |
| 850 | Modelling potential distribution of bramble (rubus cuneifolius) using topographic, bioclimatic and remotely sensed data in the KwaZulu-Natal Drakensberg, South Africa | Ndlovu, P.; Mutanga, O.; Sibanda, M.; Odindi, J.; Rushworth, I. | 2018 | Applied Geography | 10.1016/j.apgeog.2018.07.025 |
| 851 | Risk factors associated with the occurrence of anthrax outbreaks in livestock in the country of Georgia: A case-control investigation 2013-2015 | Rao, S.; Traxler, R.; Napetavaridze, T.; Asanishvili, Z.; Rukhadze, K.; Maghlakelidze, G.; Geleishvili, M.; Broladze, M.; Kokhreidze, M.; Reynolds, D.; Shadomy, S.; Salman, M. | 2019 | PLoS ONE | 10.1371/journal.pone.0215228 |
| 852 | A cutaneous Anthrax outbreak in Koraput District of Odisha-India 2015 | Nayak, P.; Sodha, S.V.; Laserson, K.F.; Padhi, A.K.; Swain, B.K.; Hossain, S.S.; Shrivastava, A.; Khasnobis, P.; Venkatesh, S.R.; Patnaik, B.; Dash, K.C. | 2019 | BMC Public Health | 10.1186/s12889-019-6787-0 |
| 853 | Prion-Like Propagation in Neurodegenerative Diseases | Peelaerts, W.; Baekelandt, V.; Brundin, P. | 2018 | The Molecular and Cellular Basis of Neurodegenerative Diseases: Underlying Mechanisms | 10.1016/B978-0-12-811304-2.00008-0 |
| 854 | Inflammation, aging and cancer: Biological injustices to molecular village of immunity that guard health | Khatami, M. | 2018 | Inflammation, Aging and Cancer: Biological Injustices to Molecular Village of Immunity that Guard Health | 10.1007/978-3-319-66475-0 |
| 855 | Case Studies in Public Health | Tulchinsky, T.H. | 2018 | Case Studies in Public Health | 10.1016/C2015-0-01739-7 |
| 856 | Mammalia: Proboscidea: Elephant immune system | Abegglen, L.M.; Fuery, A.; Kiso, W.K.; Schmitt, D.L.; Ling, P.D.; Schiffman, J.D. | 2018 | Advances in Comparative Immunology | 10.1007/978-3-319-76768-0_24 |
| 857 | Skin Infections | Prieto-Granada, C.N.; Lobo, A.Z.C.; Mihm, M.C. | 2018 | Diagnostic Pathology of Infectious Disease | 10.1016/B978-0-323-44585-6.00020-5 |
| 858 | Bovidae, antilocapridae, giraffidae, tragulidae, hippopotamidae | Jones, M.E.B.; Gasper, D.J.; Mitchelln√©e Lane, E. | 2018 | Pathology of Wildlife and Zoo Animals | 10.1016/B978-0-12-805306-5.00005-5 |
| 859 | Procyonidae, viverridae, hyenidae, herpestidae, eupleridae, and prionodontidae | Church, M.E.; Terio, K.A.; Keel, M.K. | 2018 | Pathology of Wildlife and Zoo Animals | 10.1016/B978-0-12-805306-5.00012-2 |
| 860 | Has doxycycline, in combination with anti-malarial drugs, a role to play in intermittent preventive treatment of Plasmodium falciparum malaria infection in pregnant women in Africa? | Gaillard, T.; Boxberger, M.; Madamet, M.; Pradines, B. | 2018 | Malaria Journal | 10.1186/s12936-018-2621-x |
| 861 | Surfen and oxalyl surfen decrease tau hyperphosphorylation and mitigate neuron deficits in vivo in a zebrafish model of tauopathy | Naini, S.M.A.; Yanicostas, C.; Hassan-Abdi, R.; Blondeel, S.; Bennis, M.; Weiss, R.J.; Tor, Y.; Esko, J.D.; Soussi-Yanicostas, N. | 2018 | Translational Neurodegeneration | 10.1186/s40035-018-0111-2 |
| 862 | Microbiological safety of street-vended foods in Bangladesh | Jahan, M.; Rahman, M.; Sikder, T.; Uson-Lopez, R.A.; Selim, A.S.M.; Saito, T.; Kurasaki, M. | 2018 | Journal fur Verbraucherschutz und Lebensmittelsicherheit | 10.1007/s00003-018-1174-9 |
| 863 | The calendar of epidemics: Seasonal cycles of infectious diseases | Martinez, M.E. | 2018 | PLoS Pathogens | 10.1371/journal.ppat.1007327 |
| 864 | Cultures of militarism: An introduction to supplement 19 | Gusterson, H.; Besteman, C. | 2019 | Current Anthropology | 10.1086/700648 |
| 865 | Intrasexually selected weapons | Rico-Guevara, A.; Hurme, K.J. | 2019 | Biological Reviews | 10.1111/brv.12436 |
| 866 | The ANC's war against apartheid: Umkhonto we Sizwe and the liberation of South Africa | Davis, S.R. | 2018 | The ANC's War against Apartheid: Umkhonto we Sizwe and the Liberation of South Africa |  |
| 867 | Pathogenicity, population genetics and dissemination of Bacillus anthracis | Pilo, P.; Frey, J. | 2018 | Infection, Genetics and Evolution | 10.1016/j.meegid.2018.06.024 |
| 868 | Use of chicken eggshell to improve dietary calcium intake in rural sub-Saharan Africa | Bartter, J.; Diffey, H.; Yeung, Y.H.; O'Leary, F.; H√§sler, B.; Maulaga, W.; Alders, R. | 2018 | Maternal and Child Nutrition | 10.1111/mcn.12649 |
| 869 | Canidae, ursidae, and ailuridae | Kevin Keel, M.; Terio, K.A.; McAloose, D. | 2018 | Pathology of Wildlife and Zoo Animals | 10.1016/B978-0-12-805306-5.00009-2 |
| 870 | Salivarian trypanosomosis: A review of parasites involved, their global distribution and their interaction with the innate and adaptive mammalian host immune system | Radwanska, M.; Vereecke, N.; Deleeuw, V.; Pinto, J.; Magez, S. | 2018 | Frontiers in Immunology | 10.3389/fimmu.2018.02253 |
| 871 | Prediction of potential suitable distribution of Davidia involucrata Baill in China based on MaxEnt | Wang, Y.-S.; Wang, Z.H.; Xing, H.-F.; Li, J.-W.; Sun, S. | 2019 | Chinese Journal of Ecology | 10.13292/j.1000-4890.201904.024 |
| 872 | Impact of chemorophylaxis policy for AIDS-immunocompromised patients on emergence of bacterial resistance | DeNegre, A.A.; Myers, K.; Fefferman, N.H. | 2020 | PLoS ONE | 10.1371/journal.pone.0225861 |
| 873 | Systematic review of important bacterial zoonoses in africa in the last decade in light of the 'one health' concept | El Zowalaty, M.E.; Asante, J.; Noreddin, A. | 2019 | Pathogens | 10.3390/pathogens8020050 |
| 874 | Risk factors for bovine anthrax in Bangladesh, 2010-2014: A case-control study | Rume, F.I.; Karim, M.R.; Ahsan, C.R.; Yasmin, M.; Biswas, P.K. | 2020 | Epidemiology and Infection | 10.1017/S0950268820000576 |
| 875 | Viral, bacterial, and fungal infections of the oral mucosa: Types, incidence, predisposing factors, diagnostic algorithms, and management | Bandara, H.M.H.N.; Samaranayake, L.P. | 2019 | Periodontology 2000 | 10.1111/prd.12273 |
| 876 | The family bombyliidae in the kingdom of Saudi Arabia (diptera: Brachycera: Asiloidea | El-Hawagry, M.S.; Al Dhafer, H.M. | 2019 | Zootaxa | 10.11646/zootaxa.4590.1.3 |
| 877 | Inorganic Complexes and Metal-Based Nanomaterials for Infectious Disease Diagnostics | Markwalter, C.F.; Kantor, A.G.; Moore, C.P.; Richardson, K.A.; Wright, D.W. | 2019 | Chemical Reviews | 10.1021/acs.chemrev.8b00136 |
| 878 | Overcoming the challenges of phage therapy for industrial aquaculture: A review | Culot, A.; Grosset, N.; Gautier, M. | 2019 | Aquaculture | 10.1016/j.aquaculture.2019.734423 |
| 879 | Veterinary importance and integrated management of Brachycera flies in dairy farms | Baldacchino, F.; Desquesnes, M.; Duvallet, G.; Lysyk, T.; Mihok, S. | 2018 | Ecology and Control of Vector-Borne Diseases | 10.3920/978-90-8686-863-6_3 |
| 880 | Rapid design and fielding of four diagnostic technologies in Sierra Leone, Thailand, Peru, and Australia: Successes and challenges faced introducing these biosensors | Mulvaney, S.P.; Fitzgerald, L.A.; Hamdan, L.J.; Ringeisen, B.R.; Petersen, E.R.; Compton, J.R.; McAuliff, N.L.; Leski, T.A.; Taitt, C.R.; Stenger, D.A.; Myers, C.A.; Hansen, E.; Ricketts, M.; Hoegberg, C.; Homdayjanakul, K.; Ansumana, R.; Lamin, J.M.; Bangura, U.; Lahai, J.; Baio, V.; Limmathurotsakul, D.; Wongsuvan, G.; Hantrakun, V.; Wacharapluesadee, S.; Mungaomklang, A.; Putcharoen, O.; Yatoom, P.; Kruthakool, K.; Hontz, R.D.; Mores, C.; Siles, C.; Morrison, A.; Mayo, M.; Currie, B.J.; Jacobsen, K.H.; Quinn, K.; Blutman, J.; Amariei, F.; Hannan, J. | 2018 | Sensing and Bio-Sensing Research | 10.1016/j.sbsr.2018.06.003 |
| 881 | Colony formation in the cyanobacterium Microcystis | Xiao, M.; Li, M.; Reynolds, C.S. | 2018 | Biological Reviews | 10.1111/brv.12401 |
| 882 | Meat inspection and hygiene in a Meat Factory Cell ‚Äì An alternative concept | Alvseike, O.; Prieto, M.; Torkveen, K.; Ruud, C.; Nesbakken, T. | 2018 | Food Control | 10.1016/j.foodcont.2018.02.014 |
| 883 | Duplex Lateral Flow Assay for the Simultaneous Detection of Yersinia pestis and Francisella tularensis | Jauset-Rubio, M.; Tomaso, H.; El-Shahawi, M.S.; Bashammakh, A.S.; Al-Youbi, A.O.; O'Sullivan, C.K. | 2018 | Analytical Chemistry | 10.1021/acs.analchem.8b03105 |
| 884 | Effects of vulture exclusion on carrion consumption by facultative scavengers | Hill, J.E.; DeVault, T.L.; Beasley, J.C.; Rhodes, O.E.; Belant, J.L. | 2018 | Ecology and Evolution | 10.1002/ece3.3840 |
| 885 | Investigating the effects of intervention strategies in a spatio-temporal anthrax model | Pantha, B.; Day, J.; Lenhart, S. | 2020 | Discrete and Continuous Dynamical Systems - Series B | 10.3934/dcdsb.2019242 |
| 886 | Promising bacterial genera for agricultural practices: An insight on plant growth-promoting properties and microbial safety aspects | Ferreira, C.M.H.; Soares, H.M.V.M.; Soares, E.V. | 2019 | Science of the Total Environment | 10.1016/j.scitotenv.2019.04.225 |
| 887 | Rothschild‚Äôs giraffe Giraffa camelopardalis rothschildi (Linnaeus, 1758) in East Africa: A review of population trends, taxonomy and conservation status | Muller, Z. | 2019 | African Journal of Ecology | 10.1111/aje.12578 |
| 888 | Review on nanoparticles and nanostructured materials: History, sources, toxicity and regulations | Jeevanandam, J.; Barhoum, A.; Chan, Y.S.; Dufresne, A.; Danquah, M.K. | 2018 | Beilstein Journal of Nanotechnology | 10.3762/bjnano.9.98 |
| 889 | Chromosomal and plasmid-mediated fluoroquinolone resistance in human Salmonella enterica infection in Ghana | Acheampong, G.; Owusu, M.; Owusu-Ofori, A.; Osei, I.; Sarpong, N.; Sylverken, A.; Kung, H.-J.; Cho, S.-T.; Kuo, C.-H.; Park, S.E.; Marks, F.; Adu-Sarkodie, Y.; Owusu-Dabo, E. | 2019 | BMC Infectious Diseases | 10.1186/s12879-019-4522-1 |
| 890 | Knowledge, attitude and practices relating to zoonotic diseases among livestock farmers in Punjab, India | Singh, B.B.; Kaur, R.; Gill, G.S.; Gill, J.P.S.; Soni, R.K.; Aulakh, R.S. | 2019 | Acta Tropica | 10.1016/j.actatropica.2018.09.021 |
| 891 | Flood and Infectious Disease Risk Assessment | Ashbolt, N.J. | 2018 | Health in Ecological Perspectives in the Anthropocene | 10.1007/978-981-13-2526-7_12 |
| 892 | Perissodactyls | Duncan, M. | 2018 | Pathology of Wildlife and Zoo Animals | 10.1016/B978-0-12-805306-5.00017-1 |
| 893 | Stability analysis model of Bacillus antracis using SEIQR population compartment with quarantine in Indonesia | Saptaningtyas, F.Y. | 2018 | Journal of Physics: Conference Series | 10.1088/1742-6596/983/1/012088 |
| 894 | Molecular investigation and phylogeny of species of the Anaplasmataceae infecting animals and ticks in Senegal | Dahmani, M.; Davoust, B.; Sambou, M.; Bassene, H.; Scandola, P.; Ameur, T.; Raoult, D.; Fenollar, F.; Mediannikov, O. | 2019 | Parasites and Vectors | 10.1186/s13071-019-3742-y |
| 895 | Hazards of a ‚Äòhealthy‚Äô trend? An appraisal of the risks of raw milk consumption and the potential of novel treatment technologies to serve as alternatives to pasteurization | Alegbeleye, O.O.; Guimar√£es, J.T.; Cruz, A.G.; Sant'Ana, A.S. | 2018 | Trends in Food Science and Technology | 10.1016/j.tifs.2018.10.007 |
| 896 | Multidrug-Resistant Bacteria and Alternative Methods to Control Them: An Overview | Vivas, R.; Barbosa, A.A.T.; Dolabela, S.S.; Jain, S. | 2019 | Microbial Drug Resistance | 10.1089/mdr.2018.0319 |
| 897 | Validation of the Canadian Fire Weather Index in Zimbabwe | Masocha, M.; Museva, T.; Dube, T. | 2018 | African Journal of Ecology | 10.1111/aje.12461 |
| 898 | Proboscidae | Landolfi, J.A.; Terrell, S.P. | 2018 | Pathology of Wildlife and Zoo Animals | 10.1016/B978-0-12-805306-5.00016-X |
| 899 | Sero-prevalence of chlamydiosis in cattle and selected wildlife species at a wildlife/livestock interface area of Zimbabwe | Ndengu, M.; Matope, G.; Tivapasi, M.; Scacchia, M.; Bonfini, B.; Pfukenyi, D.M.; de Garine-Wichatitsky, M. | 2018 | Tropical Animal Health and Production | 10.1007/s11250-018-1536-4 |
| 900 | Metabolic Potential and Biotechnological Importance of Plant Associated Endophytic Actinobacteria | Ganapathy, A.; Natesan, S. | 2018 | New and Future Developments in Microbial Biotechnology and Bioengineering: Actinobacteria: Diversity and Biotechnological Applications | 10.1016/B978-0-444-63994-3.00014-X |
| 901 | Influence of host and environmental factors on the distribution of the Japanese encephalitis vector culex tritaeniorhynchus in China | Liu, B.; Gao, X.; Ma, J.; Jiao, Z.; Xiao, J.; Wang, H. | 2018 | International Journal of Environmental Research and Public Health | 10.3390/ijerph15091848 |
| 902 | The conservation status of West African vultures: An updated review and a strategy for conservation | Divittorio, M.; Hema, E.M.; Dendi, D.; Akani, G.C.; Cortone, G.; Lopez-Lopez, P.; Amadi, N.; Segniagbeto, G.H.; Battistf, C.; Luiselli, L. | 2018 | Vie et Milieu |  |
| 903 | The White Redoubt, the Great Powers and the Struggle for Southern Africa, 1960‚Äì1980 |  | 2018 | Cambridge Imperial and Post-Colonial Studies |  |
| 904 | Potential distribution of dominant malaria vector species in tropical region under climate change scenarios | Akpan, G.E.; Adepoju, K.A.; Oladosu, O.R. | 2019 | PLoS ONE | 10.1371/journal.pone.0218523 |
| 905 | The Replication-Transmission Relativity Theory for Multiscale Modelling of Infectious Disease Systems | Garira, W. | 2019 | Scientific Reports | 10.1038/s41598-019-52820-3 |
| 906 | Predicting the geographic distribution of the bacillus anthracis A1.a/Western North American sub-lineage for the continental United States: New Outbreaks, New Genotypes, and New Climate Data | Yang, A.; Mullins, J.C.; Van Ert, M.; Bowen, R.A.; Hadfield, T.L.; Blackburn, J.K. | 2020 | American Journal of Tropical Medicine and Hygiene | 10.4269/ajtmh.19-0191 |
| 907 | Rabies of canid biotype in wild dog (Lycaon pictus) and spotted hyaena (Crocuta crocuta) in Madikwe Game Reserve, South Africa in 2014-2015: Diagnosis, possible origins and implications for control | Sabeta, C.T.; Janse Van Rensburg, D.D.; Phahladira, B.; Mohale, D.; Harrison-White, R.F.; Esterhuyzen, C.; Williams, J.H. | 2018 | Journal of the South African Veterinary Association | 10.4102/jsava.v89i0.1517 |
| 908 | Brucellosis awareness and knowledge in communities worldwide: A systematic review and meta-analysis of 79 observational studies | Zhang, N.; Zhou, H.; Huang, D.-S.; Guan, P. | 2019 | PLoS Neglected Tropical Diseases | 10.1371/journal.pntd.0007366 |
| 909 | The correspondence of Charles Darwin | Burkhardt, F.; Secord, J.A. | 2016 | The Correspondence of Charles Darwin | 10.1017/9781316848166 |
| 910 | Pittosporum viridiflorum Sims (Pittosporaceae): A review on a useful medicinal plant native to South Africa and tropical Africa | Madikizela, B.; McGaw, L.J. | 2017 | Journal of Ethnopharmacology | 10.1016/j.jep.2017.05.005 |
| 911 | Consuming or being consumed in the new world order? GMOs as an insult to the dispossessed and impoverished of the earth | Nhemachena, A.; Mawere, M. | 2017 | GMOs, Consumerism and the Global Politics of Biotechnology: Rethinking Food, Bodies and Identities in Africa's 21st Century |  |
| 912 | Cutaneous anthrax: evaluation of 28 cases in the Eastern Anatolian region of Turkey | Denk, A.; Tartar, A.S.; Ozden, M.; Demir, B.; Akbulut, A. | 2016 | Cutaneous and Ocular Toxicology | 10.3109/15569527.2015.1067818 |
| 913 | Effects of host traits and land-use changes on the gut microbiota of the Namibian black-backed jackal (Canis mesomelas) | Menke, S.; Meier, M.; Mfune, J.K.E.; Melzheimer, J.; Wachter, B.; Sommer, S. | 2017 | FEMS Microbiology Ecology | 10.1093/femsec/fix123 |
| 914 | Occurrence, heat and antibiotic resistance profile of Bacillus cereus isolated from raw cow and processed milk in Mezam Division, Cameroon | Tatsinkou Fossi, B.; Tatah Kihla Akoachere, J.-F.; Nchanji, G.T.; Wanji, S. | 2017 | International Journal of Dairy Technology | 10.1111/1471-0307.12315 |
| 915 | The primate zoonoses: Culture change and emerging diseases | Cormier, L.A.; Jolly, P.E. | 2017 | The Primate Zoonoses: Culture Change and Emerging Diseases | 10.4324/9781315414898 |
| 916 | Posthumanist critique and human health: how nonhumans (could) figure in public health research | Friese, C.; Nuyts, N. | 2017 | Critical Public Health | 10.1080/09581596.2017.1294246 |
| 917 | Molecular characterization and phylogenetic relatedness of dog-derived Rabies Viruses circulating in Cameroon between 2010 and 2016 | Sadeuh-Mba, S.A.; Momo, J.B.; Besong, L.; Loul, S.; Njouom, R. | 2017 | PLoS Neglected Tropical Diseases | 10.1371/journal.pntd.0006041 |
| 918 | Awareness, knowledge, and risks of zoonotic diseases among livestock farmers in Punjab | Hundal, J.S.; Sodhi, S.S.; Gupta, A.; Singh, J.; Chahal, U.S. | 2016 | Veterinary World | 10.14202/vetworld.2016.186-191 |
| 919 | Disorders of the Hematopoietic System | Dunkel, B. | 2018 | Equine Internal Medicine: Fourth Edition | 10.1016/B978-0-323-44329-6.00015-2 |
| 920 | Problematizing official narratives of HIV and AIDS education in Scotland and Zimbabwe | Nyatsanza, T.; Wood, L. | 2017 | Sahara J | 10.1080/17290376.2017.1394908 |
| 921 | Anthropology of infectious disease | Singer, M. | 2016 | Anthropology of Infectious Disease | 10.4324/9781315434735 |
| 922 | Disability and health outcomes‚Äìfrom a cohort of people on long-term anti-retroviral therapy | Myezwa, H.; Hanass-Hancock, J.; Ajidahun, A.T.; Carpenter, B. | 2018 | Sahara J | 10.1080/17290376.2018.1459813 |
| 923 | Reintroduction as an Antelope Conservation Solution | Price, M.R.S. | 2016 | Antelope Conservation: From Diagnosis to Action | 10.1002/9781118409572.ch12 |
| 924 | Assessment of community awareness and risk perceptions of zoonotic causes of abortion in cattle at three selected livestock-wildlife interface areas of Zimbabwe | Ndengu, M.; De Garine-Wichatitsky, M.; Pfukenyi, D.M.; Tivapasi, M.; Mukamuri, B.; Matope, G. | 2017 | Epidemiology and Infection | 10.1017/S0950268817000097 |
| 925 | Monitoring of indicator and multidrug resistant bacteria in agricultural soils under different irrigation patterns | Palacios, O.A.; Contreras, C.A.; Mu√±oz-Castellanos, L.N.; Gonz√°lez-Rangel, M.O.; Rubio-Arias, H.; Palacios-Espinosa, A.; Nev√°rez-Moorill√≥n, G.V. | 2017 | Agricultural Water Management | 10.1016/j.agwat.2017.01.001 |
| 926 | A Guide to AIDS | Bagasra, O.; Pace, D.G. | 2017 | A Guide to AIDS | 10.1201/b21840 |
| 927 | Characteristics of bacterial community in cloud water at Mt Tai: Similarity and disparity under polluted and non-polluted cloud episodes | Wei, M.; Xu, C.; Chen, J.; Zhu, C.; Li, J.; Lv, G. | 2017 | Atmospheric Chemistry and Physics | 10.5194/acp-17-5253-2017 |
| 928 | Anthrax, plague, diphtheria, trachoma, and miscellaneous bacteria | Tyring, S.K.; Burnett, M.; Mwesigye, F. | 2016 | Tropical Dermatology: Second Edition | 10.1016/B978-0-323-29634-2.00028-6 |
| 929 | Building global epidemiology and response capacity with field epidemiology training programs | Jones, D.S.; Dicker, R.C.; Fontaine, R.E.; Boore, A.L.; Omolo, J.O.; Ashgar, R.J.; Baggett, H.C. | 2017 | Emerging Infectious Diseases | 10.3201/eid2313.170509 |
| 930 | Can pastoral communities offer solutions for conserving the Endangered Grevy's zebra Equus grevyi at the periphery of its range? | Parker, G.E.; Davidson, Z.; Low, B.; Lalampaa, P.R.; Sundaresan, S.; Fischer, M. | 2017 | ORYX | 10.1017/S0030605315001325 |
| 931 | Insect biodiversity of the Socotra Archipelago ‚Äì Underlined and counted | Bezdƒõk, J.; H√°jek, J. | 2017 | Acta Entomologica Musei Nationalis Pragae | 10.1515/aemnp-2017-0105 |
| 932 | Carnivory in the common hippopotamus Hippopotamus amphibius: Implications for the ecology and epidemiology of anthrax in African landscapes | Dudley, J.P.; Hang'Ombe, B.M.; Leendertz, F.H.; Dorward, L.J.; de Castro, J.; Subalusky, A.L.; Clauss, M. | 2016 | Mammal Review | 10.1111/mam.12056 |
| 933 | Agriculture-nutrition linkages: A preliminary investigation for rural India | Balaji, S.J.; Jhajhria, A.; Kumar, S.; Immanuelraj, T.K.; Kar, A. | 2017 | Outlook on Agriculture | 10.1177/0030727017745909 |
| 934 | GREATER KUDU (TRAGELAPHUS STREPSICEROS) MORTALITY in EUROPEAN ZOOLOGICAL INSTITUTIONS: A RETROSPECTIVE STUDY | Leclerc, A.; Lamglait, B.; Petit, T.; Roman, Y.; Jebram, J. | 2016 | Journal of Zoo and Wildlife Medicine | 10.1638/2015-0214.1 |
| 935 | Animal models for the pathogenesis, treatment, and prevention of infection by bacillus anthracis | Welkos, S.; Bozue, J.A.; Twenhafel, N.; Cote, C.K. | 2016 | The Bacterial Spore: From Molecules to Systems | 10.1128/9781555819323.ch14 |
| 936 | Anthrax Vaccines | Friedlander, A.M.; Grabenstein, J.D.; Brachman, P.S. | 2017 | Plotkin's Vaccines | 10.1016/B978-0-323-35761-6.00011-0 |
| 937 | Non-Hepatotropic Viral, Bacterial, and Parasitic Infections of the Liver | Zaki, S.R.; Alves, V.A.F.; Hale, G.L. | 2018 | MacSween's Pathology of the Liver | 10.1016/B978-0-7020-6697-9.00007-8 |
| 938 | Arenaviruses | Bausch, D.G. | 2016 | Clinical Virology | 10.1128/9781555819439.ch45 |
| 939 | Prevalence of Bacillus anthracis spores in wool, hairs and habitat of small ruminants | Rajput, M.; Kamboh, A.A.; Dewani, P.; Umrani, A.P.; Abro, S.H.; Khan, M.A. | 2018 | Indian Journal of Animal Research | 10.18805/ijar.v0iOF.8496 |
| 940 | Lessons from a decade of individual-based models for infectious disease transmission: A systematic review (2006-2015) | Willem, L.; Verelst, F.; Bilcke, J.; Hens, N.; Beutels, P. | 2017 | BMC Infectious Diseases | 10.1186/s12879-017-2699-8 |
| 941 | A narrative review of dermatologic protocols for primary care medical service trips in Latin America and the Caribbean | Dainton, C.; Chu, C.H. | 2017 | International Journal of Dermatology | 10.1111/ijd.13816 |
| 942 | Consumer awareness and willingness to pay for safety of street foods in developing countries: A review | Alimi, B.A.; Workneh, T.S. | 2016 | International Journal of Consumer Studies | 10.1111/ijcs.12248 |
| 943 | Soil geochemical parameters influencing the spatial distribution of anthrax in Northwest Minnesota, USA | Nath, S.; Dere, A. | 2016 | Applied Geochemistry | 10.1016/j.apgeochem.2016.09.004 |
| 944 | Efficacy of 10-day Sitafloxacin-Containing Third-Line Rescue Therapies for Helicobacter pylori Strains Containing the gyrA Mutation | Mori, H.; Suzuki, H.; Matsuzaki, J.; Tsugawa, H.; Fukuhara, S.; Miyoshi, S.; Hirata, K.; Seino, T.; Matsushita, M.; Masaoka, T.; Kanai, T. | 2016 | Helicobacter | 10.1111/hel.12286 |
| 945 | Spatiotemporal patterns of clinical bovine dermatophilosis in Zimbabwe 1995‚Äì2014 | Ndhlovu, F.; Ndhlovu, D.N.; Chikerema, S.M.; Masocha, M.; Nyagura, M.; Pfukenyi, D.M. | 2017 | Onderstepoort Journal of Veterinary Research | 10.4102/ojvr.v84i1.1386 |
| 946 | Bushmeat and human health: Assessing the evidence in tropical and sub-tropical forests | Van Vliet, N.; Moreno, J.; G√≥mez, J.; Zhou, W.; Fa, J.E.; Golden, C.; N√≥brega Alves, R.R.; Nasi, R. | 2017 | Ethnobiology and Conservation | 10.15451/ec2017-04-6.3-1-45 |
| 947 | The use of spatial and spatiotemporal modeling for surveillance of H5N1 highly pathogenic avian influenza in poultry in the middle east | Alkhamis, M.; Hijmans, R.J.; Al-Enezi, A.; Mart√≠nez-L√≥pez, B.; Perea, A.M. | 2016 | Avian Diseases | 10.1637/11106-042115-Reg |
| 948 | The role of spatial analysis in risk-based animal disease management | Stevens, K.B.; Pfeiffer, D.U. | 2016 | Handbook of Spatial Epidemiology |  |
| 949 | Health and disease | Glass, G.E. | 2017 | Comprehensive Remote Sensing | 10.1016/B978-0-12-409548-9.10433-6 |
| 950 | Mapping the Distribution of Anthrax in Mainland China, 2005‚Äì2013 | Chen, W.-J.; Lai, S.-J.; Yang, Y.; Liu, K.; Li, X.-L.; Yao, H.-W.; Li, Y.; Zhou, H.; Wang, L.-P.; Mu, D.; Yin, W.-W.; Fang, L.-Q.; Yu, H.-J.; Cao, W.-C. | 2016 | PLoS Neglected Tropical Diseases | 10.1371/journal.pntd.0004637 |
| 951 | The darkest sides of politics, II: State terrorism, "weapons of mass destruction,? Religious extremism, and organized crime | Bale, J.M. | 2017 | The Darkest Sides of Politics, II: State Terrorism, "Weapons of Mass Destruction,? Religious Extremism, and Organized Crime |  |
| 952 | Serologic survey for canine distemper virus in freeranging wild canids in the northeast of Iran | Isfahani, M.H.S.; Rostami, A.; Bahonar, A.R.; Barin, A.; Memarian, I. | 2017 | Revue de Medecine Veterinaire |  |
| 953 | Ciprofloxacin | Kwong, J.; Lindsay Grayson, M. | 2017 | Kucers the Use of Antibiotics: A Clinical Review of Antibacterial, Antifungal, Antiparasitic, and Antiviral Drugs, Seventh Edition | 10.1201/9781315152110 |
| 954 | Trimethoprim and trimethoprim-sulfamethoxazole (cotrimoxazole) | Trubiano, J.A.; Lindsay Grayson, M. | 2017 | Kucers the Use of Antibiotics: A Clinical Review of Antibacterial, Antifungal, Antiparasitic, and Antiviral Drugs, Seventh Edition | 10.1201/9781315152110 |
| 955 | Application of system dynamics and participatory spatial group model building in animal health: A case study of East Coast Fever interventions in Lundazi and Monze districts of Zambia | Mumba, C.; Skjerve, E.; Rich, M.; Rich, K.M. | 2017 | PLoS ONE | 10.1371/journal.pone.0189878 |
| 956 | Comparative anatomy of the gastrointestinal tract in Eutheria: Taxonomy, biogeography and food volume 2: Laurasiatheria, general discussion | Langer, P.; Zachos, F.E. | 2017 | Comparative Anatomy of the Gastrointestinal Tract in Eutheria II: Taxonomy, Biogeography and Food. Laurasiatheria | 10.1515/9783110562217 |
| 957 | One Health: Competing perspectives in an emerging field | Kingsley, P.; Taylor, E.M. | 2017 | Parasitology | 10.1017/S0031182015001845 |
| 958 | Molecular characterization of tsetse‚Äôs proboscis and its response to Trypanosoma congolense infection | Awuoche, E.O.; Weiss, B.L.; Vigneron, A.; Mireji, P.O.; Aksoy, E.; Nyambega, B.; Attardo, G.M.; Wu, Y.; O‚ÄôNeill, M.; Murilla, G.; Aksoy, S. | 2017 | PLoS Neglected Tropical Diseases | 10.1371/journal.pntd.0006057 |
| 959 | Antelope Diseases - the Good, the Bad and the Ugly | Kock, R.; Chardonnet, P.; Risley, C. | 2016 | Antelope Conservation: From Diagnosis to Action | 10.1002/9781118409572.ch7 |
| 960 | Communal farmers' perceptions of tick-borne diseases affecting cattle and investigation of tick control methods practiced in Zimbabwe | Sungirai, M.; Moyo, D.Z.; De Clercq, P.; Madder, M. | 2016 | Ticks and Tick-borne Diseases | 10.1016/j.ttbdis.2015.07.015 |
| 961 | Microwave-accelerated method for ultra-rapid extraction of Neisseria gonorrhoeae DNA for downstream detection | Melendez, J.H.; Santaus, T.M.; Brinsley, G.; Kiang, D.; Mali, B.; Hardick, J.; Gaydos, C.A.; Geddes, C.D. | 2016 | Analytical Biochemistry | 10.1016/j.ab.2016.06.017 |
| 962 | An Aggregate of Four Anthrax Cases during the Dry Summer of 2011 in Epirus, Greece | Gaitanis, G.; Lolis, C.J.; Tsartsarakis, A.; Kalogeropoulos, C.; Leveidiotou-Stefanou, S.; Bartzokas, A.; Bassukas, I.D. | 2016 | Dermatology | 10.1159/000440860 |
| 963 | Consequences of animals crossing the edges of transfrontier parks | de Garine-Wichatitsky, M.; Fritz, H.; Chaminuka, P.; Caron, A.; Guerbois, C.; Pfukenyi, D.M.; Matema, C.; Jori, F.; Murwira, A. | 2017 | Transfrontier Conservation Areas: People Living on the Edge | 10.4324/9781315147376 |
| 964 | Practices of traditional beef farmers in their production and marketing of cattle in Zambia | Mumba, C.; H√§sler, B.; Muma, J.B.; Munyeme, M.; Sitali, D.C.; Skjerve, E.; Rich, K.M. | 2018 | Tropical Animal Health and Production | 10.1007/s11250-017-1399-0 |
| 965 | Roan antelope Hippotragus equinus in Africa: A review of abundance, threats and ecology | Havemann, C.P.; Retief, T.A.; Tosh, C.A.; de Bruyn, P.J.N. | 2016 | Mammal Review | 10.1111/mam.12061 |
| 966 | Light emission miracle in the sea and preeminent applications of bioluminescence in recent new biotechnology | Sharifian, S.; Homaei, A.; Hemmati, R.; Khajeh, K. | 2017 | Journal of Photochemistry and Photobiology B: Biology | 10.1016/j.jphotobiol.2017.05.021 |
| 967 | Seroprevalence of brucellosis in cattle and selected wildlife species at selected livestock/wildlife interface areas of the Gonarezhou National Park, Zimbabwe | Ndengu, M.; Matope, G.; de Garine-Wichatitsky, M.; Tivapasi, M.; Scacchia, M.; Bonfini, B.; Pfukenyi, D.M. | 2017 | Preventive Veterinary Medicine | 10.1016/j.prevetmed.2017.08.004 |
| 968 | A Mathematical Model of Anthrax Transmission in Animal Populations | Saad-Roy, C.M.; van den Driessche, P.; Yakubu, A.-A. | 2017 | Bulletin of Mathematical Biology | 10.1007/s11538-016-0238-1 |
| 969 | Predicting Disease Risk, Identifying Stakeholders, and Informing Control Strategies: A Case Study of Anthrax in Montana | Morris, L.R.; Blackburn, J.K. | 2016 | EcoHealth | 10.1007/s10393-016-1119-7 |
| 970 | The discovery, function and development of the variable number tandem repeats in different Mycobacterium species | Sun, Z.; Li, W.; Xu, S.; Huang, H. | 2016 | Critical Reviews in Microbiology | 10.3109/1040841X.2015.1022506 |
| 971 | Hands-on Approaches to Managing Antelopes and their Ecosystems: A South African Perspective | Knight, M.H.; Novellie, P.; Holness, S.; Du Toit, J.; Ferreira, S.; Hofmeyr, M.; Grant, C.; Herbst, M.; Gaylard, A. | 2016 | Antelope Conservation: From Diagnosis to Action | 10.1002/9781118409572.ch8 |
| 972 | Savanna elephant numbers are only a quarter of their expected values | Robson, A.S.; Trimble, M.J.; Purdon, A.; Young-Overton, K.D.; Pimm, S.L.; Van Aarde, R.J. | 2017 | PLoS ONE | 10.1371/journal.pone.0175942 |
| 973 | The Giraffe: Biology, ecology, evolution and behaviour | Shorrocks, B. | 2016 | The Giraffe: Biology, ecology, evolution and behaviour | 10.1002/9781118587430 |
| 974 | Molecular Epidemiology of Mycobacterium bovis in Humans and Cattle | El-Sayed, A.; El-Shannat, S.; Kamel, M.; Casta√±eda-Vazquez, M.A.; Casta√±eda-Vazquez, H. | 2016 | Zoonoses and Public Health | 10.1111/zph.12242 |
| 975 | Biological warfare agents: The history, pathophysiology, diagnosis, treatment and cautions | Erkekoƒülu, P.; Ko√áer-G√ºm√º≈üel, B. | 2018 | Fabad Journal of Pharmaceutical Sciences |  |
| 976 | Soft law and global health problems: Lessons from responses to HIV/AIDS, malaria and tuberculosis | Sekalala, S. | 2017 | Soft Law and Global Health Problems: Lessons from Responses to HIV/AIDS, Malaria and Tuberculosis | 10.1017/9781107278950 |
| 977 | Chemical diversity of wild populations of Elionurus muticus (Spreng.) and the allelopathic effect of its essential oil | F√ºller, T.N.; Bertrand, C.; Antonello, B.; Wesp, C.; de Barros, I.B.I.; Barbosa Neto, J.F. | 2017 | Journal of Essential Oil Research | 10.1080/10412905.2017.1313786 |
| 978 | Veterinary Medicine, Eleventh Edition | Constable, P.D.; Hinchcliff, K.W.; Done, S.H.; Gr√ºnberg, W. | 2016 | Veterinary Medicine, Eleventh Edition | 10.1016/B978-0-7020-5246-0.00027-9 |
| 979 | Germ wars: The politics of microbes and America's landscape of fear | Armstrong, M. | 2017 | Germ Wars: The Politics of Microbes and America's Landscape of Fear |  |
| 980 | Long-term population dynamics in a multi-species assemblage of large herbivores in East Africa | Kiffner, C.; Rheault, H.; Miller, E.; Scheetz, T.; Enriquez, V.; Swafford, R.; Kioko, J.; Prins, H.H.T. | 2017 | Ecosphere | 10.1002/ecs2.2027 |
| 981 | Redefining the Australian Anthrax Belt: Modeling the Ecological Niche and Predicting the Geographic Distribution of Bacillus anthracis | Barro, A.S.; Fegan, M.; Moloney, B.; Porter, K.; Muller, J.; Warner, S.; Blackburn, J.K. | 2016 | PLoS Neglected Tropical Diseases | 10.1371/journal.pntd.0004689 |
| 982 | Bioaerosols in the Earth system: Climate, health, and ecosystem interactions | Fr√∂hlich-Nowoisky, J.; Kampf, C.J.; Weber, B.; Huffman, J.A.; P√∂hlker, C.; Andreae, M.O.; Lang-Yona, N.; Burrows, S.M.; Gunthe, S.S.; Elbert, W.; Su, H.; Hoor, P.; Thines, E.; Hoffmann, T.; Despr√©s, V.R.; P√∂schl, U. | 2016 | Atmospheric Research | 10.1016/j.atmosres.2016.07.018 |
| 983 | Review article: Reducing uncertainty for acute febrile illness in resource-limited settings: The current diagnostic landscape | Robinson, M.L.; Manabe, Y.C. | 2017 | American Journal of Tropical Medicine and Hygiene | 10.4269/ajtmh.16-0667 |
| 984 | Understanding the failure of a behavior change intervention to reduce risk behaviors for avian influenza transmission among backyard poultry raisers in rural Bangladesh: A focused ethnography | Rimi, N.A.; Sultana, R.; Ishtiak-Ahmed, K.; Rahman, M.Z.; Hasin, M.; Islam, M.S.; Azziz-Baumgartner, E.; Nahar, N.; Gurley, E.S.; Luby, S.P. | 2016 | BMC Public Health | 10.1186/s12889-016-3543-6 |
| 985 | Conservation Challenges Facing African Savanna Ecosystems | Ford, A.T.; Fryxell, J.M.; Sinclair, A.R.E. | 2016 | Antelope Conservation: From Diagnosis to Action | 10.1002/9781118409572.ch2 |
| 986 | Antibody Production in Plants and Green Algae | Yusibov, V.; Kushnir, N.; Streatfield, S.J. | 2016 | Annual Review of Plant Biology | 10.1146/annurev-arplant-043015-111812 |
| 987 | Veterinary Epidemiology: Fourth Edition | Thrusfield, M.; Christley, R.; Brown, H.; Diggle, P.J.; French, N.; Howe, K.; Kelly, L.; O'Connor, A.; Sargeant, J.; Wood, H. | 2017 | Veterinary Epidemiology: Fourth Edition | 10.1002/9781118280249 |
| 988 | Assessment of gastrointestinal parasites in wild chimpanzees (Pan troglodytes troglodytes) in southeast Cameroon | Drakulovski, P.; Bertout, S.; Locatelli, S.; Butel, C.; Pion, S.; Mpoudi-Ngole, E.; Delaporte, E.; Peeters, M.; Malli√©, M. | 2014 | Parasitology Research | 10.1007/s00436-014-3904-y |
| 989 | Spatial methods for infectious disease outbreak investigations: Systematic literature review | Smith, C.M.; Le Comber, S.C.; Fry, H.; Bull, M.; Leach, S.; Hayward, A.C. | 2015 | Eurosurveillance | 10.2807/1560-7917.ES.2015.20.39.30026 |
| 990 | Bacteriophage diversity in different habitats and their role in pathogen control | Dafale, N.A.; Hathi, Z.J.; Bit, S.; Purohit, H.J. | 2016 | Microbial Factories: Biodiversity, Biopolymers, Bioactive Molecules: Volume 2 | 10.1007/978-81-322-2595-9_17 |
| 991 | One health: Science, politics and zoonotic disease in Africa | Bardosh, K. | 2016 | One Health: Science, Politics and Zoonotic Disease in Africa | 10.4324/9781315659749 |
| 992 | Diversity of Bacillus thuringiensis Crystal Toxins and Mechanism of Action | Adang, M.J.; Crickmore, N.; Jurat-Fuentes, J.L. | 2014 | Advances in Insect Physiology | 10.1016/B978-0-12-800197-4.00002-6 |
| 993 | Quinolones | Hooper, D.C.; Strahilevitz, J. | 2014 | Mandell, Douglas, and Bennett's Principles and Practice of Infectious Diseases | 10.1016/B978-1-4557-4801-3.00034-5 |
| 994 | The incidence of jackal bites and injuries in the Zagreb anti rabies clinic during the 1995-2014 period | Vodopija, R.; Racz, A.; Pahor, D. | 2016 | Acta Clinica Croatica | 10.20471/acc.2016.55.01.20 |
| 995 | Recent developments in anti-dotes against anthrax | Dhasmana, N.; Singh, L.K.; Bhaduri, A.; Misra, R.; Singh, Y. | 2015 | Recent Patents on Anti-Infective Drug Discovery | 10.2174/1574891X09666140830213925 |
| 996 | The History of Biological Weapons Use: What We Know and What We Don't | Carus, W.S. | 2015 | Health Security | 10.1089/hs.2014.0092 |
| 997 | Intraspecific 16S rRNA gene diversity among clinical isolates of Neisseria species | Mechergui, A.; Achour, W.; Hassen, A.B. | 2014 | APMIS | 10.1111/apm.12164 |
| 998 | WARFARE AND TRACKING IN AFRICA, 1952‚Äì1990 | Stapleton, T.J. | 2015 | Warfare and Tracking in Africa, 1952‚Äì1990 | 10.4324/9781315653716 |
| 999 | The contribution of veterinary medicine to public health and poverty reduction in developing countries | Muma, J.B.; Mwacalimba, K.K.; Munang'andu, H.M.; Matope, G.; Jenkins, A.; Siamudaala, V.; Mweene, A.S.; Marcotty, T. | 2014 | Veterinaria Italiana | 10.12834/VetIt.1405.323 |
| 1000 | Model highlights likely long-term influences of mesobrowsers versus those of elephants on woodland dynamics | O'Kane, C.A.J.; Duffy, K.J.; Page, B.R.; Macdonald, D.W. | 2014 | African Journal of Ecology | 10.1111/aje.12103 |
| 1001 | Zoonoses: Infectious diseases transmissible from animals to humans | Bauerfeind, R.; von Graevenitz, A.; Kimmig, P.; Schiefer, H.G.; Schwarz, T.; Slenczka, W.; Zahner, H. | 2015 | Zoonoses: Infectious Diseases Transmissible from Animals to Humans | 10.1128/9781555819262 |
| 1002 | Descriptive epidemiology of detected anthrax outbreaks in wild wood bison (bison bison athabascae) in northern Canada, 1962-2008 | Salb, A.; Stephen, C.; Ribble, C.; Elkin, B. | 2014 | Journal of Wildlife Diseases | 10.7589/2013-04-095 |
| 1003 | Designing a risk communication strategy for health hazards posed by traditional slaughter of goats in Tshwane, South Africa | Qekwana, D.N.; McCrindle, C.M.E.; Oguttu, J.W. | 2014 | Journal of the South African Veterinary Association | 10.4102/jsava.v85i1.1035 |
| 1004 | Protective immune response against Bacillus anthracis induced by intranasal introduction of a recombinant adenovirus expressing the protective antigen fused to the Fc-fragment of IgG2a | Shcherbinin, D.N.; Esmagambetov, I.B.; Noskov, A.N.; Selyaninov, Y.O.; Tutykhina, I.L.; Shmarov, M.M.; Logunov, D.Y.; Naroditskiy, B.S.; Gintsburg, A.L. | 2014 | Acta Naturae | 10.32607/20758251-2014-6-1-76-84 |
| 1005 | Managing global health security: The world health organization and disease outbreak control | Kamradt-Scott, A. | 2015 | Managing Global Health Security: The World Health Organization and Disease Outbreak Control | 10.1057/9781137520166 |
| 1006 | Screening for anthrax occurrence in soil of flooded rural areas in Poland after rainfalls in spring 2010 | Zasada, A.A.; Formi≈Ñska, K.; Ogrodnik, A.; Gierczy≈Ñski, R.; Jagielski, M. | 2014 | Annals of Agricultural and Environmental Medicine | 10.5604/12321966.1120584 |
| 1007 | Water Conflict Chronology | Gleick, P.H.; Heberger, M. | 2014 | The World's Water | 10.5822/978-1-61091-483-3_11 |
| 1008 | Possible Use of Bacteriophages Active against Bacillus anthracis and Other B. cereus Group Members in the Face of a Bioterrorism Threat | Jo≈Ñczyk-Matysiak, E.; K≈Çak, M.; Weber-DƒÖbrowska, B.; Borysowski, J.; G√≥rski, A. | 2014 | BioMed Research International | 10.1155/2014/735413 |
| 1009 | Arenaviruses: Lassa fever, Lujo hemorrhagic fever, lymphocytic choriomeningitis, and the south american hemorrhagic fevers | Bausch, D.G.; Mills, J.N. | 2014 | Viral Infections of Humans: Epidemiology and Control | 10.1007/978-1-4899-7448-8_8 |
| 1010 | Bovine dermatophilosis: Awareness, perceptions and attitudes in the small-holder sector of North-West Zimbabwe | Ndhlovu, D.N.; Masika, P.J. | 2016 | Onderstepoort Journal of Veterinary Research | 10.4102/ojvr.v83i1.1004 |
| 1011 | Large mammals in Ruaha National Park, Tanzania, dig for water when water stops flowing and water bacterial load increases | Stommel, C.; Hofer, H.; Grobbel, M.; East, M.L. | 2016 | Mammalian Biology | 10.1016/j.mambio.2015.08.005 |
| 1012 | Bushmeat and emerging infectious diseases: Lessons from Africa | Kurpiers, L.A.; Schulte-Herbr√ºggen, B.; Ejotre, I.; Reeder, D.M. | 2015 | Problematic Wildlife: A Cross-Disciplinary Approach | 10.1007/978-3-319-22246-2_24 |
| 1013 | New record of cannibalism in the common hippo, Hippopotamus amphibius (Linnaeus, 1758) | Dorward, L.J. | 2015 | African Journal of Ecology | 10.1111/aje.12197 |
| 1014 | Tabanids: Neglected subjects of research, but important vectors of disease agents! | Baldacchino, F.; Desquesnes, M.; Mihok, S.; Foil, L.D.; Duvallet, G.; Jittapalapong, S. | 2014 | Infection, Genetics and Evolution | 10.1016/j.meegid.2014.03.029 |
| 1015 | The Global Dispersion of Pathogenic Microorganisms by Dust Storms and Its Relevance to Agriculture | Gonzalez-Martin, C.; Teigell-Perez, N.; Valladares, B.; Griffin, D.W. | 2014 | Advances in Agronomy | 10.1016/B978-0-12-800131-8.00001-7 |
| 1016 | Research activities of Hokudai center for Zoonosis Control in Zambia | Higashi, H.; Kida, H. | 2014 | Journal of Disaster Research | 10.20965/jdr.2014.p0818 |
| 1017 | Bacillus anthracis (Anthrax) | Martin, G.J.; Friedlander, A.M. | 2014 | Mandell, Douglas, and Bennett's Principles and Practice of Infectious Diseases | 10.1016/B978-1-4557-4801-3.00209-5 |
| 1018 | Anaplasma marginale: Diversity, Virulence, and Vaccine Landscape through a Genomics Approach | Quiroz-Casta√±eda, R.E.; Amaro-Estrada, I.; Rodr√≠guez-Camarillo, S.D. | 2016 | BioMed Research International | 10.1155/2016/9032085 |
| 1019 | Ecological role of vertebrate scavengers | Beasley, J.C.; Olson, Z.H.; DeVault, T.L. | 2015 | Carrion Ecology, Evolution, and Their Applications | 10.1201/b18819 |
| 1020 | Vaccines against Bacterial Enteric Infections | Holmgren, J.; Levine, M.M. | 2015 | Mucosal Immunology: Fourth Edition | 10.1016/B978-0-12-415847-4.00056-2 |
| 1021 | Review of documented beak and feather disease virus cases in wild Cape parrots in South Africa during the last 20 years | Downs, C.T.; Brown, M.; Hart, L.; Symes, C.T. | 2015 | Journal of Ornithology | 10.1007/s10336-015-1258-6 |
| 1022 | Metagenomic analysis of the viral community in Namib Desert hypoliths | Adriaenssens, E.M.; Van Zyl, L.; De Maayer, P.; Rubagotti, E.; Rybicki, E.; Tuffin, M.; Cowan, D.A. | 2015 | Environmental Microbiology | 10.1111/1462-2920.12528 |
| 1023 | Development and evaluation of an ITS1 "Touchdown" PCR for assessment of drug efficacy against animal African trypanosomosis | Tran, T.; Napier, G.; Rowan, T.; Cordel, C.; Labuschagne, M.; Delespaux, V.; Van Reet, N.; Erasmus, H.; Joubert, A.; B√ºscher, P. | 2014 | Veterinary Parasitology | 10.1016/j.vetpar.2014.03.005 |
| 1024 | Microbiology of Drinking Water Production and Distribution | Bitton, G. | 2014 | Microbiology of Drinking Water Production and Distribution | 10.1002/9781118743942 |
| 1025 | Historical evolution of human anthrax from occupational disease to potentially global threat as bioweapon | D'Amelio, E.; Gentile, B.; Lista, F.; D'Amelio, R. | 2015 | Environment International | 10.1016/j.envint.2015.09.009 |
| 1026 | Human diseases from wildlife | Conover, M.R.; Vail, R.M. | 2014 | Human Diseases from Wildlife | 10.1201/b17428 |
| 1027 | Cattle Farmer Awareness and Behavior Regarding Prevention of Zoonotic Disease Transmission in Senegal | Tebug, S.F.; Kamga-Waladjo, A.R.; Ema, P.J.N.; Muyeneza, C.; Kane, O.; Seck, A.; Ly, M.T.; Lo, M. | 2015 | Journal of Agromedicine | 10.1080/1059924X.2015.1010068 |
| 1028 | Changing Patterns of Human Anthrax in Azerbaijan during the Post-Soviet and Preemptive Livestock Vaccination Eras | Kracalik, I.; Abdullayev, R.; Asadov, K.; Ismayilova, R.; Baghirova, M.; Ustun, N.; Shikhiyev, M.; Talibzade, A.; Blackburn, J.K. | 2014 | PLoS Neglected Tropical Diseases | 10.1371/journal.pntd.0002985 |
| 1029 | Climate change and animal health risk | Hiko, A.; Malicha, G. | 2016 | Advances in Sustainability and Environmental Justice | 10.1108/S2051-503020160000019004 |
| 1030 | Smallpox Inoculation (Variolation) in East Africa with Special Reference to the Practice Among the Boran and Gabra of Northern Kenya | Imperato, P.J.; Imperato, G.H. | 2014 | Journal of Community Health | 10.1007/s10900-014-9928-5 |
| 1031 | Investigation and control of anthrax outbreak at the human-animal interface, Bhutan, 2010 | Thapa, N.K.; Wangdi, K.; Dorji, T.; Dorjee, J.; Marston, C.K.; Hoffmaster, A.R. | 2014 | Emerging Infectious Diseases | 10.3201/eid2009.140181 |
| 1032 | African buffalo syncerus caffer (Sparrman, 1779) | Corn√©lis, D.; Melletti, M.; Korte, L.; Ryan, S.J.; Mirabile, M.; Prin, T.; Prins, H.H.T. | 2014 | Ecology, Evolution and Behaviour of Wild Cattle: Implications for Conservation | 10.1017/CBO9781139568098.022 |
| 1033 | Environmental and veterinary history - Some themes and suggested ways forward | Brown, K. | 2014 | Environment and History | 10.3197/096734014X14091313617361 |
| 1034 | Fossil primates | Cachel, S. | 2015 | Fossil Primates |  |
| 1035 | Systematic review of potential health risks posed by pharmaceutical, occupational and consumer exposures to metallic and nanoscale aluminum, aluminum oxides, aluminum hydroxide and its soluble salts | Willhite, C.C.; Karyakina, N.A.; Yokel, R.A.; Yenugadhati, N.; Wisniewski, T.M.; Arnold, I.M.F.; Momoli, F.; Krewski, D. | 2014 | Critical Reviews in Toxicology | 10.3109/10408444.2014.934439 |
| 1036 | Lysinibacillus sphaericus: Toxins and Mode of Action, Applications for Mosquito Control and Resistance Management | Silva Filha, M.H.N.L.; Berry, C.; Regis, L. | 2014 | Advances in Insect Physiology | 10.1016/B978-0-12-800197-4.00003-8 |
| 1037 | Electricity-free amplification and detection for molecular point-of-care diagnosis of HIV-1 | Singleton, J.; Osborn, J.L.; Lillis, L.; Hawkins, K.; Guelig, D.; Price, W.; Johns, R.; Ebels, K.; Boyle, D.; Weigl, B.; LaBarre, P. | 2014 | PLoS ONE | 10.1371/journal.pone.0113693 |
| 1038 | First Autochthonous Coinfected Anthrax in an Immunocompetent Patient | Afshar, P.; Hedayati, M.T.; Aslani, N.; Khodavaisy, S.; Babamahmoodi, F.; Mahdavi, M.R.; Dolatabadi, S.; Badali, H. | 2015 | Case Reports in Medicine | 10.1155/2015/325093 |
| 1039 | Meyler‚Äôs Side Effects of Drugs: The International Encyclopedia of Adverse Drug Reactions and Interactions, Sixteenth Edition | Aronson, J.K. | 2015 | Meyler's Side Effects of Drugs: The International Encyclopedia of Adverse Drug Reactions and Interactions |  |
| 1040 | The emerging threat of superwarfarins: history, detection, mechanisms, and countermeasures | Feinstein, D.L.; Akpa, B.S.; Ayee, M.A.; Boullerne, A.I.; Braun, D.; Brodsky, S.V.; Gidalevitz, D.; Hauck, Z.; Kalinin, S.; Kowal, K.; Kuzmenko, I.; Lis, K.; Marangoni, N.; Martynowycz, M.W.; Rubinstein, I.; van Breemen, R.; Ware, K.; Weinberg, G. | 2016 | Annals of the New York Academy of Sciences | 10.1111/nyas.13085 |
| 1041 | The effect of the tsetse fly on african development | Alsan, M. | 2015 | American Economic Review | 10.1257/aer.20130604 |
| 1042 | Comparison of Infectious Agents Susceptibility to Photocatalytic Effects of Nanosized Titanium and Zinc Oxides: A Practical Approach | Bogdan, J.; Zarzy≈Ñska, J.; P≈Çawi≈Ñska-Czarnak, J. | 2015 | Nanoscale Research Letters | 10.1186/s11671-015-1023-z |
| 1043 | Host-Associated metagenomics: A guide to generating infectious RNA Viromes | Temmam, S.; Monteil-Bouchard, S.; Robert, C.; Pascalis, H.; Michelle, C.; Jardot, P.; Charrel, R.; Raoult, D.; Desnues, C. | 2015 | PLoS ONE | 10.1371/journal.pone.0139810 |
| 1044 | Intracellular Calcium | Campbell, A.K. | 2014 | Intracellular Calcium | 10.1002/9781118675410 |
| 1045 | Failure of sterne- and pasteur-like strains of Bacillus anthracis to replicate and survive in the Urban bluebottle blow fly Calliphora vicina under laboratory conditions | Von Terzi, B.; Turnbull, P.C.B.; Bellan, S.E.; Beyer, W. | 2014 | PLoS ONE | 10.1371/journal.pone.0083860 |
| 1046 | Spatial and Temporal Patterns of Anthrax in White-Tailed Deer, Odocoileus virginianus, and Hematophagous Flies in West Texas during the Summertime Anthrax Risk Period | Blackburn, J.K.; Hadfield, T.L.; Curtis, A.J.; Hugh-Jones, M.E. | 2014 | Annals of the Association of American Geographers | 10.1080/00045608.2014.914834 |
| 1047 | UV-tolerant culturable bacteria in an Asian dust plume transported over the East China Sea | Hara, K.; Zhang, D.; Matsusaki, H.; Sadanaga, Y.; Ikeda, K.; Hanaoka, S.; Hatakeyama, S. | 2015 | Aerosol and Air Quality Research | 10.4209/aaqr.2014.03.0067 |
| 1048 | Plant-produced candidate countermeasures against emerging and reemerging infections and bioterror agents | Streatfield, S.J.; Kushnir, N.; Yusibov, V. | 2015 | Plant Biotechnology Journal | 10.1111/pbi.12475 |
| 1049 | Guerrilla healthcare innovation: creative resilience in Zimbabwe‚Äôs chimurenga, 1971‚Äì1980 | Mavhunga, C.C. | 2015 | History and Technology | 10.1080/07341512.2015.1129205 |
| 1050 | DISEASE RISK ANALYSIS - A TOOL for POLICY MAKING WHEN EVIDENCE IS LACKING: IMPORT of RABIES-SUSCEPTIBLE ZOO MAMMALS AS A MODEL | Hartley, M.; Roberts, H. | 2015 | Journal of Zoo and Wildlife Medicine | 10.1638/2015-0001.1 |
| 1051 | Clostridium: Pathogenic roles, industrial uses and medicinal prospects of natural products as ameliorative agents against pathogenic species | Num, S.M.; Useh, N.M. | 2014 | Jordan Journal of Biological Sciences | 10.12816/0008220 |
| 1052 | Quantification of microbial risks to human health caused by waterborne viruses and bacteria in an urban slum | Katukiza, A.Y.; Ronteltap, M.; van der Steen, P.; Foppen, J.W.A.; Lens, P.N.L. | 2014 | Journal of Applied Microbiology | 10.1111/jam.12368 |
| 1053 | Genomic research in Zambia: Confronting the ethics, policy and regulatory frontiers in the 21st Century | Chanda-Kapata, P.; Kapata, N.; Moraes, A.N.; Chongwe, G.; Munthali, J. | 2015 | Health Research Policy and Systems | 10.1186/s12961-015-0053-4 |
| 1054 | Antimicrobial Treatment for Systemic Anthrax: Analysis of Cases from 1945 to 2014 Identified through a Systematic Literature Review | Pillai, S.K.; Huang, E.; Guarnizo, J.T.; Hoyle, J.D.; Katharios-Lanwermeyer, S.; Turski, T.K.; Bower, W.A.; Hendricks, K.A.; Meaney-Delman, D. | 2015 | Health Security | 10.1089/hs.2015.0033 |
| 1055 | A sylvatic lifecycle of Echinococcus equinus in the Etosha National Park, Namibia | Wassermann, M.; Aschenborn, O.; Aschenborn, J.; Mackenstedt, U.; Romig, T. | 2015 | International Journal for Parasitology: Parasites and Wildlife | 10.1016/j.ijppaw.2014.12.002 |
| 1056 | Rainfall driven changes in behavioural responses confound measuring trends in lion population size | Young-Overton, K.D.; Funston, P.J.; Ferreira, S.M. | 2014 | Wildlife Biology | 10.2981/wlb.00015 |
| 1057 | Livestock and buffalo (syncerus caffer) interfaces in africa: Ecology of disease transmission and implications for conservation and development | Kock, R.; Kock, M.; de Garine-Wichatitsky, M.; Chardonnet, P.; Caron, A. | 2014 | Ecology, Evolution and Behaviour of Wild Cattle: Implications for Conservation | 10.1017/CBO9781139568098.028 |
| 1058 | Spatiotemporal clustering analysis and risk assessments of human cutaneous anthrax in China, 2005-2012 | Zhang, W.-Y.; Wang, L.-Y.; Zhang, X.-S.; Han, Z.-H.; Hu, W.-B.; Qian, Q.; Haque, U.; Magalhaes, R.J.S.; Li, S.-L.; Tong, S.-L.; Li, C.-Y.; Sun, H.-L.; Sun, Y.-S. | 2015 | PLoS ONE | 10.1371/journal.pone.0133736 |
| 1059 | A global perspective: Reframing the history of health, medicine, and disease | Harrison, M. | 2015 | Bulletin of the History of Medicine | 10.1353/bhm.2015.0116 |
