# Supplementary material for: Time to control of anthrax outbreaks in Africa, 2014–2023: A systematic review and meta-analysis
Source: PLOS Glob Public Health. 2025 Apr 22;5(4):e0004534. doi: 10.1371/journal.pgph.0004534 (PMC12013908; doi:10.1371/journal.pgph.0004534)
Supplement: S6 Table — (DOCX) [file pgph.0004534.s007.docx]

**S6 Table. Articles excluded at full-text screening with reasons for exclusion**

| # | Title | Authors | Year | Journal | DOI | Notes |
| --- | --- | --- | --- | --- | --- | --- |
| 1 | The importance of a One Health approach for prioritising zoonotic diseases to focus on capacity-building efforts in Uganda | Nantima N.; Ilukor J.; Kaboyo W.; Ademun A.R.O.; Muwanguzi D.; Sekamatte M.; Sentumbwe J.; Monje F.; Bwire G. | 2019 | Revue scientifique et technique (International Office of Epizootics) | https://dx.doi.org/10.20506/rst.38.1.2963 | Exclusion reason: Wrong study design; |
| 2 | Design and implementation of a secure open-source digital platform for animal health surveillance: The case of the Cameroon Animal Health Information System (CAHIS) | Feussom J.-M.K.; Kenlefack S.T.; Bourdanne B.; Tchamba E.M.K.; Ndalle H.G.N.; Zanga D.A.; Ndongo M.C.K.; Garga G. | 2023 | Virologie | https://dx.doi.org/10.1684/vir.2023.1011 | Exclusion reason: No full text; |
| 3 | The outbreak of anthrax in Nigeria: Re-enforcing one health | Aborode A.T.; Ojo-Akosile T.; Uwah E.A.; Ottoho E.; Ogunleye S.C.; Kamaldeen A.B.; Olabisi P.S.; Ayoola A.Y. | 2023 | New Microbes and New Infections | https://dx.doi.org/10.1016/j.nmni.2023.101189 | Exclusion reason: Wrong study design; |
| 4 | Anthrax toxins-producing Bacillus spp. isolated from handwashing stations during COVID-19 pandemic in Lagos, Nigeria | Bamidele T.A.; Odumosu B.T.; Adenola P.T.; Ameh J.; Kareem O.K.; Osoba B.; Ezechi O.C.; Salako B.L. | 2023 | Journal of Infection in Developing Countries | https://dx.doi.org/10.3855/jidc.18228 | Exclusion reason: Wrong outcomes; |
| 5 | Spatial patterns of anthrax outbreaks and cases among Livestock in Lesotho, 2005-2016 | Lepheana R.J.; Oguttu J.W.; Qekwana D.N. | 2020 | International Journal of Environmental Research and Public Health | https://dx.doi.org/10.3390/ijerph17207584 | Exclusion reason: Wrong study design; |
| 6 | Evaluation of the weekly disease surveillance system for epidemic-prone diseases in Makonde District, Zimbabwe 2020: a descriptive cross-sectional study | Madamombe K.; Karakadzai M.; Masoja G.; Dhliwayo T.; Juru T.; Chadambuka A.; Govha E.; Gombe N.; Tshimanga M. | 2022 | Pan African Medical Journal | https://dx.doi.org/10.11604/pamj.2022.43.132.35001 | Exclusion reason: Wrong study design; |
| 7 | Anthrax bio-surveillance of livestock in Arua District, Uganda, 2017-2018 | Omodo M.; Gardela J.; Namatovu A.; Okurut R.A.; Esau M.; Acham M.; Nakanjako M.F.; Israel M.; Isingoma E.; Moses M.; Paul L.; Ssenkeera B.; Atim S.A.; Gonahasa D.N.; Sekamatte M.; Gouilh M.A.; Gonzalez J.P. | 2023 | Acta Tropica | https://dx.doi.org/10.1016/j.actatropica.2023.106841 | Exclusion reason: Wrong study design |
| 8 | Anthrax hotspot mapping in Kenya support establishing a sustainable two-phase elimination program targeting less than 6% of the country landmass | Gachohi J.; Bett B.; Otieno F.; Mogoa E.; Njoki P.; Muturi M.; Mwatondo A.; Osoro E.; Ngere I.; Dawa J.; Nasimiyu C.; Oyas H.; Njagi O.; Canfield S.; Blackburn J.; Njenga K. | 2022 | Scientific reports | https://dx.doi.org/10.1038/s41598-022-24000-3 | Exclusion reason: Wrong study design; |
| 9 | A Retrospective Study on the Epidemiology of Anthrax Among Livestock from 2011 to 2020 in Awi Administrative Zone, Amhara Region, Northwest Ethiopia | Seyoum A.F.; Bitew A.B.; Negussie H. | 2022 | Veterinary Medicine: Research and Reports | https://dx.doi.org/10.2147/VMRR.S384794 | Exclusion reason: Wrong outcomes; |
| 10 | An Investigation into Major Sheep Diseases and Management Practices in North Shewa Zone, Oromia, Ethiopia | Birhanu T.; Debelu T.; Muhammed S.; Getachew F. | 2022 | Veterinary Medicine International | https://dx.doi.org/10.1155/2022/4868391 | Exclusion reason: Wrong study design; |
| 11 | Anthrax outbreak amidst the COVID-19 pandemic in Africa: Challenges and possible solutions | Badri R.; Uwishema O.; Wellington J.; Thambi V.D.; Pradhan A.U.; Adanur I.; Patrick Onyeaka C.V.; Onyeaka H. | 2022 | Annals of Medicine and Surgery | https://dx.doi.org/10.1016/j.amsu.2022.104418 | Exclusion reason: Wrong outcomes; |
| 12 | Outbreak of cutaneous anthrax associated with handling meat of dead cows in Southwestern Uganda, May 2018 | Musewa A.; Mirembe B.B.; Monje F.; Birungi D.; Nanziri C.; Aceng F.L.; Kabwama S.N.; Kwesiga B.; Ndumu D.B.; Nyakarahuka L.; Buule J.; Cossaboom C.M.; Lowe D.; Kolton C.B.; Marston C.K.; Stoddard R.A.; Hoffmaster A.R.; Ario A.R.; Zhu B.-P. | 2022 | Tropical Medicine and Health | https://dx.doi.org/10.1186/s41182-022-00445-0 | Exclusion reason: duplicate; |
| 13 | After-action review of rabies and anthrax outbreaks multisectoral response in Tanzania, challenges and lessons | Stephen K.J.; Assenga J.; Bernard J.; Eblate E.; Mwakapeje E.; Mghamba J.; Chinyuka H.; Kambarage D. | 2022 | Journal of Public Health in Africa | https://dx.doi.org/10.4081/jphia.2022.2023 | Exclusion reason: Wrong outcomes; |
| 14 | Towards an integrated surveillance of zoonotic diseases in Burkina Faso: the case of anthrax | Nana S.D.; Caffin J.-H.; Duboz R.; Antoine-Moussiaux N.; Binot A.; Diagbouga P.S.; Hendrikx P.; Bordier M. | 2022 | BMC public health | https://dx.doi.org/10.1186/s12889-022-13878-3 | Exclusion reason: Not an investigation of anthrax; |
| 15 | Socio-ecological systems analysis of the risk management 0f anthrax in Longido District, northern Tanzania | Sindato C.; Mchome Z.; Chengula A.; Ngingo B.L.; Mwanyika G.O.; Mremi I.R.; Tungu P.; Rumisha S.F.; Bwana V.; Mboera L.E.G. | 2022 | Tanzania Journal of Health Research | https://dx.doi.org/10.4314/thrb.v23i1.1S | Exclusion reason: Wrong outcomes; |
| 16 | After-action review of rabies and anthrax outbreaks multi-sectoral response in Tanzania, challenges and lessons | John K.; Assenga J.; Bernard J.; Eblate E.; Mwakapeje E.; Mghambai J.; Chinyuka H.; Kambarage D. | 2022 | BMC Proceedings | https://dx.doi.org/10.1186/s12919-022-00231-0 | Exclusion reason: Wrong study design; |
| 17 | Outbreak investigation of an unknown gastrointestinal illness in District Victoria, Country Mala, 2016 | Syed M.A.; Shumuye N.A.; Anyorikeya M.; Usenbaev N.; Mertens E.; Bellali H.; Rutebemberwa E. | 2021 | Pan African Medical Journal | https://dx.doi.org/10.11604/pamj.supp.2021.40.2.30992 | Exclusion reason: Wrong study design |
| 18 | Implementing epidemic intelligence in the WHO African region for early detection and response to acute public health events | Williams G.S.; Impouma B.; Mboussou F.; Lee T.M.-H.; Ogundiran O.; Okot C.; Metcalf T.; Stephen M.; Fekadu S.T.; Wolfe C.M.; Farham B.; Hofer C.; Wicht B.; Tores C.C.; Flahault A.; Keiser O. | 2021 | Epidemiology and Infection | https://dx.doi.org/10.1017/S095026882100114X | Exclusion reason: Wrong study design; |
| 19 | 'We dry contaminated meat to make it safe': An assessment of knowledge, attitude and practices on anthrax during an outbreak, Kisumu, Kenya, 2019 | Mugo B.C.; Lekopien C.; Owiny M. | 2021 | PLoS ONE | https://dx.doi.org/10.1371/journal.pone.0259017 | Exclusion reason: Wrong study design; |
| 20 | Integrated community based human and animal syndromic surveillance in Adadle district of the Somali region of Ethiopia | Osman Y.; Ali S.M.; Schelling E.; Tschopp R.; Hattendorf J.; Muhumed A.; Zinsstag J. | 2021 | One Health | https://dx.doi.org/10.1016/j.onehlt.2021.100334 | Exclusion reason: Not an investigation of anthrax; |
| 21 | Prioritization of neglected tropical zoonotic diseases: A one health perspective from Tigray region, Northern Ethiopia | Mersha T.T.; Wolde B.M.; Shumuye N.A.; Hailu A.B.; Mohammed A.H.; Redda Y.T.; Abera B.H.; Menghistu H.T. | 2021 | PLoS ONE | https://dx.doi.org/10.1371/journal.pone.0254071 | Exclusion reason: Wrong outcomes; |
| 22 | Assessment of knowledge, attitudes and practices towards anthrax in Narok County, Southern Kenya | Mbai J.M.; Omolo J.O.; Wamamba D.; Maritim D.; Gura Z.; Obonyo M. | 2021 | Pan African Medical Journal | https://dx.doi.org/10.11604/pamj.2021.38.120.19439 | Exclusion reason: Wrong outcomes |
| 23 | Cross-Sectoral Zoonotic Disease Surveillance in Western Kenya: Identifying Drivers and Barriers Within a Resource Constrained Setting | Thomas L.F.; Rushton J.; Bukachi S.A.; Falzon L.C.; Howland O.; Fevre E.M. | 2021 | Frontiers in Veterinary Science | https://dx.doi.org/10.3389/fvets.2021.658454 | Exclusion reason: Not an investigation of anthrax; |
| 24 | Cutaneous anthrax associated with handling carcasses of animals that died suddenly of unknown cause: Arua district, uganda, january 2015-august 2017 | Aceng F.L.; Ario A.R.; Alitubeera P.H.; Neckyon M.M.; Kadobera D.; Sekamatte M.; Okethwangu D.; Bulage L.; Harris J.R.; Nguma W.; Ndumu D.B.; Buule J.; Nyakarahuka L.; Zhu B.-P. | 2021 | PLoS Neglected Tropical Diseases | https://dx.doi.org/10.1371/journal.pntd.0009645 | Exclusion reason: Not an investigation of anthrax; |
| 25 | Digital biosurveillance for zoonotic disease detection in kenya | Keshavamurthy R.; Thumbi S.M.; Charles L.E. | 2021 | Pathogens | https://dx.doi.org/10.3390/pathogens10070783 | Exclusion reason: Not an investigation of anthrax; |
| 26 | Spatial clustering of livestock Anthrax events associated with agro-ecological zones in Kenya, 1957-2017 | Nderitu L.M.; Gachohi J.; Otieno F.; Mogoa E.G.; Muturi M.; Mwatondo A.; Osoro E.M.; Ngere I.; Munyua P.M.; Oyas H.; Njagi O.; Lofgren E.; Marsh T.; Widdowson M.-A.; Bett B.; Njenga M.K. | 2021 | BMC Infectious Diseases | https://dx.doi.org/10.1186/s12879-021-05871-9 | Exclusion reason: Wrong outcomes; |
| 27 | Multisectoral cost analysis of a human and livestock anthrax outbreak in Songwe Region, Tanzania (December 2018-January 2019), using a novel Outbreak Costing Tool | Bodenham R.F.; Mtui-Malamsha N.; Gatei W.; Woldetsadik M.A.; Cassell C.H.; Salyer S.J.; Halliday J.E.B.; Nonga H.E.; Swai E.S.; Makungu S.; Mwakapeje E.; Bernard J.; Bebay C.; Makonnen Y.J.; Fasina F.O. | 2021 | One Health | https://dx.doi.org/10.1016/j.onehlt.2021.100259 | Exclusion reason: Wrong outcomes; |
| 28 | The pattern of anthrax at the wildlifelivestock-human interface in Zimbabwe | Mukarati N.L.; Matope G.; de Garine-Wichatitsky M.; Ndhlovu D.N.; Caron A.; Pfukenyi D.M. | 2020 | PLoS Neglected Tropical Diseases | https://dx.doi.org/10.1371/journal.pntd.0008800 | Exclusion reason: Wrong outcomes; |
| 29 | Serological evidence for human exposure to Bacillus cereus biovar anthracis in the villages around Tai National Park, Cote d'Ivoire | Dupke S.; Schubert G.; Beudje F.; Barduhn A.; Pauly M.; Couacy-Hymann E.; Grunow R.; Akoua-Koffi C.; Leendertz F.H.; Klee S.R. | 2020 | PLoS Neglected Tropical Diseases | https://dx.doi.org/10.1371/journal.pntd.0008292 | Exclusion reason: Wrong study design; |
| 30 | Epidemiological and laboratory investigation of a zoonotic anthrax outbreak in Ngenge Kween district, Uganda in 2018 | Mayanja F.; Nanfuka M.; Bahati M. | 2020 | International Journal of Infectious Diseases | https://dx.doi.org/10.1016/j.ijid.2020.11.114 | Exclusion reason: No full text |
| 31 | Successes and challenges of the One Health approach in Kenya over the last decade | Munyua P.M.; Njenga M.K.; Osoro E.M.; Onyango C.O.; Bitek A.O.; Mwatondo A.; Muturi M.K.; Musee N.; Bigogo G.; Otiang E.; Ade F.; Lowther S.A.; Breiman R.F.; Neatherlin J.; Montgomery J.; Widdowson M.-A. | 2019 | BMC public health | https://dx.doi.org/10.1186/s12889-019-6772-7 | Exclusion reason: Wrong outcomes; |
| 32 | One Health collaborations for zoonotic disease control in Ethiopia | Murphy S.C.; Negron M.E.; Pieracci E.G.; Deressa A.; Bekele W.; Regassa F.; Wassie B.A.; Afera B.; Hajito K.W.; Walelign E.; Abebe G.; Newman S.; Rwego I.B.; Mutonga D.; Gulima D.; Kebede N.; Smith W.A.; Kramer L.M.; Kibria A.; Bonnenfant Y.T.; Mortenson J.A.; Vieira A.R.; Kadzik M.; Sugerman D.; Amare B.; Kanter T.; Walke H.; Belay E.; Gallagher K. | 2019 | Revue scientifique et technique (International Office of Epizootics) | https://dx.doi.org/10.20506/rst.38.1.2940 | Exclusion reason: Wrong study design; |
| 33 | Temporal and spatial distribution of anthrax outbreaks among Kenyan wildlife, 1999-2017 | Gachohi J.M.; Gakuya F.; Lekolool I.; Osoro E.; Nderitu L.; Munyua P.; Ngere I.; Kemunto N.; Bett B.; Otieno F.; Muturi M.; Mwatondo A.; Widdowson M.A.; Kariuki Njenga M. | 2019 | Epidemiology and Infection | https://dx.doi.org/10.1017/S0950268819001304 | Exclusion reason: Not an investigation of anthrax |
| 34 | Assessing health systems in Guinea for prevention and control of priority zoonotic diseases: A One Health approach | Standley C.J.; Carlin E.P.; Sorrell E.M.; Barry A.M.; Bile E.; Diakite A.S.; Keita M.S.; Koivogui L.; Mane S.; Martel L.D.; Katz R. | 2019 | One Health | https://dx.doi.org/10.1016/j.onehlt.2019.100093 | Exclusion reason: Wrong study design; |
| 35 | Risk mapping and eco-anthropogenic assessment of anthrax in the upper Zambezi basin | Kamboyi H.K.; de Garine-Wichatitsky M.; Hang'ombe M.B.; Munyeme M. | 2019 | Veterinary medicine and science | https://dx.doi.org/10.1002/vms3.168 | Exclusion reason: Wrong outcomes; |
| 36 | Anthrax-like disease outbreak in cattle in West Africa from atypical Bacillus species: Preliminary report | Idachaba S.; Rimfa A.; Abiayi E.; Dashe Y.; Agada G.; Odugbo M. | 2019 | European Journal of Immunology | https://dx.doi.org/10.1002/eji.201970400 | Exclusion reason: No full text |
| 37 | Risk factors for human cutaneous anthrax outbreaks in the hot-spot districts of Northern Tanzania: An unmatched case-control study | Mwakapeje E.R.; Hogset S.; Softic A.; Mghamba J.; Nonga H.E.; Mdegela R.H.; Skjerve E. | 2019 | Transactions of the Royal Society of Tropical Medicine and Hygiene | https://dx.doi.org/10.1093/trstmh/trz090 | Exclusion reason: Wrong study design; |
| 38 | Interventions for prevention and control of anthrax according to the one health approach in South Omo zone, Ethiopia | Braus A.; Schug A.R.; Asrat A.; Adamu A.; Regassa G. | 2019 | Transactions of the Royal Society of Tropical Medicine and Hygiene | https://dx.doi.org/10.1093/trstmh/trz090 | Exclusion reason: No full text; |
| 39 | Evaluation of main infectious diseases outbreak detection and response timeliness in Southeast European Region | Mersini K.; Sulo J.; Agolli L.; Vasili A.; Musa S.; Kunchev A.; Mikik V.; Humolli I.; Kalaveshi A.; Crilly J.; Crawley A.; Bino S. | 2019 | International Journal of Infectious Diseases | https://dx.doi.org/10.1016/j.ijid.2018.11.315 | Exclusion reason: Wrong setting; |
| 40 | Anthrax outbreaks in the humans - livestock and wildlife interface areas of Northern Tanzania: a retrospective record review 2006-2016 | Mwakapeje E.R.; Hogset S.; Fyumagwa R.; Nonga H.E.; Mdegela R.H.; Skjerve E. | 2018 | BMC public health | https://dx.doi.org/10.1186/s12889-017-5007-z | Exclusion reason: Not an investigation of anthrax |
| 41 | Investigation of anthrax in an endemic region in Kenya: a mixed methods approach | Obonyo M.O.; Farr M.; Hikufe E.H.; Rubanzana W.; Omondi Owiny M.; Roka Z.G. | 2018 | The Pan African medical journal | https://dx.doi.org/10.11604/pamj.supp.2018.30.1.15279 | Exclusion reason: Wrong study design; |
| 42 | A serological survey of anthrax in domestic dogs in Zimbabwe: A potential tool for anthrax surveillance | Mukarati N.L.; Ndumnego O.; Van Heerden H.; Ndhlovu D.N.; Matope G.; Caron A.; De Garine-Wichatitsky M.; Pfukenyi D.M. | 2018 | Epidemiology and Infection | https://dx.doi.org/10.1017/S0950268818001577 | Exclusion reason: Wrong outcomes; |
| 43 | Recurrent Anthrax Outbreaks in Humans, Livestock, and Wildlife in the Same Locality, Kenya, 2014-2017 | Muturi M.; Gachohi J.; Mwatondo A.; Lekolool I.; Gakuya F.; Bett A.; Osoro E.; Bitek A.; Thumbi S.M.; Munyua P.; Oyas H.; Njagi O.N.; Bett B.; Njenga M.K. | 2018 | American Journal of Tropical Medicine and Hygiene | https://dx.doi.org/10.4269/ajtmh.18-0224 | Exclusion reason: Not an investigation of anthrax |
| 44 | Mapping the epidemiological distribution and incidence of major zoonotic diseases in South Tigray, North Wollo and Ab'ala (Afar), Ethiopia | Menghistu H.T.; Hailu K.T.; Shumye N.A.; Redda Y.T. | 2018 | PLoS ONE | https://dx.doi.org/10.1371/journal.pone.0209974 | Exclusion reason: Out of study time; |
| 45 | Spatio-temporal epidemiology of anthrax in Hippopotamus amphibious in Queen Elizabeth protected area, Uganda | Driciru M.; Rwego I.B.; Asiimwe B.; Travis D.A.; Alvarez J.; VanderWaal K.; Pelican K. | 2018 | PLoS ONE | https://dx.doi.org/10.1371/journal.pone.0206922 | Exclusion reason: Wrong outcomes; |
| 46 | Temporal patterns of anthrax outbreaks among livestock in Lesotho, 2005-2016 | Lepheana R.J.; Oguttu J.W.; Qekwana D.N. | 2018 | PLoS ONE | https://dx.doi.org/10.1371/journal.pone.0204758 | Exclusion reason: Wrong outcomes; |
| 47 | The reporting of a Bacillus anthracis B-clade strain in South Africa after more than 20 years | Lekota K.E.; Hassim A.; Rogers P.; Dekker E.H.; Last R.; de Klerk-Lorist L.; van Heerden H. | 2018 | BMC research notes | https://dx.doi.org/10.1186/s13104-018-3366-x | Exclusion reason: Wrong outcomes; |
| 48 | Role of food insecurity in outbreak of anthrax infections among humans and hippopotamuses living in a game reserve area, rural Zambia | Lehman M.W.; Craig A.S.; Malama C.; Kapina-Kany'anga M.; Malenga P.; Munsaka F.; Muwowo S.; Shadomy S.; Marx M.A. | 2017 | Emerging Infectious Diseases | https://dx.doi.org/10.3201/eid2309.161597 | Exclusion reason: Wrong outcomes; |
| 49 | A retrospective study of anthrax on the Ghaap Plateau, Northern Cape province of South Africa, with special reference to the 2007-2008 outbreaks | Hassim A.; Dekker E.H.; Byaruhanga C.; Reardon T.; Van Heerden H. | 2017 | The Onderstepoort journal of veterinary research | https://dx.doi.org/10.4102/ojvr.v84i1.1414 | Exclusion reason: Out of study time; |
| 50 | Mapping as a tool for predicting the risk of anthrax outbreaks in northern region of Ghana | Nsoh A.E.; Kenu E.; Forson E.K.; Afari E.; Sackey S.; Nyarko K.M.; Yebuah N. | 2016 | Pan African Medical Journal | https://dx.doi.org/10.11604/PAMJ.SUPP.2016.25.1.6205 | Exclusion reason: Wrong outcomes; |
| 51 | Detection and management of the first human anthrax outbreak in Togo | Patassi A.A.; Saka B.; Landoh D.E.; Agbenoko K.; Tamekloe T.; Salmon-Ceron D. | 2016 | Tropical Doctor | https://dx.doi.org/10.1177/0049475515622331 | Exclusion reason: Not an investigation of anthrax |
| 52 | Prioritizing zoonotic diseases in Ethiopia using a one health approach | Pieracci E.G.; Hall A.J.; Gharpure R.; Haile A.; Walelign E.; Deressa A.; Bahiru G.; Kibebe M.; Walke H.; Belay E. | 2016 | One Health | https://dx.doi.org/10.1016/j.onehlt.2016.09.001 | Exclusion reason: Wrong study design; |
| 53 | 2014 Anthrax epidemic in Koubia prefecture, Guinea-Conakry | Sow M.S.; Boushab M.B.; Balde H.; Camara A.; Sako F.B.; Traore F.A.; Diallo M.O.; Diallo M.D.; Keita M.; Sylla A.O.; Tounkara T.M.; Cisse M. | 2016 | Medecine et sante tropicales | https://dx.doi.org/10.1684/mst.2016.0635 | Exclusion reason: Wrong outcomes |
| 54 | A One Health, participatory epidemiology assessment of anthrax (Bacillus anthracis) management in Western Uganda | Coffin J.L.; Monje F.; Asiimwe-Karimu G.; Amuguni H.J.; Odoch T. | 2015 | Social Science and Medicine | https://dx.doi.org/10.1016/j.socscimed.2014.07.037 | Exclusion reason: Wrong outcomes; |
| 55 | Is Uganda a hub for zoonotic disease outbreaks? Lessons and challenges from ebola, marburg, yellow fever and anthrax outbreaks | Nabukenya I.; Lukwago L.; Okot C.; Wamala J.F.; Malimbo M.; Namukose E.M.; Musoke R.; Nanyunja M.; Makumbi I. | 2014 | International Journal of Infectious Diseases | https://dx.doi.org/10.1016/j.ijid.2014.03.916 | Exclusion reason: Wrong study design; |
| 56 | Anthrax outbreak at disease investigation farm, Techiman, Ghana, 2013 | Edward F.D.; Osei-Tutu A.; Gbeddy K.; Tsitsiwu J.; Quist C. | 2014 | International Journal of Infectious Diseases | https://dx.doi.org/10.1016/j.ijid.2014.03.727 | Exclusion reason: Out of study time; |
| 57 | Anthrax as an example of the One Health concept | Bengis R.G.; Frean J. | 2014 | Revue scientifique et technique (International Office of Epizootics) | https://www.ncbi.nlm.nih.gov/pubmed/?term=25707186 | Exclusion reason: Wrong outcomes; |
| 58 | Drivers and potential distribution of anthrax occurrence and incidence at national and sub-county levels across Kenya from 2006 to 2020 using INLA. | Ndolo, Valentina A; Redding, David William; Lekolool, Isaac; Mwangangi, David Mumo; Odhiambo, David Onyango; Deka, Mark A; Conlan, Andrew J K; Wood, James L N | 2022 | Scientific reports | https://dx.doi.org/10.1038/s41598-022-24589-5 | Exclusion reason: Wrong study design |
| 59 | Enhancing Surveillance and Diagnostics in Anthrax-Endemic Countries. | Vieira, Antonio R; Salzer, Johanna S; Traxler, Rita M; Hendricks, Katherine A; Kadzik, Melissa E; Marston, Chung K; Kolton, Cari B; Stoddard, Robyn A; Hoffmaster, Alex R; Bower, William A; Walke, Henry T | 2017 | Emerging infectious diseases | https://dx.doi.org/10.3201/eid2313.170431 | Exclusion reason: Wrong study design; |
| 60 | Persistent anthrax as a major driver of wildlife mortality in a tropical rainforest. | Hoffmann, Constanze; Zimmermann, Fee; Biek, Roman; Kuehl, Hjalmar; Nowak, Kathrin; Mundry, Roger; Agbor, Anthony; Angedakin, Samuel; Arandjelovic, Mimi; Blankenburg, Anja; Brazolla, Gregory; Corogenes, Katherine; Couacy-Hymann, Emmanuel; Deschner, Tobias; Dieguez, Paula; Dierks, Karsten; Dux, Ariane; Dupke, Susann; Eshuis, Henk; Formenty, Pierre; Yuh, Yisa Ginath; Goedmakers, Annemarie; Gogarten, Jan F; Granjon, Anne-Celine; McGraw, Scott; Grunow, Roland; Hart, John; Jones, Sorrel; Junker, Jessica; Kiang, John; Langergraber, Kevin; Lapuente, Juan; Lee, Kevin; Leendertz, Siv Aina; Leguillon, Floraine; Leinert, Vera; Lohrich, Therese; Marrocoli, Sergio; Matz-Rensing, Kerstin; Meier, Amelia; Merkel, Kevin; Metzger, Sonja; Murai, Mizuki; Niedorf, Svenja; De Nys, Helene; Sachse, Andreas; van Schijndel, Joost; Thiesen, Ulla; Ton, Els; Wu, Doris; Wieler, Lothar H; Boesch, Christophe; Klee, Silke R; Wittig, Roman M; Calvignac-Spencer, Sebastien; Leendertz, Fabian H | 2017 | Nature | https://dx.doi.org/10.1038/nature23309 | Exclusion reason: Wrong outcomes; |
| 61 | Anthrax toxins-producing<i> Bacillus</i> spp. isolated from handwashing stations during COVID-19 pandemic in Lagos, Nigeria | Bamidele, TA; Odumosu, B; Adenola, P; Ameh, J; Kareem, OK; Osoba, B; Ezechi, OC; Salako, BL | 2023 | JOURNAL OF INFECTION IN DEVELOPING COUNTRIES | https://dx.doi.org/10.3855/jidc.18228 | Exclusion reason: Wrong outcomes; |
| 62 | Investigation on Anthrax in Bangladesh during the Outbreaks of 2011 and Definition of the Epidemiological Correlations | Galante, D; Manzulli, V; Serrecchia, L; Di Taranto, P; Hugh-Jones, M; Hossain, MJ; Rondinone, V; Cipolletta, D; Pace, L; Iatarola, M; Tolve, F; Aceti, A; Poppa, E; Fasanella, A | 2021 | PATHOGENS | https://dx.doi.org/10.3390/pathogens10040481 | Exclusion reason: Wrong setting; |
| 63 | Anthrax-like disease outbreak in cattle in West Africa from atypical <i>Bacillus</i> species: preliminary report | Idachaba, S; Rimfa, A; Abiayi, E; Dashe, Y; Agada, G; Odugbo, M | 2019 | EUROPEAN JOURNAL OF IMMUNOLOGY | Not found | Exclusion reason: Wrong study design; |
| 64 | Early Detection of Emerging Zoonotic Diseases with Animal Morbidity and Mortality Monitoring | Bisson, IA; Ssebide, BJ; Marra, PP | 2015 | ECOHEALTH | https://dx.doi.org/10.1007/s10393-014-0988-x | Exclusion reason: Not an investigation of anthrax; |
| 65 | Risk factors for human anthrax outbreak in Kiruhura District, Southwestern Uganda: a population-based case control study | Migisha, R.; Mbatidde, I.; Agaba, D.C.; Turyakira, E.; Tumwine, G.; Byaruhanga, A.; Siya, A.; Ruzaaza, G.N.; Kirunda, H. | 2021 | Pan African Medical Journal One Health | https://dx.doi.org/10.11604/pamj-oh.2021.5.13.29385 | Exclusion reason: Wrong outcomes; |
